# Manipulating Unisexual–Sexual Reproduction Transition to Engineer Genome‐Reconstructed Polyploids

**DOI:** 10.1002/advs.202506024

**Published:** 2025-07-11

**Authors:** Meng Lu, Qin‐Can Zhang, Zi‐Yu Zhu, Yang Wang, Zhong‐Wei Wang, Xi‐Yin Li, Zhi Li, Xiao‐Juan Zhang, Li Zhou, Jian‐Fang Gui

**Affiliations:** ^1^ State Key Laboratory of Breeding Biotechnology and Sustainable Aquaculture Hubei Hongshan Laboratory The Innovation Academy of Seed Design Institute of Hydrobiology Chinese Academy of Sciences Wuhan 430072 China; ^2^ University of Chinese Academy of Sciences Beijing 100049 China

**Keywords:** unisexual reproduction, polyploidy, herpesvirus resistance, trait fixation, polyploid genome design

## Abstract

Both unisexuality and polyploidy are significant in agriculture, exhibiting revolutionary biotechnology potential, as their coupling has been demonstrated to enable the design of polyploid genomes in crops. However, their applicability to animals has remained a challenge. Herein, the first case of engineering polyploid genomes with desirable traits via unisexual–sexual reproduction transition is provided. First, a group of genome‐reconstructed amphitriploids (GR‐A3n) is generated involving unisexual gynogenetic *Carassius gibelio*, sexual *C. auratus*, and sexual *C. cuvieri*. Then, we found that the gynogenesis ability have transferred from *C. gibelio* to some of the GR‐A3n females. This study subsequently established three GR‐A3n clones with distinct herpesvirus resistance and in which differential transcriptome profiles are characterized in two main hematopoietic organs. Most genes of the hemoglobin metabolism pathway is found to exhibit high expression levels, as in *C. cuvieri*, which led to efficient hemoglobin biosynthesis and blood oxygen homeostasis during infection, thereby resulting in strong herpesvirus resistance. Furthermore, this study determined the resistant and susceptible haplotypes derived from chromosome 12B of *C. cuvieri*, which should be responsible for resistance differences between GR‐A3n clones. Overall, this study establishes an approach for genetic improvement through polyploid genome design in animals.

## Graphical abstract

This study developed an efficient approach to generate genome‐reconstructed polyploids involving unisexual gynogenetic *Carassius gibelio*, sexual *C. auratus*, and sexual *C. cuvieri*. The selected superior clone inherited gynogenesis ability of *C. gibelio* and herpesvirus resistance of wild *C. cuvieri*, which provided the first case of engineering polyploid genomes with desirable traits by manipulating unisexual–sexual reproduction transition in animals.

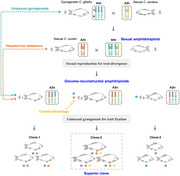

## 1 Introduction

Sexual reproduction predominates among eukaryotic organisms.^[^ ^1^, ^2^, ^3^ ^]^ During meiosis and fertilization, recombination and gamete fusion give rise to genetically variable progenies, which serve as the basis for selection in agriculture.^[^ ^4^, ^5^ ^]^ However, the beneficial traits obtained through sexual reproduction, especially heterosis, would segregate in subsequent generations.^[^ ^5^, ^6^, ^7^ ^]^ Harnessing unisexual reproduction has been hailed as the holy grail of agriculture, as it is expected to revolutionize biotechnology by allowing the perpetuation of any favored genotypes or genomes, especially in heterosis fixing.^[^ ^8^, ^9^, ^10^ ^]^ Interestingly, over 400 angiosperm species, as well as 100 vertebrate species or biotypes, have evolved modes of unisexual reproduction to produce clonal offspring.^[^ ^11^, ^12^, ^13^ ^]^ Natural unisexuality is often correlated with polyploidy.^[^ ^11^, ^14^, ^15^ ^]^ Notably, polyploidy is considered a key factor in crop and fish domestication because polyploids often have economic advantages in terms of biomass, robustness, and environmental adaptation.^[^ ^16^, ^17^, ^18^, ^19^, ^20^, ^21^ ^]^ Recently, the coupling of unisexuality and polyploidy has been demonstrated to enable the design of polyploid genomes for heterosis exploitation and fixation in crops.^[^ ^22^, ^23^ ^]^ However, utilizing unisexual reproduction to engineer polyploid genomes remains challenging in animals.

The amphitriploid *Carassius gibelio* (A3n ≈ 150, AAABBB) is an important aquaculture fish species in China with an annual production of approximately 3 million tons.^[^ ^24^ ^]^ A previous comparative genome analysis suggested that *C. gibelio* originated from the ancestral amphidiploid *C. auratus* (A2n = 100, AABB) via autotriploidy approximately 0.82–0.96 million years ago.^[^ ^25^ ^]^ *C. gibelio* can reproduce via gynogenesis, a typical unisexual reproduction mode in which unreduced eggs are triggered to initiate embryogenesis by the sperm from sympatric sexual species. To overcome the negative consequences of meiotic absence, *C. gibelio* have developed various strategies, such as paternal introgression and sporadic recombination, to prevent declines in genetic diversity.^[^ ^25^, ^26^ ^]^ Recently, this research group obtained a sexual amphitetraploid (A4n ≈ 200, AAAABBBB) male produced by occasional fusion between an egg (AAABBB) of *C. gibelio* clone A^+^, a dominant variety bred by our laboratory, and a sperm (AB) of *C. auratus*.^[^ ^27^, ^28^ ^]^ This sexual amphitetraploid male could produce large numbers of gynogenetic amphitriploid females (AAABBB) by backcrossing with the amphidiploid *C. auratus*.^[^ ^29^ ^]^ This finding demonstrated an effective strategy for generating genetic diversity and could serve as a breeding diagram for transferring desirable traits from sexual amphidiploids into gynogenetic amphitriploids based on the association between ploidy changes and reproduction transitions.^[^ ^11^ ^]^

Since 2012, *C. gibelio* has suffered an epizootic hemorrhagic disease caused by *C. auratus* herpesvirus (*Ca*HV), resulting in cumulative losses of billions of dollars.^[^ ^30^, ^31^ ^]^ In this study, polyploid genomes with improved *Ca*HV resistance were designed by coupling unisexuality and polyploidy. In addition, this work revealed the genetic mechanisms of transferring an elite resistance trait from an amphidiploid wild relative and then fixing into the genome‐reconstructed amphitriploids (GR‐A3n). More importantly, one GR‐A3n clone was developed as a new variety for aquaculture with enhanced resistance and growth advantage. Altogether, this research is the first case of engineering polyploid genomes with desirable traits by manipulating unisexual–sexual reproduction transitions in animals.

## 2 Results

### 2.1 Comparison of Three Parental Genomes and the Synthesis of Novel Polyploids

**Figure 1:**
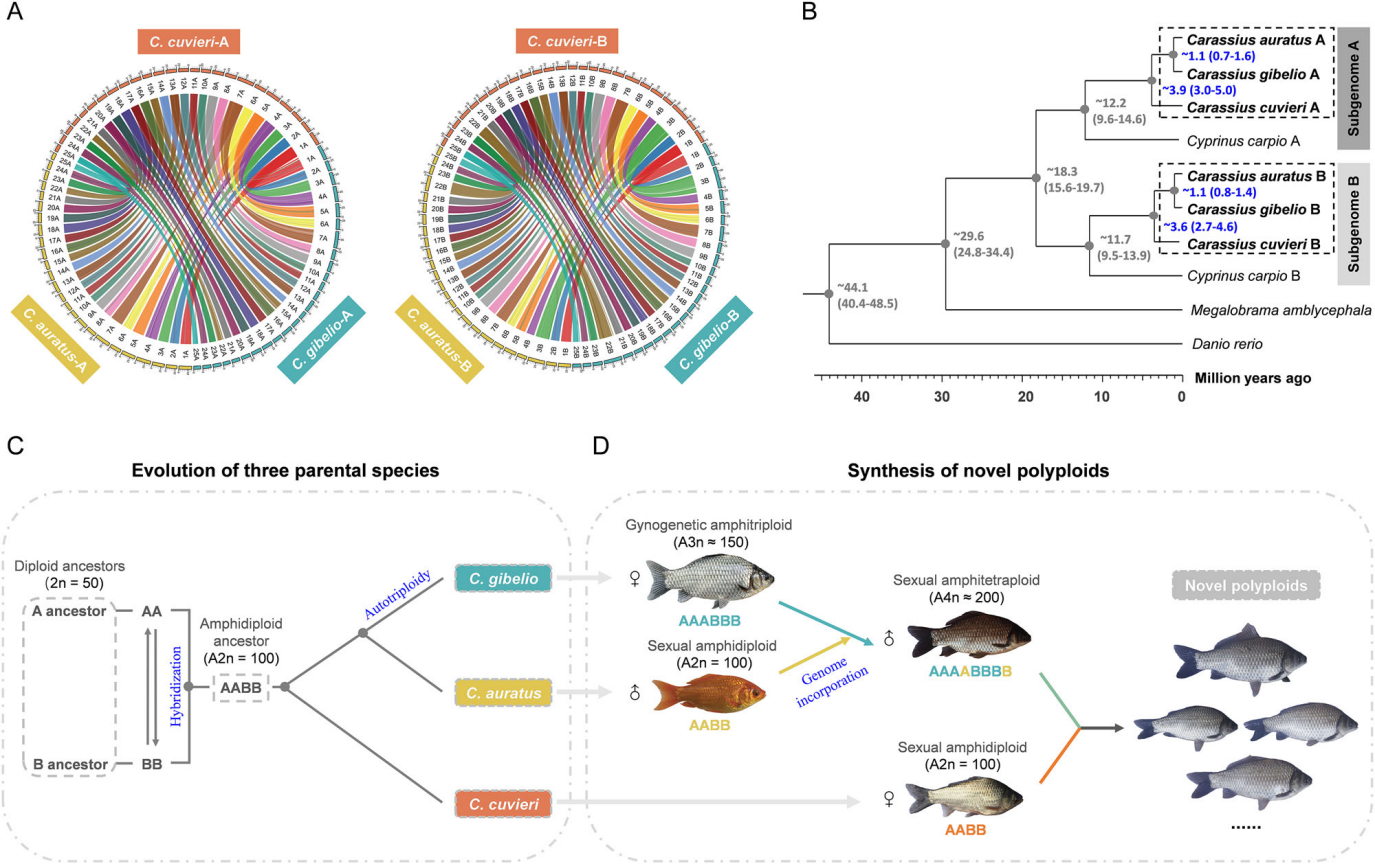
Phylogenetic analysis of three parental species and the synthesis of novel polyploids. A) Syntenic relationships of subgenomes A and B among *C. cuvieri*, *C. auratus*, and *C. gibelio*. The outermost circle shows the 25 chromosomes of *C. cuvieri* (orange, up), *C. auratus* (yellow, left), and *C. gibelio* (cyan, right) in subgenomes A (left) and B (right). The inner lines represent the syntenic relationships among the homologous chromosomes of *C. cuvieri*, *C. auratus*, and *C. gibelio*. B) Phylogenetic relationships and divergence times of the three subgenomes in the genus *Carassius*. The bootstrap values are all higher than 95. C) Genome evolution of *C. cuvieri*, *C. auratus*, and *C. gibelio*. The numbers represent the chromosome numbers. D) Schematic diagram of the generation of novel polyploids involving three parental species.

*Carassius cuvieri*, a sexual species with 100 chromosomes (Figure S1A, Supporting Information), has stronger *Ca*HV resistance than *C. auratus* and *C. gibelio*.^[^ ^32^ ^]^ To introduce *Ca*HV resistance, we selected wild *C. cuvieri* as the maternal parent to cross with the sexual amphitetraploid male to synthesize novel polyploids. The *C. cuvieri* genome was required for a better understanding of the evolutionary relationships among the three parental species, which was significant for the subsequent analysis of the novel polyploids produced in this study. Therefore, this study generated a high‐quality haplo‐genome assembly for *C. cuvieri* utilizing a combination of sequencing technologies, including next‐generation sequencing (NGS, by DNBSEQ‐T7, MGI), circular consensus sequencing (CCS, by PacBio Sequel II), and high‐resolution chromosome conformation capture (Hi‐C, by DNBSEQ‐T7, MGI) (Table S1, Supporting Information). The *C. cuvieri* genome was assembled with a size of 1.60 Gb and contig N50 of 9.29 Mb, and anchored to 50 chromosomes (Figure S1B, Supporting Information). Multiple genome alignments showed that the 50 chromosomes of *C. cuvieri* were divided into two subgenomes (A and B), each of which included 25 chromosomes. Then, synteny analysis of two subgenomes of *C. cuvieri*, *C. auratus*, and *C. gibelio* revealed a high degree of collinearity (**Figure** **1**A). To estimate their divergence time, we constructed a phylogenetic tree using 3922 single‐copy genes. As shown in Figure 1B, the A and B subgenomes of *C. cuvieri* diverged from the common ancestor of *C. auratus* and *C. gibelio* approximately 3.9 and 3.6 Mya, respectively, and the divergence of *C. auratus* and *C. gibelio* genomes was estimated at approximately1.1 Mya. These genome data suggest that ancient hybridization and the accompanying polyploidization between two distant diploid ancestors A and B (2n = 50, AA and BB genomes) may have led to the origin of the amphidiploid ancestor (A2n = 100); the amphidiploid clade of the genus *Carassius* may have diversified into *C. cuvieri* and *C. auratus*, while *C. gibelio* may descend from a recent autotriploidy event in *C. auratus* (Figure 1C). Therefore, *C*. *cuvieri* has a relatively distant relationship with *C. auratus* and *C. gibelio*, while *C. auratus* and *C. gibelio* are closely related (Figure 1C). Based on a recent finding on unisexual–sexual–unisexual reproduction transition driven by ploidy changes,^[^ ^28^, ^29^ ^]^ this study attempted to engineer polyploid genomes with desirable resistance traits. As shown in Figure 1D, the amphitetraploids were first generated by incorporating one AB haplotype genome of the sexual amphidiploid *C. auratus* into the genomes of the gynogenetic amphitriploid *C. gibelio*. Then, the sexual amphitetraploid male was mated with sexual amphidiploid *C. cuvieri*, and a group of novel polyploids possessing different genomes of the three species were thereby synthesized.

### 2.2 Reconstructed Genome Features of the Novel Polyploid

**Figure 2:**
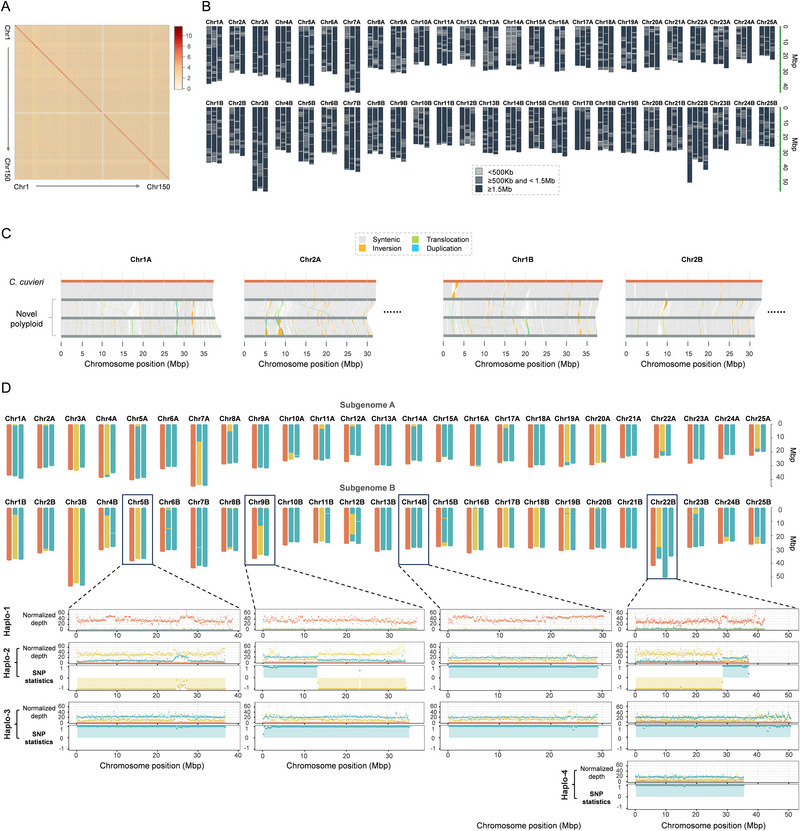
Haplotype‐resolved genome assembly and the analysis of a genome‐reconstructed amphitriploid (GR‐A3n). A) Hi‐C heatmap of the assembled haplotype‐resolved genome. The signal intensity of the Hi‐C heatmap is expressed as log_10_(Z+1). Z represents the calculated interaction intensity. B) Graphical representation of the contig distribution on the 150 assembled chromosomes. The colors represent different ranges of contig lengths. The haplotypes are drawn to scale. C) Collinearity analysis of chr1A, chr2A, chr1B, and chr2B between the three GR‐A3n haplotypes with the *C. cuvieri* haplotype. The haplotypes are depicted as horizontal lines and consist of the *C. cuvieri* haplotype (orange line, top) and three GR‐A3n haplotypes (gray lines, bottom). The collinearity analysis of all 50 homologous chromosome groups is shown in Figure S3 (Supporting Information). D) Graphical representation of a GR‐A3n karyotype. Orange, yellow, and cyan vertical ideograms represent haplotypes from *C. cuvieri*, *C. auratus*, and *C. gibelio*, respectively. The MGISeq short reads of *C. cuvieri*, *C. auratus*, and *C. gibelio* were mapped to the three haplotypes to calculate the mean normalized depth. According to the significant difference of the normalized depth between *C. cuvieri* reads with *C. auratus* and *C. gibelio* reads, haplotype‐1 derived from *C. cuvieri* can be distinguished from haplotype‐2 and haplotype‐3. Genotyping for the other two haplotypes was conducted based on the differential homozygous SNPs between *C. gibelio* and *C. auratus*. The non‐recombinant haplotype exhibited continuous and consistent SNPs compared to *C. gibelio* or *C. auratus* (1 or −1) along the entire chromosome, while discontinuous and reverse SNPs (1 and −1) existed in the recombinant haplotype.

To reveal the genome composition of the novel polyploids, we randomly selected one individual to assemble the haplotype‐resolved genome, using an integrated strategy of NGS (DNBSEQ‐T7, MGI), CCS (Pacbio Revio), and Hi‐C (DNBSEQ‐T7, MGI). We generated 197 Gb of CCS data and assembled them using HiFiasm, resulting in 4.98 Gb contigs with an N50 size of 2.95 Mb after purging (Tables S2,S3, Supporting Information). Assisted by the Hi‐C data, a total of 23555 contigs were assigned to 150 chromosomes (**Figure** **2**A,B), which was consistent with the chromosome number observed during metaphase (Figure S2, Supporting Information), and 7489 dissociative scaffolds were found (Table S4, Supporting Information). According to a collinearity analysis of the assembled *C. cuvieri* genome, the assembled 150 chromosomes were divided into 50 homologous chromosome groups and two AB subgenomes (Figure 2B,C; Figure S3, Supporting Information). The overwhelming majority of homologous groups had three haplotypes, except for Chr16A and Chr22B, which had two and four haplotypes, respectively (Figure 2B; Figure S3, Supporting Information). The results clearly show that the novel polyploid is an amphitriploid.

To analyze genome features of the novel amphitriploid, we conducted haplotype genotyping via a method based on that used in *Solanum tuberosum* L., *Coffea arabica*, and *Caenorhabditis nematodes* (Figure S4, Supporting Information).^[^ ^33^, ^34^, ^35^ ^]^ First, we conducted NGS (DNBSEQ‐T7, MGI) of the maternal *C. cuvieri* **♀** and the parents of the paternal amphitetraploid♂ (*C. gibelio*♀ and *C. auratus*♂) (Figure 1D) to obtain the MGISeq short reads (Table S5, Supporting Information). Second, we mapped the reads to the haplotype‐resolved genome, and the mean normalized depth in 10 Mb windows was calculated for each haplotype. As shown in Figure 2D, the mean normalized depth of *C. cuvieri* reads in one haplotype approached approximately 37.4, such as in Chr5B, whereas the mean normalized depths of *C. gibelio* and *C. auratus* reads in that haplotype were almost 0, indicating that haplotype‐1 of Chr5B was derived from *C. cuvieri* (Chr5B, Haplo‐1). The normalized depths of *C. cuvieri* reads were almost 0 in the other two haplotypes, whereas the normalized depths of *C. gibelio* and *C. auratus* reads in those two haplotypes ranged from 8.8 to 35.9, implying that the other two haplotypes were difficult to distinguish based only on depth statistics. Finally, we further distinguished the genotypes of the other two haplotypes, according to the statistics of homozygous single nucleotide polymorphisms (SNPs) that differed between *C. gibelio* and *C. auratus*. When a haplotype had the same SNPs as *C. gibelio* or *C. auratus*, it was counted as 1 or −1, respectively. The mean value in 20 Mb windows was calculated in the other two haplotypes. Therefore, the mean values of almost all windows approached −1 in haplotype‐2 of Chr5B, whereas they approached 1 along the entirety of haplotype‐3, indicating that haplotype‐2 (Chr5B, Haplo‐2) and haplotype‐3 (Chr5B, Haplo‐3) were derived from *C. auratus* and *C. gibelio*, respectively. However, the mean value was 1 at the one terminal portion of haplotype‐2 of Chr9B, whereas it was −1 in the other terminal portion, implying that a recombination event between *C. auratus* and *C. gibelio* had taken place to generate a recombinant chromosome (Chr9B, Haplo‐2). A graphical representation of the novel amphitriploid karyotype based on these analyses is shown in Figure 2D. There are four categories of representative homologous chromosome groups. The first category includes Chr3A, Chr3B, and Chr5B, which inherited one haplotype each from *C. cuvieri* (orange), *C. auratus* (yellow), and *C. gibelio* (cyan). The chromosomes in the second category, including Chr6A and Chr14B, inherited one haplotype from *C. cuvieri* and two haplotypes from *C. gibelio*. The third category contains the chromosomes that inherited one haplotype from *C. cuvieri*, one haplotype from *C. gibelio*, and a recombinant haplotype, including chromosomes Chr1B and Chr9B. The fourth category includes two aneuploid chromosomes (Chr16A and Chr22B). Overall, the novel amphitriploid inherits one genome set (AB) from the maternal *C. cuvieri* and two genome sets (AABB) from the paternal amphitetraploid. In the latter compositions, one or two recombination events have taken place in some homologous chromosomes between *C. auratus* and *C. gibelio*. Therefore, the novel amphitriploid is termed as the genome‐reconstructed amphitriploid (GR‐A3n).

### 2.3 Regain of Unisexual Gynogenesis Ability

**Figure 3:**
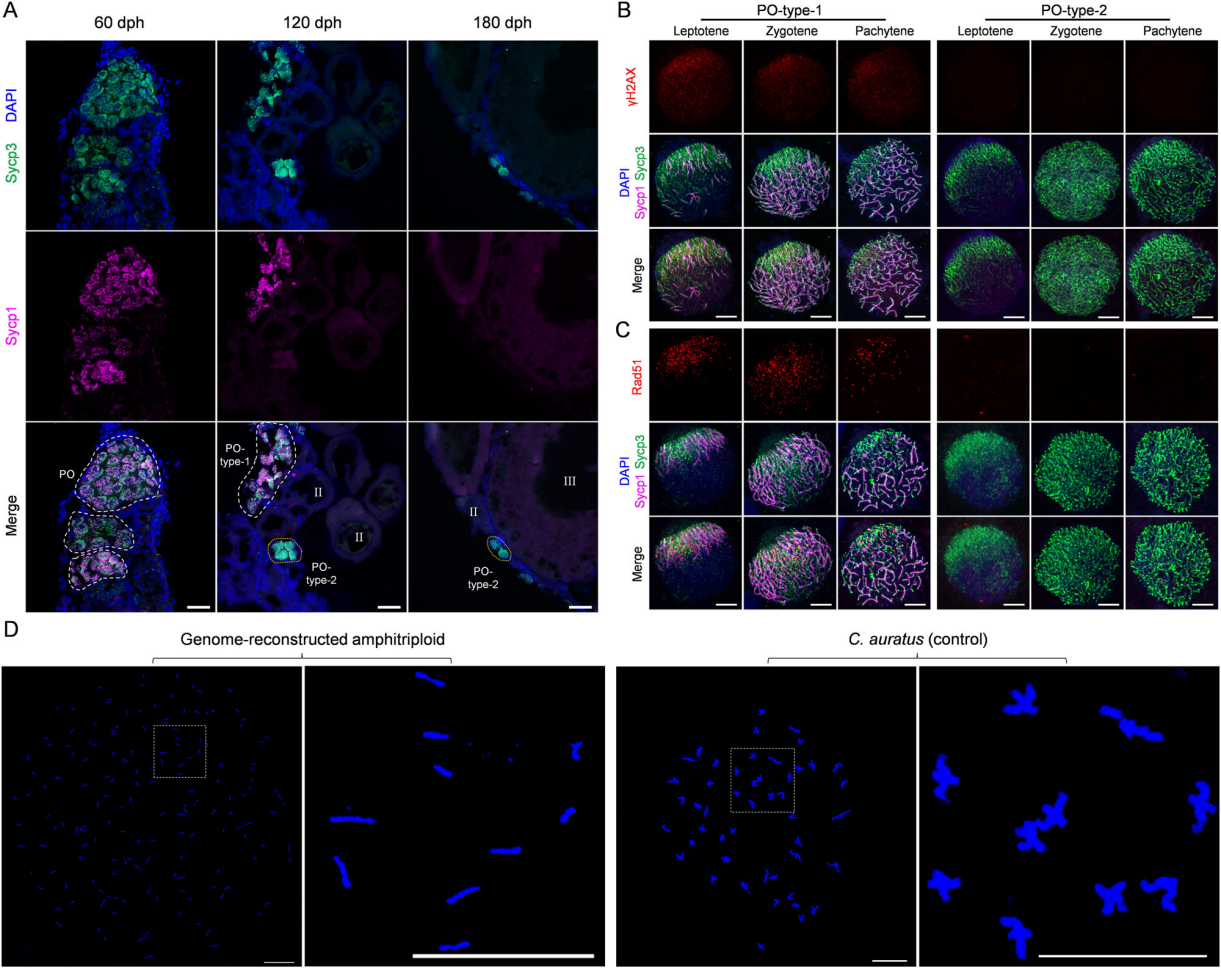
Cytological evidence on regaining the gynogenesis ability of GR‐A3n. A) Immunohistochemical analysis of GR‐A3n ovaries based on anti‐Sycp1 (magenta) and anti‐Sycp3 (green) antibodies. Nuclei were stained with DAPI (blue). The white and gold dotted circles indicate that the oocyte clusters were filled with type‐1 and type‐2 primary oocytes, respectively. Females at 60 dph (n = 10), 120 dph (n = 10), and 180 dph (n = 10) were sampled for immunohistochemical analysis. dph, days post hatching; PO, primary oocyte; II, growth stage oocyte; III, vitellogenic oocyte. Scale bar, 25 µm. B, C) Chromosomal spreads of two types of primary oocytes were co‐immunostained by anti‐Sycp1 (magenta), anti‐Sycp3 (green), and anti‐γH2AX (red) (B) or anti‐Rad51 (red) (C) antibodies. Nuclei were stained with DAPI (blue). Scale bar, 5 µm. Females at 120 dph (n = 10) were sampled for oocyte chromosomal spreads. D) DAPI‐stained chromosome spread of germinal vesicles at metaphase I. A total of 30 eggs each from genome‐reconstructed amphitriploids (n = 3) and *C. auratus* (n = 3) were collected to isolate GVs. Scale bar, 20 µm.

Considering the meiotic difficulty in the pairing and equal segregation of three homologous chromosomes in the GR‐A3n, we investigated the cytological process of meiosis I during oogenesis. Immunohistochemical analysis was performed to examine synaptonemal complex (SC) formation in the GR‐A3n ovaries using the antibodies for the SC transverse element (Sycp1) and lateral element (Sycp3). At 60 days post hatching (dph), large numbers of oogonia had entered prophase I, and abundant primary oocytes (POs) were observed (**Figure** **3**A). Sycp1 and Sycp3 signals were detected simultaneously, indicating that synapsis had occurred in almost all the primary oocytes (Figure 3A, 60 dph). Interestingly, two distinct kinds of POs were distributed in separate clusters at 120 dph (Figure 3A, 120 dph). Nuclear micro‐spreads exhibited more detailed cytological processes based on the co‐staining of antibodies against Sycp1, Sycp3, phosphorylated histone variant γH2AX, or recombinase Rad51, among which the latter two antibodies are markers of DSB (DNA double‐strand breaks) formation and recombinational repair, respectively (Figure 3B,C). In the type‐1 POs, both bivalent SCs with co‐localized Sycp1 and Sycp3 signals and univalent SCs with only Sycp3 signals were detected. Abundant γH2AX signals were still mostly distributed in the clustered regions of univalent SCs, and considerable numbers of Rad51 foci remained on the axes of univalent SCs at pachytene, indicating that improper SC assembly and incomplete DSB repair occurred in the primary oocytes (Figure 3B,C). In type‐2 POs, the Sycp3 signal was detected, while the γH2AX, Sycp1, and Rad51 signals were detected at negligible levels. This indicated that DSB formation, homologous synapsis, and recombination were largely inhibited (Figure 3B,C), which was similar to findings in *C. gibelio* oocytes.^[^ ^25^, ^29^ ^]^ Approximately 53.8% of the GR‐A3n females developed normal ovaries (Figure 3A, 180 dph) and could spawn mature eggs (Table S6, Supporting Information). Subsequently, homologous chromosome pairing at metaphase I was observed in the germinal vesicles (GVs) of eggs. Approximately 150 unpaired univalent chromosomes were detected in GVs of GR‐A3n, whereas 50 paired bivalents with standard crossovers existed in the control GVs of amphidiploid *C. auratus* (Figure 3D). Consistent with the findings reported in *C. gibelio* ^25,28^, the results revealed that the type‐2 POs of GR‐A3n adopted the same ameiotic pathway as *C. gibelio* oocytes via the alternative regulation of DSB formation.

To further evaluate fertility, the GR‐A3n eggs were fertilized with sperm from *C. cuvieri*, and the nuclear behavior during fertilization was traced. The sperm nuclei of *C. cuvieri* remained in a condensed state and failed to fuse with the female pronucleus, whereas the fertilized egg accomplished the first cleavage normally (Figure S5, Supporting Information). The average fertilization rate and larval survival rate were over 80% in the crossed groups of GR‐A3n♀ × *C. cuvieri*♂ (Table S7, Supporting Information). In addition, approximately 46.2% of GR‐A3n females exhibited dysplastic ovaries with large numbers of arrested or apoptotic oocytes (Figure S6A, Supporting Information), and only one kind of PO was detected in the ovaries (Figure S6B, Supporting Information, PO‐type‐1), which demonstrated that the type‐1 POs could not develop into mature eggs. These results indicated that the unisexual gynogenesis ability was transferred from *C. gibelio* into some GR‐A3n individuals.

### 2.4 Establishment of Three GR‐A3n Clones

**Figure 4:**
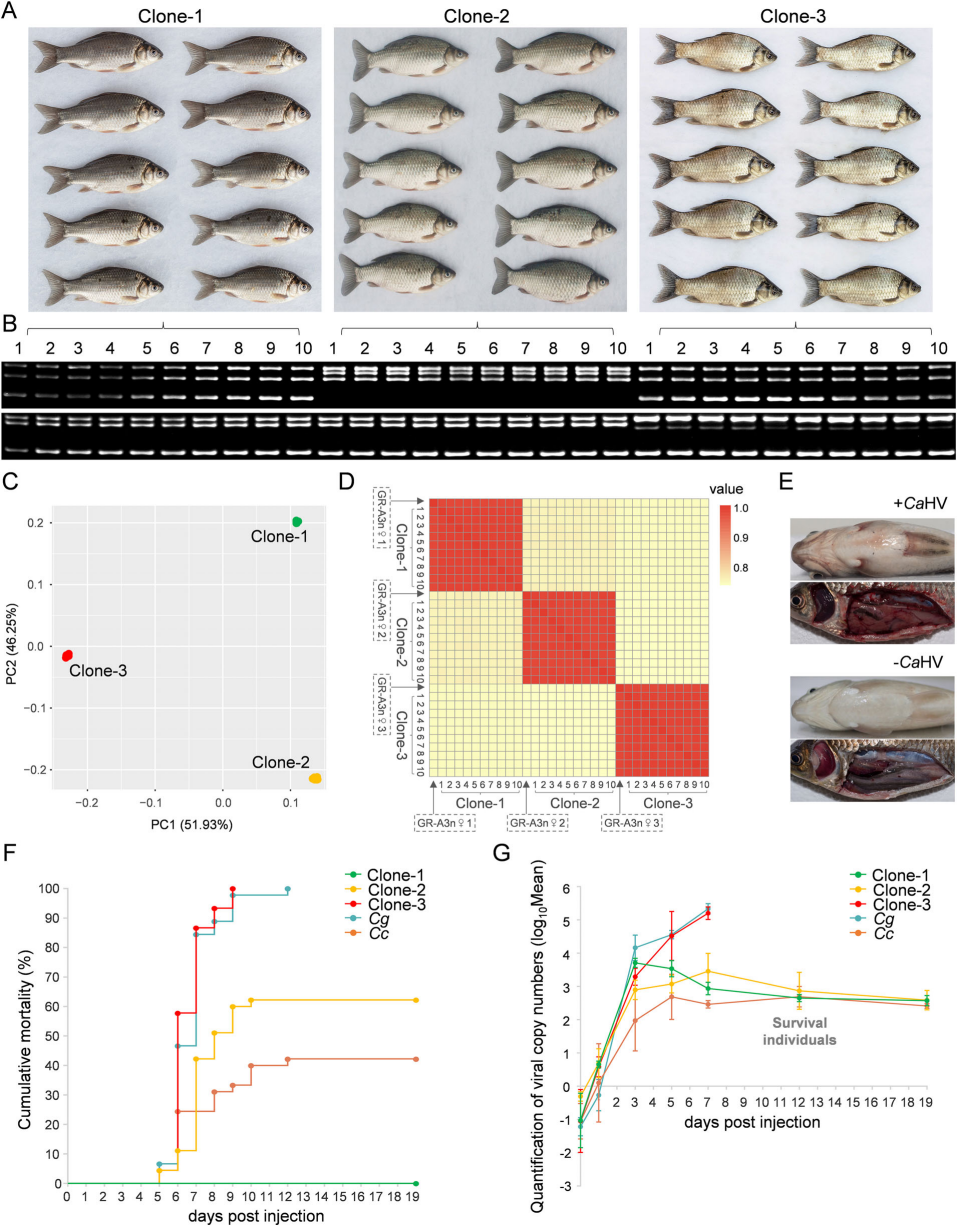
Three GR‐A3n clones with distinct herpesvirus resistance. A) Morphological comparison of three GR‐A3n clones reproduced via recovered gynogenesis. B) Microsatellite genotypes of the three GR‐A3n clones amplified by two primers of YJ0029 (up) and YJ0018 (down). C) Principal component analysis illustrating genetic differentiation among the three GR‐A3n clones. D) Heatmap from identity by‐state analysis illustrating genetic differentiation between all lineages. GR‐A3n♀1–3, three maternal GR‐A3ns; Clone‐1, gynogenetic offspring of GR‐A3n♀1; Clone‐2, gynogenetic offspring of GR‐A3n♀2; Clone‐3, gynogenetic offspring of GR‐A3n♀3. E) Symptoms of diseased fish (+ *Ca*HV): hyperemia at the base of the fins and on the abdomen, bleeding gills, and internal organ hemorrhaging. F) Survival analysis after herpesvirus infection. The values are the mean ± standard deviation (SD) of three replicate tanks. *Cg*, *C. gibelio*; *Cc*, *C. cuvieri*. G) Analysis of viral loads during infection. Data are shown as the mean ± SD (n = 6), representing *Ca*HV copy number per ng DNA of the head‐kidney.

As some GR‐A3n females recovered the ability to perform unisexual gynogenesis, various clones were established to screen for *Ca*HV‐resistant varieties. During this process, three females with growth advantages, weighing 380, 398, and 398 g respectively, were selected to cross with a *C. cuvieri* male to generate three gynogenetic clones. The intra‐clonal homogeneity and inter‐clonal heterogeneity of the three clones were obvious not only in body morphological characteristics (**Figure** **4**A) but also in microsatellite genotypes (Figure 4B). Furthermore, 10 individuals from each clone were randomly selected to assess genetic diversity by NGS (DNBSEQ‐T7, MGI) (Table S8, Supporting Information). Principal component analysis (PCA) confirmed that the average genetic variation within individuals of each clone was low; however, the three clones were clearly distinguishable based on the SNP data (Figure 4C). The average genetic identity between two individuals of the same clone ranged up to 98.2%, and that between two individuals of different clones was only approximately 73.0% (Figure 4D).

### 2.5 Different Herpesvirus Resistance of the three GR‐A3n Clones

This study assessed the *Ca*HV resistance among the three clones based on herpesvirus challenge experiments, with *C. gibelio* clone A^+^ and *C. cuvieri* selected as controls. The severely infected fish died due to gill and internal organ hemorrhaging (Figure 4E). Consistent with previous research,^[^ ^32^ ^]^ *C. gibelio* was highly susceptible to *Ca*HV, while *C. cuvieri* exhibited strong herpesvirus resistance, with approximately 58.0% of infected *C. cuvieri* individuals surviving (Figure 4F). Similar to *C. gibelio*, all infected clone‐3 individuals died within 9 days post infection (dpi). Approximately 38.9% of the clone‐2 individuals survived the *Ca*HV infection. Significantly, no clone‐1 individuals died, which demonstrated complete resistance against *Ca*HV (Figure 4F). These results indicate that the three GR‐A3n clones possess distinct levels of *Ca*HV resistance. Then, the viral loads were evaluated in head‐kidney tissues using real‐time PCR. As shown in Figure 4G, *Ca*HV was barely detectable in all uninfected groups (0 dpi), and viral loads increased significantly at 3 dpi, indicating that massive replication of *Ca*HV occurred in all groups within 3 dpi. Similar to *C. gibelio*, the average viral load of susceptible clone‐3 continued to exponentially increase to 10^5.20^ particles/ng DNA at 7 dpi. Severe pathologic damage and massive lesions were detected in the head‐kidney tissue, characterized by tissue loose and lymphocyte surge at 3 dpi and by tissue fibrosis and lymphocyte necrosis at 5 dpi (Figure S7, Supporting Information). However, the average viral load of clone‐1 decreased remarkably after 3 dpi, displaying strong resistance against *Ca*HV. The inflection points of *C. cuvieri* and clone‐2 appeared at 5 and 7 dpi, respectively (Figure 4G). Collectively, these analyses demonstrate that *Ca*HV resistance can be transferred from sexual *C. cuvieri* into partial GR‐A3n clones.

### 2.6 Gene Expression Patterns of the three GR‐A3n Clones

**Figure 5:**
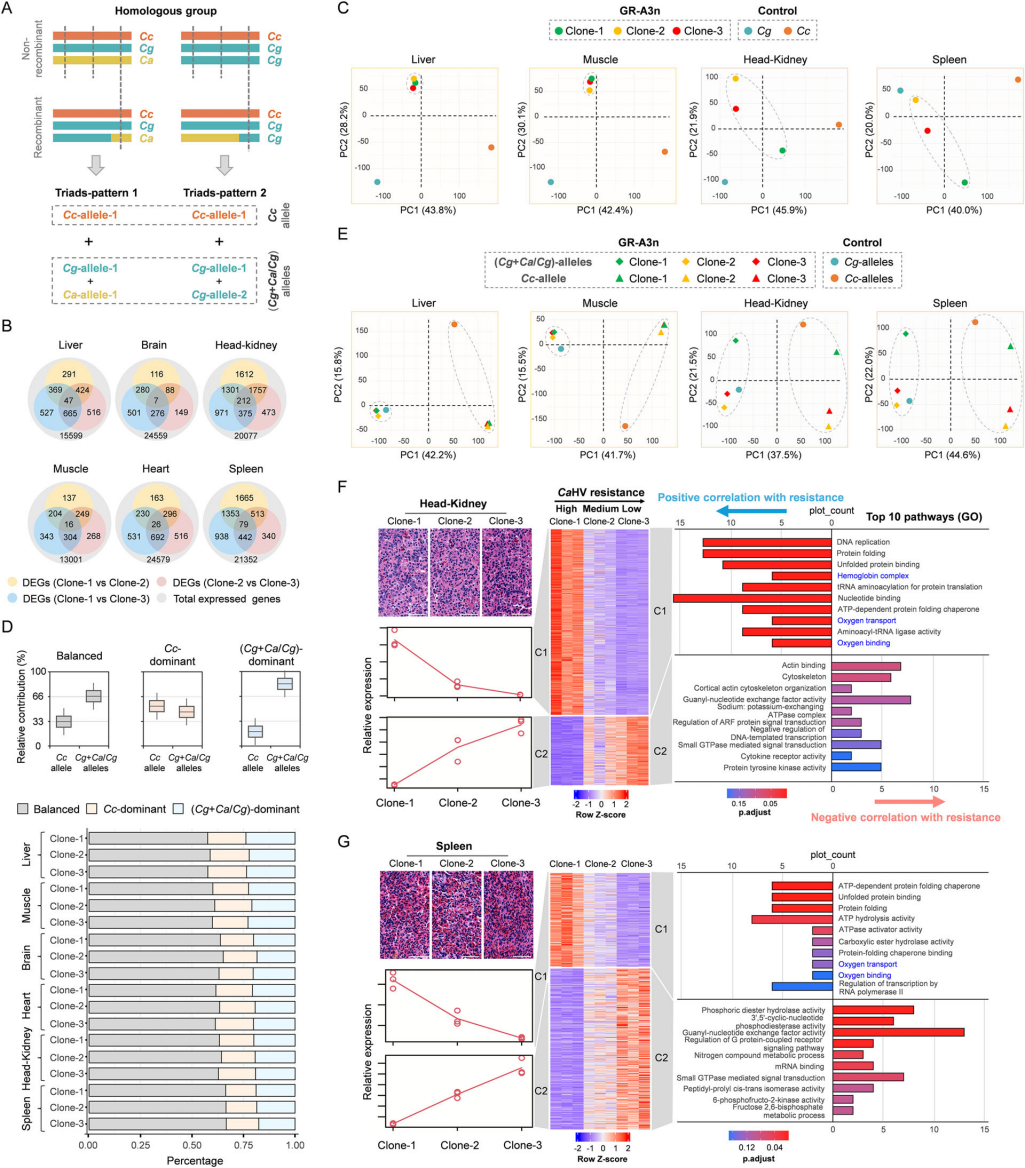
Transcriptional landscape of the three GR‐A3n clones. A) Graphical representation of two patterns of allele triads in the GR‐A3n. Orange, yellow, and cyan vertical ideograms represent haplotypes derived from *C. cuvieri*, *C. auratus*, and *C. gibelio*, respectively. The first pattern includes three alleles each from *C. cuvieri*, *C. gibelio* and *C. auratus*, and the second pattern includes one allele from *C. cuvieri* and two alleles from *C. gibelio. Cc*, *C. cuvieri*; *Cg*, *C. gibelio*; *Ca*, *C. auratus*; *Cc*‐derived allele, one allele derived from *C. cuvieri*; (*Cg*+*Ca*/*Cg*)‐derived alleles, two alleles derived from *C. gibelio* and *C. auratus* or two alleles derived from *C. gibelio*. B) Venn diagrams of DEG numbers between different clones in six adult tissues. DEG, differentially expressed gene. C) Principal component analysis illustrating an overview of gene expression patterns in four tissues from the three GR‐A3n clones; the gene expression patterns of *C. cuvieri* and *C. gibelio* are shown as the controls. D) Proportion of three allele expression categories across the six tissues in the three GR‐A3n clones. E) Principal component analysis illustrating an overview of allele expression patterns in four tissues from the three GR‐A3n clones; gene expression patterns of *C. cuvieri* and *C. gibelio* are shown as controls. F, G) Analysis of genome‐wide transcriptomic alterations in the head‐kidney (F) and spleen (G) among the three GR‐A3n clones. Left, relative expression patterns with corresponding tissue sections above. Scale bar, 50 µm. Middle, heatmap of expression patterns. Right, top 10 enriched pathways (GO) for each pattern. n =  3 individuals per clone. C1 and C2 are the two expression clusters exhibiting positive and negative correlations with herpesvirus resistance, respectively.

Understanding how multi‐genome reconstruction affects gene expression is critical to determine the genetic mechanism of distinct resistance levels among the three GR‐A3n clones. To uncover the global gene expression, we performed transcriptome analyses of six tissues, and the transcriptomes of *C. cuvieri* and *C. gibelio* were analyzed as controls (Table S9, Supporting Information). Based on the chromosome compositions of homologous groups in GR‐A3n (Figure 2D), triads of alleles could be divided into two patterns. The first pattern included one allele each from *C. cuvieri*, *C. gibelio*, and *C. auratus*, and the second pattern included one allele from *C. cuvieri* and two alleles from *C. gibelio* (**Figure** **5**A). Given the complexity of the GR‐A3n genome, we first evaluated the reference genome using RNA‐seq reads from the liver of *C. cuvieri*, *C. gibelio*, and *C. auratus* (see Experimental Section for details). When a mixed genome dataset of *C. gibelio* and *C. cuvieri* was used as the reference genome, 84.3% of the *C. cuvieri* RNA‐seq reads could be preferentially mapped to the *C. cuvieri* genome, and 81.1% and 80.0% of the *C. gibelio* and *C. auratus* reads could be preferentially mapped to the *C. gibelio* genome, respectively, owing to the close relationship between *C. gibelio* and *C. auratus* (Figure 1B). Therefore, we obtained the expression level of the *C. cuvieri*‐derived allele (*Cc*‐derived allele) and the total expression levels of one *C. gibelio*‐derived allele and one *C. auratus*‐derived allele or two *C. gibelio*‐derived alleles ((*Cg*+*Ca*/*Cg*)‐derived alleles) (Figure 5A). The total expression level of GR‐A3n was calculated based on the sum of the expression levels of the *Cc*‐derived allele and the (*Cg*+*Ca*/*Cg*)‐derived alleles. We identified 15599, 13001, 24559, 24579, 20077, and 21352 genes expressed from the liver, muscle, brain, heart, head‐kidney, and spleen, respectively (Figure 5B). Among these expressed genes, we identified 2839, 1521, 1417, 2454, 6701, and 5330 differentially expressed genes (DEGs), which were significantly differentially expressed between at least two clones (Figure 5B). DEGs accounted for approximately 5.8% (brain) to 33.4% (head‐kidney) of all expressed genes. To obtain an overview of gene expression patterns in the three clones, PCA was conducted using the total expression levels of three alleles (Figure 5C; Figure S8, Supporting Information) and bi‐plots were generated for the first (PC1) and second (PC2) principal components, which explained approximately 70.0% of the total variance. In four tissues, namely, the liver, muscle, brain, and heart, the three GR‐A3n clones clustered closely with each other, implying that they shared similar transcriptional patterns. However, the three clones were separated from each other in the two main hematopoietic organs of fish (the head‐kidney and spleen), which implied that the gene expression involved in hematopoietic function might differ among the three clones (Figure 5C).

To compare the expression differences and differing contributions between the *Cc*‐derived allele and (*Cg*+*Ca*/*Cg*)‐derived alleles in three clones, we defined three allele expression categories based on the relative expression level of the *Cc*‐derived allele and (*Cg*+*Ca*/*Cg*)‐derived alleles: one balanced category with a relative expression level close to 1:2, which is consistent with the dose, and two allele‐dominant categories, classified on the basis of a higher (≥ 1:1, *Cc*‐dominant) or lower (≤ 1:4, *Cg*+*Ca*/*Cg*‐dominant) relative expression level (Figure 5D). Most expressed genes within the six tissues were assigned to the balanced category, with balanced triads ranging from 58.0% (liver) to 66.5% (spleen) (Table S10, Supporting Information). An average of 37.6% expressed triads exhibited unbalanced expression between the *Cc*‐derived allele and (*Cg*+*Ca*/*Cg*)‐derived alleles. Triads with (*Cg*+*Ca*/*Cg*)‐derived allele dominance (20.5%; range among tissues, 18.4%–23.2%) were more frequent than triads with *Cc*‐derived allele dominance (17.1%; range among tissues, 16.4%–18.8%). Across all six tissues, there was no significant difference in the proportion of the three categories among the three clones. To better understand the expression landscape of different derived alleles, we performed PCA using allele expression data (Figure 5E; Figure S9, Supporting Information). PC1 separated the *Cc*‐derived allele and (*Cg*+*Ca*/*Cg*)‐derived alleles of the three clones, but clustered the *Cc*‐derived allele and (*Cg*+*Ca*/*Cg*)‐derived alleles of the three clones with alleles of *C. cuvieri* and alleles of *C. gibelio*, respectively. Consistent with the overview of total expression patterns (Figure 5C), the *Cc*‐derived allele or (*Cg*+*Ca*/*Cg*)‐derived alleles of the three clones clustered closely with each other in four non‐hematopoietic organs but were clearly separated in the head‐kidney and spleen (Figure 5E; Figure S9, Supporting Information), reflecting the differential allele expression patterns among the three clones in hematopoietic organs.

### 2.7 Differential Gene Expression of Hematopoietic Organs Among GR‐A3n Clones

Next, this study further analyzed the genome‐wide transcriptomic alterations in the two hematopoietic organs among the three GR‐A3n clones. A total of 6701 and 5330 DEGs (Figure 5B) that were significantly different in the head‐kidney and spleen between at least two clones, respectively, were screened to perform hierarchical clustering. The DEGs in the two clusters indicating a positive (C1) and negative (C2) correlation with *Ca*HV resistance were selected for Gene Ontology (GO) pathway enriched analysis (Table S11, Supporting Information). Among the top 10 pathways in C1, “oxygen transport” and “oxygen binding” were shared between the head‐kidney and spleen, and “hemoglobin complex” was also enriched in the head‐kidney (Figure 5F,G). These data imply that oxygen metabolism may be associated with *Ca*HV resistance in the GR‐A3n.

### 2.8 *Ca*HV Resistance is Potentially Related to Hemoglobin Biosynthesis

**Figure 6:**
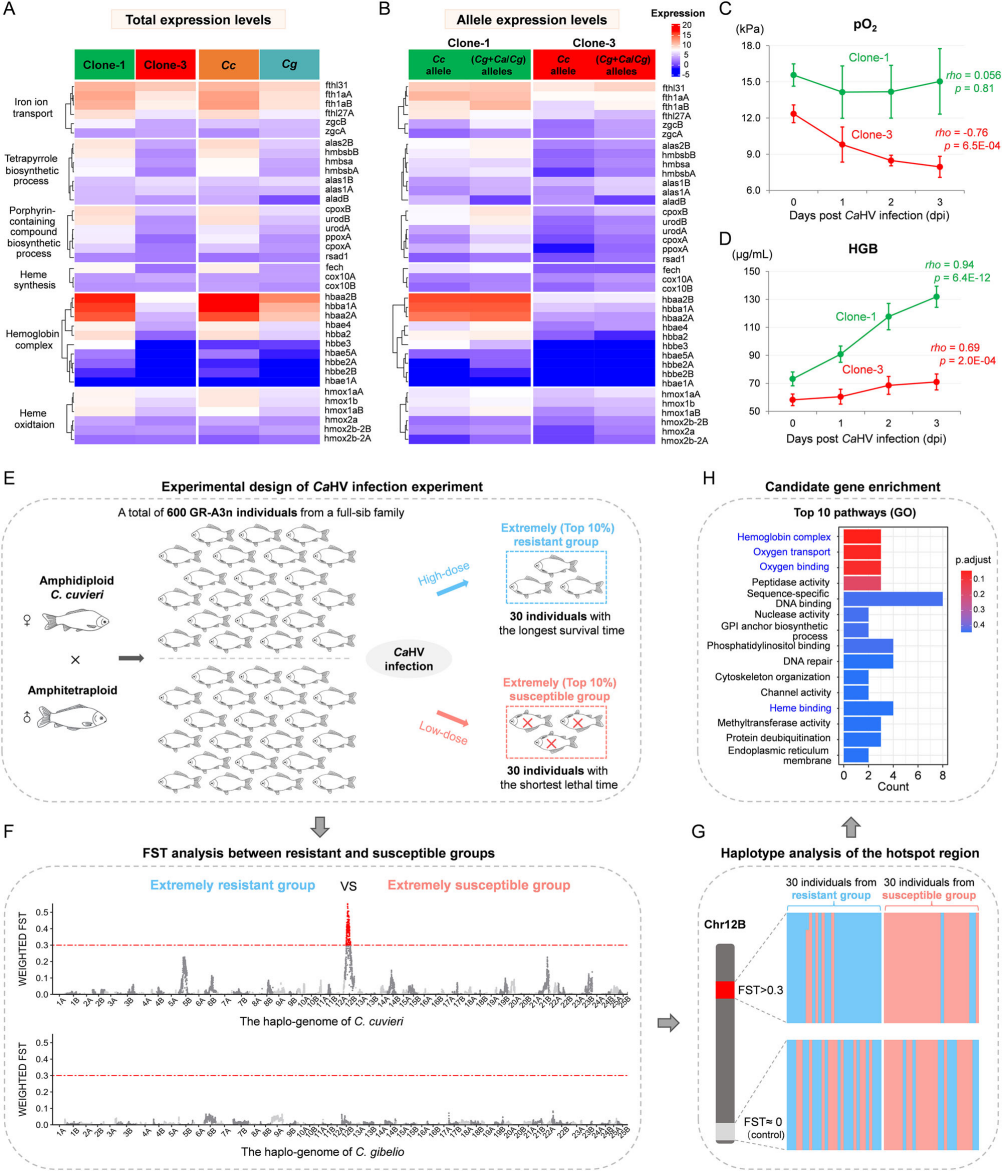
Hemoglobin biosynthesis and haplotype differences between distinct resistant GR‐A3ns. A) Heatmap of 38 expressed genes related to six main pathways of hemoglobin metabolism in the head‐kidney of resistant clone‐1, susceptible clone‐3, *C. cuvieri*, and *C. gibelio*. B) Heatmap of the *Cc*‐derived and (*Cg*+*Ca*/*Cg*)‐derived alleles of 38 expressed genes related to six main pathways of hemoglobin metabolism in the head‐kidney of clone‐1 and clone‐3. *Cc*, *C. cuvieri*; *Cg*, *C. gibelio*; *Ca*, *C. auratus*. C, D) Dynamic changes in the arterial partial pressure of oxygen (pO_2_) (**C**) and hemoglobin concentration (HGB) (D) in clone‐1 and clone‐3 during *Ca*HV infection. The values are the mean ± standard deviation for each time point (n = 4–6). The correlation between days of infection and pO_2_ or HGB were marked by Spearman's Rho with the corresponding p‐value. E) Schematic diagram of design of *Ca*HV infection experiment, in which extremely resistant and susceptible GR‐A3n individuals were selected from a full‐sib family. F) Fixation index (Fst) analysis of 30 GR‐A3n individuals each from the extremely resistant and susceptible groups to identify genomic regions associated with the resistance trait. The hotspot region (Fst ≥ 0.3) was concentrated on chromosome 12B of *C. cuvieri*. G) Haplotype analysis of the hotspot region. The hotspot region (Fst ≥ 0.3) showed two distinct *C. cuvieri*‐haplotypes between the two groups, while the haplotypes in the control region (FST ≈ 0) were random. H) Gene ontology enrichment of candidate genes in the hotspot region.

To reveal the detailed transcriptional differences in hemoglobin biosynthesis and catabolism, 38 expressed genes (FPKM ≥ 1) in six main pathways, including iron ion transport, tetrapyrrole biosynthetic process, and porphyrin‐containing compound, were selected to compare expression levels between the most resistant (clone‐1) and most susceptible (clone‐3). In the head‐kidney and spleen (**Figure** **6**A; Figure S10A, Supporting Information), the overwhelming majority of these genes in resistant clone‐1 exhibited significantly higher expression levels than those in susceptible clone‐3. A similar gene expression difference was observed between the resistant *C. cuvieri* and susceptible *C. gibelio* (Figure 6A; Figure S10A, Supporting Information), which implied that the transcriptional activity of hemoglobin metabolism might be positively associated with *Ca*HV resistance. Interestingly, both the *Cc*‐derived allele and (*Cg*+*Ca*/*Cg*)‐alleles of clone‐1 exhibited higher expression levels, similar to *C. cuvieri*, demonstrating the dominance of the *C. cuvieri* expression level (Figure 6B; Figure S10B, Supporting Information). By contrast, the alleles of most genes in clone‐3 had a lower expression level, similar to that in *C. gibelio* (Figure 6B; Figure S10B, Supporting Information). The results suggest that muti‐genome reconstruction should have a significant effect on the allele expression patterns in the hemoglobin metabolism pathway, potentially leading to differences in hemoglobin biosynthesis during infection between clone‐1 and clone‐3.

To further reveal the relationship between *Ca*HV resistance and hemoglobin biosynthesis, we investigated the dynamic changes in the arterial partial pressure of oxygen (pO_2_) and the hemoglobin concentration (HGB) in clone‐1 and clone‐3 during infection. The pO_2_ values remained stable (≈15.0 kPa) in clone‐1, whereas the pO_2_ values decreased from 12.4 ± 0.7 to 8.0 ± 0.9 kPa in clone‐3 within 3 dpi (Figure 6C). The HGB in the blood increased rapidly from 73.2 ± 5.0 to 131.9 ± 7.5 µg/mL in clone‐1, but rose only slightly from 58.2 ± 4.1 to 71.0 ± 5.7 µg/mL in clone‐3 within 3 dpi (Figure 6D). Similar variation trends in pO_2_ and HGB were observed in the resistant *C. cuvieri* and susceptible *C. gibelio* (Figure S11, Supporting Information). These results suggest that clone‐1 maintains blood oxygen homeostasis during *Ca*HV infection via efficient hemoglobin biosynthesis, which may result in high resistance against *Ca*HV.

### 2.9 Identification of Resistant and Susceptible Haplotypes Derived from *C. cuvieri*

Distinct *Ca*HV resistance between clones should be caused by genetic differences between their maternal GR‐A3ns. To uncover the genetic basis of differential resistance between clone‐1 and clone‐3, this study selected extremely resistant and susceptible GR‐A3n individuals to implement a population genetic analysis. As shown in Figure 6E, a total of 600 GR‐A3n individuals from a full‐sib family were randomly divided into two groups, which were subjected to high (1.2 × 10^8^ virus particles/g) and low (1.2 × 10^6^ virus particles/g) dose *Ca*HV infection experiments. A total of 30 individuals (top 10%) with the longest survival time from the high‐dose group, and 30 individuals (top 10%) with the shortest lethal time from the low‐dose group (Figure S12, Supporting Information) were selected for NGS (DNBSEQ‐T7, MGI) (Table S12, Supporting Information). We calculated the fixation index (*Fst*) between the extremely resistant and susceptible groups to identify genomic regions associated with the *Ca*HV resistance trait; the mixed genome dataset of *C. cuvieri* and *C. gibelio* was used as a reference genome. Interestingly, one genomic region with a high degree of population differentiation (*Fst* ≥ 0.3) was concentrated on chromosome 12B of *C. cuvieri* (Figure 6F). This hotspot region had two distinct *C. cuvieri* haplotypes between the extremely resistant and susceptible groups, except for a small number of individuals (Figure 6G), and the resistant and susceptible haplotypes were consistent with the *C. cuvieri* haplotypes of clone‐1 and clone‐3, respectively (Figure S13, Supporting Information). The ∼11.0 Mb sequence harbored 292 genes (Table S13, Supporting Information), which were significantly enriched in hemoglobin biosynthesis, oxygen transport, oxygen binding, and heme binding pathway (Figure 6H; Table S14, Supporting Information). A hemoglobin gene cluster that consisted of three hemoglobin subunits (*hbae4, hbbe2B*, and*hbbe3*) was identified. Therefore, we speculate that the haplotype of the hemoglobin gene cluster in the resistant group and clone‐1 is probably the favored type, which may contribute to the efficient synthesis of hemoglobin and oxygen homeostasis maintenance during *Ca*HV infection. The data suggest that the haplotype difference of Chr12B derived from *C. cuvieri* leads to the distinct resistances between GR‐A3n individuals, and the distinct levels of resistance can be inherited and fixed into the corresponding unisexual clones.

## 3 Discussion

**Figure 7:**
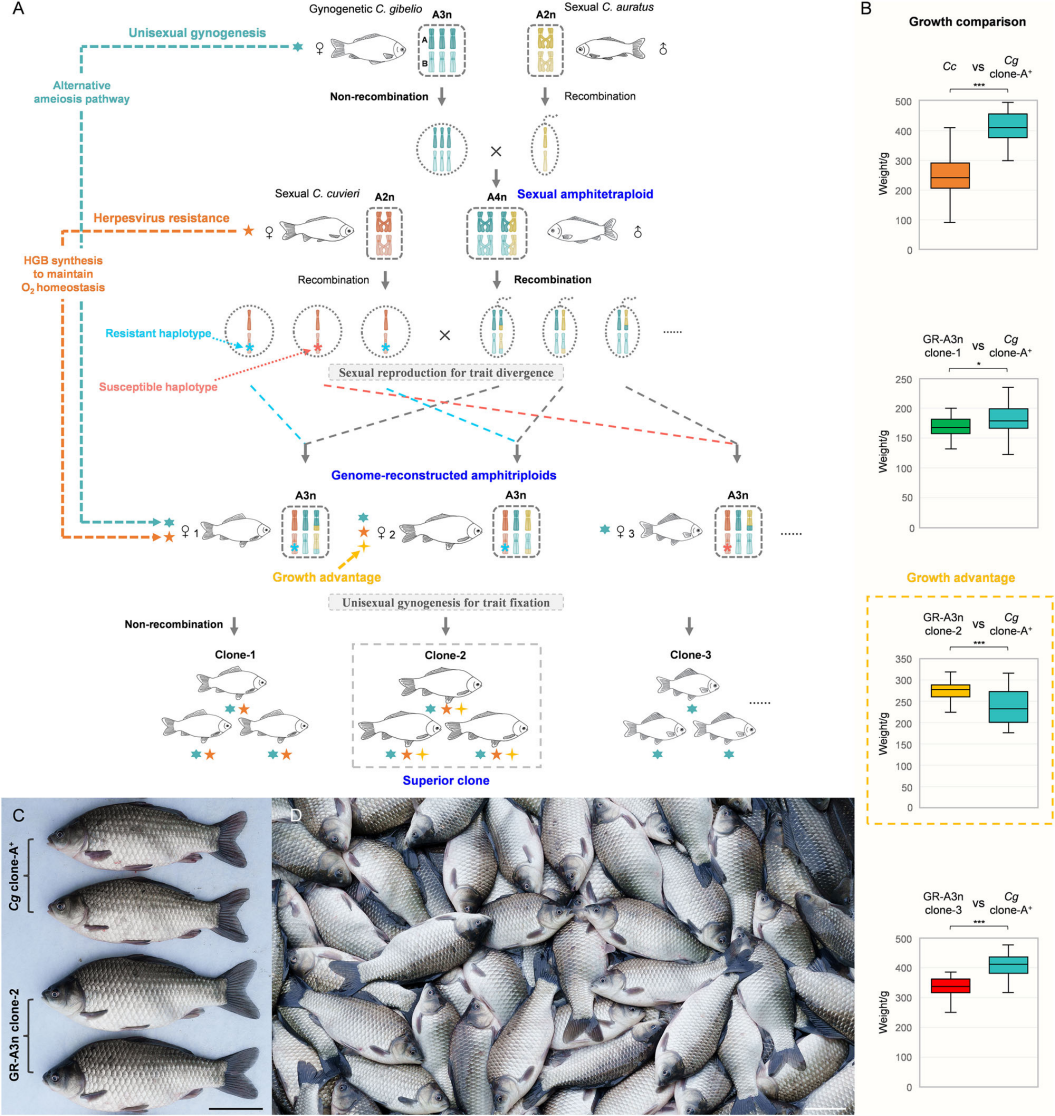
Manipulating unisexual–sexual reproduction transition to engineer genome‐reconstructed polyploids with elite traits. A) Schematic diagram of ploidy changes and reproduction transitions driving resistance trait divergence and fixation in the genome‐reconstructed amphitriploids. Six, five, and four‐pointed stars represent gynogenesis ability, herpesvirus resistance, and growth advantage, respectively. Blue and red asterisks (^*^) represent resistant and susceptible haplotypes, respectively. A2n, amphidiploid; A3n, amphitriploid; A4n, amphitetraploid; GR‐A3n, genome‐reconstructed amphitriploid. B) Growth comparison between the three GR‐A3n clones and *C. cuvieri* with *C. gibelio* clone‐A^+^ (control), respectively. *C. gibelio* clone‐A^+^ is a dominant variety that has been cultured throughout China. The average weights of *C. cuvieri*, GR‐A3n clone‐1, and GR‐A3n clone‐3 reached 59.3%, 93.7%, and 83.1% of the average weight of *C. gibelio* clone‐A^+^, respectively, while the average weight of GR‐A3n clone‐2 was 1.13 times that of *C. gibelio* clone‐A^+^. Asterisks (^*^) and (^***^) indicate significant differences (0.01 < P <0.05 and P <0.001, respectively). *Cg*, *C. gibelio*; *Cc*, *C. cuvieri*. C) Morphological comparison between *C. gibelio* clone‐A^+^ (top) and GR‐A3n clone‐2 (bottom). Scale bar, 10 cm. D) Image of the GR‐A3n clone‐2 being widely cultured as a source of high‐quality protein in China. Scale bar, 10 cm.

Both unisexuality and polyploidy are significant in agriculture, exhibiting revolutionary biotechnology potential.^[^ ^16^, ^17^, ^22^, ^23^ ^]^ Here, we provide the first successful case of engineering polyploid genomes with desirable traits by manipulating unisexual–sexual reproduction transition in animals. As shown in **Figure** **7**A, this study generated a sexual amphitetraploid (AAAABBBB) by incorporating one haplotype genome (AB) from amphidiploid *C. auratus* into amphitriploid *C. gibelio* (AAABBB). Then, the sexual amphitetraploid was mated with amphidiploid *C. cuvieri* (AABB), in which the fusion of recombinant sperm and egg led to the formation of genome‐reconstructed amphitriploids (AAABBB). Therefore, through the multi‐genome reconstruction and reproduction transitions driven by ploidy changes, both the gynogenesis ability of *C. gibelio* and the herpesvirus resistance of a wild sexual *C. cuvieri* were introduced into the genome‐reconstructed amphitriploid. Moreover, we provide comprehensive evidence that the gynogenesis transfer is achieved by the alternative ameiosis pathway during oogenesis (Figure 3) and that the herpesvirus resistance is highly correlated with hemoglobin biosynthesis and oxygen homeostasis maintenance during infection (Figure 6A–D). Furthermore, the findings reveal that the distinct resistances between GR‐A3n individuals are likely caused by the integration of two different haplotypes derived from *C. cuvieri* (Figure 6E–H). The favored resistant haplotype is subsequently fixed into the resistant clone through the recovered gynogenesis (Figure 7A), and the growth advantage is also fixed into the GR‐A3n clone‐2 (Figure 7A,B), which has been developed as a new variety for aquaculture owing to its enhanced resistance and increased yield (Figure 7C,D).

Sexual reproduction is typically characterized by the generation of genetically diverse individuals through homologous recombination and ploidy reduction via meiosis and through ploidy restoration by the fusion of biparental pronuclei during fertilization.^[^ ^13^ ^]^ In agricultural breeding, sexual reproduction is necessary to generate elite genotypes and the basis for heterosis; however, homologous chromosome recombination and segregation can also break up favorable combinations of genotypes accumulated through selection, leading to the segregation of desirable traits and reduced heterosis in subsequent generations.^[^ ^5^, ^6^, ^7^ ^]^ Utilization of heterosis thus depends on the production of F1 hybrids by crosses, which significantly increases the cost of seed production.^[^ ^6^, ^36^ ^]^ Unisexual reproduction is achieved via a wide variety of modified processes, ranging from alternative ameiosis pathways to different ploidy maintenance mechanisms, as in parthenogenesis or gynogenesis.^[^ ^11^, ^37^ ^]^ Unisexual reproduction represents an efficient means to fix and amplify elite genotypes resulting from sexual reproduction.^[^ ^1^, ^12^, ^36^, ^38^ ^]^ Therefore, harnessing unisexual reproduction has potential applications for heterosis fixation and polyploid genome design in agriculture.^[^ ^22^, ^23^, ^24^, ^36^, ^39^ ^]^

Two approaches, namely, introgression through conventional backcrossing and *de novo* engineering, have been proposed to produce clonal progeny in crop breeding.^[^ ^9^ ^]^ To date, small number of cases, such as turf grass hybrid varieties (*Poa pratensis* L.) and hybrid lines of *Hieracium pilosella*,^[^ ^10^, ^40^ ^]^ have demonstrated that the introgression of apomixis (a form of asexual reproduction through seed) can be employed in plant breeding to fix complex traits across generations. More progress has been made in *de‐novo* engineering apomixis in several major crops by fine‐tuning a small number of key regulators of meiosis and embryogenesis, such as the “mitosis instead of meiosis” (*MiMe*) system and haploid induction, which can produce clonal gametes and eliminate the contribution of the paternal genome, respectively.^[^ ^7^, ^38^, ^41^ ^]^ The *MiMe* system was previously established in hybrid rice and was recently applied to establish polyploid genome design in hybrid tomatoes; however, the fertility of the engineered polyploid tomatoes was remarkably reduced and the clonal seed rate remained low, hindering agriculture practices.^[^ ^22^, ^23^, ^42^ ^]^ Manipulating unisexual reproduction to engineer polyploid genomes remains a challenge in animals. In the present study, unisexual–sexual reproduction transitions were driven by ploidy changes, and the sexual amphitetraploid acted as a bridge to transfer the gynogenesis ability from *C. gibelio* into the GR‐A3n females. A better understanding is needed to explain how GR‐A3n females regain their gynogenesis ability, necessitating the search for the mechanism of DSB formation regulation. This study uncovered two types of primary oocytes (DSB formation or inhibition) in fertile GR‐A3n females (Figure 3A–C), and showed that these two types of primary oocytes were distributed in separate clusters of ovaries (Figure 3A), implying that they originated from two different types of oogonia. Oogonia within the same ovary should possess the same genomic composition. Thus, we speculated that epigenetic regulation prior to meiosis within oogonia might be responsible for the alternative regulation of DSB formation and fertility establishment in GR‐A3n females. Single‐cell epigenomics may be able to reveal the epigenetic differences between the two types of oogonia in future studies.^[^ ^43^ ^]^ If the epigenetic regulation mechanism and controlling genes are discovered, they may find wide applications in the production of novel polyploid animals.

Another intriguing finding in this study was the observation that herpesvirus resistance was highly associated with oxygen homeostasis in fish (Figure 6). Oxygen is an indispensable factor required for many biological processes in aerobic organisms;^[^ ^44^ ^]^ thus, severe and prolonged hypoxia caused by the virus attacking respiratory organs is fatal. On the other hand, numerous studies have confirmed the prominent role of oxygen metabolism and hypoxia in innate immunity.^[^ ^45^ ^]^ Oxygen can act as a direct substrate for some enzymes, such as the EGLN prolyl hydroxylases, which are the factors inhibiting HIF asparaginyl hydroxylase.^[^ ^46^ ^]^ A recent study in zebrafish confirmed that oxygen could enhance antiviral innate immunity through the maintenance of EGLN1‐catalyzed proline hydroxylation of interferon regulatory factor 3 (IRF3), revealing a direct link between oxygen and antiviral responses and highlighting the key mechanism by which oxygen regulates innate immunity.^[^ ^46^ ^]^ By comparing the transcriptome data of the head‐kidney between 0 and 1 dpi, we also found that the activation of the immune‐related pathway in resistant clone‐1, which maintained oxygen homeostasis during infection, was significantly faster than in the hypoxic clone‐3 (Figure S14, Supporting Information). In addition, the maintenance of oxygen homeostasis during infection was found to be achieved through the efficient synthesis of hemoglobin in the resistant clone (Figure 6A–D). Moreover, a hotspot region, containing a hemoglobin gene cluster, hypoxia‐inducible factor 1 (*hif2a*), and cysteamine (2‐aminoethanethiol) dioxygenase (*ado*), was found to localize on chromosome 12B derived from *C. cuvieri* (Table S13, Supporting Information). These genes have been reported to be associated with hemoglobin synthesis and oxygen homeostasis.^[^ ^47^, ^48^, ^49^ ^]^ Future studies on their functions will help elucidate the detailed antiviral mechanisms.

In conclusion, this study demonstrates that unisexual–sexual reproduction transition enables polyploid genome design in animals, potentially paving the way for completely novel breeding schemes.

## 4 Experimental Section

### Experimental Fish

All experimental fish, including amphidiploid *C. cuvieri* (*Cc*) and *C. auratus* (*Ca*), amphitriploid *C. gibelio* clone A^+^ (*Cg*), amphitetraploid male (A4n♂), and genome‐reconstructed amphitriploids (GR‐A3n) were maintained and sampled from the National Aquatic Biological Resource Center. Animal experiment was approved by the Animal Care and Use Committee of the Institute of Hydrobiology (IHB), Chinese Academy of Sciences (CAS).

### Genome Assembly and Annotation of C. cuvieri

Genomic DNA was extracted from the blood cells of a female adult individual from *C*. *cuvieri*. The genomic long reads and short reads were obtained using PacBio Sequel II and DNBSEQ‐T7 (MGI) platform, respectively. Hi‐C library was constructed and sequenced according to the published protocol.^[^ ^50^ ^]^ RNA was extracted from six adult tissues (brain, heart, head‐kidney, liver, muscle, and spleen) and used to construct libraries, which were sequenced on DNBSEQ‐T7 platform. Genome assembly, chromosome anchoring, and genome annotation were performed as described previously.^[^ ^25^ ^]^ The assembled genome of *C*. *cuvieri* was aligned to *C*. *auratus* or *C*. *gibelio* genome by minimap 2 (v2.24‐r1122).^[^ ^51^ ^]^

### Phylogenetic Analysis of Three Species in the Genus Carassius

To understand the evolutionary relationships of three *Carassius* species, genomes of six Cyprinidae fishes, including *C. cuvieri* (PRJNA1189180), *C. gibelio* (PRJNA546443), *C. auratus* (PRJNA546444), *Cyprinus carpio* (ASM1834038v1), *Megalobrama amblycephala* (ASM1881202v1), and *Danio rerio* (GRCz11), were used to construct phylogenetic tree. The 10 peptide sequence sets from two genomes (*M. amblycephala* and *D. rerio*) and eight subgenomes (subgenome A of *C. cuvieri*, *C. gibelio*, *C. auratus*, and *C. carpio*, subgenome B of *C. cuvieri*, *C. gibelio*, *C. auratus*, and *C. carpio*) were subject to all‐to‐all blast to identify the potential homologous sequences with an *E*‐value <10^−5^. Blast results were clustered to identify single copy orthologs from the genomes of *M. amblycephala*, *D. rerio* and the subgenomes A and B of *C. cuvieri, C. gibelio*, *C. auratus*, and *C. carpio* by OrthoMCL (v2.0.9),^[^ ^52^ ^]^ and these single copy orthologs were used to construct phylogenetic tree. Multiple sequence alignment was performed by MAFFT (v7.487),^[^ ^53^ ^]^ and phylogenetic tree was constructed according to 4DTv extracted by Gblocks (v0.91b).^[^ ^54^ ^]^ Divergence times between genomes and subgenomes were estimated by mcmctree (v4.9).^[^ ^55^ ^]^ Collinearity analysis was performed using coding sequences from *C. cuvieri*, *C. gibelio*, and *C. auratus* by JCVI (v1.1.22) with default parameters.^[^ ^56^ ^]^

### Haplotype‐Resolved Genome Assembly of the Genome‐Reconstructed Amphitriploid

Genomic DNA was extracted from the blood cells of a GR‐A3n individual. The genomic long reads were obtained using PacBio Revio platform. PacBio long reads were *de novo* assembled to contigs by HiFiasm (v0.19.6) software,^[^ ^57^ ^]^ and the haplotype‐resolved processed unitig graph was selected. Assisted by the Hi‐C reads, contigs were assigned to chromosomes. The assembled haplotype‐resolved genome was aligned to the assembled *C. cuvieri* genome to determine the homologous chromosome groups using mummer (v4.0).^[^ ^58^ ^]^

### Histological Section, Preparation of Oocyte Chromosomal Spreads and Immunostaining

Immunohistochemical analysis, preparation of oocyte chromosomal spreads, and immunostaining were performed as described previously.^[^ ^39^, ^59^ ^]^ All antibodies and dilutions used are listed in Table S15 (Supporting Information). The images were acquired under Leica STELLARIS 8 FALCON confocal microscopy (Analytical & Testing Center, IHB, CAS).

### Bivalent Spread of Germinal Vesicle and Observation of Nuclear Events During Fertilization

Isolation of germinal vesicle, bivalent spread, and observation of nuclear events during fertilization were performed as described previously.^[^ ^29^ ^]^ The images were acquired under Leica SP8 DLS confocal microscopy (Analytical & Testing Center, IHB, CAS).

### Microsatellite Analysis

Microsatellite analysis was conducted according to the standard procedures as described previously.^[^ ^29^ ^]^ Two pairs of microsatellite primers (YJ0029 and YJ0018) were used in this study.

### Genetic Diversity Analysis Among Three GR‐A3n Clones

Genomic DNA was extracted from the caudal fins of 30 individuals from the three clones and their maternal GR‐A3n females. The NGS was performed on the DNBSEQ‐T7 platform (Table S8, Supporting Information). The MGISeq short reads of 33 individuals were aligned to the mixed genome dataset of *C. gibelio* and *C. cuvieri*. SNP calling and identity by‐state analysis were conducted as described previously.^[^ ^29^ ^]^ Heatmap was drawn using the R package, and PCA analysis was performed using prcomp function in R (v4.3.2).

### CaHV Infection and Quantification of Viral Copy Numbers

*Ca*HV was isolated from the tissues of naturally diseased *C. gibelio* with acute gill hemorrhages. Healthy individuals with a weight of approximately 30 grams were selected for *Ca*HV infection. Individuals of three GR‐A3n clones, *C. cuvieri*, and *C. gibelio* clone‐A^+^ were randomly divided into four tanks with 20–30 fishes per tank. *Ca*HV infection were performed with 300 µL viral suspension (7.2 × 10^8^ virus particles) per fish by intraperitoneal injection. Individuals in three tanks were used to calculate the average mortality rate. The fishes in the remaining tank were used for sampling and other analysis, such as arterial blood gas analysis and hemoglobin concentration measurement, as well as viral quantification and transcriptome analysis of head kidney and spleen. Quantification of viral copy numbers were conducted according to the standard procedures as described previously.^[^ ^60^ ^]^

### Transcriptome Sequencing and Gene Expression Analysis

RNA was extracted from samples of *C. cuvieri*, *C. auratus*, *C. gibelio*, and the three GR‐A3n clones, including six adult tissues (brain, heart, head‐kidney, liver, muscle, and spleen). Three biological replicates were analyzed per sample. In total, 90 RNA‐seq libraries were constructed and sequenced on DNBSEQ‐T7 platform (Table S9, Supporting Information). Given the genome complexity of the GR‐A3n, we first evaluated the reference genome. RNA‐seq reads from liver of *C. cuvieri*, *C. gibelio*, and *C. auratus* were mapped to the two reference genomes, including a mixed genome dataset of *C. cuvieri*, *C. gibelio*, and *C. auratus* (*C. cuvieri* + *C. gibelio* + *C. auratus*), and a mixed genome dataset of *C. cuvieri* and *C. gibelio* (*C. cuvieri* + *C. gibelio*). According to the mapping rate (Table S16, Supporting Information), less than 50.0% of the *C. auratus* and *C. gibelio* reads could be correctly mapped to their corresponding genomes, thus the mixed genome dataset of *C. cuvieri*, *C. gibelio*, and *C. auratus* was insufficient as the reference genome. When the mixed genome dataset of *C. gibelio* and *C. cuvieri* was used as the reference genome, 84.3% of *C. cuvieri* reads could be preferentially mapped to the *C. cuvieri* genome, and 80.0% and 81.1% of *C. gibelio* and *C. auratus* reads could be preferentially mapped to the *C. gibelio* genome (Table S16, Supporting Information). Therefore, the mixed genome dataset of *C. gibelio* and *C. cuvieri* was selected as the reference genome for transcriptome analysis. The expression level of the *C. cuvieri* allele (*Cc*‐derived allele) and the total expression levels of the *C. gibelio* allele and *C. auratus* allele ((*Cg*+*Ca*/*Cg*)‐derived alleles) were obtained, respectively. The total expression level of the GR‐A3n was calculated based on the sum of the expression levels of *Cc*‐derived allele and (*Cg*+*Ca*/*Cg*)‐derived alleles. In addition, the *C. cuvieri* and *C. gibelio* genomes were used as the reference genomes of *C. cuvieri* and *C. gibelio* samples, respectively. Subsequently, expression quantification was performed by Rsem (v1.3.1).^[^ ^61^ ^]^ PCA was conducted using the prcomp function in R (v4.3.2). Hierarchical clustering was performed as described method.^[^ ^62^ ^]^

### Arterial Blood Gas Analysis and Hemoglobin Concentration Measurement

About 300 to 500 µL of mixed arterial blood was collected from tail artery, and the blood was analyzed by the blood gas analyzer (Radiometer ABL9 Blood gas electrolyte and metabolite analyzer, Denmark) within 30 s. Hemoglobin concentration was detected through enzyme‐linked immunoassay by fish hemoglobin ELISA kit (MM‐2370O1, China).

### FST Analysis Between the Extremely Resistant and Susceptible GR‐A3n Individuals

Genomic DNA was extracted from the caudal fins of 60 individuals from the extremely resistant and susceptible groups. The NGS was performed on the DNBSEQ‐T7 platform (Table S12, Supporting Information). FST value between resistant and susceptible groups was calculated by VCFtools (v0.1.13).^[^ ^63^ ^]^ Enrichment analysis of genes in the hotspot region was performed by clusterProfiler (v4.10.1).^[^ ^64^ ^]^

### Growth Comparison

The growth differences among the three GR‐A3n clones, *C. cuvieri*, and *C. gibelio* clone‐A^+^ were evaluated by weight comparison. Fingerlings of approximately the same size (0.15–0.30 g) were employed to test and compare their growth rates. A total of 1000 individuals from each of the three GR‐A3n clones and *C. cuvieri* were co‐cultured with 1000 individuals of *C. gibelio* clone‐A^+^ in four independent ponds (666 m^2^). After 6 months, 30–50 individuals each from the three GR‐A3n clones, *C. cuvieri*, and co‐cultured *C. gibelio* were randomly weighted (Table S17, Supporting Information). In the first pond, the average weights of *C. cuvieri* and co‐cultured *C. gibelio* clone‐A^+^ were 243.2 and 410.4 g, respectively. In the second pond, the average weights of GR‐A3n clone‐1 and co‐cultured *C. gibelio* clone‐A^+^ were 169.0 and 180.3 g, respectively. In the third pond, the average weights of GR‐A3n clone‐2 and co‐cultured *C. gibelio* clone‐A^+^ were 270.1 and 238.0 g, respectively. In the fourth pond, the average weights of GR‐A3n clone‐3 and co‐cultured *C. gibelio* clone‐A^+^ were 332.6 and 400.2 g, respectively.

## Conflict of Interest

The authors declare no conflict of interest.

## Author Contributions

M.L., Q.‐C.Z., and Z.‐Y.Z. contributed equally to this work. J.‐F.G., L.Z., M.L. designed the study. M.L., Q.‐C.Z., Z.‐Y.Z., Y.W., X.‐Y.L., Z.‐W.W., Z.L., and X.‐J.Z. prepared the samples and carried out the experiments. M.L., L.Z., and J.‐F.G. analyzed and discussed the results. M.L., L.Z., and J.‐F.G. wrote the paper.

## Supporting information



Supporting Information

## Data Availability

The data that support the findings of this study are available in the supplementary material of this article.;

## References

[1] R. Butlin , Nat. Rev. Genet. 2002, 3, 311. 11967555 10.1038/nrg749

[2] D. Speijer , J. Lukeš , M. Eliáš , Proc. Natl Acad. Sci. U. S. A 2015, 112, 8827. 26195746 10.1073/pnas.1501725112 PMC4517231

[3] V. Yadav , S. Sun , J. Heitmana , Proc. Natl Acad. Sci. U. S. A 2023, 120, 2219120120. 10.1073/pnas.2219120120 PMC10013875 36867686

[4] A F. Agrawal , Nature 2001, 411, 692. 11395771 10.1038/35079590

[5] S P. Otto , Genetics 2003, 164, 1099. 12871918 10.1093/genetics/164.3.1099 PMC1462613

[6] F. Hochholdinger , J A. Baldauf , Curr. Biol. 2018, 28, R1089. 30253145 10.1016/j.cub.2018.06.041

[7] A. Mahlandt , D K. Singh , R. Mercier , Theor. Appl. Genet. 2023, 136, 131. 37199785 10.1007/s00122-023-04357-3 PMC10195744

[8] J P V. Calzada , C F. Crane , D M. Stelly , Science 1996, 274, 1322.

[9] C. Spillane , M D. Curtis , U. Grossniklaus , Nat. Biotechnol. 2004, 22, 687. 15175691 10.1038/nbt976

[10] C. Sailer , B. Schmid , U. Grossniklaus , Curr. Biol. 2016, 26, 331. 26832437 10.1016/j.cub.2015.12.045

[11] M. Lu , L. Zhou , J F. Gui , Sci. China Life Sci. 2024, 67, 449. 38198030 10.1007/s11427-023-2486-2

[12] R. Barbuti , S. Mautner , G. Carnevale , P. Milazzo , A. Rama , C. Sturmbauer , BMC Evol. Biol. 2012, 12, 49. 22489797 10.1186/1471-2148-12-49 PMC3353185

[13] J. Lafond , C. Leung , B. Angers , Nat. Commun. 2024, 15, 1. 39223116 10.1038/s41467-024-52041-x PMC11368912

[14] D. Hojsgaard , E. Hörandl , Front. Plant Sci. 2019, 10, 358. 31001296 10.3389/fpls.2019.00358 PMC6454013

[15] J C. Avise , Proc. Natl Acad. Sci. U. S. A 2015, 112, 8867. 26195735 10.1073/pnas.1501820112 PMC4517198

[16] A. Leitch , I. Leitch , Science 2008, 320, 481. 18436776 10.1126/science.1153585

[17] Y. Van de Peer , E. Mizrachi , K. Marchal , Nat. Rev. Genet. 2017, 18, 411. 28502977 10.1038/nrg.2017.26

[18] R. H. Ramírez‐González , P. Borrill , D. Lang , S. A. Harrington , J. Brinton , L. Venturini , M. Davey , J. Jacobs , F. van Ex , A. Pasha , Y. Khedikar , S. J. Robinson , A. T. Cory , T. Florio , L. Concia , C. Juery , H. Schoonbeek , B. Steuernagel , D. Xiang , C. J. Ridout , B. Chalhoub , K. F. X. Mayer , M. Benhamed , D. Latrasse , A. Bendahmane , B. B. H. Wulff , R. Appels , V. Tiwari , R. Datla , F. Choulet , C. J. Pozniak , N. J. Provart , A. G. Sharpe , E. Paux , M. Spannagl , A. Bräutigam , C. Uauy , Science 2018, 361, aar6089.

[19] H. Yu , T. Lin , X. Meng , H. Du , J. Zhang , G. Liu , M. Chen , Y. Jing , L. Kou , X. Li , Q. Gao , Y. Liang , X. Liu , Z. Fan , Y. Liang , Z. Cheng , M. Chen , Z. Tian , Y. Wang , C. Chu , J. Zuo , J. Wan , Q. Qian , B. Han , A. Zuccolo , R A. Wing , C. Gao , C. Liang , J. Li , Cell 2021, 184, 1156. 33539781 10.1016/j.cell.2021.01.013

[20] J F. Gui , L. Zhou , X Y. Li , Water Biol. Secur. 2022, 1, 100002.

[21] H. Sun , W..B. Jiao , K. Krause , J A. Campoy , M. Goel , K. Folz‐Donahue , C. Kukat , B. Huettel , K. Schneeberger , Nat. Genet. 2022, 54, 342. 35241824 10.1038/s41588-022-01015-0 PMC8920897

[22] Y. Wang , R. R. Fuentes , W M. J. van Rengs , S. Effgen , M. W. A. M. Zaidan , R. Franzen , T. Susanto , J. B. Fernandes , R. Mercier , C J. Underwood , Nat. Genet. 2024, 56, 1075. 38741016 10.1038/s41588-024-01750-6 PMC11176054

[23] M J A. Awan , I. Amin , G. Hensel , S. Mansoor , Trends Plant Sci. 2024, 29, 1285. 39097426 10.1016/j.tplants.2024.07.010

[24] J F. Gui , Water Biol. Secur. 2024, 3, 100271.

[25] Y. Wang , Xi‐Y Li , W. J. Xu , K. Wang , B. Wu , M. Xu , Y. Chen , Li‐J Miao , Z. W. Wang , Z. Li , X. J. Zhang , Z. Yin , Bo‐T Zhou , Yu‐L Yang , C. L. Zhu , M. L. Hu , J. M. Zheng , C. G. Feng , Q. Qiu , Le‐T Tian , M. Lu , F. Peng , W. J. Lu , J. F. Tong , J. G. Tong , B‐De Fu , P. Yu , M. Ding , R. H. Gan , Q. Q. Zhang , et al., Nat. Ecol. Evol. 2022, 6, 1354. 35817827 10.1038/s41559-022-01813-z PMC9439954

[26] M. Ding , Xi‐Y Li , Z. X. Zhu , J. H. Chen , M. Lu , Q. Shi , Y. Wang , Z. Li , X. Zhao , T. Wang , W. X. Du , C. Miao , T‐Zi Yao , M. T. Wang , X. J. Zhang , Z. W. Wang , Li Zhou , J. F. Gui , PLoS Genet. 2021, 17, 1009760. 10.1371/journal.pgen.1009760 PMC8448357 34491994

[27] Z W. Wang , H P. Zhu , D. Wang , F F. Jiang , W. Guo , L. Zhou , J F. Gui , BMC Res. Notes 2011, 4, 82. 21439093 10.1186/1756-0500-4-82 PMC3072332

[28] M. Lu , Xi‐Y Li , Z. Li , W. X. Du , Li Zhou , Y. Wang , X. J. Zhang , Z. W. Wang , J. F. Gui , Sci. China Life Sci. 2020, 64, 77. 32529288 10.1007/s11427-020-1694-7

[29] M. Lu , Z. Li , Zi‐Yu Zhu , F. Peng , Y. Wang , Xi‐Y Li , Z. W. Wang , X. J. Zhang , Li Zhou , J. F. Gui , Mol. Biol. Evol. 2022, 39, msac18832. 10.1093/molbev/msac188 PMC9486886 36056821

[30] Q Y. Zhang , J F. Gui , Sci. China Life Sci. 2018, 61, 1486. 30443861 10.1007/s11427-018-9414-7

[31] Q Y. Zhang , F. Ke , L. Gui , Z. Zhao , Water Biol. Secur. 2022, 1, 100062.

[32] X‐Li Yang , Y. Wang , Z. Li , P. Yu , M. Lu , Xi‐Y Li , Z. W. Wang , X. J. Zhang , J. F. Gui , Li Zhou , Aquaculture 2023, 574, 739690.

[33] Q. Zhou , D. Tang , Wu Huang , Z. Yang , Yu Zhang , J P. Hamilton , R G. F. Visser , C W. B. Bachem , C. Robin Buell , Z. Zhang , C. Zhang , S. Huang , Nat. Genet. 2020, 52, 1018. 32989320 10.1038/s41588-020-0699-x PMC7527274

[34] S. Scalabrin , G. Magris , M. Liva , N. Vitulo , M. Vidotto , D. Scaglione , L. Del Terra , M. R. Ruosi , L. Navarini , G. Pellegrino , J. C. Berny Mier y Teran , L. Toniutti , F. Suggi Liverani , M. Cerutti , G. Di Gaspero , M. Morgante , Nat. Commun. 2024, 15, 1. 38263403 10.1038/s41467-023-44449-8 PMC10805892

[35] P. Lamelza , J M. Young , L M. Noble , L. Caro , A. Isakharov , M. Palanisamy , M V. Rockman , H S. Malik , M. Ailion , PLoS Genet. 2019, 15, 1008520. 10.1371/journal.pgen.1008520 PMC6946170 31841515

[36] P J. Van Dijk , D. Rigola , S E. Schauer , Curr. Biol. 2016, 26, R122. 26859270 10.1016/j.cub.2015.12.010

[37] D. Hojsgaard , M. Schartl , BioEssays 2021, 43, 2000111. 10.1002/bies.202000111 33169369

[38] C. Wang , Q. Liu , Yi Shen , Y. Hua , J. Wang , J. Lin , M. Wu , T. Sun , Z. Cheng , R. Mercier , K. Wang , Nat. Biotechnol. 2019, 37, 283. 30610223 10.1038/s41587-018-0003-0

[39] M. Lu , Q. C. Zhang , Zi‐Yu Zhu , F. Peng , Z. Li , Y. Wang , Xi‐Y Li , Z. W. Wang , X. J. Zhang , Li Zhou , J. F. Gui , Sci. Bull. 2023, 68, 1038. 10.1016/j.scib.2023.04.029 37173259

[40] C A. Rose‐Fricker , M L. Fraser , W A. Meyer , C R. Funk , Crop Sci. 2002, 42, 663. 10.2135/cropsci2002.3070 11756300

[41] C J. Underwood , R. Mercier , Annu. Rev. Plant Biol. 2022, 73, 201. 35138881 10.1146/annurev-arplant-102720-013958

[42] D. Mieulet , S. Jolivet , M. Rivard , L. Cromer , A. Vernet , P. Mayonove , L. Pereira , G. Droc , B. Courtois , E. Guiderdoni , R. Mercier , Cell Res. 2016, 26, 1242. 27767093 10.1038/cr.2016.117 PMC5099866

[43] K. Buyukcelebi , A J. Duval , F. Abdula , H. Elkafas , F. Seker‐Polat , M. Adli , Nat. Commun. 2024, 15, 1169. 38326302 10.1038/s41467-024-45382-0 PMC10850163

[44] E U. Hammarlund , E. Flashman , S. Mohlin , F. Licausi , Science 2020, 370, aba3512. 10.1126/science.aba3512 33093080

[45] C T. Taylor , S P. Colgan , Nat. Rev. Immunol. 2017, 17, 774. 28972206 10.1038/nri.2017.103 PMC5799081

[46] X. Liu , J H. Tang , Z X. Wang , C C. Zhu , H Y. Deng , X Y. Sun , G Q. Yu , F J. Rong , X Y. Chen , Q. Liao , S K. Jia , W. Liu , H Y. Zha , S J. Fan , X L. Cai , J F. Gui , W H. Xiao , Nat. Commun. 3533, 15, 3533. 38670937 10.1038/s41467-024-47814-3 PMC11053110

[47] W G. Kaelin , P J. Ratcliffe , Mol. Cell 2008, 30, 393. 18498744 10.1016/j.molcel.2008.04.009

[48] N. Masson , T P. Keeley , B. Giuntoli , M D. White , M. L. Puerta , P. Perata , R J. Hopkinson , E. Flashman , F. Licausi , P J. Ratcliffe , Science 2019, 365, 65. 31273118 10.1126/science.aaw0112 PMC6715447

[49] N. Liu , S. Xu , Q. Yao , Q. Zhu , Y. Kai , J Y. Hsu , P. Sakon , L. Pinello , G. C. Yuan , D E. Bauer , S H. Orkin , Nat. Genet. 2021, 53, 511. 33649594 10.1038/s41588-021-00798-y PMC8038971

[50] E. Lieberman‐Aiden , N L. van Berkum , L. Williams , M. Imakaev , T. Ragoczy , A. Telling , I. Amit , B R. Lajoie , P J. Sabo , M O. Dorschner , R. Sandstrom , B. Bernstein , M. A. Bender , M. Groudine , A. Gnirke , J. Stamatoyannopoulos , L A. Mirny , E S. Lander , J. Dekker , Science 2009, 326, 289. 19815776 10.1126/science.1181369 PMC2858594

[51] H. Li , Bioinformatics 2018, 34, 3094. 29750242 10.1093/bioinformatics/bty191 PMC6137996

[52] L. Li , C. J. Stoeckert, Jr, D. S. Roos , Genome Res. 2003, 13, 2178. 12952885 10.1101/gr.1224503 PMC403725

[53] T. Nakamura , D K. Yamada , K. Tomii , K. Katoh , Bioinformatics 2018, 34, 2490. 29506019 10.1093/bioinformatics/bty121 PMC6041967

[54] G. Talavera , J. Castresana , Syst. Biol. 2007, 56, 564. 17654362 10.1080/10635150701472164

[55] Z. Yang , Mol. Biol. Evol. 2007, 24, 1586. 17483113 10.1093/molbev/msm088

[56] H. Tang , J E. Bowers , X. Wang , X Y. Ming , M. Alam , A H. Paterson , Science 2008, 320, 486. 18436778 10.1126/science.1153917

[57] H Y. Cheng , G T. Concepcion , X W. Feng , H W. Zhang , H. Li , 2021, Nat. Methods 18, 1. 33526886 10.1038/s41592-020-01056-5 PMC7961889

[58] M. J. Jang , H. J. Cho , Y. S. Park , H. Y. Lee , E. K. Bae , S. Jung , H. Jin , J. Woo , E. Park , S. J. Kim , J. W. Choi , G. Y. Chae , Ji‐Y Guk , Do Y Kim , S. H. Kim , M. J. Kang , H. Lee , K. S. Cheon , In S Kim , Y. M. Kim , M. S. Kim , J. H. Ko , K. S. Kang , D. Choi , E. J. Park , S. Kim , Nat. Genet. 2024, 56, 2551. 39428511 10.1038/s41588-024-01944-y

[59] R H. Gan , Y. Wang , Z. Li , Z X. Yu , X Y. Li , J F. Tong , Z W. Wang , X J. Zhang , L. Zhou , J F. Gui , Mol. Biol. Evol. 2021, 38, 1995. 33432361 10.1093/molbev/msab002 PMC8097289

[60] F. X. Gao , Y. Wang , Qi‐Ya Zhang , C. Y. Mou , Z. Li , Y. S. Deng , Li Zhou , J. F. Gui , BMC Genomics 2017, 18, 561. 28738780 10.1186/s12864-017-3945-6 PMC5525251

[61] B. Li , C N. Dewey , BMC Bioinformatics 2011, 12, 323. 21816040 10.1186/1471-2105-12-323 PMC3163565

[62] X. Zhao , P. Psarianos , L. S. Ghoraie , K. Yip , D. Goldstein , R. Gilbert , I. Witterick , H. Pang , A. Hussain , Ju H Lee , J. Williams , S V. Bratman , L. Ailles , B. Haibe‐Kains , F. F. Liu , Nat. Metab. 2019, 1, 147. 32694814 10.1038/s42255-018-0008-5

[63] P. Danecek , A. Auton , G. Abecasis , C A. Albers , E. Banks , M A. DePristo , R E. Handsaker , G. Lunter , G T. Marth , S T. Sherry , G. McVean , R. Durbin , Bioinformatics 2011, 27, 2156.21653522

[64] G C. Yu , L G. Wang , Y Y. Han , Q Y. He , OMICS 2012, 16, 284. 22455463 10.1089/omi.2011.0118 PMC3339379

