# Supplementary material for: Manipulating Unisexual–Sexual Reproduction Transition to Engineer Genome‐Reconstructed Polyploids
Source: Adv Sci (Weinh). 2025 Jul 11;12(33):e06024. doi: 10.1002/advs.202506024 (PMC12412582; doi:10.1002/advs.202506024)
Supplement: Supplementary file 1 — Supporting Information [file ADVS-12-e06024-s001.pdf]

ADVANCED SCIENCE
Open Access

Supporting Information

for *Adv. Sci.*, DOI 10.1002/advs.202506024

Manipulating Unisexual–Sexual Reproduction Transition to Engineer Genome-Reconstructed Polyploids

Meng Lu, Qin-Can Zhang, Zi-Yu Zhu, Yang Wang, Zhong-Wei Wang, Xi-Yin Li, Zhi Li, Xiao-Juan Zhang, Li Zhou* and Jian-Fang Gui**

Supplementary Figures

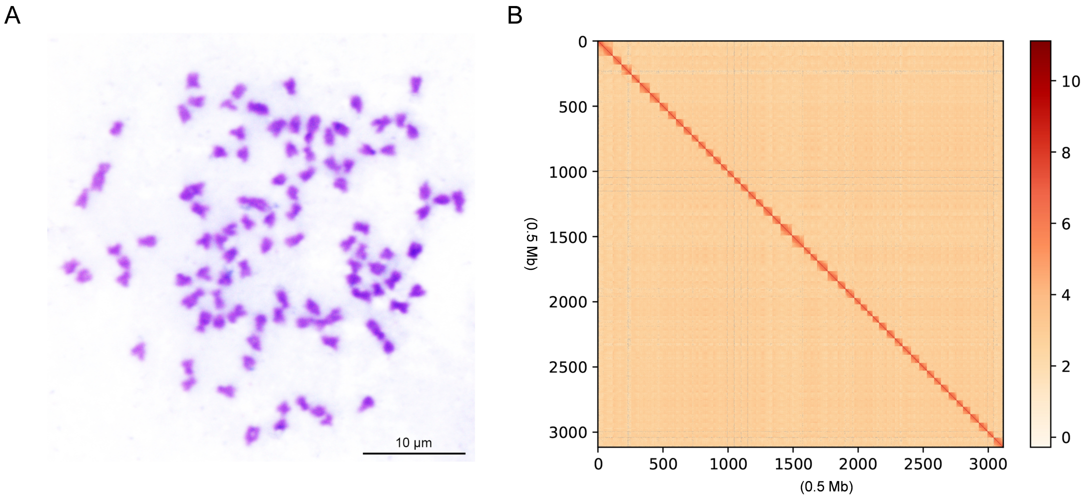

Supplementary Figure 1. Representative metaphase of *C. cuvieri* with 100 chromosomes (A) and Hi-C heatmap of the assembled *C. cuvieri* genome (B). The signal intensity of the Hi-C heatmap is expressed as $\log_{10}(Z+1)$. Z represents the calculated interaction intensity.

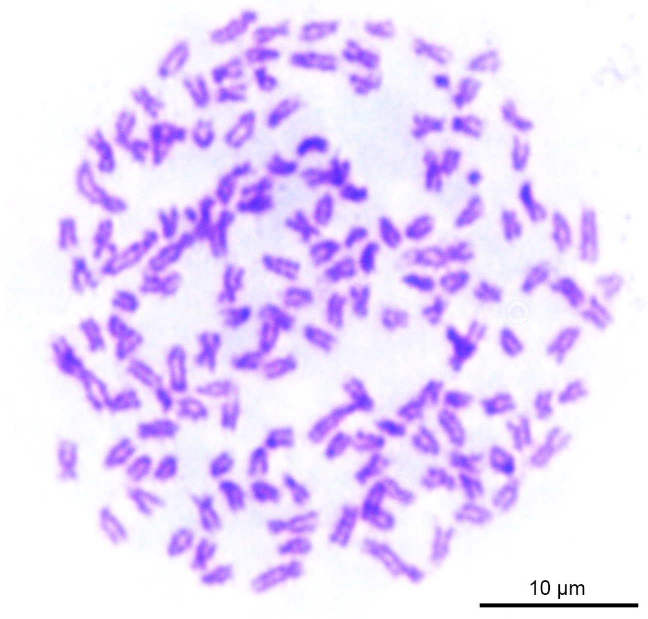

Supplementary Figure 2. Representative metaphase of the novel polyploid with approximately 150 chromosomes.

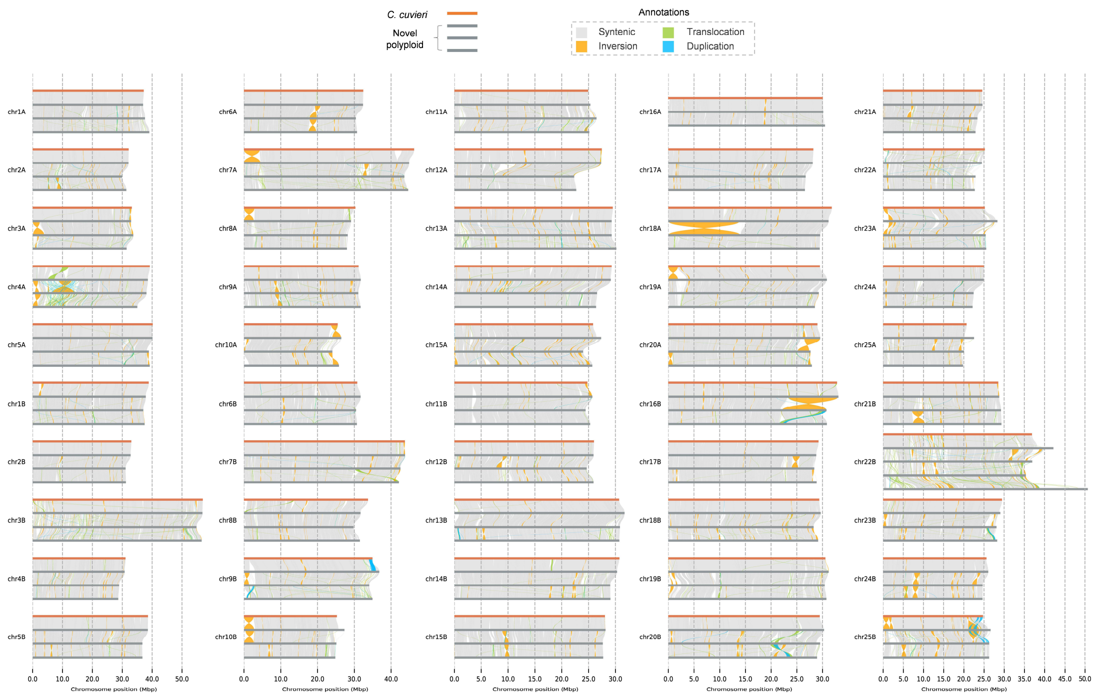

Supplementary Figure 3. Collinearity analysis of all 50 homologous chromosome groups between the three GR-A3n haplotypes with the *C. cuvieri* haplotype. The haplotypes are depicted as horizontal lines and consist of the *C. cuvieri* haplotype (orange line, top) and three GR-A3n haplotypes (gray lines, bottom).

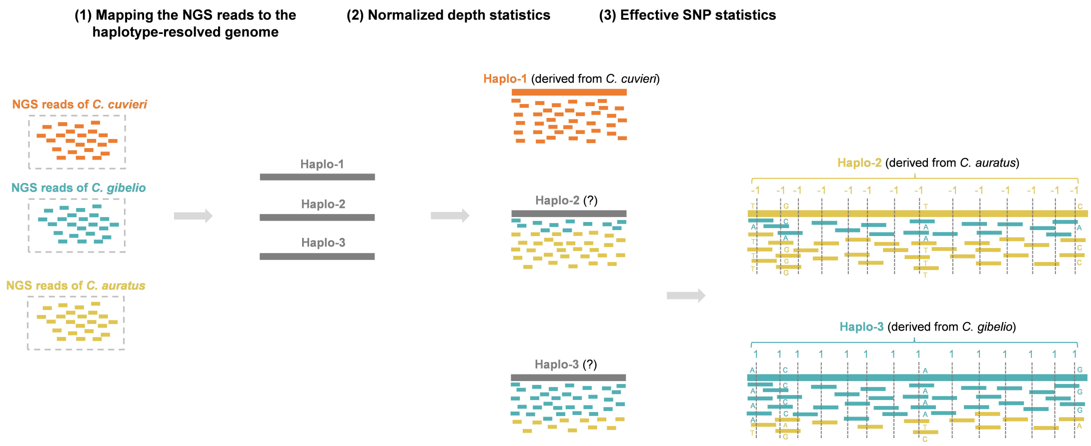

Supplementary Figure 4. Schematic diagram for haplotype genotyping was conducted based on normalized depth statistics and effective SNP statistics.

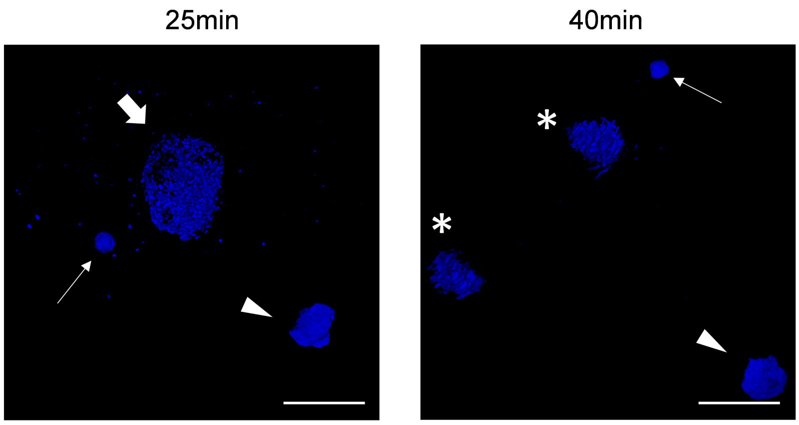

Supplementary Figure 5. Nuclear behaviors in the fertilized eggs of a GR-A3n female crossed with a *C. cuvieri* male at 25 and 40 min after fertilization. The sperm nucleus and female pronucleus are indicated by thin and thick arrows, respectively. The second polar body is indicated by triangular arrowheads, and the nuclei of the zygotes after the first mitosis are indicated by asterisks. A total of 30 fertilized eggs were observed. Scale bar, 10 μm.

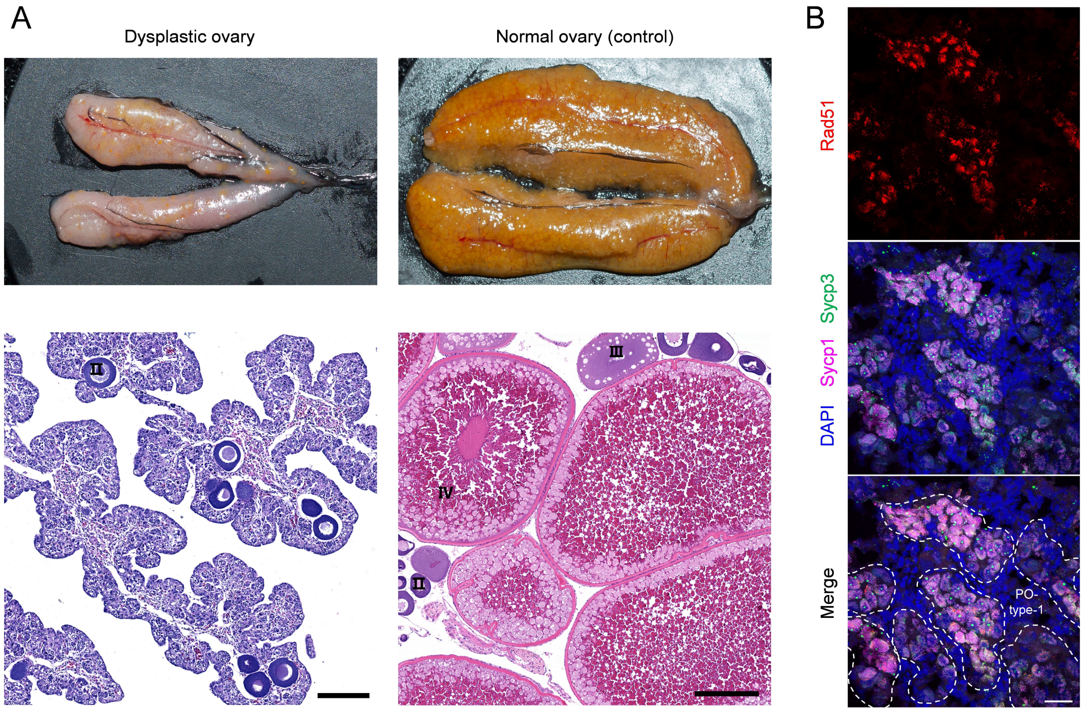

Supplementary Figure 6. Histological analysis of dysplastic ovaries in sterile GR-A3n females. (**A**) Histology structure of the dysplastic and normal ovaries. Og, oogonia; II, growth stage oocyte; III, vitellogenic oocyte; IV, maturing oocyte. Scale bar, 300 μm. (**B**) Immunohistochemical analysis of the dysplastic ovary by anti-Sycp1 (magenta), anti-Sycp3 (green), and anti-Rad51 (red) antibodies. Only one kind of primary oocyte was detected, and the development of primary oocytes was arrested in the dysplastic ovary (n=5). PO, primary oocyte. Scale bar, 25 μm.

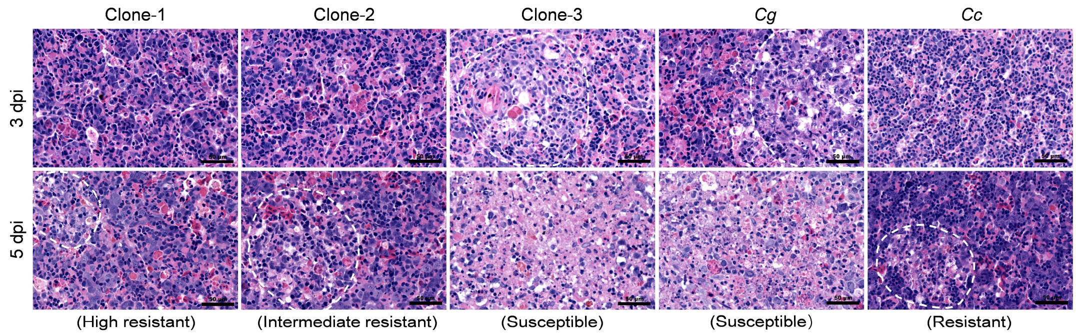

Supplementary Figure 7. Histopathological photographs of the head-kidney from infected individuals in the five groups at 3 and 5 dpi (n=5). The white dotted circles indicate the lesions caused by *Ca*HV infection. dpi, days post infection.

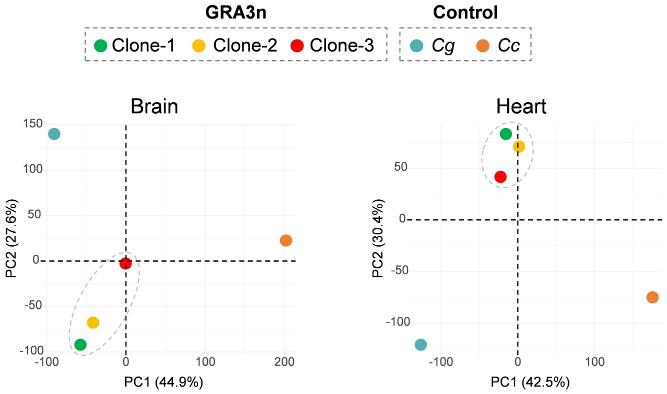

Supplementary Figure 8. Principal component analysis illustrating an overview of gene expression patterns in the brain and heart from the three GR-A3n clones, *C. cuvieri*, and *C. gibelio*. *Cc*, *C. cuvieri*; *Cg*, *C. gibelio*; GR-A3n, genome-reconstructed amphitriploid.

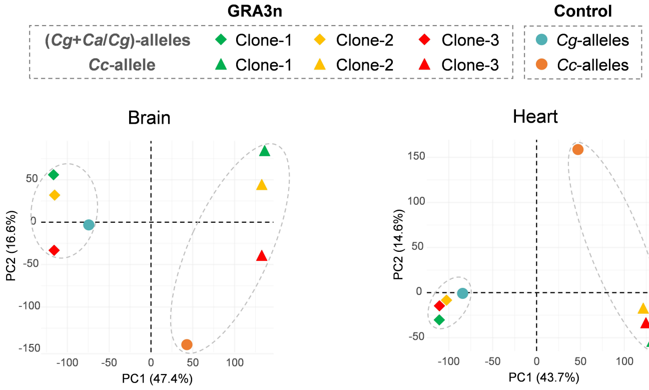

Supplementary Figure 9. Principal component analysis illustrating an overview of allele expression patterns in the brain and heart from the three GR-A3n clones; gene expression patterns of *C. cuvieri* and *C. gibelio* are shown as controls. *Cc*, *C. cuvieri*; *Cg*, *C. gibelio*; *Ca*, *C. auratus*; GR-A3n, genome-reconstructed amphitriploid.

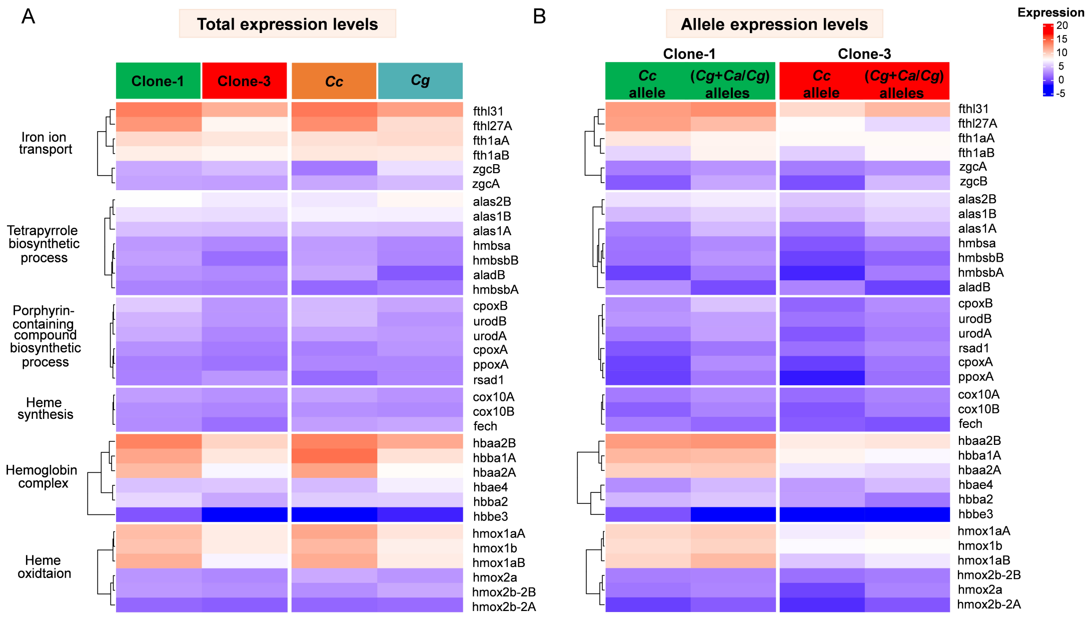

Supplementary Figure 10. Hemoglobin metabolism pathways in the spleen. (**A**) Heatmap of 38 expressed genes related to six main pathways of hemoglobin metabolism in the spleen of the resistant clone-1, susceptible clone-3, *C. cuvieri*, and *C. gibelio*. (**B**) Heatmap of the *Cc*-derived and (*Cg*+*Ca*/*Cg*)-derived alleles of 38 expressed genes related to six main pathways of hemoglobin metabolism in the spleen of clone-1 and clone-3. *Cc*, *C. cuvieri*; *Cg*, *C. gibelio*; *Ca*, *C. auratus*.

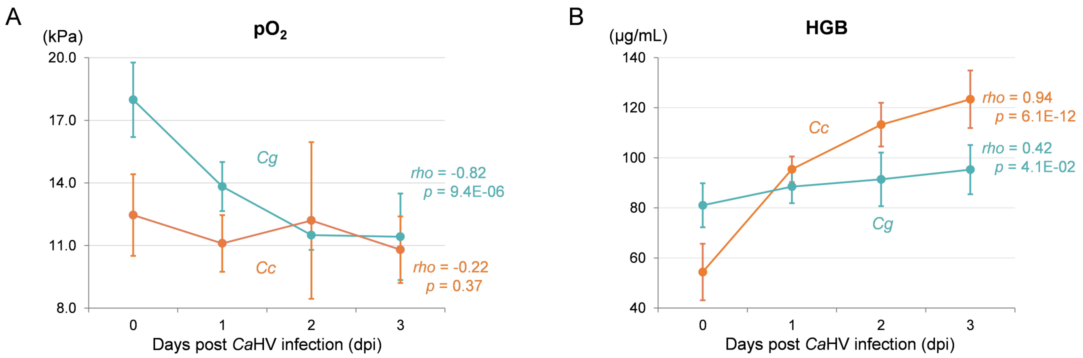

Supplementary Figure 11. Dynamic changes in the arterial partial pressure of oxygen (pO_2) (A) and hemoglobin concentration (HGB) (B) in *C. cuvieri* and *C. gibelio* during *Ca*HV infection. The values are the mean ± standard deviation for each time point (n = 4–6). The correlation between days of infection and pO_2 or HGB were marked by Spearman's Rho with the corresponding p-value.

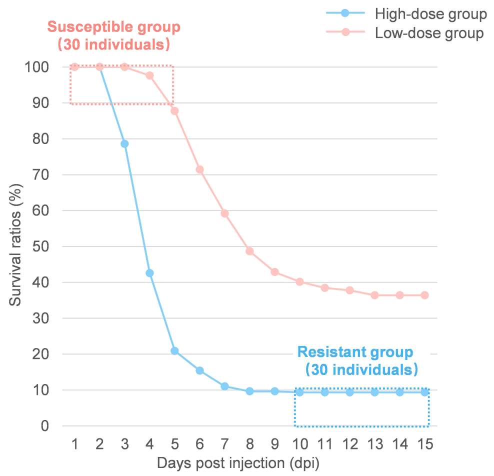

Supplementary Figure 12. Survival analysis of high and low-dose infection. The death rate in the high-dose group was significantly faster than that in the low-dose group, and 9.34% and 36.39% of individuals survived from high and low-dose infection, respectively. A total of 30 GR-A3n individuals (top 10%) with the longest survival time from high-dose group, and a total of 30 GR-A3n individuals (top 10%) with the shortest lethal time from low-dose group were selected for population genetic analysis.

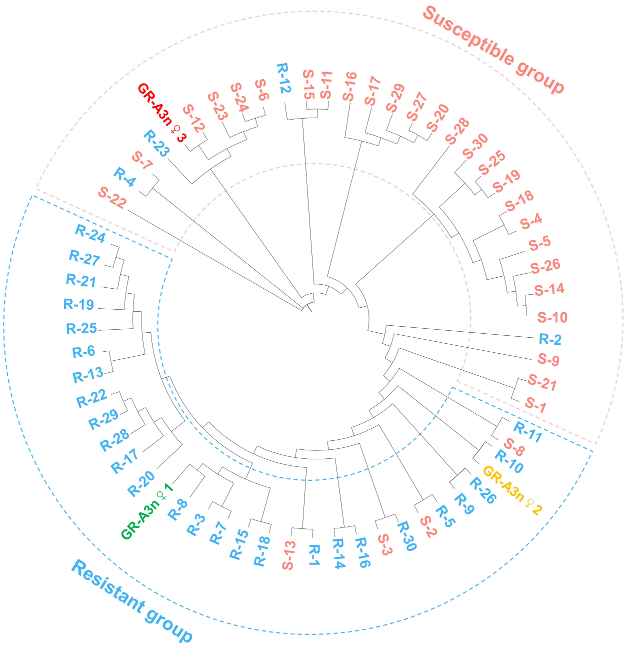

Supplementary Figure 13. Clustering analysis of haplotype in hotspot region among the maternal GR-A3ns of clone-1, clone-2, and clone-3 with the resistant and susceptible GR-A3n groups. The maternal GR-A3ns of clone-1 (GR-A3n♀1) and clone 2 (GR-A3n♀2) clustered with the most individuals of resistant group, while the maternal GR-A3n of clone-3 (GR-A3n♀3) clustered with the most individuals of susceptible group.

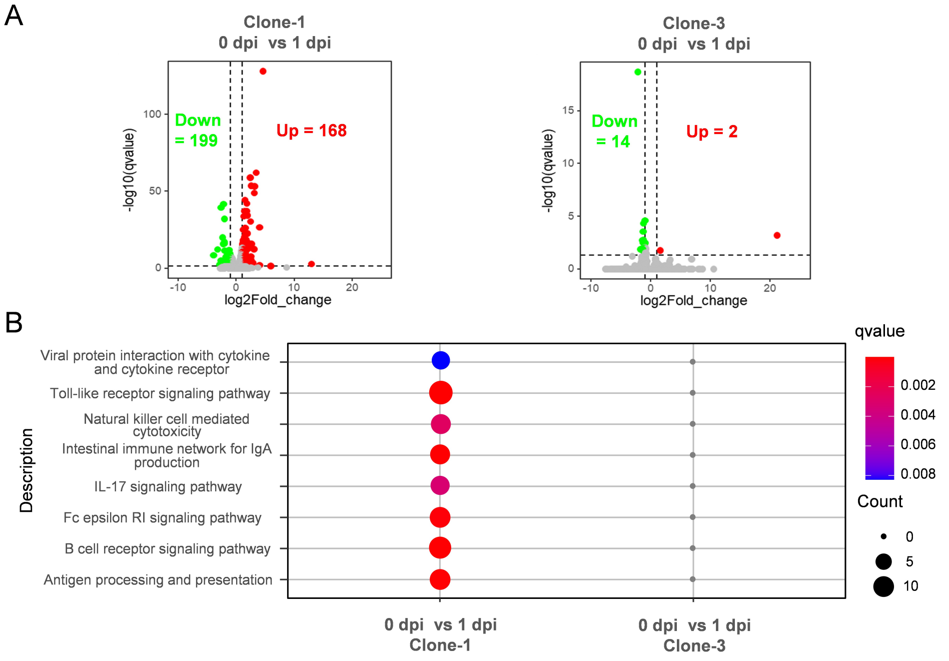

Supplementary Figure 14. Differentially expressed genes (DEGs) between the uninfected (0 dpi) and infected (1 dpi) head-kidney in clone-1 and clone-3. (**A**) The volcano maps show the number of DEGs in clone-1 (left) and clone-3 (right), respectively. The number of DEGs in clone-1 (n = 367) was significantly higher than that in clone-3 (n = 16). dpi, days post infection. (**B**) The bubble map shows the enrichment of DEGs in eight antiviral immune-related pathways. The number of DEGs in eight antiviral immune-related pathways of clone-1 was significantly higher than that in clone-3, which indicated that the activation of antiviral immune-related pathways in clone-1 was significantly faster than that in clone-3 at 1 dpi.

Supplementary Tables

Supplementary Table 1. Comparison of assembly and annotation of the genomes between *C. cuvieri* with *C. auratus* and *C. gibelio*.

		C. cuvieri	*C. auratus*	*C. gibelio*
Assembly	Genome size (bp)	1,601,185,401	1,522,436,151	1,589,834,947
	Gap length (bp)	31,500	255,950	534,155
	Pseudochromosomes size (bp)	1,557,952,734	1,476,454,911	1,502,175,770
	Scaffold number	702	231	312
	Scaffold N50 (bp)	30,569,300	29,195,226	28,762,065
	Scaffold L50	23	22	22
	Scaffold N90 (bp)	24,912,493	23,375,766	22,316,396
	Scaffold L90	46	46	47
	Contig number	1,017	1,332	2,757
	Contig N50 (bp)	9,293,763	3,878,405	1,709,526
	Contig L50	55	109	244
	Contig N90 (bp)	2,024,582	582,964	249,458
	Contig L90	192	474	1,198
	GC rate (%)	37.6	37.6	37.6
Annotation	Total gene number	42,794	44,164	45,130
	Gene number in pseudochromosomes	42,250	43,414	43,588
	BUSCO actinopterygii_odb10	97.1	97.5	97.4
	TEs rate (%)	54.70	43.62	44.86

Supplementary Table 2. Summary statistics of the haplotype-resolved genome assembly.

Mode	Total length (bp)	Total number	Total number (>= 2 kb)	Max length (bp)	N50 (bp)	N90 (bp)	GC content (%)
Hifiasm	5,317,089,085	19,382	19,382	56,849,150	29,309,304	49,552	37.56
Purge	4,976,191,239	7,639	7,639	56,849,150	29,559,034	22,529,892	37.9

Supplementary Table 3. Summary statistics of assembled sequence.

	Contig Length (bp)	Contig Number	Scaffold Length (bp)	Scaffold Number
N90	210,907	2,950	22,529,892	145
N80	713,055	1,653	25,548,554	124
N70	1,280,643	1,142	27,652,961	105
N60	1,809,999	816	28,857,858	88
N50	2,412,953	579	29,559,034	71
Total length	4,975,773,939	-	4,976,191,239	-
Number (>=100bp)	-	11,812	-	7,639
Number (>=2kb)	-	11,812	-	7,639
Max length	21,288,682	-	56,849,150	-

Supplementary Table 4. Summary statistics of assembled genome assisted by the Hi-C.

Genome version	Sequence set	Sequence length (bp)	Sequence number	Contig N50 (bp)	Scaffold N50 (bp)	Contig N90 (bp)	Scaffold N90 (bp)
Before mount	whole genome	5,316,671,785	23,555	2,216,636	2,216,636	49,403	49,403
After mount	whole genome	4,976,191,239	7,639	2,412,953	29,559,034	210,907	22,529,892
	chromosome	4,606,039,627	150	2,624,193	30,322,596	482,701	24,834,961
	dissociative sequence	370,151,612	7,489	47,994	47,997	32,906	32,918

Supplementary Table 5. Summary statistics of the MGI sequencing data.

Samples	Raw_Base	Clean_Base	Clean_Base_Percent	GC_Content	>Q20	>Q30
Cc♀ (Maternal parent of novel amphitriploid)	175436595400 (175.44G)	173687715495 (173.69G)	99.00%	38.64%	97.97%	94.13%
*C*g♀ (Maternal parent of amphitetraploid)	260666991300 (260.67G)	254567689490 (254.57G)	97.66%	39.30%	97.96%	94.36%
Ca♂ (Paternal parent of amphitetraploid)	174174669900 (174.17G)	158043765434 (158.04G)	90.74%	38.11%	95.63%	89.53%

Supplementary Table 6. Ratio of normal ovary in three GR-A3n groups.

Crossed combination	Female with normal ovary	Female with dysplastic ovary	Normal/Total
Cc♀1 × A4n♂1	27	23	54.0%
Cc♀2 × A4n♂1	24	19	55.8%
Cc♀3 × A4n♂1	16	15	51.6%
Average	-	-	53.8%
Standard deviation	-	-	1.7%

Supplementary Table 7. Fertilization rate and survival rate of three crossed groups.

Crossed combination	Fertilization rate	Survival rate
GR-A3n♀1 × *Cc*♂1	84.5%	95.5%
GR-A3n♀2 × *Cc*♂1	88.5%	83.0%
GR-A3n♀3 × *Cc*♂1	80.5%	76.0%
Average rate	84.5%	84.8%
Standard deviation	3.3%	8.1%

Supplementary Table 8. Summary statistics of the MGI sequencing data.

Platform	Samples	Raw_Base (Gb)	Clean_Base (Gb)	Clean_Base_Percent (%)	>Q20 (%)	>Q30 (%)
MGI (DNBSEQ-T7)	GR-A3n♀1	60.46	60.46	100	97.66	92.84
	Clone-1-1	64.58	64.58	100	99.16	97.32
	Clone-1-2	82.76	82.76	100	99.25	97.58
	Clone-1-3	65.31	65.31	100	99.17	97.36
	Clone-1-4	56.2	56.20	100	99.13	97.23
	Clone-1-5	60.2	60.20	100	99.11	97.19
	Clone-1-6	54.27	54.27	100	98.98	96.81
	Clone-1-7	56.76	56.76	100	98.97	96.71
	Clone-1-8	54.35	54.35	100	99.04	96.94
	Clone-1-9	78.69	78.69	100	99.18	97.39
	Clone-1-10	73.96	73.96	100	99.23	97.55
	GR-A3n♀2	62.25	62.23	99.97	98.21	94.11
	Clone-2-1	60.23	60.13	99.84	97.15	91.91
	Clone-2-2	62.09	62.08	99.98	97.93	93.29
	Clone-2-3	61.57	61.47	99.83	97.45	92.79
	Clone-2-4	57.74	57.63	99.81	97.71	93.46
	Clone-2-5	62.31	62.29	99.97	98.25	94.33
	Clone-2-6	59.26	59.11	99.75	97.79	93.65
	Clone-2-7	60.27	60.26	99.98	98.07	93.76
	Clone-2-8	62.71	62.60	99.83	97.1	91.85
	Clone-2-9	64.87	64.77	99.85	97.09	91.75
	Clone-2-10	59.20	59.10	99.83	97.16	91.93
	GR-A3n♀3	60.36	60.36	100	97.35	91.94
	Clone-3-1	60.36	60.36	100	99.19	97.42
	Clone-3-2	66.11	66.11	100	99.21	97.46
	Clone-3-3	56.47	56.47	100	99.21	97.5
	Clone-3-4	55.01	55.01	100	99.03	96.88
	Clone-3-5	58.14	58.14	100	99.07	97.02
	Clone-3-6	54.69	54.69	100	99.09	97.15
	Clone-3-7	54.95	54.95	100	99.13	97.23
	Clone-3-8	60.8	60.8	100	99.12	97.16
	Clone-3-9	60.46	60.46	100	99.14	97.27
	Clone-3-10	63.17	63.17	100	99.1	97.13

Supplementary Table 9. Summary statistics of the transcriptome data.

Platform	Samples	Raw_Read (M)	Clean_Read (M)	Clean_Base (G)	Clean_Read_Percent (%)	>Q20 (%)	>Q30 (%)
	Cc-B-1	77.97	77.97	11.53	100.00	99.43	98.08
	Cc-B-2	84.59	84.59	12.51	100.00	99.42	98.06
	Cc-B-3	86.66	86.66	12.81	100.00	99.43	98.12
	Cc-H-1	97.32	97.32	14.41	100.00	99.47	98.22
	Cc-H-2	93.09	93.09	13.81	100.00	99.39	97.96
	Cc-H-3	93.22	93.22	13.81	100.00	99.44	98.12
	Cc-Hk-1	74.93	73.56	10.97	98.17	96.95	91.59
	Cc-Hk-2	81.43	79.44	11.84	97.55	96.38	90.42
	Cc-Hk-3	76.52	75.08	11.20	98.13	96.26	90.08
	Cc-L-1	104.45	104.45	15.45	100.00	99.43	98.09
	Cc-L-2	102.20	102.20	15.11	100.00	99.51	98.31
	Cc-L-3	92.37	92.37	13.70	100.00	99.49	98.25
	Cc-M-1	89.54	89.54	13.29	100.00	99.48	98.20
	Cc-M-2	96.39	96.39	14.27	100.00	99.50	98.27
	Cc-M-3	102.43	102.43	15.12	100.00	99.44	98.08
	Cc-S-1	80.82	80.82	12.05	100.00	98.94	96.57
	Cc-S-2	76.33	76.33	11.40	100.00	98.69	95.82
	Cc-S-3	82.49	82.49	12.21	100.00	98.97	96.71
	Cg-B-1	82.79	82.79	12.28	100.00	99.46	98.19
MGI	*Cg*-B-2	90.94	90.94	13.49	100.00	99.45	98.16
(DNBSEQ-T7)	*Cg*-B-3	96.98	96.98	14.37	100.00	99.47	98.22
	Cg-H-1	101.40	101.40	15.04	100.00	99.47	98.23
	Cg-H-2	97.05	97.05	14.38	100.00	99.47	98.21
	Cg-H-3	97.30	97.30	14.45	100.00	99.47	98.23
	Cg-Hk-1	66.18	64.76	9.64	97.85	96.67	91.02
	Cg-Hk-2	66.45	64.86	9.67	97.60	96.34	90.39
	Cg-Hk-3	80.59	77.24	11.51	95.84	96.30	90.40
	Cg-L-1	101.29	101.29	15.02	100.00	99.46	98.16
	Cg-L-2	88.36	88.36	13.14	100.00	99.50	98.29
	Cg-L-3	91.44	91.44	13.60	100.00	99.49	98.24
	Cg-M-1	76.32	76.32	11.33	100.00	99.44	98.10
	Cg-M-2	85.02	85.02	12.63	100.00	99.47	98.19
	Cg-M-3	78.76	78.76	11.73	100.00	99.44	98.10
	Cg-S-1	84.01	84.01	12.50	100.00	98.87	96.37
	Cg-S-2	78.31	78.31	11.66	100.00	98.90	96.47
	Cg-S-3	80.91	80.91	12.04	100.00	98.96	96.63
	Clone-1-B-1	94.64	94.64	14.00	100.00	99.46	98.19
	Clone-1-B-2	100.17	100.17	14.88	100.00	99.42	98.08
	Clone-1-B-3	76.61	76.61	11.37	100.00	99.45	98.16
	Clone-1-H-1	97.91	97.91	14.50	100.00	99.45	98.18
	Clone-1-H-2	95.82	95.82	14.21	100.00	99.46	98.17

	Clone-1-H-3	103.75	103.75	15.38	100.00	99.46	98.19
	Clone-1-Hk-1	76.95	75.18	11.21	97.69	96.14	89.98
	Clone-1-Hk-2	80.71	78.57	11.71	97.35	96.40	90.49
	Clone-1-Hk-3	81.24	79.60	11.86	97.98	96.50	90.71
	Clone-1-L-1	95.52	95.52	14.22	100.00	99.54	98.45
	Clone-1-L-2	91.75	91.75	13.65	100.00	99.48	98.21
	Clone-1-L-3	81.91	81.91	12.20	100.00	99.46	98.16
	Clone-1-M-1	96.83	96.83	14.34	100.00	99.48	98.22
	Clone-1-M-2	96.99	96.99	14.40	100.00	99.45	98.11
	Clone-1-M-3	98.04	98.04	14.52	100.00	99.46	98.16
	Clone-1-S-1	80.99	80.99	12.05	100.00	98.92	96.53
	Clone-1-S-2	80.59	80.59	12.00	100.00	98.93	96.55
	Clone-1-S-3	81.92	81.92	12.21	100.00	98.98	96.69
	Clone-2-B-1	93.69	93.69	13.87	100.00	99.41	98.04
	Clone-2-B-2	96.73	96.73	14.35	100.00	99.43	98.10
	Clone-2-B-3	108.12	108.12	15.96	100.00	99.46	98.20
	Clone-2-H-1	89.75	89.75	13.33	100.00	99.46	98.19
	Clone-2-H-2	87.02	87.02	12.90	100.00	99.48	98.25
	Clone-2-H-3	102.76	102.76	15.17	100.00	99.47	98.21
	Clone-2-Hk-1	70.42	68.62	10.22	97.44	96.31	90.33
	Clone-2-Hk-2	72.48	70.60	10.52	97.41	96.20	90.10
MGI	Clone-2-Hk-3	70.04	68.50	10.22	97.81	96.60	90.91
(DNBSEQ-T7)	Clone-2-L-1	107.71	107.71	16.00	100.00	99.48	98.21
	Clone-2-L-2	94.83	94.83	14.11	100.00	99.46	98.17
	Clone-2-L-3	93.73	93.73	13.94	100.00	99.49	98.25
	Clone-2-M-1	90.86	90.86	13.47	100.00	99.47	98.18
	Clone-2-M-2	98.03	98.03	14.54	100.00	99.49	98.24
	Clone-2-M-3	100.69	100.69	14.91	100.00	99.43	98.06
	Clone-2-S-1	83.97	83.97	12.50	100.00	98.97	96.67
	Clone-2-S-2	82.61	82.61	12.24	100.00	98.93	96.57
	Clone-2-S-3	81.74	81.74	12.12	100.00	98.98	96.72
	Clone-3-B-1	114.44	114.44	16.92	100.00	99.50	98.36
	Clone-3-B-2	100.36	100.36	14.84	100.00	99.44	98.15
	Clone-3-B-3	98.84	98.84	14.63	100.00	99.46	98.21
	Clone-3-H-1	96.87	96.87	14.40	100.00	99.48	98.25
	Clone-3-H-2	91.22	91.22	13.52	100.00	99.49	98.29
	Clone-3-H-3	79.50	79.50	11.77	100.00	99.50	98.32
	Clone-3-Hk-1	73.86	71.91	10.72	97.36	96.58	90.84
	Clone-3-Hk-2	77.08	75.43	11.25	97.87	96.47	90.65
	Clone-3-Hk-3	73.89	72.15	10.76	97.65	96.17	90.06
	Clone-3-L-1	98.12	98.12	14.62	100.00	99.46	98.16
	Clone-3-L-2	99.51	99.51	14.76	100.00	99.49	98.26
	Clone-3-L-3	68.58	68.58	10.17	100.00	99.51	98.32
	Clone-3-M-1	96.07	96.07	14.28	100.00	99.44	98.08

MGI (DNBSEQ-T7)	Clone-3-M-2	96.28	96.28	14.29	100.00	99.46	98.17
	Clone-3-M-3	78.42	78.42	11.65	100.00	99.43	98.06
	Clone-3-S-1	78.77	78.77	11.73	100.00	98.92	96.56
	Clone-3-S-2	73.71	73.71	10.97	100.00	98.97	96.68
	Clone-3-S-3	78.43	78.43	11.67	100.00	98.94	96.59

Supplementary Table 10. Proportion of three allele expression categories across the six tissues in the three GR-A3n clones.

		Balanced	*Cc*-dominant	*Cg*-dominant
Brain	Clone-1	63.7%	16.1%	20.1%
	Clone-2	65.1%	16.4%	18.5%
	Clone-3	63.1%	16.6%	20.3%
	Mean	64.0%	16.4%	19.6%
Heart	Clone-1	61.6%	17.8%	20.7%
	Clone-2	63.4%	17.8%	19.3%
	Clone-3	61.3%	17.8%	20.2%
	Mean	62.1%	17.8%	20.1%
Head-kidney	Clone-1	63.2%	16.7%	20.0%
	Clone-2	64.2%	16.4%	19.4%
	Clone-3	62.8%	18.0%	19.2%
	Mean	63.4%	17.1%	19.5%
Liver	Clone-1	57.6%	18.6%	23.8%
	Clone-2	58.8%	19.0%	22.2%
	Clone-3	57.7%	18.8%	23.6%
	Mean	58.0%	18.8%	23.2%
Muscle	Clone-1	60.1%	17.3%	22.6%
	Clone-2	61.0%	18.0%	21.0%
	Clone-3	59.8%	17.3%	22.8%
	Mean	60.3%	17.6%	22.1%
Spleen	Clone-1	66.3%	14.8%	18.9%
	Clone-2	66.5%	14.9%	18.6%
	Clone-3	66.6%	15.8%	17.6%
	Mean	66.5%	15.2%	18.4%

Supplementary Table 11-1. GO enrichment of DEGs in cluster 1 of head-kidney.

ID	Description	Gene Ratio	BgRatio	pvalue	p.adjust	qvalue	Count	*C.gibelio*_homolog_id	*C.cuvier*i_homolog_id
GO:0006260	DNA replication	13/603	75/29370	4.002E-09	1.405E-06	1.108E-06	13	gene_Cg_evm.model.chr5A.149/gene_Cg_evm.model.chr4A.771/gene_Cg_evm.model.chr5B.1004/gene_Cg_evm.model.chr22B.115/gene_Cg_evm.model.chr8B.246/gene_Cg_evm.model.chr6A.236/gene_Cg_evm.model.chr18A.381/gene_Cg_evm.model.chr19B.144/gene_Cg_evm.model.chr12A.179/gene_Cg_evm.model.chr24B.493/gene_Cg_evm.model.chr10A.323/gene_Cg_evm.model.chr24A.451/gene_Cg_evm.model.chr21A.367	gene_Car0037540/gene_Car0053560/gene_Car0076170/gene_Car0088780/gene_Car0105490/gene_Car0150890/gene_Car0165910/gene_Car0213080/gene_Car0330430/gene_Car0351180/gene_Car0355560/gene_Car0386090/gene_Car0413050
GO:0006457	protein folding	13/603	107/29370	3.145E-07	4.309E-05	3.399E-05	13	gene_Cg_evm.model.chr1B.128/gene_Cg_evm.model.chr1B.367/gene_Cg_evm.model.chr1B.450/gene_Cg_evm.model.chr1A.547/gene_Cg_evm.model.chr1A.622/gene_Cg_evm.model.chr22B.859/gene_Cg_evm.model.chr9B.361/gene_Cg_evm.model.chr9A.284/gene_Cg_evm.model.chr23B.33/gene_Cg_evm.model.chr14A.511/gene_Cg_evm.model.chr20A.777/gene_Cg_evm.model.chr12A.54/gene_Cg_evm.model.chr21A.505	gene_Car0056970/gene_Car0059390/gene_Car0060290/gene_Car0083350/gene_Car0084070/gene_Car0093240/gene_Car0100170/gene_Car0174540/gene_Car0269590/gene_Car0279080/gene_Car0293160/gene_Car0331620/gene_Car0411580
GO:0051082	unfolded protein binding	11/603	76/29370	4.272E-07	4.309E-05	3.399E-05	11	gene_Cg_evm.model.chr1B.128/gene_Cg_evm.model.chr1B.367/gene_Cg_evm.model.chr1B.450/gene_Cg_evm.model.chr1A.547/gene_Cg_evm.model.chr1A.622/gene_Cg_evm.model.chr22B.859/gene_Cg_evm.model.chr9B.636/gene_Cg_evm.model.chr23B.33/gene_Cg_evm.model.chr14A.511/gene_Cg_evm.model.chr20A.777/gene_Cg_evm.model.chr21A.505	gene_Car0056970/gene_Car0059390/gene_Car0060290/gene_Car0083350/gene_Car0084070/gene_Car0093240/gene_Car0097440/gene_Car0269590/gene_Car0279080/gene_Car0293160/gene_Car0411580
GO:0005833	hemoglobin complex	6/603	16/29370	4.911E-07	4.309E-05	3.399E-05	6	gene_Cg_evm.model.chr3B.1284/gene_Cg_evm.model.chr3B.1287/gene_Cg_evm.model.chr3A.773/gene_Cg_evm.model.chr3A.774/gene_Cg_evm.model.chr12A.417/gene_Cg_evm.model.chr12B.438	gene_Car0010720/gene_Car0010750/gene_Car0120620/gene_Car0120630/gene_Car0328130/gene_Car0335110
GO:0006418	tRNA aminoacylation for protein translation	9/603	55/29370	1.674E-06	0.0001115	8.791E-05	9	gene_Cg_evm.model.chr7A.1007/gene_Cg_evm.model.chr8B.72/gene_Cg_evm.model.chr16B.376/gene_Cg_evm.model.chr6A.813/gene_Cg_evm.model.chr19B.226/gene_Cg_evm.model.chr14A.607/gene_Cg_evm.model.chr17B.820/gene_Cg_evm.model.chr21B.95/gene_Cg_evm.model.chr21A.666	gene_Car0023810/gene_Car0103680/gene_Car0140670/gene_Car0144700/gene_Car0213830/gene_Car0278120/gene_Car0284920/gene_Car0308680/gene_Car0409980
GO:0000166	nucleotide binding	16/603	188/29370	1.905E-06	0.0001115	8.791E-05	16	gene_Cg_evm.model.chr7A.1007/gene_Cg_evm.model.chr8B.72/gene_Cg_evm.model.chr8B.121/gene_Cg_evm.model.chr2B.157/gene_Cg_evm.model.chr16B.376/gene_Cg_evm.model.chr6A.813/gene_Cg_evm.model.chr18A.381/gene_Cg_evm.model.chr19B.226/gene_Cg_evm.model.chr20B.710/gene_Cg_evm.model.chr14A.607/gene_Cg_evm.model.chr17B.820/gene_Cg_evm.model.chr20A.680/gene_Cg_evm.model.chr21B.95/gene_Cg_evm.model.chr15B.719/gene_Cg_evm.model.chr24A.256/gene_Cg_evm.model.chr21A.666	gene_Car0023810/gene_Car0103680/gene_Car0104180/gene_Car0132680/gene_Car0140670/gene_Car0144700/gene_Car0165910/gene_Car0213830/gene_Car0254680/gene_Car0278120/gene_Car0284920/gene_Car0294210/gene_Car0308680/gene_Car0323930/gene_Car0384170/gene_Car0409980
GO:0140662	ATP-dependent protein folding chaperone	9/603	61/29370	4.093E-06	0.0001807	0.0001426	9	gene_Cg_evm.model.chr3B.841/gene_Cg_evm.model.chr9B.361/gene_Cg_evm.model.chr9A.284/gene_Cg_evm.model.chr23B.33/gene_Cg_evm.model.chr17B.143/gene_Cg_evm.model.chr20A.777/gene_Cg_evm.model.chr21B.721/gene_Cg_evm.model.chr17A.135/gene_Cg_evm.model.chr21A.710	gene_Car0006710/gene_Car0100170/gene_Car0174540/gene_Car0269590/gene_Car0291640/gene_Car0293160/gene_Car0301370/gene_Car0315830/gene_Car0409540
GO:0015671	oxygen transport	6/603	22/29370	4.119E-06	0.0001807	0.0001426	6	gene_Cg_evm.model.chr3B.1284/gene_Cg_evm.model.chr3B.1287/gene_Cg_evm.model.chr3A.773/gene_Cg_evm.model.chr3A.774/gene_Cg_evm.model.chr12A.417/gene_Cg_evm.model.chr12B.438	gene_Car0010720/gene_Car0010750/gene_Car0120620/gene_Car0120630/gene_Car0328130/gene_Car0335110
GO:0004812	aminoacyl-tRNA ligase activity	9/603	62/29370	4.702E-06	0.0001834	0.0001446	9	gene_Cg_evm.model.chr7A.1007/gene_Cg_evm.model.chr8B.72/gene_Cg_evm.model.chr16B.376/gene_Cg_evm.model.chr6A.813/gene_Cg_evm.model.chr19B.226/gene_Cg_evm.model.chr14A.607/gene_Cg_evm.model.chr17B.820/gene_Cg_evm.model.chr21B.95/gene_Cg_evm.model.chr21A.666	gene_Car0023810/gene_Car0103680/gene_Car0140670/gene_Car0144700/gene_Car0213830/gene_Car0278120/gene_Car0284920/gene_Car0308680/gene_Car0409980
GO:0019825	oxygen binding	6/603	27/29370	1.498E-05	0.0005257	0.0004146	6	gene_Cg_evm.model.chr3B.1284/gene_Cg_evm.model.chr3B.1287/gene_Cg_evm.model.chr3A.773/gene_Cg_evm.model.chr3A.774/gene_Cg_evm.model.chr12A.417/gene_Cg_evm.model.chr12B.438	gene_Car0010720/gene_Car0010750/gene_Car0120620/gene_Car0120630/gene_Car0328130/gene_Car0335110
GO:0005839	proteasome core complex	7/603	42/29370	2.147E-05	0.0006281	0.0004954	7	gene_Cg_evm.model.chr2A.271/gene_Cg_evm.model.chr19B.305/gene_Cg_evm.model.chr19B.468/gene_Cg_evm.model.chr23B.836/gene_Cg_evm.model.chr17A.583/gene_Cg_evm.model.chr25B.164/gene_Cg_evm.model.chr21A.171	gene_Car0159930/gene_Car0214550/gene_Car0216180/gene_Car0261920/gene_Car0311190/gene_Car0407620/gene_Car0415010

GO:0006413	translational initiation	7/603	42/29370	2.147E-05	0.0006281	0.0004954	7	gene_Cg_evm.model.chr3A.706/gene_Cg_evm.model.chr8A.152/gene_Cg_evm.model.chr8A.193/gene_Cg_evm.model.chr20B.251/gene_Cg_evm.model.chr24B.461/gene_Cg_evm.model.chr10A.112/gene_Cg_evm.model.chr10B.111	gene_Car0120010/gene_Car0221780/gene_Car0222200/gene_Car0259180/gene_Car0350840/gene_Car0353670/gene_Car0360770
GO:0006270	DNA replication initiation	5/603	18/29370	2.463E-05	0.0006651	0.0005246	5	gene_Cg_evm.model.chr4A.771/gene_Cg_evm.model.chr22B.115/gene_Cg_evm.model.chr6A.236/gene_Cg_evm.model.chr24B.493/gene_Cg_evm.model.chr24A.451	gene_Car0053560/gene_Car0088780/gene_Car0150890/gene_Car0351180/gene_Car0386090
GO:0051603	proteolysis involved in protein catabolic process	7/603	44/29370	2.944E-05	0.000738	0.0005821	7	gene_Cg_evm.model.chr2A.271/gene_Cg_evm.model.chr19B.305/gene_Cg_evm.model.chr19B.468/gene_Cg_evm.model.chr23B.836/gene_Cg_evm.model.chr17A.583/gene_Cg_evm.model.chr25B.164/gene_Cg_evm.model.chr21A.171	gene_Car0159930/gene_Car0214550/gene_Car0216180/gene_Car0261920/gene_Car0311190/gene_Car0407620/gene_Car0415010
GO:0008199	ferric iron binding	4/603	10/29370	3.348E-05	0.0007835	0.000618	4	gene_Cg_evm.model.chr7A.197/gene_Cg_evm.model.chr7B.273/gene_Cg_evm.model.chr3A.515/gene_Cg_evm.model.chr24B.120	gene_Car0015970/gene_Car0027570/gene_Car0118110/gene_Car0347410
GO:0003697	single-stranded DNA binding	6/603	32/29370	4.202E-05	0.0008927	0.0007041	6	gene_Cg_evm.model.chr4A.771/gene_Cg_evm.model.chr20B.710/gene_Cg_evm.model.chr20A.680/gene_Cg_evm.model.chr12A.179/gene_Cg_evm.model.chr10A.164/gene_Cg_evm.model.chr10B.164	gene_Car0053560/gene_Car0254680/gene_Car0294210/gene_Car0330430/gene_Car0354140/gene_Car0361260
GO:0030527	structural constituent of chromatin	8/603	63/29370	4.323E-05	0.0008927	0.0007041	8	gene_Cg_evm.model.chr7B.1153/gene_Cg_evm.model.chr5A.449/gene_Cg_evm.model.chr5B.330/gene_Cg_evm.model.chr8B.397/gene_Cg_evm.model.chr3A.752/gene_Cg_evm.model.chr10A.127/gene_Cg_evm.model.chr25B.707/gene_Cg_evm.model.chr25A.532	gene_Car0035770/gene_Car0040700/gene_Car0069320/gene_Car0107130/gene_Car0120400/gene_Car0353810/gene_Car0402180/gene_Car0417330
GO:0033014	tetrapyrrole biosynthetic process	4/603	11/29370	5.176E-05	0.0010094	0.0007961	4	gene_Cg_evm.model.chr5B.329/gene_Cg_evm.model.chr8B.578/gene_Cg_evm.model.chr15B.414/gene_Cg_evm.model.chr15A.346	gene_Car0069310/gene_Car0109010/gene_Car0320940/gene_Car0342890
GO:0042555	MCM complex	4/603	12/29370	7.638E-05	0.0014111	0.0011129	4	gene_Cg_evm.model.chr22B.115/gene_Cg_evm.model.chr6A.236/gene_Cg_evm.model.chr24B.493/gene_Cg_evm.model.chr24A.451	gene_Car0088780/gene_Car0150890/gene_Car0351180/gene_Car0386090
GO:0000786	nucleosome	8/603	70/29370	9.295E-05	0.0016312	0.0012866	8	gene_Cg_evm.model.chr7B.1153/gene_Cg_evm.model.chr5A.449/gene_Cg_evm.model.chr5B.330/gene_Cg_evm.model.chr8B.397/gene_Cg_evm.model.chr3A.752/gene_Cg_evm.model.chr10A.127/gene_Cg_evm.model.chr25B.707/gene_Cg_evm.model.chr25A.532	gene_Car0035770/gene_Car0040700/gene_Car0069320/gene_Car0107130/gene_Car0120400/gene_Car0353810/gene_Car0402180/gene_Car0417330
GO:0006879	intracellular iron ion homeostasis	4/603	15/29370	0.0002005	0.003352	0.0026438	4	gene_Cg_evm.model.chr7A.197/gene_Cg_evm.model.chr7B.273/gene_Cg_evm.model.chr3A.515/gene_Cg_evm.model.chr24B.120	gene_Car0015970/gene_Car0027570/gene_Car0118110/gene_Car0347410
GO:0030170	pyridoxal phosphate binding	8/603	81/29370	0.0002601	0.0041494	0.0032727	8	gene_Cg_evm.model.chr3B.1176/gene_Cg_evm.model.chr8B.578/gene_Cg_evm.model.chr3A.183/gene_Cg_evm.model.chr3A.687/gene_Cg_evm.model.chr6A.824/gene_Cg_evm.model.chr6B.880/gene_Cg_evm.model.chr6B.385/gene_Cg_evm.model.chr12A.502	gene_Car0009870/gene_Car0109010/gene_Car0114850/gene_Car0119820/gene_Car0144610/gene_Car0185610/gene_Car0190640/gene_Car0327350
GO:0003690	double-stranded DNA binding	5/603	29/29370	0.0002833	0.0043233	0.0034099	5	gene_Cg_evm.model.chr4A.771/gene_Cg_evm.model.chr1A.29/gene_Cg_evm.model.chr19B.435/gene_Cg_evm.model.chr20B.710/gene_Cg_evm.model.chr20A.680	gene_Car0053560/gene_Car0078200/gene_Car0215880/gene_Car0254680/gene_Car0294210
GO:0032508	DNA duplex unwinding	4/603	17/29370	0.0003384	0.0048194	0.0038011	4	gene_Cg_evm.model.chr22B.115/gene_Cg_evm.model.chr6A.236/gene_Cg_evm.model.chr24B.493/gene_Cg_evm.model.chr24A.451	gene_Car0088780/gene_Car0150890/gene_Car0351180/gene_Car0386090
GO:0003735	structural constituent of ribosome	14/603	232/29370	0.0003433	0.0048194	0.0038011	14	gene_Cg_evm.model.chr3B.799/gene_Cg_evm.model.chr7A.220/gene_Cg_evm.model.chr9B.417/gene_Cg_evm.model.chr8B.702/gene_Cg_evm.model.chr16B.836/gene_Cg_evm.model.chr2A.753/gene_Cg_evm.model.chr14B.663/gene_Cg_evm.model.chr19B.657/gene_Cg_evm.model.chr17B.504/gene_Cg_evm.model.chr21B.460/gene_Cg_evm.model.chr21B.334/gene_Cg_evm.model.chr12B.179/gene_Cg_evm.model.chr24B.390/gene_Cg_evm.model.chr24A.349	gene_Car0006330/gene_Car0016220/gene_Car0099590/gene_Car0110340/gene_Car0135970/gene_Car0154490/gene_Car0196170/gene_Car0218150/gene_Car0288140/gene_Car0304630/gene_Car0305990/gene_Car0337650/gene_Car0350100/gene_Car0385100
GO:0140664	ATP-dependent DNA damage sensor activity	4/603	18/29370	0.0004281	0.0057793	0.0045583	4	gene_Cg_evm.model.chr20B.710/gene_Cg_evm.model.chr20A.680/gene_Cg_evm.model.chr12A.444/gene_Cg_evm.model.chr12B.345	gene_Car0254680/gene_Car0294210/gene_Car0327870/gene_Car0336050
GO:0009408	response to heat	3/603	10/29370	0.0009279	0.0112308	0.008858	3	gene_Cg_evm.model.chr1B.367/gene_Cg_evm.model.chr1A.622/gene_Cg_evm.model.chr22B.859	gene_Car0059390/gene_Car0084070/gene_Car0093240
GO:0016209	antioxidant activity	3/603	10/29370	0.0009279	0.0112308	0.008858	3	gene_Cg_evm.model.chr1B.241/gene_Cg_evm.model.chr1A.731/gene_Cg_evm.model.chr13B.292	gene_Car0058120/gene_Car0085160/gene_Car0205890
GO:0051920	peroxiredoxin activity	3/603	10/29370	0.0009279	0.0112308	0.008858	3	gene_Cg_evm.model.chr1B.241/gene_Cg_evm.model.chr1A.731/gene_Cg_evm.model.chr13B.292	gene_Car0058120/gene_Car0085160/gene_Car0205890

GO:0003743	translation initiation factor activity	7/603	78/29370	0.0011139	0.0130322	0.0102788	7	gene_Cg_evm.model.chr3A.706/gene_Cg_evm.model.chr8A.152/gene_Cg_evm.model.chr8A.193/gene_Cg_evm.model.chr20B.251/gene_Cg_evm.model.chr24B.461/gene_Cg_evm.model.chr10A.112/gene_Cg_evm.model.chr10B.111	gene_Car0120010/gene_Car0221780/gene_Car0222200/gene_Car0259180/gene_Car0350840/gene_Car0353670/gene_Car0360770
GO:0016491	oxidoreductase activity	17/603	356/29370	0.0012239	0.0138579	0.0109301	17	gene_Cg_evm.model.chr5A.191/gene_Cg_evm.model.chr4A.749/gene_Cg_evm.model.chr1B.241/gene_Cg_evm.model.chr1A.731/gene_Cg_evm.model.chr8B.457/gene_Cg_evm.model.chr16B.759/gene_Cg_evm.model.chr4B.581/gene_Cg_evm.model.chr6B.758/gene_Cg_evm.model.chr13B.292/gene_Cg_evm.model.chr8A.395/gene_Cg_evm.model.chr8A.514/gene_Cg_evm.model.chr16A.751/gene_Cg_evm.model.chr16A.705/gene_Cg_evm.model.chr20B.309/gene_Cg_evm.model.chr14A.263/gene_Cg_evm.model.chr21B.506/gene_Cg_evm.model.chr21A.313	gene_Car0037940/gene_Car0053360/gene_Car0058120/gene_Car0085160/gene_Car0107810/gene_Car0136760/gene_Car0183280/gene_Car0186750/gene_Car0205890/gene_Car0224150/gene_Car0225380/gene_Car0230730/gene_Car0231210/gene_Car0258450/gene_Car0281410/gene_Car0304120/gene_Car0413560
GO:0009058	biosynthetic process	7/603	82/29370	0.0014943	0.0163906	0.0129276	7	gene_Cg_evm.model.chr4A.749/gene_Cg_evm.model.chr8B.578/gene_Cg_evm.model.chr3A.183/gene_Cg_evm.model.chr4B.581/gene_Cg_evm.model.chr6B.385/gene_Cg_evm.model.chr21B.161/gene_Cg_evm.model.chr12A.502	gene_Car0053360/gene_Car0109010/gene_Car0114850/gene_Car0183280/gene_Car0190640/gene_Car0307840/gene_Car0327350
GO:0031072	heat shock protein binding	3/603	12/29370	0.0016498	0.017548	0.0138405	3	gene_Cg_evm.model.chr1B.367/gene_Cg_evm.model.chr1A.622/gene_Cg_evm.model.chr22B.859	gene_Car0059390/gene_Car0084070/gene_Car0093240
GO:0004518	nuclease activity	4/603	26/29370	0.0018365	0.018959	0.0149534	4	gene_Cg_evm.model.chr4B.780/gene_Cg_evm.model.chr17A.453/gene_Cg_evm.model.chr12A.367/gene_Cg_evm.model.chr24B.178	gene_Car0185290/gene_Car0312570/gene_Car0328610/gene_Car0347980
GO:0016788	hydrolase activity, acting on ester bonds	4/603	28/29370	0.002435	0.0244198	0.0192605	4	gene_Cg_evm.model.chr1A.748/gene_Cg_evm.model.chr17B.739/gene_Cg_evm.model.chr17A.453/gene_Cg_evm.model.chr24B.178	gene_Car0085490/gene_Car0285790/gene_Car0312570/gene_Car0347980
GO:0004298	threonine-type endopeptidase activity	3/603	14/29370	0.0026475	0.0258129	0.0203592	3	gene_Cg_evm.model.chr2A.271/gene_Cg_evm.model.chr23B.836/gene_Cg_evm.model.chr21A.171	gene_Car0159930/gene_Car0261920/gene_Car0415010
GO:0006164	purine nucleotide biosynthetic process	3/603	15/29370	0.0032592	0.0309184	0.0243861	3	gene_Cg_evm.model.chr1B.946/gene_Cg_evm.model.chr8B.457/gene_Cg_evm.model.chr8A.395	gene_Car0065080/gene_Car0107810/gene_Car0224150
GO:0003724	RNA helicase activity	6/603	71/29370	0.0033844	0.0312613	0.0246565	6	gene_Cg_evm.model.chr23B.679/gene_Cg_evm.model.chr10A.65/gene_Cg_evm.model.chr10A.84/gene_Cg_evm.model.chr10A.749/gene_Cg_evm.model.chr23A.578/gene_Cg_evm.model.chr21A.633	gene_Car0263660/gene_Car0353280/gene_Car0353430/gene_Car0359710/gene_Car0375680/gene_Car0410310
GO:0006412	translation	11/603	212/29370	0.0046105	0.0414943	0.0327276	11	gene_Cg_evm.model.chr7A.220/gene_Cg_evm.model.chr9B.417/gene_Cg_evm.model.chr8B.72/gene_Cg_evm.model.chr16B.836/gene_Cg_evm.model.chr14B.663/gene_Cg_evm.model.chr19B.657/gene_Cg_evm.model.chr17B.504/gene_Cg_evm.model.chr21B.460/gene_Cg_evm.model.chr21B.334/gene_Cg_evm.model.chr24B.390/gene_Cg_evm.model.chr24A.349	gene_Car0016220/gene_Car0099590/gene_Car0103680/gene_Car0135970/gene_Car0196170/gene_Car0218150/gene_Car0288140/gene_Car0304630/gene_Car0305990/gene_Car0350100/gene_Car0385100
GO:0005200	structural constituent of cytoskeleton	5/603	54/29370	0.0049561	0.0434894	0.0343011	5	gene_Cg_evm.model.chr5B.400/gene_Cg_evm.model.chr6A.711/gene_Cg_evm.model.chr6B.745/gene_Cg_evm.model.chr17B.215/gene_Cg_evm.model.chr17A.207	gene_Car0069980/gene_Car0145860/gene_Car0186860/gene_Car0290940/gene_Car0315160
GO:0005741	mitochondrial outer membrane	4/603	35/29370	0.0055617	0.0466661	0.0368067	4	gene_Cg_evm.model.chr8B.103/gene_Cg_evm.model.chr8B.889/gene_Cg_evm.model.chr19B.578/gene_Cg_evm.model.chr12A.406	gene_Car0103990/gene_Car0112380/gene_Car0217280/gene_Car0328260
GO:0019773	proteasome core complex, alpha-subunit complex	3/603	18/29370	0.005584	0.0466661	0.0368067	3	gene_Cg_evm.model.chr19B.305/gene_Cg_evm.model.chr17A.583/gene_Cg_evm.model.chr25B.164	gene_Car0214550/gene_Car0311190/gene_Car0407620
GO:0003678	DNA helicase activity	5/603	56/29370	0.0057897	0.0472603	0.0372753	5	gene_Cg_evm.model.chr6A.236/gene_Cg_evm.model.chr18A.119/gene_Cg_evm.model.chr12A.179/gene_Cg_evm.model.chr24B.493/gene_Cg_evm.model.chr24A.451	gene_Car0150890/gene_Car0168630/gene_Car0330430/gene_Car0351180/gene_Car0386090
GO:0016274	protein-arginine N-methyltransferase activity	3/603	19/29370	0.0065309	0.049834	0.0393053	3	gene_Cg_evm.model.chr7A.247/gene_Cg_evm.model.chr3A.525/gene_Cg_evm.model.chr25A.216	gene_Car0016490/gene_Car0118200/gene_Car0420560
GO:0018216	peptidyl-arginine methylation	3/603	19/29370	0.0065309	0.049834	0.0393053	3	gene_Cg_evm.model.chr7A.247/gene_Cg_evm.model.chr3A.525/gene_Cg_evm.model.chr25A.216	gene_Car0016490/gene_Car0118200/gene_Car0420560
GO:0030544	Hsp70 protein binding	3/603	19/29370	0.0065309	0.049834	0.0393053	3	gene_Cg_evm.model.chr1B.367/gene_Cg_evm.model.chr1A.622/gene_Cg_evm.model.chr9B.636	gene_Car0059390/gene_Car0084070/gene_Car0097440
GO:0000278	mitotic cell cycle	3/603	20/29370	0.0075677	0.0553387	0.043647	3	gene_Cg_evm.model.chr6B.886/gene_Cg_evm.model.chr18B.262/gene_Cg_evm.model.chr21B.429	gene_Car0185560/gene_Car0274900/gene_Car0304940

GO:0019843	rRNA binding	3/603	20/29370	0.0075677	0.0553387	0.043647	3	gene_Cg_evm.model.chr3B.1300/gene_Cg_evm.model.chr5B.254/gene_Cg_evm.model.chr2A.753	gene_Car0010860/gene_Car0068530/gene_Car0154490
GO:0046982	protein heterodimerization activity	7/603	111/29370	0.008052	0.0576783	0.0454923	7	gene_Cg_evm.model.chr7B.1153/gene_Cg_evm.model.chr5A.449/gene_Cg_evm.model.chr5B.330/gene_Cg_evm.model.chr3A.752/gene_Cg_evm.model.chr10A.127/gene_Cg_evm.model.chr25B.707/gene_Cg_evm.model.chr25A.532	gene_Car0035770/gene_Car0040700/gene_Car0069320/gene_Car0120400/gene_Car0353810/gene_Car0402180/gene_Car0417330
GO:0016887	ATP hydrolysis activity	14/603	333/29370	0.0094804	0.0665527	0.0524917	14	gene_Cg_evm.model.chr5A.149/gene_Cg_evm.model.chr1B.940/gene_Cg_evm.model.chr5B.1004/gene_Cg_evm.model.chr1A.190/gene_Cg_evm.model.chr1A.237/gene_Cg_evm.model.chr6A.475/gene_Cg_evm.model.chr9A.69/gene_Cg_evm.model.chr13B.393/gene_Cg_evm.model.chr13A.470/gene_Cg_evm.model.chr23B.33/gene_Cg_evm.model.chr20A.777/gene_Cg_evm.model.chr20A.152/gene_Cg_evm.model.chr12A.444/gene_Cg_evm.model.chr24A.256	gene_Car0037540/gene_Car0065030/gene_Car0076170/gene_Car0080210/gene_Car0080670/gene_Car0148350/gene_Car0176620/gene_Car0206910/gene_Car0241240/gene_Car0269590/gene_Car0293160/gene_Car0299360/gene_Car0327870/gene_Car0384170
GO:0008233	peptidase activity	4/603	41/29370	0.0097688	0.0672322	0.0530276	4	gene_Cg_evm.model.chr7A.1005/gene_Cg_evm.model.chr17A.413/gene_Cg_evm.model.chr12B.400/gene_Cg_evm.model.chr11B.517	gene_Car0023790/gene_Car0312970/gene_Car0335500/gene_Car0399960
GO:0006281	DNA repair	8/603	152/29370	0.0134887	0.0910488	0.0718124	8	gene_Cg_evm.model.chr5B.894/gene_Cg_evm.model.chr18A.381/gene_Cg_evm.model.chr19B.144/gene_Cg_evm.model.chr20B.710/gene_Cg_evm.model.chr20A.680/gene_Cg_evm.model.chr12A.367/gene_Cg_evm.model.chr24B.504/gene_Cg_evm.model.chr24A.493	gene_Car0075030/gene_Car0165910/gene_Car0213080/gene_Car0254680/gene_Car0294210/gene_Car0328610/gene_Car0351270/gene_Car0386580
GO:0005021	vascular endothelial growth factor receptor activity	2/603	10/29370	0.0169783	0.1010066	0.0796663	2	gene_Cg_evm.model.chr2B.247/gene_Cg_evm.model.chr2A.210	gene_Car0131760/gene_Car0160570
GO:0005315	inorganic phosphate transmembrane transporter activity	2/603	10/29370	0.0169783	0.1010066	0.0796663	2	gene_Cg_evm.model.chr8B.368/gene_Cg_evm.model.chr10A.579	gene_Car0106830/gene_Car0357980
GO:0009331	glycerol-3-phosphate dehydrogenase complex	2/603	10/29370	0.0169783	0.1010066	0.0796663	2	gene_Cg_evm.model.chr16B.759/gene_Cg_evm.model.chr16A.705	gene_Car0136760/gene_Car0231210
GO:0015075	monoatomic ion transmembrane transporter activity	2/603	10/29370	0.0169783	0.1010066	0.0796663	2	gene_Cg_evm.model.chr13B.291/gene_Cg_evm.model.chr12B.174	gene_Car0205880/gene_Car0337700
GO:0016272	prefoldin complex	2/603	10/29370	0.0169783	0.1010066	0.0796663	2	gene_Cg_evm.model.chr14A.511/gene_Cg_evm.model.chr21A.505	gene_Car0279080/gene_Car0411580
GO:0030507	spectrin binding	2/603	10/29370	0.0169783	0.1010066	0.0796663	2	gene_Cg_evm.model.chr3A.798/gene_Cg_evm.model.chr2A.176	gene_Car0120860/gene_Car0160920
GO:0031175	neuron projection development	2/603	10/29370	0.0169783	0.1010066	0.0796663	2	gene_Cg_evm.model.chr3A.798/gene_Cg_evm.model.chr2A.176	gene_Car0120860/gene_Car0160920
GO:0006364	rRNA processing	4/603	49/29370	0.0179865	0.1052209	0.0829903	4	gene_Cg_evm.model.chr3B.1300/gene_Cg_evm.model.chr5B.254/gene_Cg_evm.model.chr9B.852/gene_Cg_evm.model.chr16B.807	gene_Car0010860/gene_Car0068530/gene_Car0095410/gene_Car0136270
GO:0001525	angiogenesis	2/603	11/29370	0.0204721	0.1072491	0.08459	2	gene_Cg_evm.model.chr2B.247/gene_Cg_evm.model.chr2A.210	gene_Car0131760/gene_Car0160570
GO:0004364	glutathione transferase activity	2/603	11/29370	0.0204721	0.1072491	0.08459	2	gene_Cg_evm.model.chr13B.425/gene_Cg_evm.model.chr8A.420	gene_Car0207230/gene_Car0224430
GO:0005750	mitochondrial respiratory chain complex III	2/603	11/29370	0.0204721	0.1072491	0.08459	2	gene_Cg_evm.model.chr6B.80/gene_Cg_evm.model.chr21A.133	gene_Car0193770/gene_Car0415360
GO:0005758	mitochondrial intermembrane space	2/603	11/29370	0.0204721	0.1072491	0.08459	2	gene_Cg_evm.model.chr24B.272/gene_Cg_evm.model.chr24A.240	gene_Car0348900/gene_Car0384030
GO:0006122	mitochondrial electron transport, ubiquinol to cytochrome c	2/603	11/29370	0.0204721	0.1072491	0.08459	2	gene_Cg_evm.model.chr6B.80/gene_Cg_evm.model.chr21A.133	gene_Car0193770/gene_Car0415360
GO:0006298	mismatch repair	2/603	11/29370	0.0204721	0.1072491	0.08459	2	gene_Cg_evm.model.chr12A.444/gene_Cg_evm.model.chr12B.345	gene_Car0327870/gene_Car0336050
GO:0030983	mismatched DNA binding	2/603	11/29370	0.0204721	0.1072491	0.08459	2	gene_Cg_evm.model.chr12A.444/gene_Cg_evm.model.chr12B.345	gene_Car0327870/gene_Car0336050
GO:0015986	proton motive force-driven ATP synthesis	3/603	30/29370	0.0231763	0.118153	0.0931901	3	gene_Cg_evm.model.chr3B.644/gene_Cg_evm.model.chr5A.455/gene_Cg_evm.model.chr4A.480	gene_Car0004960/gene_Car0040790/gene_Car0050870

GO:0020037	heme binding	8/603	169/29370	0.0238677	0.118153	0.0931901	8	gene_Cg_evm.model.chr3B.1284/gene_Cg_evm.model.chr3B.1287/gene_Cg_evm.model.chr3A.773/gene_Cg_evm.model.chr3A.774/gene_Cg_evm.model.chr2B.926/gene_Cg_evm.model.chr12A.417/gene_Cg_evm.model.chr12B.438/gene_Cg_evm.model.chr25B.182	gene_Car0010720/gene_Car0010750/gene_Car0120620/gene_Car0120630/gene_Car0124680/gene_Car0328130/gene_Car0335110/gene_Car0407390
GO:0000775	chromosome, centromeric region	2/603	12/29370	0.0242365	0.118153	0.0931901	2	gene_Cg_evm.model.chr19B.482/gene_Cg_evm.model.chr21B.429	gene_Car0216310/gene_Car0304940
GO:0005049	nuclear export signal receptor activity	2/603	12/29370	0.0242365	0.118153	0.0931901	2	gene_Cg_evm.model.chr17A.424/gene_Cg_evm.model.chr10A.555	gene_Car0312860/gene_Car0357740
GO:0006072	glycerol-3-phosphate metabolic process	2/603	12/29370	0.0242365	0.118153	0.0931901	2	gene_Cg_evm.model.chr16B.759/gene_Cg_evm.model.chr16A.705	gene_Car0136760/gene_Car0231210
GO:0008168	methyltransferase activity	6/603	109/29370	0.0250903	0.1206395	0.0951512	6	gene_Cg_evm.model.chr3B.1298/gene_Cg_evm.model.chr22B.679/gene_Cg_evm.model.chr16B.807/gene_Cg_evm.model.chr6A.606/gene_Cg_evm.model.chr23B.256/gene_Cg_evm.model.chr25A.216	gene_Car0010840/gene_Car0091460/gene_Car0136270/gene_Car0146950/gene_Car0267810/gene_Car0420560
GO:0000398	mRNA splicing, via spliceosome	5/603	82/29370	0.0269003	0.126904	0.1000922	5	gene_Cg_evm.model.chr7A.291/gene_Cg_evm.model.chr22B.1049/gene_Cg_evm.model.chr6B.893/gene_Cg_evm.model.chr10A.424/gene_Cg_evm.model.chr11B.210	gene_Car0016960/gene_Car0094060/gene_Car0185480/gene_Car0356590/gene_Car0396770
GO:0006325	chromatin organization	3/603	32/29370	0.0274778	0.126904	0.1000922	3	gene_Cg_evm.model.chr1A.860/gene_Cg_evm.model.chr9A.572/gene_Cg_evm.model.chr9A.110	gene_Car0086380/gene_Car0171530/gene_Car0176240
GO:0016620	oxidoreductase activity, acting on the aldehyde or oxo group of donors, NAD or NADP as acceptor	3/603	32/29370	0.0274778	0.126904	0.1000922	3	gene_Cg_evm.model.chr4A.749/gene_Cg_evm.model.chr4B.581/gene_Cg_evm.model.chr8A.514	gene_Car0053360/gene_Car0183280/gene_Car0225380
GO:0000077	DNA damage checkpoint signaling	2/603	13/29370	0.0282591	0.127166	0.1002989	2	gene_Cg_evm.model.chr21B.412/gene_Cg_evm.model.chr15B.426	gene_Car0305140/gene_Car0321060
GO:0002161	aminoacyl-tRNA editing activity	2/603	13/29370	0.0282591	0.127166	0.1002989	2	gene_Cg_evm.model.chr16B.376/gene_Cg_evm.model.chr21A.666	gene_Car0140670/gene_Car0409980
GO:0000287	magnesium ion binding	7/603	147/29370	0.0324765	0.1442944	0.1138085	7	gene_Cg_evm.model.chr5B.154/gene_Cg_evm.model.chr6B.602/gene_Cg_evm.model.chr13B.505/gene_Cg_evm.model.chr19B.707/gene_Cg_evm.model.chr18B.262/gene_Cg_evm.model.chr15B.719/gene_Cg_evm.model.chr24A.256	gene_Car0067470/gene_Car0188350/gene_Car0208040/gene_Car0218760/gene_Car0274900/gene_Car0323930/gene_Car0384170
GO:0009395	phospholipid catabolic process	2/603	15/29370	0.0370306	0.1604658	0.1265632	2	gene_Cg_evm.model.chr5B.1130/gene_Cg_evm.model.chr17B.523	gene_Car0077480/gene_Car0287970
GO:0016818	hydrolase activity, acting on acid anhydrides, in phosphorus-containing anhydrides	2/603	15/29370	0.0370306	0.1604658	0.1265632	2	gene_Cg_evm.model.chr18A.381/gene_Cg_evm.model.chr18A.119	gene_Car0165910/gene_Car0168630
GO:0005739	mitochondrion	5/603	90/29370	0.0380118	0.1627089	0.1283324	5	gene_Cg_evm.model.chr3B.799/gene_Cg_evm.model.chr3B.1314/gene_Cg_evm.model.chr23B.620/gene_Cg_evm.model.chr17B.654/gene_Cg_evm.model.chr10A.124	gene_Car0006330/gene_Car0011020/gene_Car0264250/gene_Car0286720/gene_Car0353780
GO:0004000	adenosine deaminase activity	2/603	16/29370	0.0417563	0.1704239	0.1344174	2	gene_Cg_evm.model.chr22B.615/gene_Cg_evm.model.chr23B.448	gene_Car0090930/gene_Car0265910
GO:0015934	large ribosomal subunit	2/603	16/29370	0.0417563	0.1704239	0.1344174	2	gene_Cg_evm.model.chr9B.417/gene_Cg_evm.model.chr16B.836	gene_Car0099590/gene_Car0135970
GO:0042393	histone binding	2/603	16/29370	0.0417563	0.1704239	0.1344174	2	gene_Cg_evm.model.chr1A.286/gene_Cg_evm.model.chr9A.572	gene_Car0079510/gene_Car0171530
GO:0044237	cellular metabolic process	2/603	16/29370	0.0417563	0.1704239	0.1344174	2	gene_Cg_evm.model.chr5B.944/gene_Cg_evm.model.chr18A.381	gene_Car0075580/gene_Car0165910
GO:0008299	isoprenoid biosynthetic process	2/603	17/29370	0.0466938	0.1862447	0.1468956	2	gene_Cg_evm.model.chr13B.561/gene_Cg_evm.model.chr10A.739	gene_Car0208630/gene_Car0359610
GO:0030150	protein import into mitochondrial matrix	2/603	17/29370	0.0466938	0.1862447	0.1468956	2	gene_Cg_evm.model.chr22B.249/gene_Cg_evm.model.chr19B.578	gene_Car0088370/gene_Car0217280

Supplementary Table 11-2. GO enrichment of DEGs in cluster 2 of head-kidney.

ID	Description	Gene Ratio	BgRatio	pvalue	p.adjust	qvalue	Count	C.gibelio_homolog_id	C.cuvieri_homolog_id
GO:0003779	actin binding	7/198	230/29370	0.0009911	0.084294	0.0782917	7	gene_Cg_evm.model.chr4A.795/gene_Cg_evm.model.chr19B.79/gene_Cg_evm.model.chr19A.88/gene_Cg_evm.model.chr12A.231/gene_Cg_evm.model.chr12B.246/gene_Cg_evm.model.chr15A.430/gene_Cg_evm.model.chr11A.341	gene_Car0053760/gene_Car0212280/gene_Car0245980/gene_Car0329910/gene_Car0336970/gene_Car0343700/gene_Car0390980
GO:0005856	cytoskeleton	6/198	171/29370	0.0011019	0.084294	0.0782917	6	gene_Cg_evm.model.chr16B.486/gene_Cg_evm.model.chr2A.810/gene_Cg_evm.model.chr19B.79/gene_Cg_evm.model.chr19A.88/gene_Cg_evm.model.chr21B.19/gene_Cg_evm.model.chr17A.683	gene_Car0139480/gene_Car0153890/gene_Car0212280/gene_Car0245980/gene_Car0309110/gene_Car0310150
GO:0030866	cortical actin cytoskeleton organization	2/198	11/29370	0.0023895	0.1194935	0.1109847	2	gene_Cg_evm.model.chr19B.79/gene_Cg_evm.model.chr19A.88	gene_Car0212280/gene_Car0245980
GO:0005085	guanyl-nucleotide exchange factor activity	8/198	372/29370	0.0038988	0.1194935	0.1109847	8	gene_Cg_evm.model.chr7B.161/gene_Cg_evm.model.chr3A.66/gene_Cg_evm.model.chr19A.839/gene_Cg_evm.model.chr15B.53/gene_Cg_evm.model.chr12A.447/gene_Cg_evm.model.chr12B.469/gene_Cg_evm.model.chr15A.47/gene_Cg_evm.model.chr10A.73	gene_Car0026190/gene_Car0113590/gene_Car0252780/gene_Car0317550/gene_Car0327840/gene_Car0334790/gene_Car0339940/gene_Car0353340
GO:0005890	sodium:potassium-exchanging ATPase complex	2/198	15/29370	0.0044816	0.1194935	0.1109847	2	gene_Cg_evm.model.chr1A.184/gene_Cg_evm.model.chr23A.723	gene_Car0079900/gene_Car0374320
GO:0032012	regulation of ARF protein signal transduction	3/198	50/29370	0.004686	0.1194935	0.1109847	3	gene_Cg_evm.model.chr3A.66/gene_Cg_evm.model.chr12A.447/gene_Cg_evm.model.chr12B.469	gene_Car0113590/gene_Car0327840/gene_Car0334790
GO:0045892	negative regulation of DNA-templated transcription	3/198	60/29370	0.0077893	0.1631309	0.1515148	3	gene_Cg_evm.model.chr20A.797/gene_Cg_evm.model.chr11A.408/gene_Cg_evm.model.chr11B.476	gene_Car0293060/gene_Car0391700/gene_Car0399580
GO:0007264	small GTPase mediated signal transduction	5/198	185/29370	0.0085297	0.1631309	0.1515148	5	gene_Cg_evm.model.chr1A.45/gene_Cg_evm.model.chr19A.839/gene_Cg_evm.model.chr15B.53/gene_Cg_evm.model.chr15A.47/gene_Cg_evm.model.chr10A.73	gene_Car0078330/gene_Car0252780/gene_Car0317550/gene_Car0339940/gene_Car0353340
GO:0004896	cytokine receptor activity	2/198	25/29370	0.0122513	0.1899156	0.1763922	2	gene_Cg_evm.model.chr16A.366/gene_Cg_evm.model.chr21A.383	gene_Car0234420/gene_Car0412880
GO:0004713	protein tyrosine kinase activity	5/198	204/29370	0.0126299	0.1899156	0.1763922	5	gene_Cg_evm.model.chr18A.36/gene_Cg_evm.model.chr14B.575/gene_Cg_evm.model.chr17B.425/gene_Cg_evm.model.chr17A.683/gene_Cg_evm.model.chr23A.737	gene_Car0169430/gene_Car0197100/gene_Car0288900/gene_Car0310150/gene_Car0374180
GO:0030837	negative regulation of actin filament polymerization	2/198	27/29370	0.0142083	0.1899156	0.1763922	2	gene_Cg_evm.model.chr3A.769/gene_Cg_evm.model.chr15A.430	gene_Car0120570/gene_Car0343700
GO:0006814	sodium ion transport	3/198	78/29370	0.0158584	0.1899156	0.1763922	3	gene_Cg_evm.model.chr1A.184/gene_Cg_evm.model.chr12A.633/gene_Cg_evm.model.chr23A.723	gene_Car0079900/gene_Car0326010/gene_Car0374320
GO:0000122	negative regulation of transcription by RNA polymerase II	2/198	30/29370	0.0173779	0.1899156	0.1763922	2	gene_Cg_evm.model.chr11A.408/gene_Cg_evm.model.chr11B.476	gene_Car0391700/gene_Car0399580
GO:0016780	phosphotransferase activity, for other substituted phosphate groups	2/198	30/29370	0.0173779	0.1899156	0.1763922	2	gene_Cg_evm.model.chr12A.608/gene_Cg_evm.model.chr12B.603	gene_Car0326270/gene_Car0333490
GO:0006357	regulation of transcription by RNA polymerase II	6/198	319/29370	0.021519	0.2194936	0.203864	6	gene_Cg_evm.model.chr3A.1017/gene_Cg_evm.model.chr16B.367/gene_Cg_evm.model.chr23B.824/gene_Cg_evm.model.chr20A.685/gene_Cg_evm.model.chr10B.11/gene_Cg_evm.model.chr11B.125	gene_Car0122960/gene_Car0140760/gene_Car0261810/gene_Car0294170/gene_Car0360000/gene_Car0396040
GO:0007010	cytoskeleton organization	2/198	36/29370	0.0245144	0.2344188	0.2177265	2	gene_Cg_evm.model.chr12A.231/gene_Cg_evm.model.chr12B.246	gene_Car0329910/gene_Car0336970
GO:0008092	cytoskeletal protein binding	2/198	39/29370	0.0284577	0.2561191	0.2378815	2	gene_Cg_evm.model.chr19B.79/gene_Cg_evm.model.chr19A.88	gene_Car0212280/gene_Car0245980
GO:0003729	mRNA binding	2/198	49/29370	0.0432375	0.3078622	0.2859401	2	gene_Cg_evm.model.chr17B.225/gene_Cg_evm.model.chr12B.334	gene_Car0290840/gene_Car0336160
GO:0043565	sequence-specific DNA binding	6/198	400/29370	0.0545766	0.3078622	0.2859401	6	gene_Cg_evm.model.chr5A.833/gene_Cg_evm.model.chr16B.367/gene_Cg_evm.model.chr16A.493/gene_Cg_evm.model.chr12B.535/gene_Cg_evm.model.chr10B.11/gene_Cg_evm.model.chr11B.125	gene_Car0044570/gene_Car0140760/gene_Car0233240/gene_Car0334190/gene_Car0360000/gene_Car0396040

GO:0046983	protein dimerization activity	6/198	403/29370	0.0561853	0.3078622	0.2859401	6	gene_Cg_evm.model.chr23B.396/gene_Cg_evm.model.chr12B.327/gene_Cg_evm.model.chr11A.189/gene_Cg_evm.model.chr11A.376/gene_Cg_evm.model.chr11A.408/gene_Cg_evm.model.chr11B.476	gene_Car0266440/gene_Car0336210/gene_Car0389450/gene_Car0391360/gene_Car0391700/gene_Car0399580
GO:0015075	monoatomic ion transmembrane transporter activity	1/198	10/29370	0.0654166	0.3078622	0.2859401	1	gene_Cg_evm.model.chr13A.337	gene_Car0239880
GO:0016012	sarcoglycan complex	1/198	10/29370	0.0654166	0.3078622	0.2859401	1	gene_Cg_evm.model.chr19B.360	gene_Car0215060
GO:0019786	protein-phosphatidylethanolamide deconjugating activity	1/198	10/29370	0.0654166	0.3078622	0.2859401	1	gene_Cg_evm.model.chr3B.1505	gene_Car0012710
GO:0023051	regulation of signaling	1/198	10/29370	0.0654166	0.3078622	0.2859401	1	gene_Cg_evm.model.chr10B.386	gene_Car0363400
GO:0140911	pore-forming activity	1/198	10/29370	0.0654166	0.3078622	0.2859401	1	gene_Cg_evm.model.chr3B.932	gene_Car0007570
GO:0030036	actin cytoskeleton organization	2/198	62/29370	0.0657206	0.3078622	0.2859401	2	gene_Cg_evm.model.chr23B.323/gene_Cg_evm.model.chr11A.84	gene_Car0267140/gene_Car0388450
GO:0005178	integrin binding	1/198	11/29370	0.0717193	0.3078622	0.2859401	1	gene_Cg_evm.model.chr3A.762	gene_Car0120500
GO:0008028	monocarboxylic acid transmembrane transporter activity	1/198	11/29370	0.0717193	0.3078622	0.2859401	1	gene_Cg_evm.model.chr8A.375	gene_Car0223960
GO:0015718	monocarboxylic acid transport	1/198	11/29370	0.0717193	0.3078622	0.2859401	1	gene_Cg_evm.model.chr8A.375	gene_Car0223960
GO:0048384	retinoic acid receptor signaling pathway	1/198	11/29370	0.0717193	0.3078622	0.2859401	1	gene_Cg_evm.model.chr12B.535	gene_Car0334190
GO:0042981	regulation of apoptotic process	3/198	143/29370	0.0727357	0.3078622	0.2859401	3	gene_Cg_evm.model.chr16B.591/gene_Cg_evm.model.chr16A.556/gene_Cg_evm.model.chr23B.663	gene_Car0138410/gene_Car0232730/gene_Car0263830
GO:0043547	positive regulation of GTPase activity	2/198	66/29370	0.0732745	0.3078622	0.2859401	2	gene_Cg_evm.model.chr7B.161/gene_Cg_evm.model.chr12A.423	gene_Car0026190/gene_Car0328070
GO:0008013	beta-catenin binding	1/198	12/29370	0.0779797	0.3078622	0.2859401	1	gene_Cg_evm.model.chr1B.318	gene_Car0058900
GO:0008349	MAP kinase kinase kinase kinase activity	1/198	12/29370	0.0779797	0.3078622	0.2859401	1	gene_Cg_evm.model.chr15B.809	gene_Car0324830
GO:0015630	microtubule cytoskeleton	1/198	12/29370	0.0779797	0.3078622	0.2859401	1	gene_Cg_evm.model.chr19B.688	gene_Car0218490
GO:0042910	xenobiotic transmembrane transporter activity	1/198	12/29370	0.0779797	0.3078622	0.2859401	1	gene_Cg_evm.model.chr17B.471	gene_Car0288420
GO:0001784	phosphotyrosine residue binding	1/198	13/29370	0.0841981	0.3078622	0.2859401	1	gene_Cg_evm.model.chr10B.386	gene_Car0363400
GO:0004861	cyclin-dependent protein serine/threonine kinase inhibitor activity	1/198	13/29370	0.0841981	0.3078622	0.2859401	1	gene_Cg_evm.model.chr25A.11	gene_Car0422390
GO:0005523	tropomyosin binding	1/198	13/29370	0.0841981	0.3078622	0.2859401	1	gene_Cg_evm.model.chr11A.341	gene_Car0390980
GO:0008061	chitin binding	1/198	13/29370	0.0841981	0.3078622	0.2859401	1	gene_Cg_evm.model.chr11A.360	gene_Car0391170
GO:0051694	pointed-end actin filament capping	1/198	13/29370	0.0841981	0.3078622	0.2859401	1	gene_Cg_evm.model.chr11A.341	gene_Car0390980
GO:0004879	nuclear receptor activity	2/198	72/29370	0.085093	0.3078622	0.2859401	2	gene_Cg_evm.model.chr16A.493/gene_Cg_evm.model.chr12B.535	gene_Car0233240/gene_Car0334190
GO:0004683	calmodulin-dependent protein kinase activity	1/198	14/29370	0.0903748	0.3078622	0.2859401	1	gene_Cg_evm.model.chr7B.918	gene_Car0033840
GO:0038036	sphingosine-1-phosphate receptor activity	1/198	14/29370	0.0903748	0.3078622	0.2859401	1	gene_Cg_evm.model.chr22B.128	gene_Car0088720

GO:0045893	positive regulation of DNA-templated transcription	2/198	76/29370	0.0932691	0.3078622	0.2859401	2	gene_Cg_evm.model.chr12A.257/gene_Cg_evm.model.chr11A.376	gene_Car0329660/gene_Car0391360
GO:0005516	calmodulin binding	2/198	77/29370	0.0953477	0.3078622	0.2859401	2	gene_Cg_evm.model.chr7B.918/gene_Cg_evm.model.chr4A.795	gene_Car0033840/gene_Car0053760
GO:0016758	hexosyltransferase activity	2/198	77/29370	0.0953477	0.3078622	0.2859401	2	gene_Cg_evm.model.chr22B.887/gene_Cg_evm.model.chr21B.510	gene_Car0093590/gene_Car0304080
GO:0022857	transmembrane transporter activity	5/198	361/29370	0.0982152	0.3078622	0.2859401	5	gene_Cg_evm.model.chr7A.406/gene_Cg_evm.model.chr8A.375/gene_Cg_evm.model.chr17B.471/gene_Cg_evm.model.chr10B.423/gene_Cg_evm.model.chr11A.141	gene_Car0018070/gene_Car0223960/gene_Car0288420/gene_Car0363800/gene_Car0388950
GO:0005579	membrane attack complex	1/198	16/29370	0.1026041	0.3078622	0.2859401	1	gene_Cg_evm.model.chr3B.932	gene_Car0007570
GO:0005925	focal adhesion	1/198	16/29370	0.1026041	0.3078622	0.2859401	1	gene_Cg_evm.model.chr17A.683	gene_Car0310150
GO:0035556	intracellular signal transduction	5/198	367/29370	0.1034478	0.3078622	0.2859401	5	gene_Cg_evm.model.chr3B.827/gene_Cg_evm.model.chr14B.575/gene_Cg_evm.model.chr13B.4/gene_Cg_evm.model.chr19B.787/gene_Cg_evm.model.chr19A.839	gene_Car0006590/gene_Car0197100/gene_Car0203140/gene_Car0219440/gene_Car0252780
GO:0001609	G protein-coupled adenosine receptor activity	1/198	17/29370	0.1086572	0.3078622	0.2859401	1	gene_Cg_evm.model.chr8A.333	gene_Car0223490
GO:0001973	G protein-coupled adenosine receptor signaling pathway	1/198	17/29370	0.1086572	0.3078622	0.2859401	1	gene_Cg_evm.model.chr8A.333	gene_Car0223490
GO:0005248	voltage-gated sodium channel activity	1/198	17/29370	0.1086572	0.3078622	0.2859401	1	gene_Cg_evm.model.chr12A.633	gene_Car0326010
GO:0000978	RNA polymerase II cis-regulatory region sequence-specific DNA binding	2/198	88/29370	0.1190288	0.3193848	0.2966423	2	gene_Cg_evm.model.chr12A.257/gene_Cg_evm.model.chr11A.376	gene_Car0329660/gene_Car0391360
GO:0005096	GTPase activator activity	3/198	178/29370	0.119391	0.3193848	0.2966423	3	gene_Cg_evm.model.chr3B.843/gene_Cg_evm.model.chr7B.161/gene_Cg_evm.model.chr12A.423	gene_Car0006750/gene_Car0026190/gene_Car0328070
GO:0005548	phospholipid transporter activity	1/198	19/29370	0.120642	0.3193848	0.2966423	1	gene_Cg_evm.model.chr10B.103	gene_Car0360680
GO:0005198	structural molecule activity	3/198	182/29370	0.1252973	0.3193848	0.2966423	3	gene_Cg_evm.model.chr19B.79/gene_Cg_evm.model.chr19A.88/gene_Cg_evm.model.chr10B.497	gene_Car0212280/gene_Car0245980/gene_Car0364560
GO:0007188	adenylate cyclase-modulating G protein-coupled receptor signaling pathway	1/198	20/29370	0.1265741	0.3193848	0.2966423	1	gene_Cg_evm.model.chr11A.200	gene_Car0389550
GO:0006865	amino acid transport	1/198	21/29370	0.1324663	0.3193848	0.2966423	1	gene_Cg_evm.model.chr7A.406	gene_Car0018070
GO:0016791	phosphatase activity	4/198	293/29370	0.1368997	0.3193848	0.2966423	4	gene_Cg_evm.model.chr4A.441/gene_Cg_evm.model.chr8A.242/gene_Cg_evm.model.chr14A.457/gene_Cg_evm.model.chr15A.430	gene_Car0050270/gene_Car0222480/gene_Car0279620/gene_Car0343700
GO:0005247	voltage-gated chloride channel activity	1/198	22/29370	0.1383191	0.3193848	0.2966423	1	gene_Cg_evm.model.chr7A.378	gene_Car0017810
GO:0006807	nitrogen compound metabolic process	1/198	22/29370	0.1383191	0.3193848	0.2966423	1	gene_Cg_evm.model.chr2A.654	gene_Car0155890
GO:0070403	NAD+ binding	1/198	22/29370	0.1383191	0.3193848	0.2966423	1	gene_Cg_evm.model.chr9A.503	gene_Car0172230
GO:0008138	protein tyrosine/serine/threonine phosphatase activity	2/198	99/29370	0.1439765	0.3193848	0.2966423	2	gene_Cg_evm.model.chr8A.242/gene_Cg_evm.model.chr15A.430	gene_Car0222480/gene_Car0343700
GO:0003746	translation elongation factor activity	1/198	23/29370	0.1441325	0.3193848	0.2966423	1	gene_Cg_evm.model.chr20A.752	gene_Car0293370
GO:0045104	intermediate filament cytoskeleton organization	1/198	23/29370	0.1441325	0.3193848	0.2966423	1	gene_Cg_evm.model.chr2A.810	gene_Car0153890
GO:0001518	voltage-gated sodium channel complex	1/198	24/29370	0.1499069	0.3193848	0.2966423	1	gene_Cg_evm.model.chr12A.633	gene_Car0326010

GO:0004697	protein kinase C activity	1/198	24/29370	0.1499069	0.3193848	0.2966423	1	gene_Cg_evm.model.chr13B.547	gene_Car0208470
GO:0008146	sulfotransferase activity	2/198	103/29370	0.1533023	0.3193848	0.2966423	2	gene_Cg_evm.model.chr8B.76/gene_Cg_evm.model.chr14A.659	gene_Car0103720/gene_Car0277630
GO:0004721	phosphoprotein phosphatase activity	1/198	25/29370	0.1556426	0.3193848	0.2966423	1	gene_Cg_evm.model.chr5B.675	gene_Car0072850
GO:0006631	fatty acid metabolic process	1/198	25/29370	0.1556426	0.3193848	0.2966423	1	gene_Cg_evm.model.chr9A.503	gene_Car0172230
GO:0000209	protein polyubiquitination	1/198	26/29370	0.1613397	0.3193848	0.2966423	1	gene_Cg_evm.model.chr1A.813	gene_Car0086130
GO:0005520	insulin-like growth factor binding	1/198	26/29370	0.1613397	0.3193848	0.2966423	1	gene_Cg_evm.model.chr23B.666	gene_Car0263800
GO:0006414	translational elongation	1/198	26/29370	0.1613397	0.3193848	0.2966423	1	gene_Cg_evm.model.chr20A.752	gene_Car0293370
GO:0006897	endocytosis	1/198	26/29370	0.1613397	0.3193848	0.2966423	1	gene_Cg_evm.model.chr3B.843	gene_Car0006750
GO:0030145	manganese ion binding	1/198	26/29370	0.1613397	0.3193848	0.2966423	1	gene_Cg_evm.model.chr5B.675	gene_Car0072850
GO:0000145	exocyst	1/198	27/29370	0.1669986	0.3193848	0.2966423	1	gene_Cg_evm.model.chr25B.301	gene_Car0406230
GO:0003707	nuclear steroid receptor activity	1/198	27/29370	0.1669986	0.3193848	0.2966423	1	gene_Cg_evm.model.chr5A.833	gene_Car0044570
GO:0017017	MAP kinase tyrosine/serine/threonine phosphatase activity	1/198	27/29370	0.1669986	0.3193848	0.2966423	1	gene_Cg_evm.model.chr8A.242	gene_Car0222480
GO:0051015	actin filament binding	2/198	115/29370	0.1819142	0.3436158	0.3191478	2	gene_Cg_evm.model.chr23B.323/gene_Cg_evm.model.chr11A.84	gene_Car0267140/gene_Car0388450
GO:0016311	dephosphorylation	3/198	221/29370	0.1876939	0.3464352	0.3217664	3	gene_Cg_evm.model.chr4A.441/gene_Cg_evm.model.chr8A.242/gene_Cg_evm.model.chr15A.430	gene_Car0050270/gene_Car0222480/gene_Car0343700
GO:0008081	phosphoric diester hydrolase activity	2/198	118/29370	0.1891889	0.3464352	0.3217664	2	gene_Cg_evm.model.chr3B.827/gene_Cg_evm.model.chr2B.885	gene_Car0006590/gene_Car0125140
GO:0006325	chromatin organization	1/198	32/29370	0.1947283	0.3464352	0.3217664	1	gene_Cg_evm.model.chr8B.157	gene_Car0104600
GO:0006334	nucleosome assembly	1/198	32/29370	0.1947283	0.3464352	0.3217664	1	gene_Cg_evm.model.chr24A.491	gene_Car0386560
GO:1990904	ribonucleoprotein complex	1/198	32/29370	0.1947283	0.3464352	0.3217664	1	gene_Cg_evm.model.chr11A.450	gene_Car0392130
GO:0005737	cytoplasm	5/198	468/29370	0.2097095	0.3667187	0.3406056	5	gene_Cg_evm.model.chr7B.46/gene_Cg_evm.model.chr12A.423/gene_Cg_evm.model.chr12B.327/gene_Cg_evm.model.chr11A.408/gene_Cg_evm.model.chr11B.476	gene_Car0024930/gene_Car0328070/gene_Car0336210/gene_Car0391700/gene_Car0399580
GO:0046856	phosphatidylinositol dephosphorylation	1/198	35/29370	0.2109232	0.3667187	0.3406056	1	gene_Cg_evm.model.chr14A.457	gene_Car0279620
GO:0004198	calcium-dependent cysteine-type endopeptidase activity	1/198	37/29370	0.2215394	0.3731128	0.3465444	1	gene_Cg_evm.model.chr13A.721	gene_Car0244080
GO:0006821	chloride transport	1/198	37/29370	0.2215394	0.3731128	0.3465444	1	gene_Cg_evm.model.chr7A.378	gene_Car0017810
GO:0006470	protein dephosphorylation	3/198	242/29370	0.2240687	0.3731128	0.3465444	3	gene_Cg_evm.model.chr4A.441/gene_Cg_evm.model.chr8A.242/gene_Cg_evm.model.chr15A.430	gene_Car0050270/gene_Car0222480/gene_Car0343700
GO:0004435	phosphatidylinositol phospholipase C activity	1/198	38/29370	0.226794	0.3731128	0.3465444	1	gene_Cg_evm.model.chr3B.827	gene_Car0006590
GO:0006887	exocytosis	1/198	38/29370	0.226794	0.3731128	0.3465444	1	gene_Cg_evm.model.chr25B.301	gene_Car0406230
GO:0005261	monoatomic cation channel activity	1/198	39/29370	0.2320134	0.3776388	0.3507481	1	gene_Cg_evm.model.chr12A.633	gene_Car0326010
GO:0005003	ephrin receptor activity	1/198	40/29370	0.2371977	0.3780339	0.3511151	1	gene_Cg_evm.model.chr23A.737	gene_Car0374180

GO:0007178	transmembrane receptor protein serine/threonine kinase signaling pathway	1/198	40/29370	0.2371977	0.3780339	0.3511151	1	gene_Cg_evm.model.chr12A.305	gene_Car0329180
GO:0004675	transmembrane receptor protein serine/threonine kinase activity	1/198	41/29370	0.2423472	0.3783584	0.3514165	1	gene_Cg_evm.model.chr12A.305	gene_Car0329180
GO:0008233	peptidase activity	1/198	41/29370	0.2423472	0.3783584	0.3514165	1	gene_Cg_evm.model.chr12B.395	gene_Car0335550
GO:0043087	regulation of GTPase activity	1/198	42/29370	0.2474621	0.3824415	0.3552088	1	gene_Cg_evm.model.chr2A.339	gene_Car0159130
GO:0000226	microtubule cytoskeleton organization	1/198	43/29370	0.2525427	0.3863903	0.3588765	1	gene_Cg_evm.model.chr19B.688	gene_Car0218490
GO:0015914	phospholipid transport	1/198	45/29370	0.2626016	0.3978025	0.369476	1	gene_Cg_evm.model.chr10B.103	gene_Car0360680
GO:0003713	transcription coactivator activity	1/198	46/29370	0.2675805	0.3986773	0.3702885	1	gene_Cg_evm.model.chr1B.318	gene_Car0058900
GO:0031267	small GTPase binding	2/198	150/29370	0.2683906	0.3986773	0.3702885	2	gene_Cg_evm.model.chr13A.337/gene_Cg_evm.model.chr20A.128	gene_Car0239880/gene_Car0299650
GO:0019001	guanyl nucleotide binding	1/198	49/29370	0.2823173	0.4153321	0.3857574	1	gene_Cg_evm.model.chr11A.200	gene_Car0389550
GO:0031683	G-protein beta/gamma-subunit complex binding	1/198	50/29370	0.2871637	0.4184385	0.3886425	1	gene_Cg_evm.model.chr11A.200	gene_Car0389550
GO:0000139	Golgi membrane	1/198	54/29370	0.3062258	0.4407267	0.4093437	1	gene_Cg_evm.model.chr25B.622	gene_Car0402740
GO:0006486	protein glycosylation	2/198	166/29370	0.3082207	0.4407267	0.4093437	2	gene_Cg_evm.model.chr22B.887/gene_Cg_evm.model.chr21B.510	gene_Car0093590/gene_Car0304080
GO:0004553	hydrolase activity, hydrolyzing O-glycosyl compounds	1/198	57/29370	0.3201888	0.4536007	0.421301	1	gene_Cg_evm.model.chr11A.360	gene_Car0391170
GO:0004114	3',5'-cyclic-nucleotide phosphodiesterase activity	1/198	61/29370	0.3383721	0.4744112	0.4406296	1	gene_Cg_evm.model.chr2B.885	gene_Car0125140
GO:0051726	regulation of cell cycle	1/198	62/29370	0.3428418	0.4744112	0.4406296	1	gene_Cg_evm.model.chr25A.11	gene_Car0422390
GO:0016747	acyltransferase activity, transferring groups other than amino-acyl groups	1/198	63/29370	0.3472814	0.4744112	0.4406296	1	gene_Cg_evm.model.chr5A.54	gene_Car0036460
GO:0030527	structural constituent of chromatin	1/198	63/29370	0.3472814	0.4744112	0.4406296	1	gene_Cg_evm.model.chr24A.491	gene_Car0386560
GO:0046872	metal ion binding	4/198	453/29370	0.3649678	0.4941599	0.458972	4	gene_Cg_evm.model.chr1B.23/gene_Cg_evm.model.chr17B.225/gene_Cg_evm.model.chr12B.334/gene_Cg_evm.model.chr10B.103	gene_Car0055790/gene_Car0290840/gene_Car0336160/gene_Car0360680
GO:0005667	transcription regulator complex	1/198	68/29370	0.369036	0.4952851	0.4600172	1	gene_Cg_evm.model.chr12B.327	gene_Car0336210
GO:0000786	nucleosome	1/198	70/29370	0.3775344	0.4979549	0.4624968	1	gene_Cg_evm.model.chr24A.491	gene_Car0386560
GO:0030215	29emaphoring receptor binding	1/198	70/29370	0.3775344	0.4979549	0.4624968	1	gene_Cg_evm.model.chr5A.185	gene_Car0037910
GO:0005506	iron ion binding	2/198	199/29370	0.3886381	0.5015174	0.4658056	2	gene_Cg_evm.model.chr5B.675/gene_Cg_evm.model.chr6B.695	gene_Car0072850/gene_Car0187370
GO:0006397	mRNA processing	1/198	73/29370	0.3900691	0.5015174	0.4658056	1	gene_Cg_evm.model.chr11A.8	gene_Car0387680
GO:0008009	chemokine activity	1/198	73/29370	0.3900691	0.5015174	0.4658056	1	gene_Cg_evm.model.chr24A.364	gene_Car0385240
GO:0006955	immune response	2/198	208/29370	0.4098984	0.5189411	0.4819887	2	gene_Cg_evm.model.chr3B.932/gene_Cg_evm.model.chr24A.364	gene_Car0007570/gene_Car0385240
GO:0003743	translation initiation factor activity	1/198	78/29370	0.4104044	0.5189411	0.4819887	1	gene_Cg_evm.model.chr11A.286	gene_Car0390420
GO:0140359	ABC-type transporter activity	1/198	83/29370	0.4300651	0.5393439	0.5009386	1	gene_Cg_evm.model.chr18A.601	gene_Car0163840

GO:0016746	acyltransferase activity	1/198	89/29370	0.4527986	0.5632373	0.5231306	1	gene_Cg_evm.model.chr16A.527	gene_Car0233040
GO:0006813	potassium ion transport	2/198	245/29370	0.4931583	0.6018608	0.5590038	2	gene_Cg_evm.model.chr1A.184/gene_Cg_evm.model.chr23A.723	gene_Car0079900/gene_Car0374320
GO:0003774	cytoskeletal motor activity	1/198	101/29370	0.4955942	0.6018608	0.5590038	1	gene_Cg_evm.model.chr19B.137	gene_Car0212910
GO:0005923	bicellular tight junction	1/198	102/29370	0.4990064	0.6018608	0.5590038	1	gene_Cg_evm.model.chr10B.497	gene_Car0364560
GO:0016787	hydrolase activity	2/198	248/29370	0.4995838	0.6018608	0.5590038	2	gene_Cg_evm.model.chr5B.675/gene_Cg_evm.model.chr14B.317	gene_Car0072850/gene_Car0199740
GO:0007169	transmembrane receptor protein tyrosine kinase signaling pathway	1/198	110/29370	0.5254902	0.6270641	0.5824125	1	gene_Cg_evm.model.chr23A.737	gene_Car0374180
GO:0004497	monooxygenase activity	1/198	111/29370	0.5287011	0.6270641	0.5824125	1	gene_Cg_evm.model.chr6B.695	gene_Car0187370
GO:0016459	myosin complex	1/198	118/29370	0.5505806	0.647991	0.6018492	1	gene_Cg_evm.model.chr19B.137	gene_Car0212910
GO:0098609	cell-cell adhesion	1/198	120/29370	0.5566441	0.6501264	0.6038325	1	gene_Cg_evm.model.chr3A.762	gene_Car0120500
GO:0061630	ubiquitin protein ligase activity	1/198	126/29370	0.5743507	0.6638712	0.6165986	1	gene_Cg_evm.model.chr1A.813	gene_Car0086130
GO:0004725	protein tyrosine phosphatase activity	1/198	128/29370	0.5800951	0.6638712	0.6165986	1	gene_Cg_evm.model.chr4A.441	gene_Car0050270
GO:0016705	oxidoreductase activity, acting on paired donors, with incorporation or reduction of molecular oxygen	1/198	129/29370	0.5829383	0.6638712	0.6165986	1	gene_Cg_evm.model.chr6B.695	gene_Car0187370
GO:0006886	intracellular protein transport	2/198	291/29370	0.5857687	0.6638712	0.6165986	2	gene_Cg_evm.model.chr17B.771/gene_Cg_evm.model.chr20A.128	gene_Car0285510/gene_Car0299650
GO:0000981	DNA-binding transcription factor activity, RNA polymerase II-specific	3/198	467/29370	0.6119847	0.6884827	0.6394577	3	gene_Cg_evm.model.chr2A.597/gene_Cg_evm.model.chr11A.357/gene_Cg_evm.model .chr11B.125	gene_Car0156500/gene_Car0391140/gene_Car0396040
GO:0004674	protein serine/threonine kinase activity	2/198	317/29370	0.6323487	0.7050703	0.654864	2	gene_Cg_evm.model.chr13B.547/gene_Cg_evm.model.chr8A.285	gene_Car0208470/gene_Car0222980
GO:0005615	extracellular space	1/198	149/29370	0.6359457	0.7050703	0.654864	1	gene_Cg_evm.model.chr9A.536	gene_Car0171800
GO:0006629	lipid metabolic process	1/198	155/29370	0.6504994	0.7160173	0.6650315	1	gene_Cg_evm.model.chr3B.827	gene_Car0006590
GO:0008289	lipid binding	1/198	164/29370	0.6712514	0.7335819	0.6813454	1	gene_Cg_evm.model.chr9A.536	gene_Car0171800
GO:0007166	cell surface receptor signaling pathway	1/198	168/29370	0.6800765	0.7350957	0.6827515	1	gene_Cg_evm.model.chr10B.386	gene_Car0363400
GO:0020037	heme binding	1/198	169/29370	0.6822457	0.7350957	0.6827515	1	gene_Cg_evm.model.chr6B.695	gene_Car0187370
GO:0000166	nucleotide binding	1/198	188/29370	0.7207888	0.7711936	0.7162789	1	gene_Cg_evm.model.chr7A.503	gene_Car0019070
GO:0008083	growth factor activity	1/198	203/29370	0.747901	0.7946448	0.7380602	1	gene_Cg_evm.model.chr3B.843	gene_Car0006750
GO:0008237	metallopeptidase activity	1/198	223/29370	0.7800183	0.8230537	0.7644462	1	gene_Cg_evm.model.chr12B.395	gene_Car0335550
GO:0004842	ubiquitin-protein transferase activity	1/198	232/29370	0.7931096	0.8311354	0.7719524	1	gene_Cg_evm.model.chr10B.386	gene_Car0363400
GO:0004252	serine-type endopeptidase activity	1/198	239/29370	0.8027533	0.8355188	0.7760236	1	gene_Cg_evm.model.chr12B.437	gene_Car0335120
GO:0005975	carbohydrate metabolic process	1/198	251/29370	0.8182561	0.8458999	0.7856655	1	gene_Cg_evm.model.chr11A.360	gene_Car0391170
GO:0005216	monoatomic ion channel activity	2/198	466/29370	0.8243171	0.8464464	0.7861731	2	gene_Cg_evm.model.chr5B.565/gene_Cg_evm.model.chr12A.633	gene_Car0071730/gene_Car0326010
GO:0005886	plasma membrane	2/198	496/29370	0.8499296	0.8669281	0.8051964	2	gene_Cg_evm.model.chr12A.633/gene_Cg_evm.model.chr23A.737	gene_Car0326010/gene_Car0374180

GO:0016887	ATP hydrolysis activity	1/198	333/29370	0.8962133	0.9080837	0.8434214	1	gene_Cg_evm.model.chr18A.601	gene_Car0163840
GO:0016491	oxidoreductase activity	1/198	356/29370	0.9113313	0.9173269	0.8520064	1	gene_Cg_evm.model.chr1B.377	gene_Car0059480
GO:0007155	cell adhesion	1/198	373/29370	0.9210777	0.9210777	0.8554901	1	gene_Cg_evm.model.chr3A.762	gene_Car0120500

Supplementary Table 11-3. GO enrichment of DEGs in cluster 1 of spleen.

ID	Description	Gene Ratio	BgRatio	pvalue	p.adjust	qvalue	Count	C.gibelio_homolog_id	C.cuvieri_homolog_id
GO:0140662	ATP-dependent protein folding chaperone	6/172	61/29370	1.572E-06	0.0002342	0.0002234	6	gene_Cg_evm.model.chr23B.33/gene_Cg_evm.model.chr20A.777/gene_Cg_evm.model.chr21B.721/gene_Cg_evm.model.chr15A.496/gene_Cg_evm.model.chr10A.445/gene_Cg_evm.model.chr21A.710	gene_Car0269590/gene_Car0293160/gene_Car0301370/gene_Car0344240/gene_Car0356800/gene_Car0409540
GO:0051082	unfolded protein binding	6/172	76/29370	5.756E-06	0.0004288	0.0004089	6	gene_Cg_evm.model.chr1B.128/gene_Cg_evm.model.chr1A.833/gene_Cg_evm.model.chr23B.33/gene_Cg_evm.model.chr20A.777/gene_Cg_evm.model.chr10A.445/gene_Cg_evm.model.chr25A.404	gene_Car0056970/gene_Car0086320/gene_Car0269590/gene_Car0293160/gene_Car0356800/gene_Car0418640
GO:0006457	protein folding	6/172	107/29370	4.097E-05	0.0020347	0.0019406	6	gene_Cg_evm.model.chr1B.128/gene_Cg_evm.model.chr1A.833/gene_Cg_evm.model.chr23B.33/gene_Cg_evm.model.chr20A.777/gene_Cg_evm.model.chr10A.445/gene_Cg_evm.model.chr25A.404	gene_Car0056970/gene_Car0086320/gene_Car0269590/gene_Car0293160/gene_Car0356800/gene_Car0418640
GO:0016887	ATP hydrolysis activity	8/172	333/29370	0.0008106	0.0301933	0.0287962	8	gene_Cg_evm.model.chr5A.374/gene_Cg_evm.model.chr1B.233/gene_Cg_evm.model.chr1A.190/gene_Cg_evm.model.chr3A.932/gene_Cg_evm.model.chr23B.33/gene_Cg_evm.model.chr20A.777/gene_Cg_evm.model.chr10A.445/gene_Cg_evm.model.chr22A.24	gene_Car0039880/gene_Car0058060/gene_Car0080210/gene_Car0122220/gene_Car0269590/gene_Car0293160/gene_Car0356800/gene_Car0368380
GO:0001671	ATPase activator activity	2/172	11/29370	0.0018114	0.0539791	0.0514813	2	gene_Cg_evm.model.chr17B.156/gene_Cg_evm.model.chr17A.147	gene_Car0291520/gene_Car0315710
GO:0052689	carboxylic ester hydrolase activity	2/172	17/29370	0.0043769	0.1086922	0.1036627	2	gene_Cg_evm.model.chr8A.304/gene_Cg_evm.model.chr15B.31	gene_Car0223200/gene_Car0317320
GO:0051087	protein-folding chaperone binding	2/172	21/29370	0.0066555	0.1358322	0.1295468	2	gene_Cg_evm.model.chr17B.156/gene_Cg_evm.model.chr17A.147	gene_Car0291520/gene_Car0315710
GO:0015671	oxygen transport	2/172	22/29370	0.007293	0.1358322	0.1295468	2	gene_Cg_evm.model.chr3B.1287/gene_Cg_evm.model.chr3B.1501	gene_Car0010750/gene_Car0012670
GO:0019825	oxygen binding	2/172	27/29370	0.0108715	0.1714291	0.1634965	2	gene_Cg_evm.model.chr3B.1287/gene_Cg_evm.model.chr3B.1501	gene_Car0010750/gene_Car0012670
GO:0006357	regulation of transcription by RNA polymerase II	6/172	319/29370	0.0115053	0.1714291	0.1634965	6	gene_Cg_evm.model.chr3B.661/gene_Cg_evm.model.chr3A.236/gene_Cg_evm.model.chr14B.658/gene_Cg_evm.model.chr20B.575/gene_Cg_evm.model.chr14A.503/gene_Cg_evm.model.chr10A.486	gene_Car0005100/gene_Car0115390/gene_Car0196210/gene_Car0255820/gene_Car0279140/gene_Car0357190
GO:0016491	oxidoreductase activity	6/172	356/29370	0.0187633	0.254157	0.2423963	6	gene_Cg_evm.model.chr6A.53/gene_Cg_evm.model.chr6B.758/gene_Cg_evm.model.chr8A.514/gene_Cg_evm.model.chr21B.392/gene_Cg_evm.model.chr15B.461/gene_Cg_evm.model.chr25A.500	gene_Car0152800/gene_Car0186750/gene_Car0225380/gene_Car0305420/gene_Car0321400/gene_Car0417680
GO:0008233	peptidase activity	2/172	41/29370	0.0240788	0.2759798	0.2632092	2	gene_Cg_evm.model.chr7A.1005/gene_Cg_evm.model.chr12B.400	gene_Car0023790/gene_Car0335500
GO:0015267	channel activity	2/172	41/29370	0.0240788	0.2759798	0.2632092	2	gene_Cg_evm.model.chr2B.945/gene_Cg_evm.model.chr18A.82	gene_Car0124510/gene_Car0168990
GO:0035091	phosphatidylinositol binding	3/172	133/29370	0.0435504	0.3013337	0.28739	3	gene_Cg_evm.model.chr8B.934/gene_Cg_evm.model.chr2B.481/gene_Cg_evm.model.chr21B.441	gene_Car0112870/gene_Car0129430/gene_Car0304850
GO:0004519	endonuclease activity	1/172	10/29370	0.0570522	0.3013337	0.28739	1	gene_Cg_evm.model.chr4B.663	gene_Car0184100
GO:0008199	ferric iron binding	1/172	10/29370	0.0570522	0.3013337	0.28739	1	gene_Cg_evm.model.chr3B.969	gene_Car0007960
GO:0043248	proteasome assembly	1/172	10/29370	0.0570522	0.3013337	0.28739	1	gene_Cg_evm.model.chr21A.112	gene_Car0415560
GO:0051568	histone H3-K4 methylation	1/172	10/29370	0.0570522	0.3013337	0.28739	1	gene_Cg_evm.model.chr10A.63	gene_Car0353260
GO:0061817	endoplasmic reticulum-plasma membrane tethering	1/172	10/29370	0.0570522	0.3013337	0.28739	1	gene_Cg_evm.model.chr23A.358	gene_Car0377810
GO:0016616	oxidoreductase activity, acting on the CH-OH group of donors, NAD or NADP as acceptor	2/172	69/29370	0.0619858	0.3013337	0.28739	2	gene_Cg_evm.model.chr21B.392/gene_Cg_evm.model.chr25A.500	gene_Car0305420/gene_Car0417680
GO:0050660	flavin adenine dinucleotide binding	2/172	69/29370	0.0619858	0.3013337	0.28739	2	gene_Cg_evm.model.chr6A.53/gene_Cg_evm.model.chr15B.461	gene_Car0152800/gene_Car0321400
GO:0004017	adenylate kinase activity	1/172	11/29370	0.0625763	0.3013337	0.28739	1	gene_Cg_evm.model.chr5A.374	gene_Car0039880
GO:0004308	exo-alpha-sialidase activity	1/172	11/29370	0.0625763	0.3013337	0.28739	1	gene_Cg_evm.model.chr19B.165	gene_Car0213280

GO:0004364	glutathione transferase activity	1/172	11/29370	0.0625763	0.3013337	0.28739	1	gene_Cg_evm.model.chr13B.22	gene_Car0203260
GO:0006400	tRNA modification	1/172	11/29370	0.0625763	0.3013337	0.28739	1	gene_Cg_evm.model.chr24B.277	gene_Car0348940
GO:0030168	platelet activation	1/172	11/29370	0.0625763	0.3013337	0.28739	1	gene_Cg_evm.model.chr18A.687	gene_Car0163180
GO:0033177	proton-transporting two-sector ATPase complex, proton-transporting domain	1/172	11/29370	0.0625763	0.3013337	0.28739	1	gene_Cg_evm.model.chr2B.427	gene_Car0129990
GO:0006935	chemotaxis	2/172	70/29370	0.0635705	0.3013337	0.28739	2	gene_Cg_evm.model.chr7A.150/gene_Cg_evm.model.chr18B.612	gene_Car0015580/gene_Car0271520
GO:0003724	RNA helicase activity	2/172	71/29370	0.0651688	0.3013337	0.28739	2	gene_Cg_evm.model.chr10A.65/gene_Cg_evm.model.chr10A.84	gene_Car0353280/gene_Car0353430
GO:0003873	6-phosphofructo-2-kinase activity	1/172	12/29370	0.0680682	0.3013337	0.28739	1	gene_Cg_evm.model.chr4A.474	gene_Car0050810
GO:0006003	fructose 2,6-bisphosphate metabolic process	1/172	12/29370	0.0680682	0.3013337	0.28739	1	gene_Cg_evm.model.chr4A.474	gene_Car0050810
GO:0008643	carbohydrate transport	1/172	12/29370	0.0680682	0.3013337	0.28739	1	gene_Cg_evm.model.chr19B.225	gene_Car0213820
GO:0031072	heat shock protein binding	1/172	12/29370	0.0680682	0.3013337	0.28739	1	gene_Cg_evm.model.chr25A.404	gene_Car0418640
GO:0016874	ligase activity	1/172	13/29370	0.0735281	0.3013337	0.28739	1	gene_Cg_evm.model.chr23A.128	gene_Car0380140
GO:0004298	threonine-type endopeptidase activity	1/172	14/29370	0.0789562	0.3013337	0.28739	1	gene_Cg_evm.model.chr2A.271	gene_Car0159930
GO:0006000	fructose metabolic process	1/172	14/29370	0.0789562	0.3013337	0.28739	1	gene_Cg_evm.model.chr4A.474	gene_Car0050810
GO:0008320	protein transmembrane transporter activity	1/172	14/29370	0.0789562	0.3013337	0.28739	1	gene_Cg_evm.model.chr5B.813	gene_Car0074230
GO:0004176	ATP-dependent peptidase activity	1/172	15/29370	0.0843527	0.3013337	0.28739	1	gene_Cg_evm.model.chr22A.24	gene_Car0368380
GO:0006879	intracellular iron ion homeostasis	1/172	15/29370	0.0843527	0.3013337	0.28739	1	gene_Cg_evm.model.chr3B.969	gene_Car0007960
GO:0016763	pentosyltransferase activity	1/172	15/29370	0.0843527	0.3013337	0.28739	1	gene_Cg_evm.model.chr24B.277	gene_Car0348940
GO:0016818	hydrolase activity, acting on acid anhydrides, in phosphorus-containing anhydrides	1/172	15/29370	0.0843527	0.3013337	0.28739	1	gene_Cg_evm.model.chr18A.119	gene_Car0168630
GO:0005833	hemoglobin complex	1/172	16/29370	0.0897178	0.3013337	0.28739	1	gene_Cg_evm.model.chr3B.1287	gene_Car0010750
GO:0005978	glycogen biosynthetic process	1/172	16/29370	0.0897178	0.3013337	0.28739	1	gene_Cg_evm.model.chr3A.362	gene_Car0116620
GO:0048188	Set1C/COMPASS complex	1/172	16/29370	0.0897178	0.3013337	0.28739	1	gene_Cg_evm.model.chr10A.63	gene_Car0353260
GO:0000978	RNA polymerase II cis-regulatory region sequence-specific DNA binding	2/172	88/29370	0.0942289	0.3013337	0.28739	2	gene_Cg_evm.model.chr3B.661/gene_Cg_evm.model.chr3A.236	gene_Car0005100/gene_Car0115390
GO:0030150	protein import into mitochondrial matrix	1/172	17/29370	0.0950516	0.3013337	0.28739	1	gene_Cg_evm.model.chr5B.813	gene_Car0074230
GO:0070006	metalloaminopeptidase activity	1/172	17/29370	0.0950516	0.3013337	0.28739	1	gene_Cg_evm.model.chr8B.262	gene_Car0105660
GO:0006633	fatty acid biosynthetic process	1/172	18/29370	0.1003543	0.3115165	0.2971016	1	gene_Cg_evm.model.chr3B.160	gene_Car0001750
GO:0030544	Hsp70 protein binding	1/172	19/29370	0.1056261	0.3144655	0.2999141	1	gene_Cg_evm.model.chr25A.404	gene_Car0418640
GO:0015631	tubulin binding	1/172	20/29370	0.1108673	0.3144655	0.2999141	1	gene_Cg_evm.model.chr2A.344	gene_Car0159090
GO:0016298	lipase activity	1/172	20/29370	0.1108673	0.3144655	0.2999141	1	gene_Cg_evm.model.chr8A.304	gene_Car0223200

GO:0030163	protein catabolic process	1/172	20/29370	0.1108673	0.3144655	0.2999141	1	gene_Cg_evm.model.chr22A.24	gene_Car0368380
GO:0006417	regulation of translation	1/172	21/29370	0.1160779	0.3144655	0.2999141	1	gene_Cg_evm.model.chr22B.965	gene_Car0094520
GO:0030833	regulation of actin filament polymerization	1/172	21/29370	0.1160779	0.3144655	0.2999141	1	gene_Cg_evm.model.chr19B.222	gene_Car0213790
GO:0032259	methylation	1/172	21/29370	0.1160779	0.3144655	0.2999141	1	gene_Cg_evm.model.chr6A.606	gene_Car0146950
GO:0006351	DNA-templated transcription	2/172	101/29370	0.1184772	0.3152339	0.300647	2	gene_Cg_evm.model.chr7A.653/gene_Cg_evm.model.chr14B.21	gene_Car0020470/gene_Car0202370
GO:0045104	intermediate filament cytoskeleton organization	1/172	23/29370	0.1264081	0.3247717	0.3097434	1	gene_Cg_evm.model.chr19B.557	gene_Car0217000
GO:0008168	methyltransferase activity	2/172	109/29370	0.1340885	0.3247717	0.3097434	2	gene_Cg_evm.model.chr22B.679/gene_Cg_evm.model.chr6A.606	gene_Car0091460/gene_Car0146950
GO:0008484	sulfuric ester hydrolase activity	1/172	25/29370	0.1366184	0.3247717	0.3097434	1	gene_Cg_evm.model.chr10A.125	gene_Car0353790
GO:0033179	proton-transporting V-type ATPase, V0 domain	1/172	25/29370	0.1366184	0.3247717	0.3097434	1	gene_Cg_evm.model.chr2B.427	gene_Car0129990
GO:0004518	nuclease activity	1/172	26/29370	0.1416789	0.3247717	0.3097434	1	gene_Cg_evm.model.chr4B.663	gene_Car0184100
GO:0008324	monoatomic cation transmembrane transporter activity	1/172	26/29370	0.1416789	0.3247717	0.3097434	1	gene_Cg_evm.model.chr25A.283	gene_Car0419880
GO:0019752	carboxylic acid metabolic process	1/172	26/29370	0.1416789	0.3247717	0.3097434	1	gene_Cg_evm.model.chr25A.500	gene_Car0417680
GO:0030145	manganese ion binding	1/172	26/29370	0.1416789	0.3247717	0.3097434	1	gene_Cg_evm.model.chr8B.262	gene_Car0105660
GO:0031625	ubiquitin protein ligase binding	1/172	26/29370	0.1416789	0.3247717	0.3097434	1	gene_Cg_evm.model.chr2A.344	gene_Car0159090
GO:0004715	non-membrane spanning protein tyrosine kinase activity	1/172	27/29370	0.14671	0.3312089	0.3158827	1	gene_Cg_evm.model.chr2B.883	gene_Car0125150
GO:0015078	proton transmembrane transporter activity	1/172	28/29370	0.1517117	0.3373887	0.3217766	1	gene_Cg_evm.model.chr2B.427	gene_Car0129990
GO:0051537	2 iron, 2 sulfur cluster binding	1/172	29/29370	0.1566843	0.343323	0.3274362	1	gene_Cg_evm.model.chr15B.461	gene_Car0321400
GO:0006952	defense response	1/172	30/29370	0.1616279	0.3490226	0.3328721	1	gene_Cg_evm.model.chr7A.150	gene_Car0015580
GO:0003899	DNA-directed 5'-3' RNA polymerase activity	1/172	31/29370	0.1665427	0.354498	0.3380942	1	gene_Cg_evm.model.chr14B.21	gene_Car0202370
GO:0003697	single-stranded DNA binding	1/172	32/29370	0.1714288	0.3547625	0.3383464	1	gene_Cg_evm.model.chr4A.841	gene_Car0054250
GO:0016620	oxidoreductase activity, acting on the aldehyde or oxo group of donors, NAD or NADP as acceptor	1/172	32/29370	0.1714288	0.3547625	0.3383464	1	gene_Cg_evm.model.chr8A.514	gene_Car0225380
GO:0005741	mitochondrial outer membrane	1/172	35/29370	0.1859171	0.3689981	0.3519233	1	gene_Cg_evm.model.chr5B.813	gene_Car0074230
GO:0046856	phosphatidylinositol dephosphorylation	1/172	35/29370	0.1859171	0.3689981	0.3519233	1	gene_Cg_evm.model.chr5A.1085	gene_Car0047080
GO:0050661	NADP binding	1/172	35/29370	0.1859171	0.3689981	0.3519233	1	gene_Cg_evm.model.chr23B.165	gene_Car0268660
GO:0007010	cytoskeleton organization	1/172	36/29370	0.1906903	0.3689981	0.3519233	1	gene_Cg_evm.model.chr11B.30	gene_Car0395050
GO:0015293	symporter activity	1/172	36/29370	0.1906903	0.3689981	0.3519233	1	gene_Cg_evm.model.chr19B.225	gene_Car0213820

GO:0006099	tricarboxylic acid cycle	1/172	38/29370	0.2001534	0.3823443	0.3646519	1	gene_Cg_evm.model.chr11B.496	gene_Car0399780
GO:0042981	regulation of apoptotic process	2/172	143/29370	0.2044044	0.3855223	0.3676828	2	gene_Cg_evm.model.chr7B.444/gene_Cg_evm.model.chr11B.519	gene_Car0029320/gene_Car0399980
GO:0016740	transferase activity	1/172	40/29370	0.2095065	0.389112	0.3711065	1	gene_Cg_evm.model.chr3B.160	gene_Car0001750
GO:0004950	chemokine receptor activity	1/172	41/29370	0.2141422	0.389112	0.3711065	1	gene_Cg_evm.model.chr18B.612	gene_Car0271520
GO:0030198	extracellular matrix organization	1/172	41/29370	0.2141422	0.389112	0.3711065	1	gene_Cg_evm.model.chr25B.62	gene_Car0408490
GO:0005839	proteasome core complex	1/172	42/29370	0.2187509	0.3926973	0.3745259	1	gene_Cg_evm.model.chr2A.271	gene_Car0159930
GO:0051603	proteolysis involved in protein catabolic process	1/172	44/29370	0.2278877	0.4042294	0.3855244	1	gene_Cg_evm.model.chr2A.271	gene_Car0159930
GO:0003713	transcription coactivator activity	1/172	46/29370	0.2369184	0.4152581	0.3960427	1	gene_Cg_evm.model.chr12A.82	gene_Car0331320
GO:0004197	cysteine-type endopeptidase activity	1/172	48/29370	0.245844	0.4152581	0.3960427	1	gene_Cg_evm.model.chr14A.122	gene_Car0282840
GO:0003729	mRNA binding	1/172	49/29370	0.2502678	0.4152581	0.3960427	1	gene_Cg_evm.model.chr22B.965	gene_Car0094520
GO:0016592	mediator complex	1/172	49/29370	0.2502678	0.4152581	0.3960427	1	gene_Cg_evm.model.chr10A.486	gene_Car0357190
GO:0046961	proton-transporting ATPase activity, rotational mechanism	1/172	49/29370	0.2502678	0.4152581	0.3960427	1	gene_Cg_evm.model.chr2B.427	gene_Car0129990
GO:0043130	ubiquitin binding	1/172	50/29370	0.2546658	0.4152581	0.3960427	1	gene_Cg_evm.model.chr2B.481	gene_Car0129430
GO:0006139	nucleobase-containing compound metabolic process	1/172	51/29370	0.2590381	0.4152581	0.3960427	1	gene_Cg_evm.model.chr18A.119	gene_Car0168630
GO:0020037	heme binding	2/172	169/29370	0.260428	0.4152581	0.3960427	2	gene_Cg_evm.model.chr3B.1287/gene_Cg_evm.model.chr3B.1501	gene_Car0010750/gene_Car0012670
GO:0004866	endopeptidase inhibitor activity	1/172	52/29370	0.263385	0.4152581	0.3960427	1	gene_Cg_evm.model.chr1A.856	gene_Car0086390
GO:0007017	microtubule-based process	1/172	52/29370	0.263385	0.4152581	0.3960427	1	gene_Cg_evm.model.chr24B.37	gene_Car0346730
GO:0005856	cytoskeleton	2/172	171/29370	0.2647619	0.4152581	0.3960427	2	gene_Cg_evm.model.chr2B.883/gene_Cg_evm.model.chr19B.557	gene_Car0125150/gene_Car0217000
GO:0005200	structural constituent of cytoskeleton	1/172	54/29370	0.2720028	0.4200493	0.4006122	1	gene_Cg_evm.model.chr24B.37	gene_Car0346730
GO:0046872	metal ion binding	4/172	453/29370	0.2747204	0.4200493	0.4006122	4	gene_Cg_evm.model.chr8B.262/gene_Cg_evm.model.chr6B.294/gene_Cg_evm.model.chr13A.592/gene_Cg_evm.model.chr23B.748	gene_Car0105660/gene_Car0191790/gene_Car0242840/gene_Car0263070
GO:0006418	tRNA aminoacylation for protein translation	1/172	55/29370	0.276274	0.4200493	0.4006122	1	gene_Cg_evm.model.chr14A.607	gene_Car0278120
GO:0003678	DNA helicase activity	1/172	56/29370	0.2805203	0.4221973	0.4026608	1	gene_Cg_evm.model.chr18A.119	gene_Car0168630
GO:0004386	helicase activity	1/172	57/29370	0.2847419	0.4242654	0.4046332	1	gene_Cg_evm.model.chr18A.119	gene_Car0168630
GO:0004812	aminoacyl-tRNA ligase activity	1/172	62/29370	0.3054831	0.4462449	0.4255956	1	gene_Cg_evm.model.chr14A.607	gene_Car0278120
GO:0030036	actin cytoskeleton organization	1/172	62/29370	0.3054831	0.4462449	0.4255956	1	gene_Cg_evm.model.chr22B.994	gene_Car0094760
GO:0003712	transcription coregulator activity	1/172	66/29370	0.3216446	0.4652917	0.4437611	1	gene_Cg_evm.model.chr10A.486	gene_Car0357190
GO:0006869	lipid transport	1/172	67/29370	0.3256262	0.4665222	0.4449346	1	gene_Cg_evm.model.chr23A.358	gene_Car0377810
GO:0005874	microtubule	1/172	68/29370	0.3295846	0.4676962	0.4460543	1	gene_Cg_evm.model.chr24B.37	gene_Car0346730
GO:0006812	monoatomic cation transport	1/172	69/29370	0.3335199	0.4688157	0.447122	1	gene_Cg_evm.model.chr25A.283	gene_Car0419880
GO:0008009	chemokine activity	1/172	73/29370	0.3490326	0.4860361	0.4635456	1	gene_Cg_evm.model.chr7A.150	gene_Car0015580
GO:0006260	DNA replication	1/172	75/29370	0.3566539	0.4920502	0.4692814	1	gene_Cg_evm.model.chr4A.841	gene_Car0054250
GO:0005922	connexin complex	1/172	78/29370	0.3679197	0.5029361	0.4796635	1	gene_Cg_evm.model.chr20B.625	gene_Car0255320

GO:0008237	metallopeptidase activity	2/172	223/29370	0.3760093	0.5060114	0.4825965	2	gene_Cg_evm.model.chr7A.1005/gene_Cg_evm.model.chr10A.235	gene_Car0023790/gene_Car0354750
GO:0030170	pyridoxal phosphate binding	1/172	81/29370	0.3789894	0.5060114	0.4825965	1	gene_Cg_evm.model.chr6A.509	gene_Car0148010
GO:0009058	biosynthetic process	1/172	82/29370	0.3826363	0.5060114	0.4825965	1	gene_Cg_evm.model.chr22A.205	gene_Car0369860
GO:0140359	ABC-type transporter activity	1/172	83/29370	0.3862619	0.5060114	0.4825965	1	gene_Cg_evm.model.chr3A.932	gene_Car0122220
GO:0003777	microtubule motor activity	1/172	84/29370	0.3898663	0.5060114	0.4825965	1	gene_Cg_evm.model.chr18B.324	gene_Car0274220
GO:0003779	actin binding	2/172	230/29370	0.3905457	0.5060114	0.4825965	2	gene_Cg_evm.model.chr22B.994/gene_Cg_evm.model.chr11B.30	gene_Car0094760/gene_Car0395050
GO:0016746	acyltransferase activity	1/172	89/29370	0.4075752	0.5190488	0.4950306	1	gene_Cg_evm.model.chr3B.160	gene_Car0001750
GO:1902600	proton transmembrane transport	1/172	89/29370	0.4075752	0.5190488	0.4950306	1	gene_Cg_evm.model.chr2B.427	gene_Car0129990
GO:0008234	cysteine-type peptidase activity	1/172	92/29370	0.4179543	0.5277558	0.5033347	1	gene_Cg_evm.model.chr14A.122	gene_Car0282840
GO:0016787	hydrolase activity	2/172	248/29370	0.427204	0.5349025	0.5101507	2	gene_Cg_evm.model.chr3B.1204/gene_Cg_evm.model.chr15B.31	gene_Car0010180/gene_Car0317320
GO:0051015	actin filament binding	1/172	115/29370	0.4917462	0.6105848	0.582331	1	gene_Cg_evm.model.chr11B.30	gene_Car0395050
GO:0016459	myosin complex	1/172	118/29370	0.5006585	0.6165133	0.5879851	1	gene_Cg_evm.model.chr7B.811	gene_Car0032810
GO:0006886	intracellular protein transport	2/172	291/29370	0.5097766	0.6225961	0.5937864	2	gene_Cg_evm.model.chr10A.362/gene_Cg_evm.model.chr10B.5	gene_Car0355970/gene_Car0359950
GO:0007154	cell communication	1/172	124/29370	0.5180196	0.6227579	0.5939408	1	gene_Cg_evm.model.chr20B.625	gene_Car0255320
GO:0005737	cytoplasm	3/172	468/29370	0.5182683	0.6227579	0.5939408	3	gene_Cg_evm.model.chr9B.679/gene_Cg_evm.model.chr8B.262/gene_Cg_evm.model.chr25A.500	gene_Car0097040/gene_Car0105660/gene_Car0417680
GO:0007018	microtubule-based movement	1/172	127/29370	0.5264737	0.6258697	0.5969085	1	gene_Cg_evm.model.chr18B.324	gene_Car0274220
GO:0004725	protein tyrosine phosphatase activity	1/172	128/29370	0.5292589	0.6258697	0.5969085	1	gene_Cg_evm.model.chr23B.632	gene_Car0264120
GO:0038023	signaling receptor activity	1/172	131/29370	0.537517	0.6306302	0.6014488	1	gene_Cg_evm.model.chr20A.621	gene_Car0294650
GO:0005615	extracellular space	1/172	149/29370	0.5841269	0.6756937	0.6444271	1	gene_Cg_evm.model.chr1A.856	gene_Car0086390
GO:0031267	small GTPase binding	1/172	150/29370	0.5865748	0.6756937	0.6444271	1	gene_Cg_evm.model.chr19B.222	gene_Car0213790
GO:0006281	DNA repair	1/172	152/29370	0.5914278	0.6756937	0.6444271	1	gene_Cg_evm.model.chr4B.663	gene_Car0184100
GO:0005179	hormone activity	1/172	155/29370	0.5986011	0.6756937	0.6444271	1	gene_Cg_evm.model.chr4B.550	gene_Car0183000
GO:0006629	lipid metabolic process	1/172	155/29370	0.5986011	0.6756937	0.6444271	1	gene_Cg_evm.model.chr8A.304	gene_Car0223200
GO:0008289	lipid binding	1/172	164/29370	0.6193786	0.6936824	0.6615833	1	gene_Cg_evm.model.chr23A.358	gene_Car0377810
GO:0006486	protein glycosylation	1/172	166/29370	0.6238486	0.6936824	0.6615833	1	gene_Cg_evm.model.chr10A.230	gene_Car0354700
GO:0007155	cell adhesion	2/172	373/29370	0.6443234	0.7111422	0.6782352	2	gene_Cg_evm.model.chr17B.815/gene_Cg_evm.model.chr17A.759	gene_Car0284980/gene_Car0309430
GO:0005096	GTPase activator activity	1/172	178/29370	0.6495934	0.7116869	0.6787547	1	gene_Cg_evm.model.chr16B.310	gene_Car0141350
GO:0006511	ubiquitin-dependent protein catabolic process	1/172	186/29370	0.6657755	0.7220451	0.6886336	1	gene_Cg_evm.model.chr19B.574	gene_Car0217240
GO:0000166	nucleotide binding	1/172	188/29370	0.6697036	0.7220451	0.6886336	1	gene_Cg_evm.model.chr14A.607	gene_Car0278120
GO:0004222	metalloendopeptidase activity	1/172	190/29370	0.6735857	0.7220451	0.6886336	1	gene_Cg_evm.model.chr10A.235	gene_Car0354750
GO:0005506	iron ion binding	1/172	199/29370	0.6905015	0.734003	0.7000382	1	gene_Cg_evm.model.chr3B.1501	gene_Car0012670
GO:0008017	microtubule binding	1/172	203/29370	0.6977371	0.734003	0.7000382	1	gene_Cg_evm.model.chr18B.324	gene_Car0274220
GO:0004713	protein tyrosine kinase activity	1/172	204/29370	0.6995196	0.734003	0.7000382	1	gene_Cg_evm.model.chr2B.883	gene_Car0125150
GO:0006955	immune response	1/172	208/29370	0.7065456	0.7361909	0.7021248	1	gene_Cg_evm.model.chr7A.150	gene_Car0015580
GO:0004842	ubiquitin-protein transferase activity	1/172	232/29370	0.7454002	0.7712821	0.7355923	1	gene_Cg_evm.model.chr13B.808	gene_Car0211070

GO:0004252	serine-type endopeptidase activity	1/172	239/29370	0.755737	0.7756555	0.7397633	1	gene_Cg_evm.model.chr22A.24	gene_Car0368380
GO:0006470	protein dephosphorylation	1/172	242/29370	0.7600383	0.7756555	0.7397633	1	gene_Cg_evm.model.chr23B.632	gene_Car0264120
GO:0016791	phosphatase activity	1/172	293/29370	0.8226415	0.8338339	0.7952496	1	gene_Cg_evm.model.chr5A.1085	gene_Car0047080
GO:0035556	intracellular signal transduction	1/172	367/29370	0.8857276	0.8917122	0.8504497	1	gene_Cg_evm.model.chr2B.883	gene_Car0125150
GO:0046983	protein dimerization activity	1/172	403/29370	0.9077664	0.9077664	0.8657609	1	gene_Cg_evm.model.chr14B.21	gene_Car0202370

Supplementary Table 11-4. GO enrichment of DEGs in cluster 2 of spleen.

ID	Description	Gene Ratio	BgRatio	pvalue	p.adjust	qvalue	Count	C.gibelio_homolog_id	C.cuvieri_homolog_id
GO:0008081	phosphoric diester hydrolase activity	8/283	118/29370	1.971E-05	0.0025669	0.0022611	8	gene_Cg_evm.model.chr7A.482/gene_Cg_evm.model.chr7B.431/gene_Cg_evm.model.chr2B.885/gene_Cg_evm.model.chr2A.561/gene_Cg_evm.model.chr18A.6/gene_Cg_evm.model.chr8A.580/gene_Cg_evm.model.chr17B.785/gene_Cg_evm.model.chr24A.309	gene_Car0018860/gene_Car0029180/gene_Car0125140/gene_Car0156890/gene_Car0169720/gene_Car0226110/gene_Car0285300/gene_Car0384700
GO:0004114	3',5'-cyclic-nucleotide phosphodiesterase activity	6/283	61/29370	2.702E-05	0.0025669	0.0022611	6	gene_Cg_evm.model.chr7A.482/gene_Cg_evm.model.chr7B.431/gene_Cg_evm.model.chr2B.885/gene_Cg_evm.model.chr18A.6/gene_Cg_evm.model.chr8A.580/gene_Cg_evm.model.chr24A.309	gene_Car0018860/gene_Car0029180/gene_Car0125140/gene_Car0169720/gene_Car0226110/gene_Car0384700
GO:0005085	guanyl-nucleotide exchange factor activity	13/283	372/29370	7.382E-05	0.0046753	0.0041184	13	gene_Cg_evm.model.chr5B.532/gene_Cg_evm.model.chr6A.172/gene_Cg_evm.model.chr6A.121/gene_Cg_evm.model.chr4B.283/gene_Cg_evm.model.chr6B.123/gene_Cg_evm.model.chr8A.299/gene_Cg_evm.model.chr21B.20/gene_Cg_evm.model.chr12A.358/gene_Cg_evm.model.chr10B.437/gene_Cg_evm.model.chr11A.352/gene_Cg_evm.model.chr11B.481/gene_Cg_evm.model.chr21A.87/gene_Cg_evm.model.chr21A.11	gene_Car0071370/gene_Car0151580/gene_Car0152110/gene_Car0180360/gene_Car0193370/gene_Car0223150/gene_Car0309120/gene_Car0328670/gene_Car0363920/gene_Car0391070/gene_Car0399620/gene_Car0415770/gene_Car0416430
GO:0008277	regulation of G protein-coupled receptor signaling pathway	4/283	26/29370	0.0001067	0.0050703	0.0044664	4	gene_Cg_evm.model.chr3B.1124/gene_Cg_evm.model.chr3A.628/gene_Cg_evm.model.chr12A.22/gene_Cg_evm.model.chr24B.647	gene_Car0009340/gene_Car0119200/gene_Car0331900/gene_Car0352580
GO:0006807	nitrogen compound metabolic process	3/283	22/29370	0.0011902	0.0402655	0.0354693	3	gene_Cg_evm.model.chr2B.733/gene_Cg_evm.model.chr2A.654/gene_Cg_evm.model.chr6B.363	gene_Car0126750/gene_Car0155890/gene_Car0190810
GO:0003729	mRNA binding	4/283	49/29370	0.0012715	0.0402655	0.0354693	4	gene_Cg_evm.model.chr7B.225/gene_Cg_evm.model.chr13A.254/gene_Cg_evm.model.chr18B.702/gene_Cg_evm.model.chr12A.316	gene_Car0026830/gene_Car0239090/gene_Car0270700/gene_Car0329090
GO:0007264	small GTPase mediated signal transduction	7/283	185/29370	0.0021926	0.0595133	0.0524245	7	gene_Cg_evm.model.chr5B.532/gene_Cg_evm.model.chr3A.309/gene_Cg_evm.model.chr8A.299/gene_Cg_evm.model.chr21B.20/gene_Cg_evm.model.chr10B.437/gene_Cg_evm.model.chr21A.87/gene_Cg_evm.model.chr21A.11	gene_Car0071370/gene_Car0116130/gene_Car0223150/gene_Car0309120/gene_Car0363920/gene_Car0415770/gene_Car0416430
GO:0003755	peptidyl-prolyl cis-trans isomerase activity	4/283	68/29370	0.0042381	0.0921631	0.0811853	4	gene_Cg_evm.model.chr6A.596/gene_Cg_evm.model.chr6B.632/gene_Cg_evm.model.chr20A.646/gene_Cg_evm.model.chr15B.448	gene_Car0147050/gene_Car0188070/gene_Car0294390/gene_Car0321280
GO:0003873	6-phosphofructo-2-kinase activity	2/283	12/29370	0.0057292	0.0921631	0.0811853	2	gene_Cg_evm.model.chr6A.555/gene_Cg_evm.model.chr6B.576	gene_Car0147490/gene_Car0188600
GO:0006003	fructose 2,6-bisphosphate metabolic process	2/283	12/29370	0.0057292	0.0921631	0.0811853	2	gene_Cg_evm.model.chr6A.555/gene_Cg_evm.model.chr6B.576	gene_Car0147490/gene_Car0188600
GO:0008349	MAP kinase kinase kinase kinase activity	2/283	12/29370	0.0057292	0.0921631	0.0811853	2	gene_Cg_evm.model.chr7A.257/gene_Cg_evm.model.chr7B.233	gene_Car0016620/gene_Car0026920
GO:0006914	autophagy	3/283	38/29370	0.0058208	0.0921631	0.0811853	3	gene_Cg_evm.model.chr18A.33/gene_Cg_evm.model.chr15B.369/gene_Cg_evm.model.chr15A.310	gene_Car0169460/gene_Car0320470/gene_Car0342540
GO:0016874	ligase activity	2/283	13/29370	0.006728	0.0926214	0.0815889	2	gene_Cg_evm.model.chr5A.226/gene_Cg_evm.model.chr5B.68	gene_Car0038340/gene_Car0066550
GO:0006000	fructose metabolic process	2/283	14/29370	0.0077997	0.0926214	0.0815889	2	gene_Cg_evm.model.chr6A.555/gene_Cg_evm.model.chr6B.576	gene_Car0147490/gene_Car0188600
GO:0015026	coreceptor activity	2/283	14/29370	0.0077997	0.0926214	0.0815889	2	gene_Cg_evm.model.chr3B.1124/gene_Cg_evm.model.chr24B.647	gene_Car0009340/gene_Car0352580
GO:0038036	sphingosine-1-phosphate receptor activity	2/283	14/29370	0.0077997	0.0926214	0.0815889	2	gene_Cg_evm.model.chr3B.615/gene_Cg_evm.model.chr3A.174	gene_Car0004640/gene_Car0114790
GO:0035556	intracellular signal transduction	9/283	367/29370	0.0096455	0.1078025	0.0949617	9	gene_Cg_evm.model.chr3A.628/gene_Cg_evm.model.chr2A.561/gene_Cg_evm.model.chr19B.787/gene_Cg_evm.model.chr17B.785/gene_Cg_evm.model.chr12A.199/gene_Cg_evm.model.chr12A.22/gene_Cg_evm.model.chr24B.346/gene_Cg_evm.model.chr10B.437/gene_Cg_evm.model.chr24A.302	gene_Car0119200/gene_Car0156890/gene_Car0219440/gene_Car0285300/gene_Car0330240/gene_Car0331900/gene_Car0349630/gene_Car0363920/gene_Car0384640
GO:0006633	fatty acid biosynthetic process	2/283	18/29370	0.0127858	0.1349004	0.1188319	2	gene_Cg_evm.model.chr5A.226/gene_Cg_evm.model.chr5B.68	gene_Car0038340/gene_Car0066550
GO:0007009	plasma membrane organization	2/283	19/29370	0.0142	0.1349004	0.1188319	2	gene_Cg_evm.model.chr3B.562/gene_Cg_evm.model.chr12B.141	gene_Car0004180/gene_Car0337990

GO:0016712	oxidoreductase activity, acting on paired donors, with incorporation or reduction of molecular oxygen, reduced flavin or flavoprotein as one donor, and incorporation of one atom of oxygen	2/283	19/29370	0.0142	0.1349004	0.1188319	2	gene_Cg_evm.model.chr5B.223/gene_Cg_evm.model.chr20B.331	gene_Car0068230/gene_Car0258220
GO:0032958	inositol phosphate biosynthetic process	2/283	21/29370	0.0172199	0.1557993	0.1372415	2	gene_Cg_evm.model.chr6A.618/gene_Cg_evm.model.chr6A.559	gene_Car0146800/gene_Car0147450
GO:0003779	actin binding	6/283	230/29370	0.0245149	0.2117193	0.1865006	6	gene_Cg_evm.model.chr7B.552/gene_Cg_evm.model.chr13B.726/gene_Cg_evm.model.chr19B.280/gene_Cg_evm.model.chr13A.734/gene_Cg_evm.model.chr20A.66/gene_Cg_evm.model.chr25B.145	gene_Car0030360/gene_Car0210360/gene_Car0214310/gene_Car0244150/gene_Car0300570/gene_Car0407790
GO:0004935	adrenergic receptor activity	2/283	26/29370	0.0258244	0.2133319	0.1879212	2	gene_Cg_evm.model.chr1A.554/gene_Cg_evm.model.chr8A.244	gene_Car0083430/gene_Car0222470
GO:0003707	nuclear steroid receptor activity	2/283	27/29370	0.0277158	0.2167546	0.1909362	2	gene_Cg_evm.model.chr20B.498/gene_Cg_evm.model.chr20B.352	gene_Car0256540/gene_Car0257990
GO:0098609	cell-cell adhesion	4/283	120/29370	0.0290759	0.2167546	0.1909362	4	gene_Cg_evm.model.chr9B.860/gene_Cg_evm.model.chr9A.542/gene_Cg_evm.model.chr15A.165/gene_Cg_evm.model.chr24A.279	gene_Car0095320/gene_Car0171750/gene_Car0340580/gene_Car0384370
GO:0005125	cytokine activity	2/283	28/29370	0.0296612	0.2167546	0.1909362	2	gene_Cg_evm.model.chr12A.541/gene_Cg_evm.model.chr12B.541	gene_Car0326990/gene_Car0334150
GO:0016780	phosphotransferase activity, for other substituted phosphate groups	2/283	30/29370	0.0337089	0.2372105	0.2089555	2	gene_Cg_evm.model.chr12A.608/gene_Cg_evm.model.chr23A.377	gene_Car0326270/gene_Car0377600
GO:0016705	oxidoreductase activity, acting on paired donors, with incorporation or reduction of molecular oxygen	4/283	129/29370	0.0364804	0.2475459	0.2180598	4	gene_Cg_evm.model.chr5B.223/gene_Cg_evm.model.chr6B.608/gene_Cg_evm.model.chr20B.331/gene_Cg_evm.model.chr12B.576	gene_Car0068230/gene_Car0188270/gene_Car0258220/gene_Car0333800
GO:0005506	iron ion binding	5/283	199/29370	0.0441006	0.2882153	0.2538849	5	gene_Cg_evm.model.chr5B.223/gene_Cg_evm.model.chr5B.675/gene_Cg_evm.model.chr6B.608/gene_Cg_evm.model.chr20B.331/gene_Cg_evm.model.chr12B.576	gene_Car0068230/gene_Car0072850/gene_Car0188270/gene_Car0258220/gene_Car0333800
GO:0005540	hyaluronic acid binding	2/283	36/29370	0.0470246	0.2882153	0.2538849	2	gene_Cg_evm.model.chr4A.708/gene_Cg_evm.model.chr22B.561	gene_Car0052990/gene_Car0090350
GO:0007010	cytoskeleton organization	2/283	36/29370	0.0470246	0.2882153	0.2538849	2	gene_Cg_evm.model.chr13B.726/gene_Cg_evm.model.chr13A.734	gene_Car0210360/gene_Car0244150
GO:0006955	immune response	5/283	208/29370	0.05152	0.2983831	0.2628416	5	gene_Cg_evm.model.chr3A.838/gene_Cg_evm.model.chr16B.911/gene_Cg_evm.model.chr16A.546/gene_Cg_evm.model.chr12A.541/gene_Cg_evm.model.chr12B.541	gene_Car0121280/gene_Car0135170/gene_Car0232840/gene_Car0326990/gene_Car0334150
GO:0004435	phosphatidylinositol phospholipase C activity	2/283	38/29370	0.0518244	0.2983831	0.2628416	2	gene_Cg_evm.model.chr2A.561/gene_Cg_evm.model.chr17B.785	gene_Car0156890/gene_Car0285300
GO:0006629	lipid metabolic process	4/283	155/29370	0.0634253	0.3544355	0.3122174	4	gene_Cg_evm.model.chr5B.210/gene_Cg_evm.model.chr2A.561/gene_Cg_evm.model.chr17B.785/gene_Cg_evm.model.chr12A.452	gene_Car0068100/gene_Car0156890/gene_Car0285300/gene_Car0327800
GO:0007155	cell adhesion	7/283	373/29370	0.0705669	0.3625153	0.3193348	7	gene_Cg_evm.model.chr4A.708/gene_Cg_evm.model.chr1B.806/gene_Cg_evm.model.chr22B.561/gene_Cg_evm.model.chr6A.647/gene_Cg_evm.model.chr14B.803/gene_Cg_evm.model.chr14A.84/gene_Cg_evm.model.chr25B.145	gene_Car0052990/gene_Car0063800/gene_Car0090350/gene_Car0146530/gene_Car0194780/gene_Car0283260/gene_Car0407790
GO:0007166	cell surface receptor signaling pathway	4/283	168/29370	0.0799294	0.3625153	0.3193348	4	gene_Cg_evm.model.chr7A.495/gene_Cg_evm.model.chr2A.556/gene_Cg_evm.model.chr8A.286/gene_Cg_evm.model.chr20B.568	gene_Car0018980/gene_Car0156940/gene_Car0222990/gene_Car0255870
GO:0004674	protein serine/threonine kinase activity	6/283	317/29370	0.0874232	0.3625153	0.3193348	6	gene_Cg_evm.model.chr16B.514/gene_Cg_evm.model.chr18A.33/gene_Cg_evm.model.chr15B.369/gene_Cg_evm.model.chr15A.310/gene_Cg_evm.model.chr24B.346/gene_Cg_evm.model.chr24A.302	gene_Car0139150/gene_Car0169460/gene_Car0320470/gene_Car0342540/gene_Car0349630/gene_Car0384640

GO:0004497	monooxygenase activity	3/283	111/29370	0.0922941	0.3625153	0.3193348	3	gene_Cg_evm.model.chr5B.223/gene_Cg_evm.model.chr20B.331/gene_Cg_evm.model.chr12B.576	gene_Car0068230/gene_Car0258220/gene_Car0333800
GO:0000165	MAPK cascade	1/283	10/29370	0.0922978	0.3625153	0.3193348	1	gene_Cg_evm.model.chr16B.707	gene_Car0137270
GO:0004952	dopamine neurotransmitter receptor activity	1/283	10/29370	0.0922978	0.3625153	0.3193348	1	gene_Cg_evm.model.chr15A.330	gene_Car0342740
GO:0005315	inorganic phosphate transmembrane transporter activity	1/283	10/29370	0.0922978	0.3625153	0.3193348	1	gene_Cg_evm.model.chr8A.321	gene_Car0223370
GO:0015937	coenzyme A biosynthetic process	1/283	10/29370	0.0922978	0.3625153	0.3193348	1	gene_Cg_evm.model.chr12B.485	gene_Car0334660
GO:0106274	NAD+-protein-arginine ADP-ribosyltransferase activity	1/283	10/29370	0.0922978	0.3625153	0.3193348	1	gene_Cg_evm.model.chr1A.15	gene_Car0078070
GO:0140911	pore-forming activity	1/283	10/29370	0.0922978	0.3625153	0.3193348	1	gene_Cg_evm.model.chr3B.1190	gene_Car0010020
GO:0009966	regulation of signal transduction	2/283	53/29370	0.0925933	0.3625153	0.3193348	2	gene_Cg_evm.model.chr1B.967/gene_Cg_evm.model.chr20B.94	gene_Car0065180/gene_Car0260600
GO:0016301	kinase activity	3/283	115/29370	0.0999835	0.3625153	0.3193348	3	gene_Cg_evm.model.chr4A.839/gene_Cg_evm.model.chr6A.618/gene_Cg_evm.model.chr6A.559	gene_Car0054230/gene_Car0146800/gene_Car0147450
GO:0004767	sphingomyelin phosphodiesterase activity	1/283	11/29370	0.1010471	0.3625153	0.3193348	1	gene_Cg_evm.model.chr10B.453	gene_Car0364080
GO:0006605	protein targeting	1/283	11/29370	0.1010471	0.3625153	0.3193348	1	gene_Cg_evm.model.chr4B.7	gene_Car0177740
GO:0016286	small conductance calcium-activated potassium channel activity	1/283	11/29370	0.1010471	0.3625153	0.3193348	1	gene_Cg_evm.model.chr2A.528	gene_Car0157260
GO:0030168	platelet activation	1/283	11/29370	0.1010471	0.3625153	0.3193348	1	gene_Cg_evm.model.chr1A.554	gene_Car0083430
GO:0030866	cortical actin cytoskeleton organization	1/283	11/29370	0.1010471	0.3625153	0.3193348	1	gene_Cg_evm.model.chr20A.66	gene_Car0300570
GO:0045010	actin nucleation	1/283	11/29370	0.1010471	0.3625153	0.3193348	1	gene_Cg_evm.model.chr19B.280	gene_Car0214310
GO:0000775	chromosome, centromeric region	1/283	12/29370	0.1097124	0.3625153	0.3193348	1	gene_Cg_evm.model.chr21A.406	gene_Car0412630
GO:0005042	netrin receptor activity	1/283	12/29370	0.1097124	0.3625153	0.3193348	1	gene_Cg_evm.model.chr13A.518	gene_Car0242110
GO:0038007	netrin-activated signaling pathway	1/283	12/29370	0.1097124	0.3625153	0.3193348	1	gene_Cg_evm.model.chr13A.518	gene_Car0242110
GO:0005158	insulin receptor binding	1/283	13/29370	0.1182945	0.3625153	0.3193348	1	gene_Cg_evm.model.chr15B.632	gene_Car0323080
GO:0005523	tropomyosin binding	1/283	13/29370	0.1182945	0.3625153	0.3193348	1	gene_Cg_evm.model.chr23B.163	gene_Car0268680
GO:0008286	insulin receptor signaling pathway	1/283	13/29370	0.1182945	0.3625153	0.3193348	1	gene_Cg_evm.model.chr15B.632	gene_Car0323080
GO:0015467	G-protein activated inward rectifier potassium channel activity	1/283	13/29370	0.1182945	0.3625153	0.3193348	1	gene_Cg_evm.model.chr12A.474	gene_Car0327610
GO:0042043	neurexin family protein binding	1/283	13/29370	0.1182945	0.3625153	0.3193348	1	gene_Cg_evm.model.chr11B.510	gene_Car0399900
GO:0048193	Golgi vesicle transport	1/283	13/29370	0.1182945	0.3625153	0.3193348	1	gene_Cg_evm.model.chr23B.575	gene_Car0264680
GO:0051694	pointed-end actin filament capping	1/283	13/29370	0.1182945	0.3625153	0.3193348	1	gene_Cg_evm.model.chr23B.163	gene_Car0268680

GO:0002039	p53 binding	1/283	14/29370	0.1267941	0.3764198	0.3315831	1	gene_Cg_evm.model.chr19A.771	gene_Car0252390
GO:0008093	cytoskeletal anchor activity	1/283	14/29370	0.1267941	0.3764198	0.3315831	1	gene_Cg_evm.model.chr12B.141	gene_Car0337990
GO:0015269	calcium-activated potassium channel activity	1/283	15/29370	0.135212	0.3951117	0.3480485	1	gene_Cg_evm.model.chr2A.528	gene_Car0157260
GO:0022857	transmembrane transporter activity	6/283	361/29370	0.1373452	0.3951117	0.3480485	6	gene_Cg_evm.model.chr7B.937/gene_Cg_evm.model.chr13B.460/gene_Cg_evm.model.chr8A.567/gene_Cg_evm.model.chr19A.771/gene_Cg_evm.model.chr17A.489/gene_Cg_evm.model.chr15B.505	gene_Car0034040/gene_Car0207540/gene_Car0225970/gene_Car0252390/gene_Car0312230/gene_Car0321810
GO:0005496	steroid binding	1/283	16/29370	0.1435491	0.3951117	0.3480485	1	gene_Cg_evm.model.chr20B.352	gene_Car0257990
GO:0042613	MHC class II protein complex	1/283	16/29370	0.1435491	0.3951117	0.3480485	1	gene_Cg_evm.model.chr16B.911	gene_Car0135170
GO:0046872	metal ion binding	7/283	453/29370	0.1495105	0.3951117	0.3480485	7	gene_Cg_evm.model.chr7B.225/gene_Cg_evm.model.chr5B.68/gene_Cg_evm.model.chr4B.283/gene_Cg_evm.model.chr13A.254/gene_Cg_evm.model.chr18B.702/gene_Cg_evm.model.chr12A.316/gene_Cg_evm.model.chr11B.481	gene_Car0026830/gene_Car0066550/gene_Car0180360/gene_Car0239090/gene_Car0270700/gene_Car0329090/gene_Car0399620
GO:0001609	G protein-coupled adenosine receptor activity	1/283	17/29370	0.1518061	0.3951117	0.3480485	1	gene_Cg_evm.model.chr8A.333	gene_Car0223490
GO:0001973	G protein-coupled adenosine receptor signaling pathway	1/283	17/29370	0.1518061	0.3951117	0.3480485	1	gene_Cg_evm.model.chr8A.333	gene_Car0223490
GO:0004623	phospholipase A2 activity	1/283	17/29370	0.1518061	0.3951117	0.3480485	1	gene_Cg_evm.model.chr10B.90	gene_Car0360570
GO:0050482	arachidonic acid secretion	1/283	17/29370	0.1518061	0.3951117	0.3480485	1	gene_Cg_evm.model.chr10B.90	gene_Car0360570
GO:0004888	transmembrane signaling receptor activity	5/283	302/29370	0.1680519	0.4182472	0.3684283	5	gene_Cg_evm.model.chr7A.495/gene_Cg_evm.model.chr2A.556/gene_Cg_evm.model.chr8A.286/gene_Cg_evm.model.chr16A.546/gene_Cg_evm.model.chr20B.568	gene_Car0018980/gene_Car0156940/gene_Car0222990/gene_Car0232840/gene_Car0255870
GO:0016279	protein-lysine N-methyltransferase activity	1/283	19/29370	0.1680828	0.4182472	0.3684283	1	gene_Cg_evm.model.chr19A.771	gene_Car0252390
GO:0019205	nucleobase-containing compound kinase activity	1/283	19/29370	0.1680828	0.4182472	0.3684283	1	gene_Cg_evm.model.chr2A.892	gene_Car0155150
GO:0016641	oxidoreductase activity, acting on the CH-NH2 group of donors, oxygen as acceptor	1/283	20/29370	0.1761041	0.4182472	0.3684283	1	gene_Cg_evm.model.chr5A.397	gene_Car0040140
GO:0000278	mitotic cell cycle	1/283	20/29370	0.1761041	0.4182472	0.3684283	1	gene_Cg_evm.model.chr21A.406	gene_Car0412630
GO:0019882	antigen processing and presentation	1/283	20/29370	0.1761041	0.4182472	0.3684283	1	gene_Cg_evm.model.chr16B.911	gene_Car0135170
GO:0019904	protein domain specific binding	1/283	20/29370	0.1761041	0.4182472	0.3684283	1	gene_Cg_evm.model.chr3B.1540	gene_Car0013120
GO:0002224	toll-like receptor signaling pathway	1/283	21/29370	0.1840483	0.4284673	0.377431	1	gene_Cg_evm.model.chr16A.546	gene_Car0232840
GO:0043565	sequence-specific DNA binding	6/283	400/29370	0.1904943	0.4284673	0.377431	6	gene_Cg_evm.model.chr8B.771/gene_Cg_evm.model.chr16B.924/gene_Cg_evm.model.chr16A.745/gene_Cg_evm.model.chr20B.498/gene_Cg_evm.model.chr20B.352/gene_Cg_evm.model.chr11A.545	gene_Car0111080/gene_Car0135070/gene_Car0230790/gene_Car0256540/gene_Car0257990/gene_Car0393160
GO:0004143	ATP-dependent diacylglycerol kinase activity	1/283	22/29370	0.1919162	0.4284673	0.377431	1	gene_Cg_evm.model.chr4A.839	gene_Car0054230
GO:0007205	protein kinase C-activating G protein-coupled receptor signaling pathway	1/283	22/29370	0.1919162	0.4284673	0.377431	1	gene_Cg_evm.model.chr4A.839	gene_Car0054230

GO:0034968	histone lysine methylation	1/283	22/29370	0.1919162	0.4284673	0.377431	1	gene_Cg_evm.model.chr19A.771	gene_Car0252390
GO:0003746	translation elongation factor activity	1/283	23/29370	0.1997085	0.4284673	0.377431	1	gene_Cg_evm.model.chr20A.752	gene_Car0293370
GO:0045104	intermediate filament cytoskeleton organization	1/283	23/29370	0.1997085	0.4284673	0.377431	1	gene_Cg_evm.model.chr19A.320	gene_Car0248120
GO:0008654	phospholipid biosynthetic process	1/283	24/29370	0.2074259	0.4284673	0.377431	1	gene_Cg_evm.model.chr23A.377	gene_Car0377600
GO:0019229	regulation of vasoconstriction	1/283	24/29370	0.2074259	0.4284673	0.377431	1	gene_Cg_evm.model.chr1A.554	gene_Car0083430
GO:0008289	lipid binding	3/283	164/29370	0.2107134	0.4284673	0.377431	3	gene_Cg_evm.model.chr2A.298/gene_Cg_evm.model.chr17B.617/gene_Cg_evm.model .chr15A.511	gene_Car0159710/gene_Car0287050/gene_Car0344430
GO:0016746	acyltransferase activity	2/283	89/29370	0.2116971	0.4284673	0.377431	2	gene_Cg_evm.model.chr7B.774/gene_Cg_evm.model.chr16A.527	gene_Car0032420/gene_Car0233040
GO:0004721	phosphoprotein phosphatase activity	1/283	25/29370	0.2150691	0.4284673	0.377431	1	gene_Cg_evm.model.chr5B.675	gene_Car0072850
GO:0004983	neuropeptide Y receptor activity	1/283	25/29370	0.2150691	0.4284673	0.377431	1	gene_Cg_evm.model.chr1A.22	gene_Car0078120
GO:0005044	scavenger receptor activity	2/283	91/29370	0.2187604	0.4284673	0.377431	2	gene_Cg_evm.model.chr5A.397/gene_Cg_evm.model.chr18A.664	gene_Car0040140/gene_Car0163410
GO:0005520	insulin-like growth factor binding	1/283	26/29370	0.2226389	0.4284673	0.377431	1	gene_Cg_evm.model.chr20B.217	gene_Car0259460
GO:0006414	translational elongation	1/283	26/29370	0.2226389	0.4284673	0.377431	1	gene_Cg_evm.model.chr20A.752	gene_Car0293370
GO:0030145	manganese ion binding	1/283	26/29370	0.2226389	0.4284673	0.377431	1	gene_Cg_evm.model.chr5B.675	gene_Car0072850
GO:0042803	protein homodimerization activity	1/283	26/29370	0.2226389	0.4284673	0.377431	1	gene_Cg_evm.model.chr4B.56	gene_Car0177610
GO:0020037	heme binding	3/283	169/29370	0.223254	0.4284673	0.377431	3	gene_Cg_evm.model.chr5B.223/gene_Cg_evm.model.chr20B.331/gene_Cg_evm.model. chr12B.576	gene_Car0068230/gene_Car0258220/gene_Car0333800
GO:0000145	exocyst	1/283	27/29370	0.2301359	0.4372583	0.3851749	1	gene_Cg_evm.model.chr5B.469	gene_Car0070610
GO:0005452	solute:inorganic anion antiporter activity	1/283	28/29370	0.2375609	0.4382192	0.3860213	1	gene_Cg_evm.model.chr14B.768	gene_Car0195140
GO:0016788	hydrolase activity, acting on ester bonds	1/283	28/29370	0.2375609	0.4382192	0.3860213	1	gene_Cg_evm.model.chr12A.452	gene_Car0327800
GO:0043066	negative regulation of apoptotic process	1/283	28/29370	0.2375609	0.4382192	0.3860213	1	gene_Cg_evm.model.chr11A.371	gene_Car0391290
GO:0006820	monoatomic anion transport	1/283	29/29370	0.2449146	0.4389978	0.3867072	1	gene_Cg_evm.model.chr14B.768	gene_Car0195140
GO:0007034	vacuolar transport	1/283	29/29370	0.2449146	0.4389978	0.3867072	1	gene_Cg_evm.model.chr1B.950	gene_Car0065120
GO:0062023	collagen-containing extracellular matrix	1/283	29/29370	0.2449146	0.4389978	0.3867072	1	gene_Cg_evm.model.chr1B.967	gene_Car0065180
GO:0016042	lipid catabolic process	1/283	30/29370	0.2521975	0.4478274	0.394485	1	gene_Cg_evm.model.chr17B.785	gene_Car0285300
GO:0004869	cysteine-type endopeptidase inhibitor activity	1/283	31/29370	0.2594105	0.451587	0.3977969	1	gene_Cg_evm.model.chr13B.592	gene_Car0209000
GO:0051056	regulation of small GTPase mediated signal transduction	1/283	31/29370	0.2594105	0.451587	0.3977969	1	gene_Cg_evm.model.chr5A.512	gene_Car0041400
GO:0008146	sulfotransferase activity	2/283	103/29370	0.2614451	0.451587	0.3977969	2	gene_Cg_evm.model.chr8B.76/gene_Cg_evm.model.chr2B.292	gene_Car0103720/gene_Car0131320
GO:0006334	nucleosome assembly	1/283	32/29370	0.2665541	0.45219	0.398328	1	gene_Cg_evm.model.chr6B.611	gene_Car0188230

GO:0030001	metal ion transport	1/283	32/29370	0.2665541	0.45219	0.398328	1	gene_Cg_evm.model.chr1B.950	gene_Car0065120
GO:0004089	carbonate dehydratase activity	1/283	33/29370	0.2736291	0.4560484	0.4017269	1	gene_Cg_evm.model.chr3B.1078	gene_Car0008910
GO:0042157	lipoprotein metabolic process	1/283	33/29370	0.2736291	0.4560484	0.4017269	1	gene_Cg_evm.model.chr2A.298	gene_Car0159710
GO:0007156	homophilic cell adhesion via plasma membrane adhesion molecules	3/283	190/29370	0.2772538	0.4580715	0.4035089	3	gene_Cg_evm.model.chr1B.806/gene_Cg_evm.model.chr14B.803/gene_Cg_evm.model.chr14A.84	gene_Car0063800/gene_Car0194780/gene_Car0283260
GO:0031418	L-ascorbic acid binding	1/283	35/29370	0.2875756	0.4670031	0.4113767	1	gene_Cg_evm.model.chr6B.608	gene_Car0188270
GO:0046856	phosphatidylinositol dephosphorylation	1/283	35/29370	0.2875756	0.4670031	0.4113767	1	gene_Cg_evm.model.chr13A.822	gene_Car0245020
GO:0007411	axon guidance	1/283	37/29370	0.3012553	0.4809959	0.4237027	1	gene_Cg_evm.model.chr6A.647	gene_Car0146530
GO:0042054	histone methyltransferase activity	1/283	37/29370	0.3012553	0.4809959	0.4237027	1	gene_Cg_evm.model.chr19A.771	gene_Car0252390
GO:0006887	exocytosis	1/283	38/29370	0.3079967	0.4836311	0.4260241	1	gene_Cg_evm.model.chr5B.469	gene_Car0070610
GO:0030286	dynein complex	1/283	38/29370	0.3079967	0.4836311	0.4260241	1	gene_Cg_evm.model.chr15A.228	gene_Car0341740
GO:0008092	cytoskeletal protein binding	1/283	39/29370	0.3146732	0.4900649	0.4316915	1	gene_Cg_evm.model.chr20A.66	gene_Car0300570
GO:0004950	chemokine receptor activity	1/283	41/29370	0.3278344	0.5023269	0.442493	1	gene_Cg_evm.model.chr9B.581	gene_Car0098030
GO:0008233	peptidase activity	1/283	41/29370	0.3278344	0.5023269	0.442493	1	gene_Cg_evm.model.chr12B.395	gene_Car0335550
GO:0043087	regulation of GTPase activity	1/283	42/29370	0.3343202	0.5081668	0.4476372	1	gene_Cg_evm.model.chr23B.575	gene_Car0264680
GO:0005886	plasma membrane	6/283	496/29370	0.3448394	0.5192915	0.4574369	6	gene_Cg_evm.model.chr1B.806/gene_Cg_evm.model.chr1B.967/gene_Cg_evm.model.chr6A.647/gene_Cg_evm.model.chr14B.803/gene_Cg_evm.model.chr14A.84/gene_Cg_evm.model.chr25B.541	gene_Car0063800/gene_Car0065180/gene_Car0146530/gene_Car0194780/gene_Car0283260/gene_Car0403770
GO:0005242	inward rectifier potassium channel activity	1/283	44/29370	0.3471054	0.5192915	0.4574369	1	gene_Cg_evm.model.chr12A.474	gene_Car0327610
GO:0000723	telomere maintenance	1/283	46/29370	0.3596459	0.5338493	0.4702606	1	gene_Cg_evm.model.chr4B.56	gene_Car0177610
GO:0008237	metallopeptidase activity	3/283	223/29370	0.3638512	0.5359049	0.4720714	3	gene_Cg_evm.model.chr15B.793/gene_Cg_evm.model.chr12B.612/gene_Cg_evm.model.chr12B.395	gene_Car0324670/gene_Car0333400/gene_Car0335550
GO:0005164	tumor necrosis factor receptor binding	1/283	48/29370	0.3719463	0.5436138	0.478862	1	gene_Cg_evm.model.chr3A.838	gene_Car0121280
GO:0005743	mitochondrial inner membrane	1/283	49/29370	0.3780079	0.5482558	0.4829511	1	gene_Cg_evm.model.chr3A.1011	gene_Car0122910
GO:0006139	nucleobase-containing compound metabolic process	1/283	51/29370	0.3899568	0.5530191	0.487147	1	gene_Cg_evm.model.chr2A.892	gene_Car0155150
GO:0006644	phospholipid metabolic process	1/283	51/29370	0.3899568	0.5530191	0.487147	1	gene_Cg_evm.model.chr10B.90	gene_Car0360570
GO:0016192	vesicle-mediated transport	3/283	234/29370	0.3925174	0.5530191	0.487147	3	gene_Cg_evm.model.chr6A.121/gene_Cg_evm.model.chr6B.123/gene_Cg_evm.model.chr19B.280	gene_Car0152110/gene_Car0193370/gene_Car0214310
GO:0016567	protein ubiquitination	2/283	141/29370	0.3947029	0.5530191	0.487147	2	gene_Cg_evm.model.chr5B.544/gene_Cg_evm.model.chr16A.776	gene_Car0071470/gene_Car0230470
GO:0007017	microtubule-based process	1/283	52/29370	0.3958453	0.5530191	0.487147	1	gene_Cg_evm.model.chr15A.228	gene_Car0341740
GO:0005507	copper ion binding	1/283	53/29370	0.401677	0.5570703	0.4907157	1	gene_Cg_evm.model.chr5A.397	gene_Car0040140
GO:0006888	endoplasmic reticulum to Golgi vesicle-mediated transport	1/283	55/29370	0.4131728	0.563046	0.4959796	1	gene_Cg_evm.model.chr7A.161	gene_Car0015650
GO:0007219	Notch signaling pathway	1/283	55/29370	0.4131728	0.563046	0.4959796	1	gene_Cg_evm.model.chr5B.544	gene_Car0071470

GO:0000287	magnesium ion binding	2/283	147/29370	0.414876	0.563046	0.4959796	2	gene_Cg_evm.model.chr24B.346/gene_Cg_evm.model.chr24A.302	gene_Car0349630/gene_Car0384640
GO:0030414	peptidase inhibitor activity	1/283	58/29370	0.4300051	0.5794395	0.5104204	1	gene_Cg_evm.model.chr16A.290	gene_Car0235160
GO:0051726	regulation of cell cycle	1/283	62/29370	0.4517021	0.6029815	0.5311582	1	gene_Cg_evm.model.chr11A.371	gene_Car0391290
GO:0016747	acyltransferase activity, transferring groups other than amino-acyl groups	1/283	63/29370	0.4569965	0.6029815	0.5311582	1	gene_Cg_evm.model.chr5A.54	gene_Car0036460
GO:0030527	structural constituent of chromatin	1/283	63/29370	0.4569965	0.6029815	0.5311582	1	gene_Cg_evm.model.chr6B.611	gene_Car0188230
GO:0006915	apoptotic process	1/283	65/29370	0.4674329	0.6104444	0.5377322	1	gene_Cg_evm.model.chr16A.340	gene_Car0234670
GO:0000981	DNA-binding transcription factor activity, RNA polymerase II-specific	5/283	467/29370	0.4690783	0.6104444	0.5377322	5	gene_Cg_evm.model.chr3A.247/gene_Cg_evm.model.chr3A.250/gene_Cg_evm.model.chr14B.748/gene_Cg_evm.model.chr13A.699/gene_Cg_evm.model.chr19A.216	gene_Car0115460/gene_Car0115480/gene_Car0195320/gene_Car0243810/gene_Car0247170
GO:0043547	positive regulation of GTPase activity	1/283	66/29370	0.472576	0.6108125	0.5380564	1	gene_Cg_evm.model.chr24A.136	gene_Car0382910
GO:0006869	lipid transport	1/283	67/29370	0.4776695	0.6117974	0.538924	1	gene_Cg_evm.model.chr2A.298	gene_Car0159710
GO:0005667	transcription regulator complex	1/283	68/29370	0.482714	0.6117974	0.538924	1	gene_Cg_evm.model.chr12B.327	gene_Car0336210
GO:0005856	cytoskeleton	2/283	171/29370	0.4919278	0.6117974	0.538924	2	gene_Cg_evm.model.chr20A.66/gene_Cg_evm.model.chr12B.214	gene_Car0300570/gene_Car0337280
GO:0000786	nucleosome	1/283	70/29370	0.4926579	0.6117974	0.538924	1	gene_Cg_evm.model.chr6B.611	gene_Car0188230
GO:0006935	chemotaxis	1/283	70/29370	0.4926579	0.6117974	0.538924	1	gene_Cg_evm.model.chr9B.581	gene_Car0098030
GO:0030215	semaphorin receptor binding	1/283	70/29370	0.4926579	0.6117974	0.538924	1	gene_Cg_evm.model.chr19A.716	gene_Car0252980
GO:0004879	nuclear receptor activity	1/283	72/29370	0.5024113	0.619858	0.5460245	1	gene_Cg_evm.model.chr16B.924	gene_Car0135070
GO:0007601	visual perception	1/283	74/29370	0.5119778	0.625026	0.5505769	1	gene_Cg_evm.model.chr12B.134	gene_Car0338060
GO:0005096	GTPase activator activity	2/283	178/29370	0.5131792	0.625026	0.5505769	2	gene_Cg_evm.model.chr5A.512/gene_Cg_evm.model.chr24A.136	gene_Car0041400/gene_Car0382910
GO:0006260	DNA replication	1/283	75/29370	0.5166921	0.6252961	0.5508149	1	gene_Cg_evm.model.chr25A.353	gene_Car0419230
GO:0005198	structural molecule activity	2/283	182/29370	0.5250581	0.6285355	0.5536684	2	gene_Cg_evm.model.chr20A.66/gene_Cg_evm.model.chr11B.573	gene_Car0300570/gene_Car0400480
GO:0005516	calmodulin binding	1/283	77/29370	0.525985	0.6285355	0.5536684	1	gene_Cg_evm.model.chr2A.528	gene_Car0157260
GO:0005922	connexin complex	1/283	78/29370	0.5305644	0.6294417	0.5544667	1	gene_Cg_evm.model.chr9B.170	gene_Car0101930
GO:0006886	intracellular protein transport	3/283	291/29370	0.533369	0.6294417	0.5544667	3	gene_Cg_evm.model.chr3B.1124/gene_Cg_evm.model.chr4B.7/gene_Cg_evm.model.chr24B.647	gene_Car0009340/gene_Car0177740/gene_Car0352580
GO:0140359	ABC-type transporter activity	1/283	83/29370	0.5528089	0.6483561	0.5711281	1	gene_Cg_evm.model.chr18A.601	gene_Car0163840
GO:0016055	Wnt signaling pathway	1/283	88/29370	0.5740028	0.669083	0.5893861	1	gene_Cg_evm.model.chr6B.714	gene_Car0187170
GO:0005739	mitochondrion	1/283	90/29370	0.5821973	0.6744969	0.5941552	1	gene_Cg_evm.model.chr17A.383	gene_Car0313340
GO:0004867	serine-type endopeptidase inhibitor activity	1/283	94/29370	0.5981182	0.6887422	0.6067036	1	gene_Cg_evm.model.chr4A.478	gene_Car0050850
GO:0005783	endoplasmic reticulum	1/283	96/29370	0.6058505	0.6934433	0.6108448	1	gene_Cg_evm.model.chr4A.948	gene_Car0055190
GO:0003774	cytoskeletal motor activity	1/283	101/29370	0.6245387	0.710553	0.6259165	1	gene_Cg_evm.model.chr11A.333	gene_Car0390910
GO:0016757	glycosyltransferase activity	1/283	106/29370	0.6423438	0.7264602	0.639929	1	gene_Cg_evm.model.chr15B.577	gene_Car0322500
GO:0005102	signaling receptor binding	1/283	110/29370	0.6559801	0.7374924	0.649647	1	gene_Cg_evm.model.chr6B.714	gene_Car0187170

GO:0005737	cytoplasm	4/283	468/29370	0.6625684	0.7405176	0.6523119	4	gene_Cg_evm.model.chr20B.94/gene_Cg_evm.model.chr12A.41/gene_Cg_evm.model.chr12B.327/gene_Cg_evm.model.chr24A.136	gene_Car0260600/gene_Car0331730/gene_Car0336210/gene_Car0382910
GO:0051015	actin filament binding	1/283	115/29370	0.6722991	0.746999	0.6580213	1	gene_Cg_evm.model.chr11A.50	gene_Car0388110
GO:0016459	myosin complex	1/283	118/29370	0.6817178	0.7530204	0.6633254	1	gene_Cg_evm.model.chr11A.333	gene_Car0390910
GO:0006813	potassium ion transport	2/283	245/29370	0.6856449	0.7530204	0.6633254	2	gene_Cg_evm.model.chr2A.528/gene_Cg_evm.model.chr12A.474	gene_Car0157260/gene_Car0327610
GO:0016787	hydrolase activity	2/283	248/29370	0.6920573	0.7556948	0.6656813	2	gene_Cg_evm.model.chr5B.675/gene_Cg_evm.model.chr10B.453	gene_Car0072850/gene_Car0364080
GO:0007154	cell communication	1/283	124/29370	0.6997536	0.7597325	0.669238	1	gene_Cg_evm.model.chr9B.170	gene_Car0101930
GO:0038023	signaling receptor activity	1/283	131/29370	0.719512	0.7767459	0.6842249	1	gene_Cg_evm.model.chr10B.625	gene_Car0365800
GO:0042981	regulation of apoptotic process	1/283	143/29370	0.7504157	0.805531	0.7095813	1	gene_Cg_evm.model.chr16A.340	gene_Car0234670
GO:0005249	voltage-gated potassium channel activity	1/283	152/29370	0.7713449	0.818746	0.7212222	1	gene_Cg_evm.model.chr15B.731	gene_Car0324050
GO:0006281	DNA repair	1/283	152/29370	0.7713449	0.818746	0.7212222	1	gene_Cg_evm.model.chr25B.456	gene_Car0404610
GO:0016791	phosphatase activity	2/283	293/29370	0.775857	0.8189602	0.7214109	2	gene_Cg_evm.model.chr3B.791/gene_Cg_evm.model.chr13A.822	gene_Car0006250/gene_Car0245020
GO:0007275	multicellular organism development	1/283	163/29370	0.7945643	0.834073	0.7347236	1	gene_Cg_evm.model.chr6B.714	gene_Car0187170
GO:0006357	regulation of transcription by RNA polymerase II	2/283	319/29370	0.81463	0.8504379	0.7491392	2	gene_Cg_evm.model.chr14B.696/gene_Cg_evm.model.chr16A.745	gene_Car0195820/gene_Car0230790
GO:0004222	metalloendopeptidase activity	1/283	190/29370	0.8420744	0.8742849	0.7701457	1	gene_Cg_evm.model.chr15B.793	gene_Car0324670
GO:0016491	oxidoreductase activity	2/283	356/29370	0.8594969	0.8793841	0.7746375	2	gene_Cg_evm.model.chr7B.582/gene_Cg_evm.model.chr17A.383	gene_Car0030650/gene_Car0313340
GO:0008017	microtubule binding	1/283	203/29370	0.8608708	0.8793841	0.7746375	1	gene_Cg_evm.model.chr19A.320	gene_Car0248120
GO:0008083	growth factor activity	1/283	203/29370	0.8608708	0.8793841	0.7746375	1	gene_Cg_evm.model.chr25B.197	gene_Car0407300
GO:0004842	ubiquitin-protein transferase activity	1/283	232/29370	0.8951518	0.9095126	0.8011773	1	gene_Cg_evm.model.chr16A.776	gene_Car0230470
GO:0046983	protein dimerization activity	2/283	403/29370	0.9021661	0.9117636	0.8031602	2	gene_Cg_evm.model.chr23B.559/gene_Cg_evm.model.chr12B.327	gene_Car0264850/gene_Car0336210
GO:0005216	monoatomic ion channel activity	2/283	466/29370	0.9406415	0.9456185	0.8329825	2	gene_Cg_evm.model.chr11A.243/gene_Cg_evm.model.chr25B.541	gene_Car0389960/gene_Car0403770
GO:0016887	ATP hydrolysis activity	1/283	333/29370	0.9609414	0.9609414	0.8464803	1	gene_Cg_evm.model.chr18A.601	gene_Car0163840

Supplementary Table 12. Summary statistics of the MGI sequencing data.

Platform	Group	Samples	Raw_Base (Gb)	Clean_Base (Gb)	Clean_Base_Percent (%)	>Q20 (%)	>Q30 (%)
MGI (DNBSEQ-T7)	Extremely resistant group	R-1	62.09	62.02	99.88	97.67	92.43
		R-2	63.35	63.23	99.81	97.99	93.46
		R-3	60.41	60.30	99.81	97.5	91.79
		R-4	61.51	61.38	99.79	98.05	93.36
		R-5	61.67	61.53	99.78	97.27	91.13
		R-6	60.30	60.20	99.84	97.64	92.18
		R-7	61.94	61.83	99.82	97.41	91.5
		R-8	64.23	64.13	99.84	97.49	91.72
		R-9	59.12	59.04	99.86	97.89	93.15
		R-10	61.84	61.70	99.77	98.05	93.46
		R-11	83.85	83.72	99.84	98.19	94.08
		R-12	54.58	54.44	99.74	97.79	92.74
		R-13	59.40	59.26	99.76	97.71	92.37
		R-14	64.31	64.17	99.78	97.17	91.32
		R-15	62.87	62.76	99.82	97.29	91.54
		R-16	63.13	63.05	99.87	97.86	93.07
		R-17	59.49	59.40	99.85	97.64	92.32
		R-18	59.71	59.63	99.86	97.67	92.48
		R-19	65.64	65.51	99.8	97.48	92.23
		R-20	69.15	69.02	99.81	97.2	91.34
		R-21	63.63	63.49	99.78	97.07	90.92
		R-22	81.51	81.36	99.82	97.92	93.47
		R-23	60.44	60.32	99.8	97.09	90.95
		R-24	66.57	66.42	99.78	97.08	90.96
		R-25	57.18	57.06	99.79	97.22	91.5
		R-26	58.80	58.69	99.81	96.78	90.24
		R-27	58.14	58.04	99.83	97.07	91.12
		R-28	56.01	55.91	99.82	97.15	91.2
		R-29	59.51	59.40	99.81	97.09	91.06
		R-30	53.45	53.34	99.79	97.15	91.27
	Extremely susceptible group	S-1	58.71	58.60	99.81	97.59	91.97
		S-2	62.76	62.64	99.81	98.71	95.34
		S-3	59.46	59.35	99.82	97.83	92.83
		S-4	61.57	61.46	99.82	98.59	94.93
		S-5	59.69	59.58	99.82	97.83	92.86
		S-6	64.21	64.08	99.8	97.91	93.08
		S-7	63.67	63.54	99.79	98.06	92.97
		S-8	61.93	61.87	99.91	98.86	95.93
		S-9	63.82	63.76	99.91	98.79	95.65
		S-10	63.25	63.16	99.86	98.77	95.56
		S-11	55.46	55.38	99.85	98.54	94.89
		S-12	56.34	56.24	99.82	97.9	92.9
		S-13	61.83	61.74	99.85	98.46	94.68
		S-14	78.72	78.59	99.84	98.31	94.38
		S-15	61.19	61.09	99.84	96.8	90
		S-16	56.44	56.32	99.79	97.4	91.69
		S-17	60.02	59.92	99.84	97.44	91.94
		S-18	61.81	61.71	99.84	97.16	91.05
		S-19	59.41	59.32	99.85	97.93	92.76
		S-20	63.43	63.34	99.86	98.45	94.73
		S-21	61.56	61.44	99.8	97.22	91.19
		S-22	63.78	63.69	99.86	96.83	89.77
		S-23	59.17	59.07	99.83	96.78	89.92
		S-24	62.39	62.28	99.82	98.17	93.61
		S-25	62.40	62.31	99.86	98.45	94.65
		S-26	61.37	61.28	99.85	98.23	93.93
		S-27	64.15	64.03	99.81	96.99	90.5
		S-28	62.82	62.69	99.79	97.27	91.37
		S-29	60.52	60.41	99.82	97.42	91.56
		S-30	62.06	61.95	99.83	97.6	92.11

Supplementary Table 13. The harbored 292 genes of the hotspot region.

Gene_id	Start	End	Gene_name	NR_annotation	SwissProt_annotation	TrEMBL_annotation
gene_Car0333590	5019767	5043658	ipmkb	inositol polyphosphate multikinase-like [Carassius auratus]	Inositol polyphosphate multikinase OS=Homo sapiens OX=9606 GN=IPMK PE=1 SV=1	Kinase OS=Carassius auratus OX=7957 GN=LOC113111942 PE=3 SV=1
gene_Car0333600	5044444	5051222	ube2d1bB	ubiquitin-conjugating enzyme E2 D1b [Danio rerio]	Ubiquitin-conjugating enzyme E2 D4 OS=Homo sapiens OX=9606 GN=UBE2D4 PE=1 SV=1	Ubiquitin-conjugating enzyme E2 D1 OS=Astyanax mexicanus OX=7994 GN=ube2d1 PE=3 SV=1
gene_Car0333610	5053900	5058706	tfamB	transcription factor A, mitochondrial-like [Carassius auratus]	Transcription factor A, mitochondrial OS=Sus scrofa OX=9823 GN=TFAM PE=2 SV=1	transcription factor A, mitochondrial-like OS=Carassius auratus OX=7957 GN=LOC113111943 PE=4 SV=1
gene_Car0333620	5057278	5063459	zgc:171971B	DNA-directed RNA polymerase III subunit RPC4-like [Carassius auratus]	DNA-directed RNA polymerase III subunit RPC4 OS=Mus musculus OX=10090 GN=Polr3d PE=2 SV=2	DNA-directed RNA polymerase III subunit RPC4-like OS=Carassius auratus OX=7957 GN=LOC113111940 PE=4 SV=1
gene_Car0333630	5065026	5133513	bicc1bB	protein bicaudal C homolog 1 [Carassius gibelio]	Protein bicaudal C homolog 1-B OS=Xenopus laevis OX=8355 GN=bicc1-b PE=2 SV=1	protein bicaudal C homolog 1-like OS=Carassius auratus OX=7957 GN=LOC113111939 PE=3 SV=1
gene_Car0333640	5149644	5176513	phyhiplbB	phytanoyl-CoA hydroxylase-interacting protein-like isoform X1 [Carassius auratus]	Phytanoyl-CoA hydroxylase-interacting protein-like OS=Danio rerio OX=7955 GN=phyhipl PE=2 SV=1	phytanoyl-CoA hydroxylase-interacting protein-like isoform X1 OS=Carassius auratus OX=7957 GN=LOC113111938 PE=3 SV=1
gene_Car0333650	5178201	5191366	si:ch73-51i5B	protein FAM13C-like [Carassius auratus]	Protein FAM13C OS=Homo sapiens OX=9606 GN=FAM13C PE=1 SV=2	protein FAM13C-like OS=Carassius auratus OX=7957 GN=LOC113111937 PE=3 SV=1
gene_Car0333660	5211423	5220052	slc16a9bB	monocarboxylate transporter 9-like [Carassius auratus]	Monocarboxylate transporter 9 OS=Homo sapiens OX=9606 GN=SLC16A9 PE=1 SV=1	monocarboxylate transporter 9-like OS=Carassius auratus OX=7957 GN=LOC113111935 PE=4 SV=1
gene_Car0333670	5220987	5250985	ccdc6bB	coiled-coil domain-containing protein 6 [Cyprinus carpio]	Coiled-coil domain-containing protein 6 OS=Mus musculus OX=10090 GN=Ccdc6 PE=1 SV=1	Coiled-coil domain containing 6b OS=Cyprinus carpio OX=7962 PE=4 SV=1
gene_Car0333680	5252974	5253192	NA	--	--	--
gene_Car0333690	5256447	5342225	ank3bB	ankyrin-3-like isoform X30 [Cyprinus carpio]	Ankyrin-3 OS=Homo sapiens OX=9606 GN=ANK3 PE=1 SV=3	Ankyrin-3 (Fragment) OS=Anabarilius grahami OX=495550 GN=DPX16_22864 PE=4 SV=1
gene_Car0333700	5453768	5453953	NA	PREDICTED: rho-related BTB domain-containing protein 1-like [Sinocyclocheilus rhinocerous]	--	--
gene_Car0333710	5471654	5486675	rhobtb1B	rho-related BTB domain-containing protein 1-like isoform X1 [Carassius auratus]	Rho-related BTB domain-containing protein 1 OS=Homo sapiens OX=9606 GN=RHOBTB1 PE=1 SV=2	rho-related BTB domain-containing protein 1-like isoform X1 OS=Carassius auratus OX=7957 GN=LOC113111930 PE=4 SV=1
gene_Car0333720	5498902	5502399	tmem26b	transmembrane protein 26b [Carassius gibelio]	Transmembrane protein 26 OS=Homo sapiens OX=9606 GN=TMEM26 PE=1 SV=1	transmembrane protein 26-like OS=Carassius auratus OX=7957 GN=LOC113111929 PE=4 SV=1
gene_Car0333730	5514681	5568921	NA	AT-rich interactive domain-containing protein 5B-like [Carassius auratus]	AT-rich interactive domain-containing protein 5B OS=Danio rerio OX=7955 GN=arid5b PE=3 SV=1	AT-rich interactive domain-containing protein 5B OS=Cyprinus carpio OX=7962 PE=4 SV=1
gene_Car0333740	5593790	5669423	arid5bB	AT-rich interactive domain-containing protein 5B-like [Carassius auratus]	AT-rich interactive domain-containing protein 5B OS=Danio rerio OX=7955 GN=arid5b PE=3 SV=1	AT-rich interactive domain-containing protein 5B OS=Carassius auratus OX=7957 GN=LOC113111928 PE=3 SV=1
gene_Car0333750	5683079	5684438	adoa	2-aminoethanethiol dioxygenase-like [Carassius auratus]	2-aminoethanethiol dioxygenase OS=Mus musculus OX=10090 GN=Ado PE=1 SV=2	2-aminoethanethiol dioxygenase-like OS=Carassius auratus OX=7957 GN=LOC113111927 PE=4 SV=1
gene_Car0333760	5686163	5688402	egr2bB	early growth response protein 2b-like isoform X1 [Carassius auratus]	Early growth response protein 2b OS=Danio rerio OX=7955 GN=egr2b PE=2 SV=1	early growth response protein 2b-like isoform X1 OS=Carassius auratus OX=7957 GN=LOC113111926 PE=3 SV=1
gene_Car0333770	5721564	5731457	nrbf2bB	nuclear receptor-binding factor 2-like [Carassius auratus]	Nuclear receptor-binding factor 2 OS=Homo sapiens OX=9606 GN=NRBF2 PE=1 SV=1	nuclear receptor-binding factor 2-like OS=Carassius auratus OX=7957 GN=LOC113111925 PE=4 SV=1
gene_Car0333780	5726686	5858554	jmjd1cbB	probable JmjC domain-containing histone demethylation protein 2C isoform X2 [Carassius auratus]	Probable JmjC domain-containing histone demethylation protein 2C OS=Homo sapiens OX=9606 GN=JMJD1C PE=1 SV=2	Lysine-specific demethylase OS=Carassius auratus OX=7957 GN=LOC113111924 PE=3 SV=1
gene_Car0333790	5860676	5889171	reep3bB	receptor expression-enhancing protein 3 isoform X1 [Carassius auratus]	Receptor expression-enhancing protein 3 OS=Danio rerio OX=7955 GN=reep3 PE=2 SV=1	Receptor expression-enhancing protein OS=Carassius auratus OX=7957 GN=LOC113111923 PE=3 SV=1
gene_Car0333800	5913612	5916160	cyp26a1B	cytochrome P450 26A1 [Carassius auratus]	Cytochrome P450 26A1 OS=Danio rerio OX=7955 GN=cyp26a1 PE=1 SV=1	cytochrome P450 26A1 OS=Carassius auratus OX=7957 GN=LOC113111922 PE=3 SV=1
gene_Car0333810	5922193	5977865	exoc6B	exocyst complex component 6-like isoform X5 [Carassius auratus]	Exocyst complex component 6 OS=Homo sapiens OX=9606 GN=EXOC6 PE=1 SV=3	Exocyst complex component OS=Carassius auratus OX=7957 GN=LOC113111920 PE=3 SV=1
gene_Car0333820	6010326	6011600	LOC101884976B	QRFP-like peptide receptor [Carassius auratus]	Neuropeptide SIFamide receptor OS=Drosophila melanogaster OX=7227 GN=SIFaR PE=2 SV=2	QRFP-like peptide receptor OS=Carassius auratus OX=7957 GN=LOC113112182 PE=3 SV=1
gene_Car0333830	6018115	6019649	NA	uncharacterized protein LOC113118736 [Carassius auratus]	--	uncharacterized protein LOC113118736 OS=Carassius auratus OX=7957 GN=LOC113118736 PE=4 SV=1
gene_Car0333840	6023292	6030606	tubgcp2B	Gamma-tubulin complex component 2 [Labeo rohita]	Gamma-tubulin complex component 2 OS=Mus musculus OX=10090 GN=Tubgcp2 PE=1 SV=2	Gamma-tubulin complex component OS=Anabarilius grahami OX=495550 GN=DPX16_22848 PE=3 SV=1
gene_Car0333850	6033988	6035608	NA	gamma-tubulin complex component 2 [Puntigrus tetrazona]	--	Gamma-tubulin complex component OS=Sinocyclocheilus anshuiensis OX=1608454 GN=LOC107684770 PE=3 SV=1
gene_Car0333860	6039975	6064889	myofB	myoferlin-like isoform X2 [Carassius auratus]	Myoferlin OS=Homo sapiens OX=9606 GN=MYOF PE=1 SV=1	myoferlin-like isoform X2 OS=Carassius auratus OX=7957 GN=LOC113111919 PE=3 SV=1
gene_Car0333870	6072960	6107632	zgc:152977B	SUN domain-containing protein 1 isoform X2 [Carassius gibelio]	SUN domain-containing protein 1 OS=Mus musculus OX=10090 GN=Sun1 PE=1 SV=2	SUN domain-containing protein 1-like isoform X2 OS=Carassius auratus OX=7957 GN=LOC113111918 PE=4 SV=1
gene_Car0333880	6118758	6122968	crym	ketimine reductase mu-crystallin [Carassius gibelio]	Ketimine reductase mu-crystallin OS=Mus musculus OX=10090 GN=Crym PE=1 SV=1	Ketimine reductase mu-crystallin OS=Carassius auratus OX=7957 GN=crym PE=4 SV=1
gene_Car0333890	6123690	6125337	snu13bB	NHP2-like protein 1 [Carassius auratus]	NHP2-like protein 1 OS=Xenopus laevis OX=8355 GN=snu13 PE=2 SV=1	Ribonucleoprotein OS=Carassius auratus OX=7957 GN=LOC113111915 PE=3 SV=1

gene_Car0333900	6123690	6138870	uqcrc2bB	cytochrome b-c1 complex subunit 2, mitochondrial [Carassius gibelio]	Cytochrome b-c1 complex subunit 2, mitochondrial OS=Homo sapiens OX=9606 GN=UQCRC2 PE=1 SV=3	cytochrome b-c1 complex subunit 2, mitochondrial-like OS=Carassius auratus OX=7957 GN=LOC113111914 PE=3 SV=1
gene_Car0333910	6139613	6146643	zgc:153595B	modulator of smoothened protein [Puntigrus tetrazona]	Modulator of smoothened protein OS=Danio rerio OX=7955 GN=mosmob PE=2 SV=1	Modulator of smoothened OS=Cyprinus carpio OX=7962 GN=mosmo PE=4 SV=1
gene_Car0333920	6154109	6170912	eef2kB	eukaryotic elongation factor 2 kinase isoform X4 [Carassius gibelio]	Eukaryotic elongation factor 2 kinase OS=Homo sapiens OX=9606 GN=EEF2K PE=1 SV=2	Eukaryotic elongation factor 2 kinase OS=Carassius auratus OX=7957 GN=LOC113111911 PE=3 SV=1
gene_Car0333930	6171644	6183202	mki67B	proliferation marker protein Ki-67-like isoform X1 [Carassius auratus]	Proliferation marker protein Ki-67 OS=Mus musculus OX=10090 GN=Mki67 PE=1 SV=1	proliferation marker protein Ki-67-like isoform X1 OS=Carassius auratus OX=7957 GN=LOC113111910 PE=4 SV=1
gene_Car0333940	6200083	6202025	pyybB	peptide Y-like [Carassius auratus]	Peptide Y OS=Dicentrarchus labrax OX=13489 PE=3 SV=1	peptide Y-like OS=Carassius auratus OX=7957 GN=LOC113112238 PE=3 SV=1
gene_Car0333950	6203094	6429551	mpp2bB	MAGUK p55 subfamily member 2-like isoform X3 [Carassius gibelio]	MAGUK p55 subfamily member 2 OS=Rattus norvegicus OX=10116 GN=Mpp2 PE=1 SV=2	Membrane protein, palmitoylated 2b (MAGUK p55 subfamily member 2) OS=Cyprinus carpio OX=7962 PE=3 SV=1
gene_Car0333960	6431534	6453983	psmc5B	band 3 anion exchange -like protein [Labeo rohita]	26S proteasome regulatory subunit 8 OS=Bos taurus OX=9913 GN=PSMC5 PE=2 SV=1	Ataxin-7-like protein 3 OS=Labeo rohita OX=84645 GN=ATXN7L3 PE=3 SV=1
gene_Car0333970	6455386	6477880	si:dkeyp-118b1B	ataxin-7-like protein 3 isoform X1 [Carassius auratus]	Ataxin-7-like protein 3 OS=Danio rerio OX=7955 GN=atxn7l3 PE=2 SV=1	Ataxin-7-like protein 3 OS=Carassius auratus OX=7957 GN=LOC113111905 PE=3 SV=1
gene_Car0333980	6477976	6494589	slc4a1bB	band 3 anion exchange protein-like isoform X1 [Carassius auratus]	Band 3 anion exchange protein OS=Oncorhynchus mykiss OX=8022 GN=slc4a1 PE=2 SV=2	Anion exchange protein OS=Carassius auratus OX=7957 GN=LOC113111903 PE=3 SV=1
gene_Car0333990	6499147	6546193	ngfrbB	tumor necrosis factor receptor superfamily member 16 [Carassius gibelio]	Tumor necrosis factor receptor superfamily member 16 OS=Homo sapiens OX=9606 GN=NGFR PE=1 SV=1	tumor necrosis factor receptor superfamily member 16-like isoform X1 OS=Carassius auratus OX=7957 GN=LOC113111904 PE=4 SV=1
gene_Car0334000	6592635	6594333	ndufa4	cytochrome c oxidase subunit NDUFA4 [Puntigrus tetrazona]	Cytochrome c oxidase subunit NDUFA4 OS=Danio rerio OX=7955 GN=ndufa4 PE=3 SV=1	cytochrome c oxidase subunit NDUFA4 OS=Carassius auratus OX=7957 GN=LOC113112237 PE=4 SV=1
gene_Car0334010	6595786	6606494	fam117abB	protein FAM117A-like isoform X1 [Carassius auratus]	Protein FAM117A OS=Homo sapiens OX=9606 GN=FAM117A PE=1 SV=1	protein FAM117A-like isoform X1 OS=Carassius auratus OX=7957 GN=LOC113111902 PE=4 SV=1
gene_Car0334020	6608510	6624922	rundc3abB	RUN domain-containing protein 3A-like [Carassius auratus]	RUN domain-containing protein 3A OS=Danio rerio OX=7955 GN=rundc3a PE=2 SV=1	RUN domain-containing protein 3A-like OS=Carassius auratus OX=7957 GN=LOC113111901 PE=4 SV=1
gene_Car0334030	6649479	6683638	kcnh6bB	potassium voltage-gated channel subfamily H member 6-like [Carassius gibelio]	Potassium voltage-gated channel subfamily H member 6 OS=Gallus gallus OX=9031 GN=KCNH6 PE=2 SV=2	potassium voltage-gated channel subfamily H member 6-like OS=Carassius auratus OX=7957 GN=LOC113111897 PE=4 SV=1
gene_Car0334040	6687158	6720922	erbb2B	receptor tyrosine-protein kinase erbB-2-like [Carassius auratus]	Receptor tyrosine-protein kinase erbB-2 OS=Homo sapiens OX=9606 GN=ERBB2 PE=1 SV=1	Receptor protein-tyrosine kinase OS=Carassius auratus OX=7957 GN=LOC113111896 PE=3 SV=1
gene_Car0334050	6723954	6735626	pgap3B	post-GPI attachment to proteins factor 3 [Carassius auratus]	Post-GPI attachment to proteins factor 3 OS=Danio rerio OX=7955 GN=pgap3 PE=2 SV=1	Post-GPI attachment to proteins factor 3 OS=Carassius auratus OX=7957 GN=LOC113111895 PE=3 SV=1
gene_Car0334060	6740075	6744054	dnajc9B	dnaJ homolog subfamily C member 9-like [Carassius auratus]	DnaJ homolog subfamily C member 9 OS=Mus musculus OX=10090 GN=Dnajc9 PE=1 SV=2	dnaJ homolog subfamily C member 9-like OS=Carassius auratus OX=7957 GN=LOC113111894 PE=4 SV=1
gene_Car0334070	6744134	6747063	si:ch211-207i20.3-2B	hypothetical protein cypCar_00006099 [Cyprinus carpio]	--	Si:ch211-207i20.3 OS=Cyprinus carpio OX=7962 PE=4 SV=1
gene_Car0334080	6747378	6751486	nudt13	nucleoside diphosphate-linked moiety X motif 13 isoform X1 [Carassius gibelio]	NAD(P)H pyrophosphatase NUDT13, mitochondrial OS=Mus musculus OX=10090 GN=Nudt13 PE=1 SV=2	NAD(+) diphosphatase OS=Carassius auratus OX=7957 GN=nudt13 PE=4 SV=1
gene_Car0334090	6752768	6774641	p4ha1aB	prolyl 4-hydroxylase subunit alpha-1-like isoform X1 [Carassius auratus]	Prolyl 4-hydroxylase subunit alpha-1 OS=Bos taurus OX=9913 GN=P4HA1 PE=1 SV=1	procollagen-proline 4-dioxygenase OS=Carassius auratus OX=7957 GN=LOC113111890 PE=3 SV=1
gene_Car0334100	6775444	6782536	arg1B	arginase-1 [Carassius auratus]	Arginase-1 OS=Homo sapiens OX=9606 GN=ARG1 PE=1 SV=2	Arginase OS=Carassius auratus OX=7957 GN=arg1 PE=3 SV=1
gene_Car0334110	6801950	6834496	ppp1r9bbB	neurabin-2 [Carassius gibelio]	Neurabin-2 OS=Homo sapiens OX=9606 GN=PPP1R9B PE=1 SV=3	neurabin-2-like OS=Carassius auratus OX=7957 GN=LOC113111889 PE=4 SV=1
gene_Car0334120	6835349	6842034	prdm9B	histone-lysine N-methyltransferase PRDM9-like [Carassius gibelio]	Histone-lysine N-methyltransferase PRDM9 OS=Danio rerio OX=7955 GN=prdm9 PE=2 SV=1	histone-lysine N-methyltransferase PRDM9-like OS=Carassius auratus OX=7957 GN=LOC113111888 PE=4 SV=1
gene_Car0334130	6847386	6851203	NA	unnamed protein product [Mustela putorius furo]	LINE-1 reverse transcriptase homolog OS=Nycticebus coucang OX=9470 PE=4 SV=1	Reverse transcriptase domain-containing protein OS=Paramormyrops kingsleyae OX=1676925 PE=4 SV=1
gene_Car0334140	7013627	7014427	lrrc3caB	leucine-rich repeat-containing protein 3B-like [Carassius auratus]	Leucine-rich repeat-containing protein 3B OS=Homo sapiens OX=9606 GN=LRRC3B PE=1 SV=1	leucine-rich repeat-containing protein 3B-like OS=Carassius auratus OX=7957 GN=LOC113111884 PE=4 SV=1
gene_Car0334150	7025126	7028339	csf3a	uncharacterized protein LOC113112236 [Carassius auratus]	--	uncharacterized protein LOC113112236 OS=Carassius auratus OX=7957 GN=LOC113112236 PE=3 SV=1
gene_Car0334160	7030931	7054474	med24B	mediator of RNA polymerase II transcription subunit 24 [Carassius gibelio]	Mediator of RNA polymerase II transcription subunit 24 OS=Danio rerio OX=7955 GN=med24 PE=2 SV=2	Mediator of RNA polymerase II transcription subunit 24 OS=Carassius auratus OX=7957 GN=LOC113111883 PE=3 SV=1
gene_Car0334170	7057208	7065848	samd14B	sterile alpha motif domain-containing protein 14-like isoform X3 [Carassius auratus]	Sterile alpha motif domain-containing protein 14 OS=Rattus norvegicus OX=10116 GN=Samd14 PE=1 SV=1	sterile alpha motif domain-containing protein 14-like isoform X3 OS=Carassius auratus OX=7957 GN=LOC113111882 PE=4 SV=1
gene_Car0334180	7069307	7092754	top2a	DNA topoisomerase 2-alpha [Carassius gibelio]	DNA topoisomerase 2-alpha OS=Sus scrofa OX=9823 GN=TOP2A PE=2 SV=1	DNA topoisomerase 2 OS=Carassius auratus OX=7957 GN=LOC113111881 PE=3 SV=1
gene_Car0334190	7147597	7293918	raraaB	retinoic acid receptor alpha isoform X1 [Carassius gibelio]	Retinoic acid receptor alpha OS=Takifugu rubripes OX=31033 GN=rara PE=2 SV=1	retinoic acid receptor alpha isoform X1 OS=Carassius auratus OX=7957 GN=LOC113111880 PE=3 SV=1
gene_Car0334200	7301420	7309389	NA	UPF0505 C16orf62-like protein [Labeo rohita]	VPS35 endosomal protein-sorting factor-like OS=Danio rerio OX=7955 GN=vps35l PE=2 SV=1	VPS35 endosomal protein-sorting factor-like OS=Cyprinus carpio OX=7962 PE=3 SV=1
gene_Car0334210	7321618	7337300	get4B	Golgi to ER traffic protein 4 homolog [Carassius auratus]	Golgi to ER traffic protein 4 homolog OS=Salmo salar OX=8030 GN=get4 PE=2 SV=1	Golgi to ER traffic protein 4 homolog OS=Carassius auratus OX=7957 GN=LOC113111877 PE=3 SV=1

gene_Car0334220	7339120	7346879	si:rp71-19m20B	ATPase PAAT [Carassius gibelio]	ATPase PAAT OS=Homo sapiens OX=9606 GN=PAAT PE=1 SV=2	uncharacterized protein C10orf88-like OS=Carassius auratus OX=7957 GN=LOC113111876 PE=4 SV=1
gene_Car0334230	7351791	7387754	htra1bB	serine protease HTRA1B [Carassius auratus]	Serine protease HTRA1B OS=Danio rerio OX=7955 GN=htra1b PE=2 SV=1	serine protease HTRA1B OS=Carassius auratus OX=7957 GN=LOC113111875 PE=3 SV=1
gene_Car0334240	7406791	7441561	plekha1bB	pleckstrin homology domain-containing family A member 1-like isoform X1 [Carassius gibelio]	Pleckstrin homology domain-containing family A member 1 OS=Homo sapiens OX=9606 GN=PLEKHA1 PE=1 SV=2	pleckstrin homology domain-containing family A member 1-like isoform X1 OS=Carassius auratus OX=7957 GN=LOC113111873 PE=4 SV=1
gene_Car0334250	7445820	7463372	btbd16B	BTB/POZ domain-containing protein 16-like [Carassius gibelio]	BTB/POZ domain-containing protein 16 OS=Bos taurus OX=9913 GN=BTBD16 PE=2 SV=1	BTB/POZ domain-containing protein 16 OS=Carassius auratus OX=7957 GN=LOC113111872 PE=4 SV=1
gene_Car0334260	7463812	7522065	waplbB	LOW QUALITY PROTEIN: wings apart-like protein homolog [Carassius gibelio]	Wings apart-like protein homolog OS=Homo sapiens OX=9606 GN=WAPL PE=1 SV=1	wings apart-like protein homolog OS=Carassius auratus OX=7957 GN=LOC113111871 PE=3 SV=1
gene_Car0334270	7524550	7525158	atoh1cB	neurogenin-1-like [Carassius gibelio]	Transcription factor ATOH1 OS=Homo sapiens OX=9606 GN=ATOH1 PE=2 SV=1	neurogenin-1-like OS=Carassius auratus OX=7957 GN=LOC113112235 PE=4 SV=1
gene_Car0334280	7675283	7677442	NA	zinc finger MYM-type protein 1-like [Carassius auratus]	Zinc finger MYM-type protein 1 OS=Homo sapiens OX=9606 GN=ZMYM1 PE=1 SV=1	Zinc finger MYM-type protein 1 OS=Cyprinus carpio OX=7962 PE=4 SV=1
gene_Car0334290	7707619	8125923	grid1b	glutamate receptor ionotropic, delta-1 isoform X1 [Ctenopharyngodon idella]	Glutamate receptor ionotropic, delta-1 OS=Homo sapiens OX=9606 GN=GRID1 PE=2 SV=2	Glutamate receptor OS=Cyprinus carpio OX=7962 PE=3 SV=1
gene_Car0334300	8217060	8281526	ccser2bB	serine-rich coiled-coil domain-containing protein 2-like isoform X1 [Carassius auratus]	Serine-rich coiled-coil domain-containing protein 2 OS=Mus musculus OX=10090 GN=Ccser2 PE=1 SV=1	serine-rich coiled-coil domain-containing protein 2-like isoform X1 OS=Carassius auratus OX=7957 GN=LOC113112109 PE=3 SV=1
gene_Car0334310	8291788	8299441	si:ch211-103b1	protein PAT1 homolog 2 [Carassius auratus]	Protein PAT1 homolog 2 OS=Xenopus laevis OX=8355 GN=patl2 PE=1 SV=1	protein PAT1 homolog 2 OS=Carassius auratus OX=7957 GN=patl2 PE=3 SV=1
gene_Car0334320	8301696	8318931	cmn	glutenin, high molecular weight subunit PW212-like isoform X1 [Carassius auratus]	--	glutenin, high molecular weight subunit PW212-like isoform X1 OS=Carassius auratus OX=7957 GN=LOC113112112 PE=4 SV=1
gene_Car0334330	8325992	8331993	peloB	protein pelota homolog [Carassius auratus]	Protein pelota homolog OS=Danio rerio OX=7955 GN=pelo PE=2 SV=1	Protein pelota homolog OS=Carassius auratus OX=7957 GN=LOC113112114 PE=3 SV=1
gene_Car0334340	8332417	8343582	ppp1cabB	serine/threonine-protein phosphatase alpha-2 isoform-like [Cyprinus carpio]	Serine/threonine-protein phosphatase PP1-alpha catalytic subunit OS=Bos taurus OX=9913 GN=PPP1CA PE=2 SV=1	Serine/threonine-protein phosphatase OS=Cyprinus carpio OX=7962 GN=LOC109084484 PE=3 SV=1
gene_Car0334350	8391582	8402357	atp2a1lB	sarcoplasmic/endoplasmic reticulum calcium ATPase 1 isoform X2 [Carassius auratus]	Sarcoplasmic/endoplasmic reticulum calcium ATPase 1 OS=Makaira nigricans OX=13604 GN=atp2a1 PE=2 SV=2	Calcium-transporting ATPase OS=Carassius auratus OX=7957 GN=LOC113112117 PE=3 SV=1
gene_Car0334360	8406256	8412197	irf9B	interferon regulatory factor 8-like [Carassius auratus]	--	interferon regulatory factor 8-like OS=Carassius auratus OX=7957 GN=LOC113112118 PE=3 SV=1
gene_Car0334370	8413570	8419141	emc9B	ER membrane protein complex subunit 9-like [Carassius auratus]	ER membrane protein complex subunit 9 OS=Homo sapiens OX=9606 GN=EMC9 PE=1 SV=3	ER membrane protein complex subunit 9-like OS=Carassius auratus OX=7957 GN=LOC113112120 PE=3 SV=1
gene_Car0334380	8424175	8428900	psme2B	proteasome activator complex subunit 2-like [Carassius auratus]	Proteasome activator complex subunit 2 OS=Homo sapiens OX=9606 GN=PSME2 PE=1 SV=4	proteasome activator complex subunit 2-like OS=Carassius auratus OX=7957 GN=LOC113112121 PE=3 SV=1
gene_Car0334390	8430760	8460152	rnasenB	ribonuclease 3-like [Carassius auratus]	Ribonuclease 3 OS=Homo sapiens OX=9606 GN=DROSHA PE=1 SV=2	ribonuclease 3-like OS=Carassius auratus OX=7957 GN=LOC113112123 PE=3 SV=1
gene_Car0334400	8505183	8512010	kcnh4bB	potassium voltage-gated channel subfamily H member 4-like isoform X1 [Carassius auratus]	Potassium voltage-gated channel subfamily H member 4 OS=Rattus norvegicus OX=10116 GN=Kcnh4 PE=2 SV=1	potassium voltage-gated channel subfamily H member 4-like isoform X1 OS=Carassius auratus OX=7957 GN=LOC113112124 PE=4 SV=1
gene_Car0334410	8522764	8522967	NA	GH3 domain-containing protein [Puntigrus tetrazona]	--	GH3 domain-containing OS=Labeo rohita OX=84645 GN=ROHU_016022 PE=4 SV=1
gene_Car0334420	8541841	8587236	stat5bB	signal transducer and activator of transcription 5B-like isoform X2 [Carassius auratus]	Signal transducer and activator of transcription 5B OS=Mus musculus OX=10090 GN=Stat5b PE=1 SV=1	Signal transducer and activator of transcription OS=Carassius auratus OX=7957 GN=LOC113112125 PE=3 SV=1
gene_Car0334430	8611945	8616559	foxh1B	forkhead box protein H1 [Carassius auratus]	Forkhead box protein H1 OS=Danio rerio OX=7955 GN=foxh1 PE=1 SV=1	forkhead box protein H1 OS=Carassius auratus OX=7957 GN=LOC113112127 PE=4 SV=1
gene_Car0334440	8623226	8652779	ppp1r16aB	protein phosphatase 1 regulatory subunit 16A-like [Carassius auratus]	Protein phosphatase 1 regulatory subunit 16A OS=Mus musculus OX=10090 GN=Ppp1r16a PE=1 SV=1	protein phosphatase 1 regulatory subunit 16A-like OS=Carassius auratus OX=7957 GN=LOC113112129 PE=4 SV=1
gene_Car0334450	8665093	8701398	adam11B	disintegrin and metalloproteinase domain-containing protein 11-like isoform X1 [Carassius auratus]	Disintegrin and metalloproteinase domain-containing protein 11 OS=Mus musculus OX=10090 GN=Adam11 PE=1 SV=2	disintegrin and metalloproteinase domain-containing protein 11-like isoform X1 OS=Carassius auratus OX=7957 GN=LOC113112128 PE=4 SV=1
gene_Car0334460	8703268	8714709	dbf4bB	uncharacterized protein LOC113112130 isoform X1 [Carassius auratus]	Protein DBF4 homolog B OS=Xenopus laevis OX=8355 GN=dbf4b PE=1 SV=1	uncharacterized protein LOC113112130 isoform X1 OS=Carassius auratus OX=7957 GN=LOC113112130 PE=4 SV=1
gene_Car0334470	8716564	8729421	NA	peptidyl-prolyl cis-trans isomerase FKBP10-like [Cyprinus carpio]	Peptidyl-prolyl cis-trans isomerase FKBP10 OS=Mus musculus OX=10090 GN=Fkbp10 PE=1 SV=2	peptidylprolyl isomerase OS=Cyprinus carpio OX=7962 GN=LOC109104059 PE=4 SV=1
gene_Car0334480	8729554	8785634	klhl11B	kelch-like protein 11 [Cyprinus carpio]	Kelch-like protein 11 OS=Homo sapiens OX=9606 GN=KLHL11 PE=1 SV=1	Kelch-like family member 11 OS=Cyprinus carpio OX=7962 PE=4 SV=1
gene_Car0334490	8798239	8802121	NA	hypothetical protein cypCar_00029854 [Cyprinus carpio]	--	uncharacterized protein LOC113044374 OS=Carassius auratus OX=7957 GN=LOC113044374 PE=4 SV=1
gene_Car0334500	8823277	8830241	NA	uncharacterized protein LOC120480078 isoform X2 [Pimephales promelas]	--	uncharacterized protein LOC113039795 OS=Carassius auratus OX=7957 GN=LOC113039795 PE=4 SV=1
gene_Car0334510	8967739	9152663	adprhB	protein ADP-ribosylarginine hydrolase-like isoform X1 [Carassius auratus]	ADP-ribosylhydrolase ARH1 OS=Homo sapiens OX=9606 GN=ADPRH PE=1 SV=1	protein ADP-ribosylarginine hydrolase-like isoform X1 OS=Carassius auratus OX=7957 GN=LOC113112133 PE=3 SV=1
gene_Car0334520	9245415	9277044	LOC100332609B	phenylethanolamine N-methyltransferase [Carassius gibelio]	Phenylethanolamine N-methyltransferase OS=Bos taurus OX=9913 GN=PNMT PE=1 SV=2	Phenylethanolamine N-methyltransferase OS=Onychostoma macrolepis OX=369639 GN=G5714_012735 PE=3 SV=1

gene_Car0334530	9391498	9394401	NA	neurogenic differentiation factor 2-like [Carassius auratus]	Neurogenic differentiation factor 2 OS=Danio rerio OX=7955 GN=neurod2 PE=2 SV=1	Neurogenic differentiation factor OS=Carassius auratus OX=7957 GN=LOC113071170 PE=4 SV=1
gene_Car0334540	9534404	9536854	NA	uncharacterized protein LOC113065302 [Carassius auratus]	Retrovirus-related Pol polyprotein from transposon 17.6 OS=Drosophila melanogaster OX=7227 GN=pol PE=4 SV=1	uncharacterized protein LOC113065302 OS=Carassius auratus OX=7957 GN=LOC113065302 PE=4 SV=1
gene_Car0334550	9539101	9540480	NA	uncharacterized protein LOC113093154 [Carassius auratus]	--	uncharacterized protein LOC113093154 OS=Carassius auratus OX=7957 GN=LOC113093154 PE=4 SV=1
gene_Car0334560	9566728	9570313	mylpfbB	PREDICTED: myosin regulatory light chain 2, skeletal muscle isoform type 2 [Sinocyclocheilus grahami]	Myosin regulatory light chain 2, skeletal muscle isoform B OS=Danio rerio OX=7955 GN=mylpfb PE=2 SV=1	Myosin light chain, phosphorylatable, fast skeletal muscle b OS=Cyprinus carpio OX=7962 PE=4 SV=1
gene_Car0334570	9941377	9942933	NA	Transposon Tf2-8 polyprotein [Labeo rohita]	Probable RNA-directed DNA polymerase from transposon BS OS=Drosophila melanogaster OX=7227 GN=RTase PE=2 SV=1	Reverse transcriptase domain-containing protein OS=Cyprinus carpio OX=7962 PE=4 SV=1
gene_Car0334580	9951106	9952485	NA	zinc finger BED domain-containing protein 4-like [Pimephales promelas]	--	BED-type domain-containing protein OS=Cyprinus carpio OX=7962 PE=4 SV=1
gene_Car0334590	10031159	10462432	lrp2b	Low-density lipoprotein receptor-related protein 2 [Triplophysa tibetana]	Low-density lipoprotein receptor-related protein 2 OS=Mus musculus OX=10090 GN=Lrp2 PE=1 SV=1	Low-density lipoprotein receptor-related protein 2 OS=Triplophysa tibetana OX=1572043 GN=E1301_Tti006065 PE=3 SV=1
gene_Car0334600	10563457	10565617	NA	MAGUK p55 subfamily member 3-like isoform X3 [Cyprinus carpio]	MAGUK p55 subfamily member 3 OS=Homo sapiens OX=9606 GN=MPP3 PE=1 SV=2	Membrane protein, palmitoylated 3b (MAGUK p55 subfamily member 3) OS=Cyprinus carpio OX=7962 PE=3 SV=1
gene_Car0334610	10590745	10594935	NA	protein phosphatase 1 regulatory subunit 3C-B isoform X1 [Carassius gibelio]	Protein phosphatase 1 regulatory subunit 3C-B OS=Danio rerio OX=7955 GN=ppp1r3cb PE=2 SV=1	Protein phosphatase 1 regulatory subunit 3C OS=Carassius auratus OX=7957 GN=LOC113111705 PE=4 SV=1
gene_Car0334620	10683173	10701442	ankrd1b	PREDICTED: ankyrin repeat domain-containing protein 1-like [Sinocyclocheilus grahami]	Ankyrin repeat domain-containing protein 1 OS=Sus scrofa OX=9823 GN=ANKRD1 PE=2 SV=1	Ankyrin repeat domain-containing protein 1-like OS=Sinocyclocheilus grahami OX=75366 GN=LOC107584237 PE=4 SV=1
gene_Car0334630	10701161	10705139	NA	ribonuclease P protein subunit p30-like isoform X1 [Carassius auratus]	Ribonuclease P protein subunit p30 OS=Bos taurus OX=9913 GN=RPP30 PE=2 SV=1	ribonuclease P protein subunit p30-like isoform X1 OS=Carassius auratus OX=7957 GN=LOC113111702 PE=3 SV=1
gene_Car0334640	10705386	10732633	kif20bbB	kinesin-like protein KIF20B isoform X1 [Carassius auratus]	Kinesin-like protein KIF20B OS=Homo sapiens OX=9606 GN=KIF20B PE=1 SV=3	kinesin-like protein KIF20B isoform X1 OS=Carassius auratus OX=7957 GN=LOC113111701 PE=3 SV=1
gene_Car0334650	10734432	10736562	htr7B	5-hydroxytryptamine receptor 7-like [Carassius auratus]	5-hydroxytryptamine receptor 7 OS=Homo sapiens OX=9606 GN=HTR7 PE=1 SV=2	5-hydroxytryptamine receptor 7 OS=Carassius auratus OX=7957 GN=LOC113112221 PE=3 SV=1
gene_Car0334660	10763115	10772613	pank1bB	pantothenate kinase 3-like [Carassius auratus]	Pantothenate kinase 1 OS=Homo sapiens OX=9606 GN=PANK1 PE=1 SV=2	pantothenate kinase 3-like OS=Carassius auratus OX=7957 GN=LOC113111801 PE=4 SV=1
gene_Car0334670	10773723	10780808	slc16a12bB	monocarboxylate transporter 12-B [Carassius auratus]	Monocarboxylate transporter 12-B OS=Danio rerio OX=7955 GN=slc16a12b PE=2 SV=1	monocarboxylate transporter 12-B OS=Carassius auratus OX=7957 GN=LOC113111798 PE=4 SV=1
gene_Car0334680	10795773	10798575	ifit12B	interferon-induced protein with tetratricopeptide repeats 5-like [Carassius gibelio]	Interferon-induced protein with tetratricopeptide repeats 5 OS=Homo sapiens OX=9606 GN=IFIT5 PE=1 SV=1	Interferon-induced protein with tetratricopeptide repeats 1-like OS=Sinocyclocheilus rhinocerous OX=307959 GN=LOC107753456 PE=4 SV=1
gene_Car0334690	10811347	10814170	ifit9	interferon-induced protein with tetratricopeptide repeats 5-like [Carassius auratus]	Interferon-induced protein with tetratricopeptide repeats 5 OS=Homo sapiens OX=9606 GN=IFIT5 PE=1 SV=1	interferon-induced protein with tetratricopeptide repeats 5-like OS=Carassius auratus OX=7957 GN=LOC113111796 PE=4 SV=1
gene_Car0334700	10815674	10816761	ch25hB	cholesterol 25-hydroxylase-like protein [Carassius gibelio]	Cholesterol 25-hydroxylase-like protein OS=Danio rerio OX=7955 GN=ch25h PE=2 SV=1	cholesterol 25-hydroxylase-like protein OS=Carassius auratus OX=7957 GN=LOC113112232 PE=4 SV=1
gene_Car0334710	10836218	10842063	acta2B	actin, aortic smooth muscle [Danio rerio]	Actin, aortic smooth muscle OS=Bos taurus OX=9913 GN=ACTA2 PE=1 SV=1	Actin alpha 2, smooth muscle OS=Oncorhynchus mykiss OX=8022 GN=GSONMT00065464001 PE=3 SV=1
gene_Car0334720	10842737	10853041	stambpl1B	AMSH-like protease [Carassius gibelio]	AMSH-like protease OS=Homo sapiens OX=9606 GN=STAMBPL1 PE=1 SV=2	AMSH-like protease OS=Carassius auratus OX=7957 GN=stambpl1 PE=3 SV=1
gene_Car0334730	10857045	10869473	lipfB	lysosomal acid lipase/cholesteryl ester hydrolase-like [Carassius auratus]	Lysosomal acid lipase/cholesteryl ester hydrolase OS=Homo sapiens OX=9606 GN=LIPA PE=1 SV=2	lysosomal acid lipase/cholesteryl ester hydrolase-like OS=Carassius auratus OX=7957 GN=LOC113111790 PE=4 SV=1
gene_Car0334740	10869908	10932044	rnlsB	renalase isoform X1 [Carassius gibelio]	Renalase OS=Homo sapiens OX=9606 GN=RNLS PE=1 SV=1	renalase isoform X1 OS=Carassius auratus OX=7957 GN=rnls PE=4 SV=1
gene_Car0334750	10961212	10983635	ptenbB	phosphatidylinositol 3,4,5-trisphosphate 3-phosphatase and dual-specificity protein phosphatase PTEN isoform X1 [Carassius auratus]	Phosphatidylinositol 3,4,5-trisphosphate 3-phosphatase and dual-specificity protein phosphatase PTEN OS=Canis lupus familiaris OX=9615 GN=PTEN PE=2 SV=1	Phosphatidylinositol 3,4,5-trisphosphate 3-phosphatase and dual-specificity protein phosphatase PTEN OS=Carassius auratus OX=7957 GN=LOC113111789 PE=3 SV=1
gene_Car0334760	11021463	11029226	atad1bB	ATPase family AAA domain-containing protein 1 [Carassius auratus]	Outer mitochondrial transmembrane helix translocase OS=Danio rerio OX=7955 GN=atad1b PE=2 SV=2	ATPase family AAA domain-containing protein 1 OS=Carassius auratus OX=7957 GN=atad1 PE=3 SV=1
gene_Car0334770	11025330	11035212	papss2bB	bifunctional 3'-phosphoadenosine 5'-phosphosulfate synthase 2 [Carassius auratus]	Bifunctional 3'-phosphoadenosine 5'-phosphosulfate synthase 2 OS=Homo sapiens OX=9606 GN=PAPSS2 PE=1 SV=2	bifunctional 3'-phosphoadenosine 5'-phosphosulfate synthase 2 OS=Carassius auratus OX=7957 GN=papss2 PE=3 SV=1
gene_Car0334780	11036520	11039524	NA	multiple inositol polyphosphate phosphatase 1 [Carassius auratus]	Multiple inositol polyphosphate phosphatase 1 OS=Rattus norvegicus OX=10116 GN=Minpp1 PE=1 SV=3	Multiple inositol polyphosphate phosphatase 1 OS=Carassius auratus OX=7957 GN=minpp1 PE=3 SV=1
gene_Car0334790	11052878	11067019	cyth3aB	cytohesin-3-like [Carassius auratus]	Cytohesin-3 OS=Mus musculus OX=10090 GN=Cyth3 PE=1 SV=1	cytohesin-3-like OS=Carassius auratus OX=7957 GN=LOC113111783 PE=4 SV=1
gene_Car0334800	11068123	11105556	usp42B	ubiquitin carboxyl-terminal hydrolase 42-like isoform X2 [Carassius auratus]	Ubiquitin carboxyl-terminal hydrolase 42 OS=Bos taurus OX=9913 GN=USP42 PE=3 SV=1	ubiquitin carboxyl-terminal hydrolase 42-like isoform X2 OS=Carassius auratus OX=7957 GN=LOC113111777 PE=4 SV=1
gene_Car0334810	11105745	11113181	eif2ak1B	eukaryotic translation initiation factor 2-alpha kinase 1-like isoform X1 [Carassius auratus]	Eukaryotic translation initiation factor 2-alpha kinase 1 OS=Macaca fascicularis OX=9541 GN=EIF2AK1 PE=2 SV=1	eukaryotic translation initiation factor 2-alpha kinase 1-like isoform X1 OS=Carassius auratus OX=7957 GN=LOC113111780 PE=4 SV=1
gene_Car0334820	11114698	11120740	rsph10b	radial spoke head 10 homolog B [Carassius gibelio]	Radial spoke head 10 homolog B OS=Danio rerio OX=7955 GN=rsph10b PE=2 SV=1	radial spoke head 10 homolog B-like isoform X2 OS=Carassius auratus OX=7957 GN=LOC113111779 PE=4 SV=1
gene_Car0334830	11132816	11147057	ccz1B	hypothetical protein PFLUV_G00245720 [Perca fluviatilis]	Vacuolar fusion protein CCZ1 homolog OS=Danio rerio OX=7955 GN=ccz1 PE=2 SV=1	EF-hand domain-containing protein OS=Perca fluviatilis OX=8168 GN=PFLUV_G00245720 PE=3 SV=1

gene_Car0334840	11156576	11157088	pvalb2B	parvalbumin-2 [Carassius auratus]	Parvalbumin-2 OS=Danio rerio OX=7955 GN=pvalb2 PE=3 SV=3	Parvalbumin OS=Carassius auratus OX=7957 GN=LOC113111772 PE=3 SV=1
gene_Car0334850	11158806	11160591	NA	Parvalbumin-2 [Labeo rohita]	Parvalbumin beta OS=Graptemys geographica OX=8481 PE=1 SV=2	Parvalbumin OS=Labeo rohita OX=84645 GN=ROHU_033909 PE=3 SV=1
gene_Car0334860	11172464	11178690	bhlha15B	class A basic helix-loop-helix protein [Pimephales promelas]	Class A basic helix-loop-helix protein 15 OS=Homo sapiens OX=9606 GN=BHLHA15 PE=1 SV=1	BHLH domain-containing protein OS=Onychostoma macrolepis OX=369639 GN=G5714_012683 PE=4 SV=1
gene_Car0334870	11175856	11191706	tecpr1aB	tectonin beta-propeller repeat-containing protein 1 [Cyprinus carpio]	Tectonin beta-propeller repeat-containing protein 1 OS=Gallus gallus OX=9031 GN=TECPR1 PE=3 SV=1	Tectonin beta-propeller repeat containing 1a OS=Cyprinus carpio OX=7962 PE=3 SV=1
gene_Car0334880	11206641	11224327	baiap2l1aB	brain-specific angiogenesis inhibitor 1-associated protein 2-like protein 1 isoform X1 [Carassius auratus]	Brain-specific angiogenesis inhibitor 1-associated protein 2-like protein 1 OS=Mus musculus OX=10090 GN=Baiap2l1 PE=1 SV=1	brain-specific angiogenesis inhibitor 1-associated protein 2-like protein 1 isoform X1 OS=Carassius auratus OX=7957 GN=LOC113111769 PE=4 SV=1
gene_Car0334890	11235888	11246849	nptx2bB	neuronal pentraxin-2-like isoform X1 [Carassius auratus]	Neuronal pentraxin-2 OS=Homo sapiens OX=9606 GN=NPTX2 PE=1 SV=2	neuronal pentraxin-2-like isoform X1 OS=Carassius auratus OX=7957 GN=LOC113111768 PE=4 SV=1
gene_Car0334900	11253914	11335192	trrapB	transformation/transcription domain-associated protein [Carassius auratus]	Transformation/transcription domain-associated protein OS=Danio rerio OX=7955 GN=trrap PE=3 SV=1	transformation/transcription domain-associated protein OS=Carassius auratus OX=7957 GN=LOC113111767 PE=3 SV=1
gene_Car0334910	11382224	11427733	fam20cbB	extracellular serine/threonine protein kinase FAM20C [Carassius gibelio]	Extracellular serine/threonine protein kinase FAM20C OS=Mus musculus OX=10090 GN=Fam20c PE=1 SV=1	FAM20C golgi associated secretory pathway kinase b OS=Cyprinus carpio OX=7962 PE=3 SV=1
gene_Car0334920	11431020	11433022	si:dkey-7e14B	forkhead box L3 [Carassius gibelio]	Forkhead box protein L3 OS=Homo sapiens OX=9606 GN=FOXL3 PE=3 SV=1	forkhead box protein L1-like OS=Carassius auratus OX=7957 GN=LOC113112230 PE=4 SV=1
gene_Car0334930	11437513	11437985	NA	--	--	--
gene_Car0334940	11438071	11453379	tom1l2-2B	TOM1-like protein 2 isoform X3 [Puntigrus tetrazona]	TOM1-like protein 2 OS=Homo sapiens OX=9606 GN=TOM1L2 PE=1 SV=1	Target of myb1 like 2 membrane trafficking protein OS=Cyprinus carpio OX=7962 PE=3 SV=1
gene_Car0334950	11454995	11457997	atpaf2B	ATP synthase mitochondrial F1 complex assembly factor 2-like [Carassius auratus]	ATP synthase mitochondrial F1 complex assembly factor 2 OS=Mus musculus OX=10090 GN=Atpaf2 PE=1 SV=1	ATP synthase mitochondrial F1 complex assembly factor 2-like OS=Carassius auratus OX=7957 GN=LOC113111764 PE=3 SV=1
gene_Car0334960	11494236	11497544	noxo1bB	NADPH oxidase organizer 1-like [Carassius auratus]	NADPH oxidase organizer 1 OS=Homo sapiens OX=9606 GN=NOXO1 PE=1 SV=1	NADPH oxidase organizer 1-like OS=Carassius auratus OX=7957 GN=LOC113112228 PE=4 SV=1
gene_Car0334970	11499855	11502711	rnf151-2	RING finger protein 151-like [Carassius auratus]	RING finger protein 151 OS=Bos taurus OX=9913 GN=RNF151 PE=2 SV=1	RING finger protein 151-like OS=Carassius auratus OX=7957 GN=LOC113111763 PE=4 SV=1
gene_Car0334980	11503152	11515686	tex2l	testis-expressed protein 2-like [Carassius auratus]	Testis-expressed protein 2 OS=Homo sapiens OX=9606 GN=TEX2 PE=1 SV=2	testis-expressed protein 2-like OS=Carassius auratus OX=7957 GN=LOC113111762 PE=4 SV=1
gene_Car0334990	11520478	11521994	neurl2B	neuralized-like protein 2 [Carassius gibelio]	Neuralized-like protein 2 OS=Homo sapiens OX=9606 GN=NEURL2 PE=2 SV=1	neuralized-like protein 2 OS=Carassius auratus OX=7957 GN=LOC113111761 PE=4 SV=1
gene_Car0335000	11527005	11530913	meiob	meiosis-specific with OB domain-containing protein [Carassius auratus]	Meiosis-specific with OB domain-containing protein OS=Mus musculus OX=10090 GN=Meiob PE=1 SV=3	meiosis-specific with OB domain-containing protein OS=Carassius auratus OX=7957 GN=meiob PE=4 SV=1
gene_Car0335010	11531322	11535422	mlst8B	PREDICTED: target of rapamycin complex subunit LST8 [Sinocyclocheilus grahami]	Target of rapamycin complex subunit lst8 OS=Danio rerio OX=7955 GN=mlst8 PE=2 SV=1	Target of rapamycin complex subunit lst8 OS=Carassius auratus OX=7957 GN=LOC113111756 PE=3 SV=1
gene_Car0335020	11535827	11540866	bricd5	hypothetical protein cypCar_00019579 [Cyprinus carpio]	BRICHOS domain-containing protein 5 OS=Homo sapiens OX=9606 GN=BRICD5 PE=1 SV=3	BRICHOS domain-containing protein 5 isoform X1 OS=Carassius auratus OX=7957 GN=bricd5 PE=4 SV=1
gene_Car0335030	11541471	11543097	pgpB	glycerol-3-phosphate phosphatase-like [Carassius auratus]	Glycerol-3-phosphate phosphatase OS=Homo sapiens OX=9606 GN=PGP PE=1 SV=1	glycerol-3-phosphate phosphatase-like OS=Carassius auratus OX=7957 GN=LOC113111757 PE=3 SV=1
gene_Car0335040	11551825	11584472	grid2ipbB	delphilin isoform X1 [Carassius gibelio]	Delphilin OS=Danio rerio OX=7955 GN=grid2ip PE=3 SV=1	delphilin-like isoform X1 OS=Carassius auratus OX=7957 GN=LOC113111754 PE=4 SV=1
gene_Car0335050	11585451	11610083	arhgap17aB	rho GTPase-activating protein 17-like isoform X1 [Carassius auratus]	Rho GTPase-activating protein 17 OS=Mus musculus OX=10090 GN=Arhgap17 PE=1 SV=1	rho GTPase-activating protein 17-like isoform X1 OS=Carassius auratus OX=7957 GN=LOC113111753 PE=4 SV=1
gene_Car0335060	11613223	11626224	lcmt1B	leucine carboxyl methyltransferase 1-like [Carassius auratus]	Leucine carboxyl methyltransferase 1 OS=Homo sapiens OX=9606 GN=LCMT1 PE=1 SV=2	Leucine carboxyl methyltransferase 1 OS=Carassius auratus OX=7957 GN=LOC113111752 PE=3 SV=1
gene_Car0335070	11627613	11628905	aqp8aB	aquaporin-8-like [Carassius auratus]	Aquaporin-8 OS=Homo sapiens OX=9606 GN=AQP8 PE=1 SV=2	aquaporin-8-like OS=Carassius auratus OX=7957 GN=LOC113111751 PE=3 SV=1
gene_Car0335080	11630990	11631640	NA	PREDICTED: aquaporin-8-like [Sinocyclocheilus anshuiensis]	Aquaporin-8 OS=Homo sapiens OX=9606 GN=AQP8 PE=1 SV=2	Aquaporin-8-like OS=Sinocyclocheilus anshuiensis OX=1608454 GN=LOC107669187 PE=3 SV=1
gene_Car0335090	11633886	11634504	hbae4	hemoglobin subunit alpha-like [Carassius auratus]	Hemoglobin subunit alpha-4 OS=Danio rerio OX=7955 GN=hbaa1 PE=2 SV=3	hemoglobin subunit alpha-4-like OS=Carassius auratus OX=7957 GN=LOC113111750 PE=3 SV=1
gene_Car0335100	11635729	11636413	hbbe2B	hemoglobin subunit beta-2-like [Carassius auratus]	Hemoglobin subunit beta-2 OS=Boreogadus saida OX=44932 GN=hbb2 PE=1 SV=3	hemoglobin subunit beta-2-like OS=Carassius auratus OX=7957 GN=LOC113111749 PE=3 SV=1
gene_Car0335110	11641384	11642059	hbbe3	hypothetical protein cypCar_00047166 [Cyprinus carpio]	Hemoglobin subunit beta OS=Merlangius merlangus OX=8058 GN=hbb PE=2 SV=2	Hemoglobin beta embryonic-3 OS=Cyprinus carpio OX=7962 PE=3 SV=1
gene_Car0335120	11662973	11695658	rhbdf1bB	inactive rhomboid protein 1-like isoform X1 [Carassius auratus]	Inactive rhomboid protein 1 OS=Danio rerio OX=7955 GN=rhbdf1 PE=2 SV=1	Inactive rhomboid protein OS=Carassius auratus OX=7957 GN=LOC113111747 PE=3 SV=1
gene_Car0335130	11727727	11730611	foxj1bB	PREDICTED: forkhead box protein J1-B isoform X1 [Sinocyclocheilus rhinocerous]	Forkhead box protein J1-B OS=Danio rerio OX=7955 GN=foxj1b PE=2 SV=1	Forkhead box protein J1-B OS=Sinocyclocheilus rhinocerous OX=307959 GN=LOC107734171 PE=4 SV=1
gene_Car0335140	11731821	11743839	mgrn1aB	probable E3 ubiquitin-protein ligase MGRN1 isoform X1 [Carassius gibelio]	Probable E3 ubiquitin-protein ligase MGRN1 OS=Danio rerio OX=7955 GN=mgrn1 PE=2 SV=1	E3 ubiquitin-protein ligase OS=Carassius auratus OX=7957 GN=LOC113111745 PE=4 SV=1
gene_Car0335150	11744773	11754570	ubald1aB	UBA-like domain-containing protein 1 [Carassius auratus]	UBA-like domain-containing protein 1 OS=Danio rerio OX=7955 GN=ubald1 PE=2 SV=1	UBA-like domain-containing protein 1 OS=Cyprinus carpio OX=7962 GN=LOC109107293 PE=3 SV=1

gene_Car0335160	11761631	11766818	NA	uncharacterized protein LOC127968908 isoform X2 [Carassius gibelio]	Lipopolysaccharide-induced tumor necrosis factor-alpha factor homolog OS=Gallus gallus OX=9031 GN=LITAF PE=2 SV=1	LITAF domain-containing protein OS=Onychostoma macrolepis OX=369639 GN=G5714_012651 PE=3 SV=1
gene_Car0335170	11770814	11798664	mkl2aB	MKL/myocardin-like protein 2 [Carassius auratus]	Myocardin-related transcription factor B OS=Homo sapiens OX=9606 GN=MRTFB PE=1 SV=3	MKL/myocardin-like protein 2 OS=Carassius auratus OX=7957 GN=mrtfb PE=4 SV=1
gene_Car0335180	11799091	11812433	ercc4	DNA repair endonuclease XPF isoform X1 [Carassius auratus]	DNA repair endonuclease XPF OS=Homo sapiens OX=9606 GN=ERCC4 PE=1 SV=3	DNA repair endonuclease XPF isoform X1 OS=Carassius auratus OX=7957 GN=ercc4 PE=3 SV=1
gene_Car0335190	11832122	11859006	cpped1B	serine/threonine-protein phosphatase CPPED1 isoform X2 [Ctenopharyngodon idella]	Serine/threonine-protein phosphatase CPPED1 OS=Danio rerio OX=7955 GN=cpped1 PE=2 SV=1	Serine/threonine-protein phosphatase CPPED1 OS=Cyprinus carpio OX=7962 PE=3 SV=1
gene_Car0335200	11873704	11905890	shisa9aB	protein shisa-9A [Carassius auratus]	Protein shisa-9A OS=Danio rerio OX=7955 GN=shisa9a PE=2 SV=1	protein shisa-9A OS=Carassius auratus OX=7957 GN=LOC113111738 PE=4 SV=1
gene_Car0335210	11946596	12097788	snx29B	sorting nexin-29-like isoform X2 [Carassius gibelio]	Sorting nexin-29 OS=Bos taurus OX=9913 GN=SNX29 PE=2 SV=1	sorting nexin-29 isoform X2 OS=Carassius auratus OX=7957 GN=snx29 PE=4 SV=1
gene_Car0335220	12101482	12102932	NA	tumor necrosis factor receptor superfamily member 17-like [Cyprinus carpio]	--	Tumor necrosis factor receptor superfamily member 17-like OS=Cyprinus carpio OX=7962 GN=LOC109107798 PE=4 SV=1
gene_Car0335230	12103704	12104873	NA	hypothetical protein cypCar_00047611 [Cyprinus carpio]	Transmembrane protein 238 OS=Homo sapiens OX=9606 GN=TMEM238 PE=1 SV=1	Si:dkey-16i5.8 OS=Cyprinus carpio OX=7962 PE=4 SV=1
gene_Car0335240	12106868	12109716	rsl1d1	ribosomal L1 domain-containing protein 1 [Carassius auratus]	Ribosomal L1 domain-containing protein 1 OS=Bos taurus OX=9913 GN=RSL1D1 PE=2 SV=1	ribosomal L1 domain-containing protein 1 OS=Carassius auratus OX=7957 GN=rsl1d1 PE=4 SV=1
gene_Car0335250	12110842	12118816	gspt1B	eukaryotic peptide chain release factor GTP-binding subunit ERF3A [Carassius auratus]	Eukaryotic peptide chain release factor GTP-binding subunit ERF3A OS=Mus musculus OX=10090 GN=Gspt1 PE=1 SV=2	eukaryotic peptide chain release factor GTP-binding subunit ERF3A OS=Carassius auratus OX=7957 GN=gspt1 PE=4 SV=1
gene_Car0335260	12123679	12127806	tomm22B	mitochondrial import receptor subunit TOM22 homolog [Cyprinus carpio]	Mitochondrial import receptor subunit TOM22 homolog OS=Mus musculus OX=10090 GN=Tomm22 PE=1 SV=3	Mitochondrial import receptor subunit TOM22 homolog OS=Cyprinus carpio OX=7962 PE=3 SV=1
gene_Car0335270	12129139	12130117	maffB	transcription factor MafF-like [Carassius auratus]	Transcription factor MafF OS=Gallus gallus OX=9031 GN=MAFF PE=2 SV=1	transcription factor MafF-like OS=Carassius auratus OX=7957 GN=LOC113111733 PE=4 SV=1
gene_Car0335280	12131536	12140096	tmem184baB	transmembrane protein 184B isoform X1 [Cyprinus carpio]	Transmembrane protein 184B OS=Mus musculus OX=10090 GN=Tmem184b PE=2 SV=1	Transmembrane protein 184ba OS=Cyprinus carpio OX=7962 PE=4 SV=1
gene_Car0335290	12140630	12147798	csnk1eB	PREDICTED: casein kinase I-like [Sinocyclocheilus grahami]	Casein kinase I isoform epsilon OS=Mus musculus OX=10090 GN=Csnk1e PE=1 SV=2	Casein kinase I isoform X1 OS=Culter alburnus OX=194366 PE=2 SV=1
gene_Car0335300	12149449	12184141	tnrc6bB	trinucleotide repeat-containing gene 6B protein-like [Carassius auratus]	Trinucleotide repeat-containing gene 6B protein OS=Homo sapiens OX=9606 GN=TNRC6B PE=1 SV=4	trinucleotide repeat-containing gene 6B protein-like OS=Carassius auratus OX=7957 GN=LOC113111729 PE=4 SV=1
gene_Car0335310	12183142	12187415	si:ch211-141o9.10-2	uncharacterized protein LOC113111730 isoform X3 [Carassius auratus]	Apurinic-apyrimidinic endonuclease OS=Caenorhabditis elegans OX=6239 GN=apn-1 PE=2 SV=2	uncharacterized protein LOC113111730 isoform X3 OS=Carassius auratus OX=7957 GN=LOC113111730 PE=3 SV=1
gene_Car0335320	12204429	12214934	cdc42ep1aB	cdc42 effector protein 1-like [Carassius auratus]	Cdc42 effector protein 1 OS=Rattus norvegicus OX=10116 GN=Cdc42ep1 PE=1 SV=1	cdc42 effector protein 1-like OS=Carassius auratus OX=7957 GN=LOC113111727 PE=3 SV=1
gene_Car0335330	12211034	12223281	NA	SH3 domain-binding protein 1 [Carassius gibelio]	3 beta-hydroxysteroid dehydrogenase/Delta 5-->sp|Q5IFP1|3BHS_CANLF 3 beta-hydroxysteroid dehydrogenase/Delta 5-->sp|O46516|3BHS_HORSE 3 beta-hydroxysteroid dehydrogenase/Delta 5-->sp|Q9N119|3BHS_PIG 3 beta-hydroxysteroid dehydrogenase/Delta 5-->sp|Q9Y3L3|3BP1_HUMAN SH3 domain-binding protein 1 OS=Homo sapiens OX=9606 GN=SH3BP1 PE=1 SV=3	SH3 domain-binding protein 1-like OS=Carassius auratus OX=7957 GN=LOC113111726 PE=4 SV=1
gene_Car0335340	12225717	12229192	pdap1aB	28 kDa heat- and acid-stable phosphoprotein-like [Carassius auratus]	28 kDa heat- and acid-stable phosphoprotein OS=Homo sapiens OX=9606 GN=PDAP1 PE=1 SV=1	28 kDa heat- and acid-stable phosphoprotein-like OS=Carassius auratus OX=7957 GN=LOC113111725 PE=4 SV=1
gene_Car0335350	12230494	12233448	slc16a8B	monocarboxylate transporter 3-like [Carassius auratus]	Monocarboxylate transporter 3 OS=Gallus gallus OX=9031 GN=SLC16A8 PE=1 SV=3	monocarboxylate transporter 3-like OS=Carassius auratus OX=7957 GN=LOC113111724 PE=3 SV=1
gene_Car0335360	12234822	12236470	cby1	protein chibby homolog 1 [Carassius gibelio]	Protein chibby homolog 1 OS=Homo sapiens OX=9606 GN=CBY1 PE=1 SV=1	protein chibby homolog 1 OS=Carassius auratus OX=7957 GN=cby1 PE=4 SV=1
gene_Car0335370	12237392	12251797	zc3h7b-2B	zinc finger CCCH domain-containing protein 7B isoform X1 [Carassius gibelio]	Zinc finger CCCH domain-containing protein 7B OS=Homo sapiens OX=9606 GN=ZC3H7B PE=1 SV=2	zinc finger CCCH domain-containing protein 7B-like isoform X1 OS=Carassius auratus OX=7957 GN=LOC113111723 PE=4 SV=1
gene_Car0335380	12254061	12259127	tefaB	thyrotroph embryonic factor-like isoform X2 [Carassius auratus]	Thyrotroph embryonic factor OS=Mus musculus OX=10090 GN=Tef PE=2 SV=1	thyrotroph embryonic factor-like isoform X2 OS=Carassius auratus OX=7957 GN=LOC113111722 PE=3 SV=1
gene_Car0335390	12260204	12266235	phf5aB	PHD finger-like domain-containing protein 5A [Carassius gibelio]	PHD finger-like domain-containing protein 5A OS=Homo sapiens OX=9606 GN=PHF5A PE=1 SV=1	PHD finger-like domain-containing protein 5A OS=Tigriopus californicus OX=6832 GN=TCAL_09746 PE=3 SV=1
gene_Car0335400	12277796	12286328	aco2B	aconitate hydratase, mitochondrial-like [Carassius auratus]	Aconitate hydratase, mitochondrial OS=Bos taurus OX=9913 GN=ACO2 PE=1 SV=4	Aconitate hydratase, mitochondrial OS=Carassius auratus OX=7957 GN=LOC113111721 PE=3 SV=1
gene_Car0335410	12292190	12315615	si:ch73-139e5.4-2	heat-stable enterotoxin receptor-like [Carassius auratus]	Guanylyl cyclase C OS=Sus scrofa OX=9823 GN=GUCY2C PE=2 SV=2	Guanylate cyclase OS=Carassius auratus OX=7957 GN=LOC113111718 PE=3 SV=1
gene_Car0335420	12322222	12331793	kctd17B	PREDICTED: BTB/POZ domain-containing protein KCTD5-like isoform X2 [Sinocyclocheilus grahami]	BTB/POZ domain-containing protein KCTD5 OS=Bos taurus OX=9913 GN=KCTD5 PE=2 SV=1	BTB/POZ domain-containing protein KCTD5-like OS=Sinocyclocheilus grahami OX=75366 GN=LOC107584096 PE=4 SV=1
gene_Car0335430	12336949	12344829	rangap1bB	ran GTPase-activating protein 1-like isoform X2 [Carassius auratus]	Ran GTPase-activating protein 1 OS=Mus musculus OX=10090 GN=Rangap1 PE=1 SV=2	ran GTPase-activating protein 1-like isoform X2 OS=Carassius auratus OX=7957 GN=LOC113111717 PE=4 SV=1
gene_Car0335440	12345392	12355624	chadlbB	chondroadherin-like protein [Carassius auratus]	--	chondroadherin-like protein OS=Carassius auratus OX=7957 GN=chadl PE=4 SV=1
gene_Car0335450	12355188	12362271	l3mbtl2	lethal(3)malignant brain tumor-like protein 2 isoform X1 [Carassius auratus]	Lethal(3)malignant brain tumor-like protein 2 OS=Pongo abelii OX=9601 GN=L3MBTL2 PE=2 SV=1	lethal(3)malignant brain tumor-like protein 2 isoform X1 OS=Carassius auratus OX=7957 GN=LOC113111715 PE=4 SV=1

gene_Car0335460	12364987	12388535	ep300a-2B	histone acetyltransferase p300 isoform X6 [Carassius gibelio]	Histone acetyltransferase p300 OS=Homo sapiens OX=9606 GN=EP300 PE=1 SV=2	histone acetyltransferase OS=Cyprinus carpio OX=7962 PE=4 SV=1
gene_Car0335470	12388766	12393180	cbx7bB	hypothetical protein cypCar_00011623 [Cyprinus carpio]	Chromobox protein homolog 7 OS=Homo sapiens OX=9606 GN=CBX7 PE=1 SV=1	Chromobox protein homolog 7-like OS=Sinocyclocheilus grahami OX=75366 GN=LOC107554058 PE=4 SV=1
gene_Car0335480	12395382	12404795	josd1B	josephin-1-like [Carassius auratus]	Josephin-1 OS=Homo sapiens OX=9606 GN=JOSD1 PE=1 SV=1	ubiquitinyl hydrolase 1 OS=Carassius auratus OX=7957 GN=LOC113111709 PE=4 SV=1
gene_Car0335490	12404292	12410637	xrcc6	X-ray repair cross-complementing protein 6 [Carassius gibelio]	X-ray repair cross-complementing protein 5 OS=Gallus gallus OX=9031 GN=XRCC6 PE=2 SV=1	X-ray repair cross-complementing protein 6 OS=Carassius auratus OX=7957 GN=xrcc6 PE=3 SV=1
gene_Car0335500	12411716	12415849	desi1bB	desumoylating isopeptidase 1-like isoform X1 [Carassius auratus]	Desumoylating isopeptidase 1 OS=Mus musculus OX=10090 GN=Desi1 PE=1 SV=1	desumoylating isopeptidase 1-like isoform X1 OS=Carassius auratus OX=7957 GN=LOC113111710 PE=3 SV=1
gene_Car0335510	12438227	12455109	shisa8b	protein shisa-9 [Carassius gibelio]	--	protein shisa-9-like OS=Carassius auratus OX=7957 GN=LOC113112250 PE=4 SV=1
gene_Car0335520	12459679	12491455	mkl1bB	myocardin related transcription factor Ab isoform X6 [Carassius gibelio]	Myocardin-related transcription factor A OS=Mus musculus OX=10090 GN=Mrtfa PE=1 SV=2	MKL/myocardin-like protein 1 isoform X4 OS=Carassius auratus OX=7957 GN=LOC113112108 PE=4 SV=1
gene_Car0335530	12491990	12504403	rgs9bB	regulator of G-protein signaling 9-like isoform X1 [Carassius auratus]	Regulator of G-protein signaling 9 OS=Homo sapiens OX=9606 GN=RGS9 PE=1 SV=1	Regulator of G-protein signaling 9 OS=Carassius auratus OX=7957 GN=LOC113112107 PE=4 SV=1
gene_Car0335540	12510624	12518529	gna13aB	guanine nucleotide-binding protein subunit alpha-13-like isoform X1 [Carassius auratus]	Guanine nucleotide-binding protein subunit alpha-13 OS=Homo sapiens OX=9606 GN=GNA13 PE=1 SV=2	guanine nucleotide-binding protein subunit alpha-13-like isoform X1 OS=Carassius auratus OX=7957 GN=LOC113112106 PE=3 SV=1
gene_Car0335550	12518907	12523817	LOC103911930	archaemetzincin-2 [Carassius auratus]	Archaemetzincin-2 OS=Homo sapiens OX=9606 GN=AMZ2 PE=2 SV=2	archaemetzincin-2 OS=Carassius auratus OX=7957 GN=amz2 PE=3 SV=1
gene_Car0335560	12535588	12536576	LOC103911912B	monocarboxylate transporter 7 isoform X4 [Ctenopharyngodon idella]	Monocarboxylate transporter 7 OS=Homo sapiens OX=9606 GN=SLC16A6 PE=1 SV=2	Solute carrier family 16 member 6 OS=Sinocyclocheilus anshuiensis OX=1608454 PE=4 SV=1
gene_Car0335570	12541672	12545842	arsg	arylsulfatase G-like isoform X2 [Carassius auratus]	Arylsulfatase G OS=Rattus norvegicus OX=10116 GN=Arsg PE=2 SV=1	arylsulfatase G-like isoform X2 OS=Carassius auratus OX=7957 GN=LOC113112101 PE=3 SV=1
gene_Car0335580	12546755	12566080	snx11B	uncharacterized protein LOC113112100 isoform X2 [Carassius auratus]	Sorting nexin-11 OS=Bos taurus OX=9913 GN=SNX11 PE=2 SV=1	uncharacterized protein LOC113112100 isoform X2 OS=Carassius auratus OX=7957 GN=LOC113112100 PE=3 SV=1
gene_Car0335590	12566803	12571782	si:dkey-220f10B	tubby protein homolog [Carassius gibelio]	Tubby protein homolog OS=Homo sapiens OX=9606 GN=TUB PE=1 SV=1	tubby protein homolog OS=Carassius auratus OX=7957 GN=LOC113112098 PE=3 SV=1
gene_Car0335600	12572413	12578227	stx4B	syntaxin-4-like [Carassius auratus]	Syntaxin-4 OS=Homo sapiens OX=9606 GN=STX4 PE=1 SV=2	syntaxin-4-like OS=Carassius auratus OX=7957 GN=LOC113112104 PE=3 SV=1
gene_Car0335610	12578876	12585632	calcoco2B	calcium-binding and coiled-coil domain-containing protein 2-like [Carassius auratus]	Calcium-binding and coiled-coil domain-containing protein 2 OS=Macaca fascicularis OX=9541 GN=CALCOCO2 PE=2 SV=1	calcium-binding and coiled-coil domain-containing protein 2-like OS=Carassius auratus OX=7957 GN=LOC113112102 PE=4 SV=1
gene_Car0335620	12584963	12588342	snf8B	vacuolar-sorting protein SNF8 [Cyprinus carpio]	Vacuolar-sorting protein SNF8 OS=Danio rerio OX=7955 GN=snf8 PE=2 SV=2	Vacuolar-sorting protein SNF8 OS=Cyprinus carpio OX=7962 GN=LOC109100116 PE=3 SV=1
gene_Car0335630	12591120	12592656	gipB	hypothetical protein cypCar_00046544 [Cyprinus carpio]	--	Gastric inhibitory polypeptide OS=Cyprinus carpio OX=7962 PE=3 SV=1
gene_Car0335640	12607998	12613123	NA	alpha-N-acetylgalactosaminide alpha-2,6-sialyltransferase 1-like [Carassius auratus]	Alpha-N-acetylgalactosaminide alpha-2,6-sialyltransferase 1 OS=Gallus gallus OX=9031 GN=ST6GALNAC1 PE=1 SV=1	alpha-N-acetylgalactosaminide alpha-2,6-sialyltransferase 1-like OS=Carassius auratus OX=7957 GN=LOC113112097 PE=3 SV=1
gene_Car0335650	12617465	12671673	LOC564253B	caskin-2 isoform X1 [Carassius gibelio]	Caskin-2 OS=Homo sapiens OX=9606 GN=CASKIN2 PE=1 SV=2	caskin-2-like isoform X1 OS=Carassius auratus OX=7957 GN=LOC113112092 PE=4 SV=1
gene_Car0335660	12672997	12678565	nme2aB	nucleoside diphosphate kinase A-like isoform X1 [Carassius auratus]	Nucleoside diphosphate kinase OS=Gallus gallus OX=9031 PE=2 SV=1	Nucleoside diphosphate kinase OS=Carassius auratus OX=7957 GN=LOC113112093 PE=3 SV=1
gene_Car0335670	12995577	13155416	ca10aB	PREDICTED: carbonic anhydrase-related protein 10 isoform X1 [Sinocyclocheilus anshuiensis]	Carbonic anhydrase-related protein 10 OS=Homo sapiens OX=9606 GN=CA10 PE=2 SV=1	Carbonic anhydrase-related protein 10 OS=Cyprinus carpio OX=7962 GN=LOC109099532 PE=3 SV=1
gene_Car0335680	13212082	13216157	eme1B	crossover junction endonuclease EME1-like [Carassius auratus]	Crossover junction endonuclease EME1 OS=Pongo abelii OX=9601 GN=EME1 PE=3 SV=1	crossover junction endonuclease EME1-like OS=Carassius auratus OX=7957 GN=LOC113111870 PE=3 SV=1
gene_Car0335690	13217025	13218285	NA	crossover junction endonuclease EME1-like [Carassius auratus]	Crossover junction endonuclease EME1 OS=Homo sapiens OX=9606 GN=EME1 PE=1 SV=2	crossover junction endonuclease EME1-like OS=Carassius auratus OX=7957 GN=LOC113111870 PE=3 SV=1
gene_Car0335700	13219227	13222839	mrpl27B	39S ribosomal protein L27, mitochondrial-like [Carassius auratus]	39S ribosomal protein L27, mitochondrial OS=Homo sapiens OX=9606 GN=MRPL27 PE=1 SV=1	39S ribosomal protein L27, mitochondrial-like OS=Carassius auratus OX=7957 GN=LOC113111869 PE=3 SV=1
gene_Car0335710	13270150	13428538	thrabB	thyroid hormone receptor alpha isoform X1 [Carassius auratus]	Thyroid hormone receptor alpha OS=Salmo salar OX=8030 GN=thra1 PE=2 SV=1	Thyroid hormone receptor alpha-like OS=Cyprinus carpio OX=7962 GN=LOC109100142 PE=3 SV=1
gene_Car0335720	13488412	13496159	ormdl3B	PREDICTED: ORM1-like protein 3 [Sinocyclocheilus rhinocerous]	ORM1-like protein 3 OS=Danio rerio OX=7955 GN=ormdl3 PE=2 SV=1	ORM1-like protein OS=Cyprinus carpio OX=7962 GN=LOC109099472 PE=3 SV=1
gene_Car0335730	13508823	13527952	wnt3B	proto-oncogene Wnt-3 [Labeo rohita]	Proto-oncogene Wnt-3 OS=Homo sapiens OX=9606 GN=WNT3 PE=1 SV=2	Protein Wnt OS=Sinocyclocheilus anshuiensis OX=1608454 GN=LOC107657278 PE=3 SV=1
gene_Car0335740	13536397	13552916	NA	vesicle-fusing ATPase-like [Cyprinus carpio]	Vesicle-fusing ATPase OS=Cricetulus griseus OX=10029 GN=NSF PE=1 SV=1	Vesicle-fusing ATPase OS=Cyprinus carpio OX=7962 PE=3 SV=1
gene_Car0335750	13559167	13572297	si:dkey-38p12.3-2B	rho guanine nucleotide exchange factor 15-like [Carassius auratus]	Rho guanine nucleotide exchange factor 15 OS=Homo sapiens OX=9606 GN=ARHGEF15 PE=1 SV=4	rho guanine nucleotide exchange factor 15-like OS=Carassius auratus OX=7957 GN=LOC113111863 PE=4 SV=1
gene_Car0335760	13577817	13590166	hdlbpbB	vigilin-like [Carassius auratus]	Vigilin OS=Homo sapiens OX=9606 GN=HDLBP PE=1 SV=2	vigilin-like OS=Carassius auratus OX=7957 GN=LOC113111862 PE=4 SV=1

gene_Car0335770	13591712	13611412	neurl4B	neuralized-like protein 4 [Carassius auratus]	Neuralized-like protein 4 OS=Mus musculus OX=10090 GN=Neurl4 PE=1 SV=1	neuralized-like protein 4 OS=Carassius auratus OX=7957 GN=LOC113111861 PE=4 SV=1
gene_Car0335780	13612420	13617792	shbgB	sex hormone-binding globulin-like [Carassius auratus]	Sex hormone-binding globulin OS=Mus musculus OX=10090 GN=Shbg PE=2 SV=1	sex hormone-binding globulin-like OS=Carassius auratus OX=7957 GN=LOC113111860 PE=4 SV=1
gene_Car0335790	13622571	13630086	zbtb4B	zinc finger and BTB domain-containing protein 38-like [Carassius auratus]	Zinc finger and BTB domain-containing protein 4 OS=Mus musculus OX=10090 GN=Zbtb4 PE=2 SV=1	zinc finger and BTB domain-containing protein 38-like OS=Carassius auratus OX=7957 GN=LOC113111859 PE=4 SV=1
gene_Car0335800	13641555	13655000	polr2aB	DNA-directed RNA polymerase II subunit RPB1 [Carassius auratus]	DNA-directed RNA polymerase II subunit RPB1 OS=Cricetulus griseus OX=10029 GN=POLR2A PE=1 SV=2	DNA-directed RNA polymerase subunit OS=Carassius auratus OX=7957 GN=LOC113111858 PE=3 SV=1
gene_Car0335810	13663868	13669216	capgbB	macrophage-capping protein-like [Carassius auratus]	Macrophage-capping protein OS=Rattus norvegicus OX=10116 GN=Capg PE=1 SV=1	macrophage-capping protein-like OS=Carassius auratus OX=7957 GN=LOC113111857 PE=4 SV=1
gene_Car0335820	13672840	13677634	si:dkey-219e21B	E3 ubiquitin-protein ligase TRIM39-like [Cyprinus carpio]	E3 ubiquitin-protein ligase TRIM39 OS=Mus musculus OX=10090 GN=Trim39 PE=2 SV=1	Si:dkey-219e21.2 OS=Cyprinus carpio OX=7962 PE=4 SV=1
gene_Car0335830	13678784	13679602	NA	--	--	--
gene_Car0335840	13686889	13695922	mfsd8B	major facilitator superfamily domain-containing protein 8-like [Carassius auratus]	Major facilitator superfamily domain-containing protein 8 OS=Danio rerio OX=7955 GN=mfsd8 PE=2 SV=1	major facilitator superfamily domain-containing protein 8-like OS=Carassius auratus OX=7957 GN=LOC113111853 PE=4 SV=1
gene_Car0335850	13695983	13702247	NA	tripartite motif-containing protein 14 isoform X1 [Carassius gibelio]	E3 ubiquitin-protein ligase TRIM11 OS=Rattus norvegicus OX=10116 GN=Trim11 PE=2 SV=1	tripartite motif-containing protein 14-like OS=Carassius auratus OX=7957 GN=LOC113111852 PE=4 SV=1
gene_Car0335860	13706286	13709575	cdca9B	borealin-2 [Carassius gibelio]	Borealin-2 OS=Danio rerio OX=7955 GN=cdca9 PE=2 SV=1	borealin-2-like OS=Carassius auratus OX=7957 GN=LOC113112233 PE=3 SV=1
gene_Car0335870	13710294	13784019	ablim2B	actin-binding LIM protein 2 isoform X2 [Carassius gibelio]	Actin-binding LIM protein 2 OS=Homo sapiens OX=9606 GN=ABLIM2 PE=1 SV=2	actin-binding LIM protein 2-like isoform X2 OS=Carassius auratus OX=7957 GN=LOC113111849 PE=4 SV=1
gene_Car0335880	13801100	13835542	afap1B	actin filament-associated protein 1 isoform X3 [Carassius gibelio]	Actin filament-associated protein 1 OS=Gallus gallus OX=9031 GN=AFAP1 PE=1 SV=2	actin filament-associated protein 1-like isoform X3 OS=Carassius auratus OX=7957 GN=LOC113111848 PE=4 SV=1
gene_Car0335890	13838410	13847743	rab18aB	ras-related protein Rab-18-B-like [Carassius auratus]	Ras-related protein Rab-18 OS=Gallus gallus OX=9031 GN=RAB18 PE=2 SV=1	ras-related protein Rab-18-B-like OS=Carassius auratus OX=7957 GN=LOC113111847 PE=4 SV=1
gene_Car0335900	13863807	13891353	mkxaB	homeobox protein Mohawk-like [Carassius auratus]	Homeobox protein Mohawk OS=Homo sapiens OX=9606 GN=MKX PE=2 SV=2	homeobox protein Mohawk-like OS=Carassius auratus OX=7957 GN=LOC113111846 PE=4 SV=1
gene_Car0335910	13899758	13954642	armc4-2B	hypothetical protein cypCar_00003445 [Cyprinus carpio]	Outer dynein arm-docking complex subunit 2 OS=Bos taurus OX=9913 GN=ODAD2 PE=1 SV=3	Armadillo repeat containing 4 OS=Cyprinus carpio OX=7962 PE=4 SV=1
gene_Car0335920	13973718	14111026	mpp7aB	MAGUK p55 subfamily member 7 isoform X1 [Carassius gibelio]	MAGUK p55 subfamily member 7 OS=Danio rerio OX=7955 GN=mpp7 PE=1 SV=1	MAGUK p55 subfamily member 7 OS=Carassius auratus OX=7957 GN=LOC113111844 PE=3 SV=1
gene_Car0335930	14114318	14149537	wacaB	WW domain-containing adapter protein with coiled-coil isoform X2 [Danio rerio]	WW domain-containing adapter protein with coiled-coil OS=Danio rerio OX=7955 GN=waca PE=2 SV=1	WW domain-containing adapter protein with coiled-coil isoform X2 OS=Danio rerio OX=7955 GN=waca PE=1 SV=1
gene_Car0335940	14152751	14156973	bambiaB	BMP and activin membrane-bound inhibitor homolog [Carassius auratus]	BMP and activin membrane-bound inhibitor homolog OS=Homo sapiens OX=9606 GN=BAMBI PE=1 SV=1	BMP and activin membrane-bound inhibitor homolog OS=Carassius auratus OX=7957 GN=LOC113111841 PE=3 SV=1
gene_Car0335950	14245230	14253267	map3k8B	mitogen-activated protein kinase kinase kinase 8 isoform X1 [Carassius gibelio]	Mitogen-activated protein kinase kinase kinase 8 OS=Homo sapiens OX=9606 GN=MAP3K8 PE=1 SV=2	mitogen-activated protein kinase kinase kinase 8-like OS=Carassius auratus OX=7957 GN=LOC113111840 PE=4 SV=1
gene_Car0335960	14254569	14272494	mtpapB	poly(A) RNA polymerase, mitochondrial-like [Carassius auratus]	Poly(A) RNA polymerase, mitochondrial OS=Mus musculus OX=10090 GN=Mtpap PE=1 SV=1	poly(A) RNA polymerase, mitochondrial-like OS=Carassius auratus OX=7957 GN=LOC113111839 PE=4 SV=1
gene_Car0335970	14295054	14316119	jcadaB	junctional cadherin 5-associated protein [Carassius gibelio]	Junctional cadherin 5-associated protein OS=Homo sapiens OX=9606 GN=JCAD PE=1 SV=3	junctional protein associated with coronary artery disease-like OS=Carassius auratus OX=7957 GN=LOC113111838 PE=4 SV=1
gene_Car0335980	14345506	14389952	svilaB	supervillin isoform X12 [Carassius gibelio]	Supervillin OS=Bos taurus OX=9913 GN=SVIL PE=1 SV=2	supervillin-like isoform X19 OS=Carassius auratus OX=7957 GN=LOC113111835 PE=3 SV=1
gene_Car0335990	14392792	14422219	psme4bB	proteasome activator complex subunit 4B-like isoform X1 [Carassius auratus]	Proteasome activator complex subunit 4B OS=Danio rerio OX=7955 GN=psme4b PE=3 SV=2	proteasome activator complex subunit 4B-like isoform X1 OS=Carassius auratus OX=7957 GN=LOC113111833 PE=3 SV=1
gene_Car0336000	14422548	14425047	chac2	putative glutathione-specific gamma-glutamylcyclotransferase 2 [Carassius gibelio]	Putative glutathione-specific gamma-glutamylcyclotransferase 2 OS=Danio rerio OX=7955 GN=chac2 PE=2 SV=1	Gamma-glutamylcyclotransferase OS=Sinocyclocheilus rhinocerous OX=307959 GN=LOC107749368 PE=3 SV=1
gene_Car0336010	14425742	14432194	asb3B	ankyrin repeat and SOCS box protein 3 [Carassius gibelio]	Ankyrin repeat and SOCS box protein 3 OS=Homo sapiens OX=9606 GN=ASB3 PE=1 SV=1	ankyrin repeat and SOCS box protein 3-like OS=Carassius auratus OX=7957 GN=LOC113111832 PE=4 SV=1
gene_Car0336020	14571919	14743004	nrxn1aB	neurexin-1a isoform X11 [Carassius auratus]	Neurexin-1a OS=Danio rerio OX=7955 GN=nrxn1a PE=2 SV=1	neurexin-1a isoform X11 OS=Carassius auratus OX=7957 GN=LOC113111829 PE=4 SV=1
gene_Car0336030	14788981	14810328	foxn2bB	forkhead box protein N2-like [Carassius auratus]	Forkhead box protein N2 OS=Homo sapiens OX=9606 GN=FOXN2 PE=1 SV=3	forkhead box protein N2-like OS=Carassius auratus OX=7957 GN=LOC113111828 PE=4 SV=1
gene_Car0336040	14813586	14830931	fbxo11bB	F-box only protein 11-like isoform X1 [Carassius auratus]	F-box only protein 11 OS=Homo sapiens OX=9606 GN=FBXO11 PE=1 SV=3	F-box only protein 11-like isoform X1 OS=Carassius auratus OX=7957 GN=LOC113111827 PE=4 SV=1
gene_Car0336050	14831869	14840433	msh2B	hypothetical protein cypCar_00034928 [Cyprinus carpio]	DNA mismatch repair protein Msh2 OS=Chlorocebus aethiops OX=9534 GN=MSH2 PE=2 SV=1	DNA mismatch repair protein OS=Carassius auratus OX=7957 GN=LOC113111826 PE=3 SV=1
gene_Car0336060	14850258	14854551	calm2bB	hypothetical protein JZ751_011150 [Albula glossodonta]	Neo-calmodulin (Fragment) OS=Gallus gallus OX=9031 PE=2 SV=1	Calmodulin 1 OS=Myotis myotis OX=51298 GN=mMyoMyo1_002013 PE=4 SV=1
gene_Car0336070	14898757	14900343	socs5a-2B	suppressor of cytokine signaling 5-like [Carassius gibelio]	Suppressor of cytokine signaling 5 OS=Bos taurus OX=9913 GN=SOCS5 PE=2 SV=1	suppressor of cytokine signaling 5-like OS=Carassius auratus OX=7957 GN=LOC113111821 PE=4 SV=1
gene_Car0336080	14922230	14927431	gch2B	GTP cyclohydrolase 1-like [Carassius auratus]	GTP cyclohydrolase 1 OS=Gallus gallus OX=9031 GN=GCH1 PE=2 SV=1	GTP cyclohydrolase 1 OS=Carassius auratus OX=7957 GN=LOC113111822 PE=3 SV=1

gene_Car0336090	14926479	14927996	criptB	cysteine-rich PDZ-binding protein isoform X2 [Megalobrama amblycephala]	Cysteine-rich PDZ-binding protein OS=Danio rerio OX=7955 GN=cript PE=3 SV=1	Cysteine-rich PDZ-binding protein OS=Cyprinus carpio OX=7962 GN=LOC109091722 PE=3 SV=1
gene_Car0336100	14928628	14934608	pigfB	phosphatidylinositol-glycan biosynthesis class F protein-like [Carassius auratus]	Phosphatidylinositol-glycan biosynthesis class F protein OS=Homo sapiens OX=9606 GN=PIGF PE=1 SV=1	phosphatidylinositol-glycan biosynthesis class F protein-like OS=Carassius auratus OX=7957 GN=LOC113111818 PE=4 SV=1
gene_Car0336110	14932009	14952152	rhoqB	PREDICTED: rho-related GTP-binding protein RhoQ [Sinocyclocheilus grahami]	Rho-related GTP-binding protein RhoQ OS=Homo sapiens OX=9606 GN=RHOQ PE=1 SV=2	Rho-related GTP-binding protein RhoQ OS=Sinocyclocheilus anshuiensis OX=1608454 GN=LOC107698825 PE=4 SV=1
gene_Car0336120	14969954	14973390	cox7a3B	cytochrome c oxidase subunit 7A-related protein, mitochondrial isoform X1 [Carassius gibelio]	Cytochrome c oxidase subunit 7A-related protein, mitochondrial OS=Homo sapiens OX=9606 GN=COX7A2L PE=1 SV=2	Uncharacterized protein OS=Onychostoma macrolepis OX=369639 GN=G5714_012552 PE=3 SV=1
gene_Car0336130	14974326	14980976	NA	potassium voltage-gated channel subfamily G member 3-like [Carassius auratus]	Potassium voltage-gated channel subfamily G member 3 OS=Homo sapiens OX=9606 GN=KCNG3 PE=1 SV=1	potassium voltage-gated channel subfamily G member 3-like OS=Carassius auratus OX=7957 GN=LOC113111562 PE=4 SV=1
gene_Car0336140	14981821	15007024	mta3B	metastasis-associated protein MTA3 isoform X1 [Megalobrama amblycephala]	Metastasis-associated protein MTA3 OS=Bos taurus OX=9913 GN=MTA3 PE=2 SV=1	Metastasis associated 1 family, member 3 OS=Cyprinus carpio OX=7962 PE=4 SV=1
gene_Car0336150	15015796	15023296	hcar1-4B	hydroxycarboxylic acid receptor 3 [Carassius gibelio]	Hydroxycarboxylic acid receptor 3 OS=Homo sapiens OX=9606 GN=HCAR3 PE=1 SV=3	hydroxycarboxylic acid receptor 3-like OS=Carassius auratus OX=7957 GN=LOC113111561 PE=3 SV=1
gene_Car0336160	15055808	15059272	zfp36l2B	mRNA decay activator protein ZFP36L2-A-like [Carassius gibelio]	mRNA decay activator protein ZFP36L2-A OS=Xenopus laevis OX=8355 GN=zfp36l2-A PE=1 SV=1	mRNA decay activator protein ZFP36 OS=Carassius auratus OX=7957 GN=LOC113111559 PE=4 SV=1
gene_Car0336170	15080597	15114483	ppm1bbB	protein phosphatase 1B-like isoform X1 [Carassius gibelio]	Protein phosphatase 1B OS=Homo sapiens OX=9606 GN=PPM1B PE=1 SV=1	Protein phosphatase, Mg2+/Mn2+ dependent, 1Bb OS=Cyprinus carpio OX=7962 PE=3 SV=1
gene_Car0336180	15161959	15164577	six3bB	homeobox protein SIX3-like [Carassius auratus]	Homeobox protein SIX3 OS=Homo sapiens OX=9606 GN=SIX3 PE=1 SV=1	Sine oculis homeobox homolog 3 OS=Carassius auratus OX=7957 GN=Six3 PE=2 SV=1
gene_Car0336190	15172046	15173746	six2bB	homeobox protein SIX2-like [Carassius auratus]	Homeobox protein six1a OS=Danio rerio OX=7955 GN=six1a PE=2 SV=2	homeobox protein SIX2-like OS=Carassius auratus OX=7957 GN=LOC113111554 PE=4 SV=1
gene_Car0336200	15187347	15248372	prkceaB	protein kinase C epsilon type-like [Carassius auratus]	Protein kinase C epsilon type OS=Mus musculus OX=10090 GN=Prkce PE=1 SV=1	Protein kinase C OS=Carassius auratus OX=7957 GN=LOC113111553 PE=3 SV=1
gene_Car0336210	15249261	15268892	epas1aB	endothelial PAS domain-containing protein 1-like isoform X2 [Carassius auratus]	Endothelial PAS domain-containing protein 1 OS=Homo sapiens OX=9606 GN=EPAS1 PE=1 SV=3	endothelial PAS domain-containing protein 1-like isoform X2 OS=Carassius auratus OX=7957 GN=LOC113111552 PE=4 SV=1
gene_Car0336220	15278261	15293518	glud1bB	glutamate dehydrogenase, mitochondrial [Carassius auratus]	Glutamate dehydrogenase, mitochondrial OS=Chaenocephalus aceratus OX=36190 GN=glud1 PE=1 SV=1	glutamate dehydrogenase [NAD(P)(+)] OS=Carassius auratus OX=7957 GN=LOC113111551 PE=3 SV=1
gene_Car0336230	15294240	15295219	NA	hypothetical protein ROHU_031284 [Labeo rohita]	--	Uncharacterized protein OS=Labeo rohita OX=84645 GN=ROHU_031284 PE=4 SV=1
gene_Car0336240	15309488	15319923	mmrn2bB	multimerin-2 isoform X1 [Carassius gibelio]	--	multimerin-2-like isoform X1 OS=Carassius auratus OX=7957 GN=LOC113111547 PE=4 SV=1
gene_Car0336250	15316828	15355839	bmpr1abB	bone morphogenetic protein receptor type-1A-like [Carassius auratus]	Bone morphogenetic protein receptor type-1A OS=Homo sapiens OX=9606 GN=BMPR1A PE=1 SV=2	receptor protein serine/threonine kinase OS=Carassius auratus OX=7957 GN=LOC113111548 PE=3 SV=1
gene_Car0336260	15356918	15372743	ldb3bB	LIM domain-binding protein 3-like isoform X2 [Carassius auratus]	LIM domain-binding protein 3 OS=Homo sapiens OX=9606 GN=LDB3 PE=1 SV=2	LIM domain-binding protein 3-like isoform X2 OS=Carassius auratus OX=7957 GN=LOC113111546 PE=4 SV=1
gene_Car0336270	15450964	15501014	usp54bB	inactive ubiquitin carboxyl-terminal hydrolase 54-like isoform X4 [Carassius auratus]	Inactive ubiquitin carboxyl-terminal hydrolase 54 OS=Homo sapiens OX=9606 GN=USP54 PE=1 SV=4	inactive ubiquitin carboxyl-terminal hydrolase 54-like isoform X4 OS=Carassius auratus OX=7957 GN=LOC113111542 PE=4 SV=1
gene_Car0336280	15518428	15580793	NA	inactive ubiquitin carboxyl-terminal hydrolase 54-like isoform X4 [Carassius auratus]	Inactive ubiquitin carboxyl-terminal hydrolase 54 OS=Homo sapiens OX=9606 GN=USP54 PE=1 SV=4	inactive ubiquitin carboxyl-terminal hydrolase 54-like isoform X1 OS=Carassius auratus OX=7957 GN=LOC113111542 PE=4 SV=1
gene_Car0336290	15582080	15586162	myoz1bB	myozenin-1-like [Carassius auratus]	Myozenin-1 OS=Mus musculus OX=10090 GN=Myoz1 PE=1 SV=1	myozenin-1-like OS=Carassius auratus OX=7957 GN=LOC113111541 PE=3 SV=1
gene_Car0336300	15590630	15602599	synpo2lbB	synaptopodin 2-like protein [Carassius gibelio]	Synaptopodin 2-like protein OS=Homo sapiens OX=9606 GN=SYNPO2L PE=1 SV=3	Synaptopodin 2-like protein OS=Cyprinus carpio OX=7962 GN=LOC109100177 PE=4 SV=1
gene_Car0336310	15605486	15621144	itgb3bB	integrin beta-3-like [Carassius auratus]	Integrin beta-3 OS=Mus musculus OX=10090 GN=Itgb3 PE=1 SV=2	Integrin beta OS=Carassius auratus OX=7957 GN=LOC113111535 PE=3 SV=1
gene_Car0336320	15622913	15633699	ndel1aB	nuclear distribution protein nudE-like 1-A isoform X2 [Carassius auratus]	Nuclear distribution protein nudE-like 1-A OS=Danio rerio OX=7955 GN=ndel1a PE=2 SV=1	nuclear distribution protein nudE-like 1-A isoform X2 OS=Carassius auratus OX=7957 GN=LOC113111537 PE=3 SV=1
gene_Car0336330	15635394	15637477	armc7	armadillo repeat-containing protein 7 [Cyprinus carpio]	Armadillo repeat-containing protein 7 OS=Mus musculus OX=10090 GN=Armc7 PE=1 SV=2	Armadillo repeat containing 7 OS=Cyprinus carpio OX=7962 GN=armc7 PE=4 SV=1
gene_Car0336340	15648011	15655207	NA	chondroadherin [Carassius gibelio]	Chondroadherin OS=Rattus norvegicus OX=10116 GN=Chad PE=2 SV=1	Chondroadherin OS=Carassius auratus auratus OX=145527 GN=chad PE=2 SV=1
gene_Car0336350	15669677	15673159	NA	hypothetical protein cypCar_00021318 [Cyprinus carpio]	ABC-type oligopeptide transporter ABCB9 OS=Mus musculus OX=10090 GN=Abcb9 PE=1 SV=1	Transporter associated with antigen processing, subunit type t, teleost specific OS=Cyprinus carpio OX=7962 PE=4 SV=1
gene_Car0336360	15673539	15682575	slc16a5aB	monocarboxylate transporter 6-like [Carassius auratus]	Monocarboxylate transporter 6 OS=Homo sapiens OX=9606 GN=SLC16A5 PE=1 SV=1	monocarboxylate transporter 6-like OS=Carassius auratus OX=7957 GN=LOC113111532 PE=4 SV=1
gene_Car0336370	15683325	15696107	kctd2B	BTB/POZ domain-containing protein KCTD2 [Carassius gibelio]	BTB/POZ domain-containing protein KCTD2 OS=Homo sapiens OX=9606 GN=KCTD2 PE=1 SV=3	BTB/POZ domain-containing protein KCTD2 OS=Carassius auratus OX=7957 GN=kctd2 PE=4 SV=1
gene_Car0336380	15697009	15700643	hexdcB	hexosaminidase D [Carassius gibelio]	Hexosaminidase D OS=Homo sapiens OX=9606 GN=HEXD PE=1 SV=3	beta-N-acetylhexosaminidase OS=Carassius auratus OX=7957 GN=hexd PE=3 SV=1
gene_Car0336390	15701589	15752775	arhgap12bB	rho GTPase-activating protein 12-like isoform X1 [Carassius auratus]	Rho GTPase-activating protein 12 OS=Macaca fascicularis OX=9541 GN=ARHGAP12 PE=2 SV=1	rho GTPase-activating protein 12-like isoform X1 OS=Carassius auratus OX=7957 GN=LOC113111529 PE=4 SV=1
gene_Car0336400	15761481	15786237	zeb1bB	zinc finger E-box-binding homeobox 1-like isoform X2 [Carassius auratus]	Zinc finger E-box-binding homeobox 1 OS=Homo sapiens OX=9606 GN=ZEB1 PE=1 SV=2	zinc finger E-box-binding homeobox 1-like isoform X2 OS=Carassius

						auratus OX=7957 GN=LOC113111527 PE=3 SV=1
gene_Car0336410	15842354	15894669	znf438B	zinc finger protein 438 isoform X1 [Carassius gibelio]	Zinc finger protein 438 OS=Homo sapiens OX=9606 GN=ZNF438 PE=1 SV=1	zinc finger protein 438-like isoform X1 OS=Carassius auratus OX=7957 GN=LOC113111526 PE=4 SV=1
gene_Car0336420	15916393	15921550	msl1bB	male-specific lethal 1 homolog [Carassius auratus]	Male-specific lethal 1 homolog OS=Homo sapiens OX=9606 GN=MSL1 PE=1 SV=3	male-specific lethal 1 homolog OS=Carassius auratus OX=7957 GN=LOC113111524 PE=4 SV=1
gene_Car0336430	15921894	15924678	bc2B	charged multivesicular body protein 2a [Carassius auratus]	Charged multivesicular body protein 2a OS=Danio rerio OX=7955 GN=chmp2a PE=2 SV=1	charged multivesicular body protein 2a OS=Carassius auratus OX=7957 GN=LOC113111525 PE=3 SV=1
gene_Car0336440	15926136	15944078	fbrsB	autism susceptibility gene 2 protein-like isoform X3 [Carassius auratus]	Probable fibrosin-1 OS=Homo sapiens OX=9606 GN=FBRS PE=1 SV=3	autism susceptibility gene 2 protein-like isoform X3 OS=Carassius auratus OX=7957 GN=LOC113111521 PE=4 SV=1
gene_Car0336450	15944194	15975076	srcapB	helicase SRCAP-like isoform X3 [Carassius auratus]	Helicase SRCAP OS=Homo sapiens OX=9606 GN=SRCAP PE=1 SV=3	helicase SRCAP-like isoform X3 OS=Carassius auratus OX=7957 GN=LOC113111520 PE=4 SV=1
gene_Car0336460	15977367	15983319	ttll6	tubulin polyglutamylase ttll6 isoform X1 [Carassius gibelio]	Tubulin polyglutamylase ttll6 OS=Danio rerio OX=7955 GN=ttll6 PE=2 SV=1	tubulin polyglutamylase TTLL6 isoform X1 OS=Carassius auratus OX=7957 GN=ttll6 PE=4 SV=1
gene_Car0336470	15985973	15986988	hoxb8bB	homeobox protein Hox-B8b [Carassius gibelio]	Homeobox protein Hox-B8b OS=Danio rerio OX=7955 GN=hoxb8b PE=2 SV=1	homeobox protein Hox-B8b OS=Carassius auratus OX=7957 GN=LOC113111518 PE=4 SV=1
gene_Car0336480	15994325	15995373	hoxb6bB	homeobox protein Hox-B6b-like [Carassius auratus]	Homeobox protein Hox-B6b OS=Danio rerio OX=7955 GN=hoxb6b PE=2 SV=2	homeobox protein Hox-B6b-like OS=Carassius auratus OX=7957 GN=LOC113111519 PE=3 SV=1
gene_Car0336490	15996797	15998052	hoxb5bB	homeobox protein Hox-B5b [Carassius auratus]	Homeobox protein Hox-B5b OS=Danio rerio OX=7955 GN=hoxb5b PE=2 SV=2	homeobox protein Hox-B5b OS=Carassius auratus OX=7957 GN=LOC113111516 PE=3 SV=1
gene_Car0336500	16007508	16009134	hoxb1bB	homeobox protein Hox-B1b-like [Carassius auratus]	Homeobox protein Hox-B1b OS=Danio rerio OX=7955 GN=hoxb1b PE=2 SV=3	homeobox protein Hox-B1b-like OS=Carassius auratus OX=7957 GN=LOC113111515 PE=4 SV=1

Supplementary Table 14. GO enrichment of the 292 genes in the hotspot region.

ID	Description	GeneRatio	BgRatio	pvalue	p.adjust	qvalue	geneID	Count
GO:0005833	hemoglobin complex	3/223	16/29370	0.0002248	0.0411413	0.0404669	gene_Car0335090/gene_Car0335100/gene_Car0335110	3
GO:0015671	oxygen transport	3/223	22/29370	0.0005978	0.054699	0.0538023	gene_Car0335090/gene_Car0335100/gene_Car0335110	3
GO:0019825	oxygen binding	3/223	27/29370	0.0011041	0.0673519	0.0662478	gene_Car0335090/gene_Car0335100/gene_Car0335110	3
GO:0008233	peptidase activity	3/223	41/29370	0.0037219	0.1702782	0.1674868	gene_Car0334720/gene_Car0335500/gene_Car0335550	3
GO:0043565	sequence-specific DNA binding	8/223	400/29370	0.0117882	0.4311178	0.4240502	gene_Car0334190/gene_Car0334430/gene_Car0334920/gene_Car0335130/gene_Car0335710/gene_Car0336030/gene_Car0336140/gene_Car0336500	8
GO:0004518	nuclease activity	2/223	26/29370	0.0165461	0.4311178	0.4240502	gene_Car0335180/gene_Car0335680	2
GO:0006506	GPI anchor biosynthetic process	2/223	27/29370	0.0177814	0.4311178	0.4240502	gene_Car0334050/gene_Car0336100	2
GO:0035091	phosphatidylinositol binding	4/223	133/29370	0.0188467	0.4311178	0.4240502	gene_Car0334940/gene_Car0334960/gene_Car0335210/gene_Car0335580	4
GO:0006281	DNA repair	4/223	152/29370	0.0289737	0.4427225	0.4354647	gene_Car0335180/gene_Car0335310/gene_Car0335680/gene_Car0335690	4
GO:0007010	cytoskeleton organization	2/223	36/29370	0.0305276	0.4427225	0.4354647	gene_Car0335870/gene_Car0335980	2
GO:0015267	channel activity	2/223	41/29370	0.0387693	0.4427225	0.4354647	gene_Car0335070/gene_Car0335080	2
GO:0020037	heme binding	4/223	169/29370	0.0403162	0.4427225	0.4354647	gene_Car0333800/gene_Car0335090/gene_Car0335100/gene_Car0335110	4
GO:0008168	methyltransferase activity	3/223	109/29370	0.0504784	0.4427225	0.4354647	gene_Car0334520/gene_Car0334570/gene_Car0335060	3
GO:0016579	protein deubiquitination	3/223	111/29370	0.0527614	0.4427225	0.4354647	gene_Car0334720/gene_Car0334800/gene_Car0335480	3
GO:0005789	endoplasmic reticulum membrane	2/223	57/29370	0.0697863	0.4427225	0.4354647	gene_Car0335720/gene_Car0336100	2
GO:0000290	deadenylation-dependent decapping of nuclear-transcribed mRNA	1/223	10/29370	0.0733963	0.4427225	0.4354647	gene_Car0334310	1
GO:0004692	cGMP-dependent protein kinase activity	1/223	10/29370	0.0733963	0.4427225	0.4354647	gene_Car0333540	1
GO:0005746	mitochondrial respirasome	1/223	10/29370	0.0733963	0.4427225	0.4354647	gene_Car0336120	1
GO:0015937	coenzyme A biosynthetic process	1/223	10/29370	0.0733963	0.4427225	0.4354647	gene_Car0334660	1
GO:0016651	oxidoreductase activity, acting on NAD(P)H	1/223	10/29370	0.0733963	0.4427225	0.4354647	gene_Car0334740	1
GO:0004656	procollagen-proline 4-dioxygenase activity	1/223	11/29370	0.0804341	0.4427225	0.4354647	gene_Car0334090	1
GO:0006183	GTP biosynthetic process	1/223	11/29370	0.0804341	0.4427225	0.4354647	gene_Car0335660	1
GO:0006228	UTP biosynthetic process	1/223	11/29370	0.0804341	0.4427225	0.4354647	gene_Car0335660	1
GO:0006298	mismatch repair	1/223	11/29370	0.0804341	0.4427225	0.4354647	gene_Car0336050	1
GO:0008028	monocarboxylic acid transmembrane transporter activity	1/223	11/29370	0.0804341	0.4427225	0.4354647	gene_Car0335350	1
GO:0015718	monocarboxylic acid transport	1/223	11/29370	0.0804341	0.4427225	0.4354647	gene_Car0335350	1
GO:0030018	Z disc	1/223	11/29370	0.0804341	0.4427225	0.4354647	gene_Car0336290	1
GO:0030983	mismatched DNA binding	1/223	11/29370	0.0804341	0.4427225	0.4354647	gene_Car0336050	1
GO:0048384	retinoic acid receptor signaling pathway	1/223	11/29370	0.0804341	0.4427225	0.4354647	gene_Car0334190	1
GO:0051260	protein homooligomerization	3/223	136/29370	0.0852668	0.4427225	0.4354647	gene_Car0335420/gene_Car0336130/gene_Car0336370	3
GO:0006265	DNA topological change	1/223	12/29370	0.0874188	0.4427225	0.4354647	gene_Car0334180	1
GO:0006383	transcription by RNA polymerase III	1/223	12/29370	0.0874188	0.4427225	0.4354647	gene_Car0333620	1
GO:0007623	circadian rhythm	1/223	12/29370	0.0874188	0.4427225	0.4354647	gene_Car0334650	1
GO:0016706	2-oxoglutarate-dependent dioxygenase activity	1/223	12/29370	0.0874188	0.4427225	0.4354647	gene_Car0334570	1
GO:0033209	tumor necrosis factor-mediated signaling pathway	1/223	12/29370	0.0874188	0.4427225	0.4354647	gene_Car0335220	1
GO:0046983	protein dimerization activity	6/223	403/29370	0.0878923	0.4427225	0.4354647	gene_Car0333830/gene_Car0334270/gene_Car0334280/gene_Car0334530/gene_Car0334860/gene_Car0336210	6
GO:0043547	positive regulation of GTPase activity	2/223	66/29370	0.0897909	0.4427225	0.4354647	gene_Car0335050/gene_Car0335330	2
GO:0004550	nucleoside diphosphate kinase activity	1/223	13/29370	0.0943507	0.4427225	0.4354647	gene_Car0335660	1
GO:0036402	proteasome-activating activity	1/223	13/29370	0.0943507	0.4427225	0.4354647	gene_Car0333960	1
GO:0006241	CTP biosynthetic process	1/223	14/29370	0.1012301	0.4474199	0.4400851	gene_Car0335660	1
GO:0004879	nuclear receptor activity	2/223	72/29370	0.1039461	0.4474199	0.4400851	gene_Car0334190/gene_Car0335710	2
GO:0006939	smooth muscle contraction	1/223	15/29370	0.1080576	0.4474199	0.4400851	gene_Car0334650	1
GO:0007605	sensory perception of sound	1/223	15/29370	0.1080576	0.4474199	0.4400851	gene_Car0333580	1
GO:0006751	glutathione catabolic process	1/223	16/29370	0.1148333	0.4474199	0.4400851	gene_Car0336000	1
GO:0006366	transcription by RNA polymerase II	1/223	17/29370	0.1215579	0.4474199	0.4400851	gene_Car0335800	1
GO:0000123	histone acetyltransferase complex	1/223	18/29370	0.1282316	0.4474199	0.4400851	gene_Car0335460	1
GO:0007266	Rho protein signal transduction	1/223	18/29370	0.1282316	0.4474199	0.4400851	gene_Car0335540	1
GO:0042310	vasoconstriction	1/223	18/29370	0.1282316	0.4474199	0.4400851	gene_Car0334650	1
GO:0140664	ATP-dependent DNA damage sensor activity	1/223	18/29370	0.1282316	0.4474199	0.4400851	gene_Car0336050	1
GO:0005730	nucleolus	1/223	19/29370	0.1348548	0.4474199	0.4400851	gene_Car0333890	1
GO:0007009	plasma membrane organization	1/223	19/29370	0.1348548	0.4474199	0.4400851	gene_Car0334880	1
GO:0008610	lipid biosynthetic process	1/223	19/29370	0.1348548	0.4474199	0.4400851	gene_Car0334700	1
GO:0051539	4 iron, 4 sulfur cluster binding	1/223	19/29370	0.1348548	0.4474199	0.4400851	gene_Car0335400	1
GO:0022857	transmembrane transporter activity	5/223	361/29370	0.1413297	0.4474199	0.4400851	gene_Car0333660/gene_Car0334670/gene_Car0335350/gene_Car0335840/gene_Car0336360	5
GO:0030163	protein catabolic process	1/223	20/29370	0.1414279	0.4474199	0.4400851	gene_Car0333960	1
GO:0000981	DNA-binding transcription factor activity, RNA polymerase II-specific	6/223	467/29370	0.1461687	0.4474199	0.4400851	gene_Car0335900/gene_Car0336190/gene_Car0336470/gene_Car0336480/gene_Car0336490/gene_Car0336500	6
GO:0003684	damaged DNA binding	1/223	21/29370	0.1479513	0.4474199	0.4400851	gene_Car0335490	1
GO:0032259	methylation	1/223	21/29370	0.1479513	0.4474199	0.4400851	gene_Car0335060	1
GO:0032958	inositol phosphate biosynthetic process	1/223	21/29370	0.1479513	0.4474199	0.4400851	gene_Car0333590	1
GO:0035556	intracellular signal transduction	5/223	367/29370	0.1483236	0.4474199	0.4400851	gene_Car0334990/gene_Car0335410/gene_Car0335530/gene_Car0336010/gene_Car0336070	5
GO:0005096	GTPase activator activity	3/223	178/29370	0.1538504	0.4474199	0.4400851	gene_Car0335050/gene_Car0335330/gene_Car0335430	3
GO:0030178	negative regulation of Wnt signaling pathway	1/223	22/29370	0.1544253	0.4474199	0.4400851	gene_Car0333550	1

GO:0004697	protein kinase C activity	1/223	24/29370	0.1672269	0.4474199	0.4400851	gene_Car0336200	1
GO:0001664	G protein-coupled receptor binding	1/223	25/29370	0.1735551	0.4474199	0.4400851	gene_Car0335540	1
GO:0004721	phosphoprotein phosphatase activity	1/223	25/29370	0.1735551	0.4474199	0.4400851	gene_Car0336170	1
GO:0005388	P-type calcium transporter activity	1/223	25/29370	0.1735551	0.4474199	0.4400851	gene_Car0334350	1
GO:0008484	sulfuric ester hydrolase activity	1/223	25/29370	0.1735551	0.4474199	0.4400851	gene_Car0335570	1
GO:0008033	tRNA processing	1/223	26/29370	0.1798355	0.4474199	0.4400851	gene_Car0334630	1
GO:0008277	regulation of G protein-coupled receptor signaling pathway	1/223	26/29370	0.1798355	0.4474199	0.4400851	gene_Car0335530	1
GO:0008509	monoatomic anion transmembrane transporter activity	1/223	26/29370	0.1798355	0.4474199	0.4400851	gene_Car0333980	1
GO:0030145	manganese ion binding	1/223	26/29370	0.1798355	0.4474199	0.4400851	gene_Car0336170	1
GO:0006886	intracellular protein transport	4/223	291/29370	0.1811332	0.4474199	0.4400851	gene_Car0333810/gene_Car0335260/gene_Car0335580 /gene_Car0335600	4
GO:0005125	cytokine activity	1/223	28/29370	0.192254	0.4474199	0.4400851	gene_Car0334150	1
GO:0005452	solute:inorganic anion antiporter activity	1/223	28/29370	0.192254	0.4474199	0.4400851	gene_Car0333980	1
GO:0016788	hydrolase activity, acting on ester bonds	1/223	28/29370	0.192254	0.4474199	0.4400851	gene_Car0334730	1
GO:0005506	iron ion binding	3/223	199/29370	0.1930102	0.4474199	0.4400851	gene_Car0333800/gene_Car0334090/gene_Car0334700	3
GO:0006520	amino acid metabolic process	1/223	29/29370	0.1983929	0.4474199	0.4400851	gene_Car0336220	1
GO:0006820	monoatomic anion transport	1/223	29/29370	0.1983929	0.4474199	0.4400851	gene_Car0333980	1
GO:0007034	vacuolar transport	1/223	29/29370	0.1983929	0.4474199	0.4400851	gene_Car0336430	1
GO:0016573	histone acetylation	1/223	29/29370	0.1983929	0.4474199	0.4400851	gene_Car0335460	1
GO:0004402	histone acetyltransferase activity	1/223	30/29370	0.2044853	0.4474199	0.4400851	gene_Car0335460	1
GO:0042254	ribosome biogenesis	1/223	30/29370	0.2044853	0.4474199	0.4400851	gene_Car0333890	1
GO:0003899	DNA-directed 5'-3' RNA polymerase activity	1/223	31/29370	0.2105317	0.4474199	0.4400851	gene_Car0335800	1
GO:0006123	mitochondrial electron transport, cytochrome c to oxygen	1/223	31/29370	0.2105317	0.4474199	0.4400851	gene_Car0336120	1
GO:0003697	single-stranded DNA binding	1/223	32/29370	0.2165323	0.4474199	0.4400851	gene_Car0335180	1
GO:0005751	mitochondrial respiratory chain complex IV	1/223	32/29370	0.2165323	0.4474199	0.4400851	gene_Car0336120	1
GO:1990904	ribonucleoprotein complex	1/223	32/29370	0.2165323	0.4474199	0.4400851	gene_Car0333890	1
GO:0051015	actin filament binding	2/223	115/29370	0.2174406	0.4474199	0.4400851	gene_Car0335810/gene_Car0335980	2
GO:0003682	chromatin binding	1/223	33/29370	0.2224875	0.4474199	0.4400851	gene_Car0336140	1
GO:0004089	carbonate dehydratase activity	1/223	33/29370	0.2224875	0.4474199	0.4400851	gene_Car0335670	1
GO:0004993	G protein-coupled serotonin receptor activity	1/223	33/29370	0.2224875	0.4474199	0.4400851	gene_Car0334650	1
GO:0005741	mitochondrial outer membrane	1/223	35/29370	0.234263	0.4539277	0.4464863	gene_Car0335260	1
GO:0031418	L-ascorbic acid binding	1/223	35/29370	0.234263	0.4539277	0.4464863	gene_Car0334090	1
GO:0046856	phosphatidylinositol dephosphorylation	1/223	35/29370	0.234263	0.4539277	0.4464863	gene_Car0334750	1
GO:0016702	oxidoreductase activity, acting on single donors with incorporation of molecular oxygen, incorporation of two atoms of oxygen	1/223	36/29370	0.240084	0.4539277	0.4464863	gene_Car0333750	1
GO:0008237	metallopeptidase activity	3/223	223/29370	0.240351	0.4539277	0.4464863	gene_Car0334450/gene_Car0334720/gene_Car0335550	3
GO:0004843	cysteine-type deubiquitinase activity	2/223	125/29370	0.2454036	0.4539277	0.4464863	gene_Car0334800/gene_Car0335480	2
GO:0042054	histone methyltransferase activity	1/223	37/29370	0.245861	0.4539277	0.4464863	gene_Car0334120	1
GO:0016887	ATP hydrolysis activity	4/223	333/29370	0.2475294	0.4539277	0.4464863	gene_Car0333960/gene_Car0334350/gene_Car0334760 /gene_Car0335740	4
GO:0006099	tricarboxylic acid cycle	1/223	38/29370	0.2515942	0.4539277	0.4464863	gene_Car0335400	1
GO:0006914	autophagy	1/223	38/29370	0.2515942	0.4539277	0.4464863	gene_Car0333770	1
GO:0016705	oxidoreductase activity, acting on paired donors, with incorporation or reduction of molecular oxygen	2/223	129/29370	0.2566345	0.4539277	0.4464863	gene_Car0333800/gene_Car0334090	2
GO:0046872	metal ion binding	5/223	453/29370	0.261952	0.4539277	0.4464863	gene_Car0333900/gene_Car0334080/gene_Car0334100 /gene_Car0335370/gene_Car0336160	5
GO:0038023	signaling receptor activity	2/223	131/29370	0.262254	0.4539277	0.4464863	gene_Car0334290/gene_Car0335220	2
GO:0007178	transmembrane receptor protein serine/threonine kinase signaling pathway	1/223	40/29370	0.2629308	0.4539277	0.4464863	gene_Car0336250	1
GO:0043169	cation binding	1/223	40/29370	0.2629308	0.4539277	0.4464863	gene_Car0336170	1
GO:0004675	transmembrane receptor protein serine/threonine kinase activity	1/223	41/29370	0.2685349	0.4592699	0.4517409	gene_Car0336250	1
GO:0005484	SNAP receptor activity	1/223	43/29370	0.279616	0.4717832	0.4640491	gene_Car0335600	1
GO:0007399	nervous system development	1/223	44/29370	0.2850937	0.4717832	0.4640491	gene_Car0334530	1
GO:0000723	telomere maintenance	1/223	46/29370	0.2959251	0.4717832	0.4640491	gene_Car0335490	1
GO:0003713	transcription coactivator activity	1/223	46/29370	0.2959251	0.4717832	0.4640491	gene_Car0335460	1
GO:0007268	chemical synaptic transmission	1/223	46/29370	0.2959251	0.4717832	0.4640491	gene_Car0334650	1
GO:0016849	phosphorus-oxygen lyase activity	1/223	46/29370	0.2959251	0.4717832	0.4640491	gene_Car0335410	1
GO:0000287	magnesium ion binding	2/223	147/29370	0.3071355	0.4717832	0.4640491	gene_Car0334510/gene_Car0336170	2
GO:0003729	mRNA binding	1/223	49/29370	0.3118666	0.4717832	0.4640491	gene_Car0336160	1
GO:0004722	protein serine/threonine phosphatase activity	1/223	49/29370	0.3118666	0.4717832	0.4640491	gene_Car0336170	1
GO:0006364	rRNA processing	1/223	49/29370	0.3118666	0.4717832	0.4640491	gene_Car0334390	1
GO:0016592	mediator complex	1/223	49/29370	0.3118666	0.4717832	0.4640491	gene_Car0334160	1
GO:0019001	guanyl nucleotide binding	1/223	49/29370	0.3118666	0.4717832	0.4640491	gene_Car0335540	1
GO:0031683	G-protein beta/gamma-subunit complex binding	1/223	50/29370	0.3171002	0.4717832	0.4640491	gene_Car0335540	1
GO:0032012	regulation of ARF protein signal transduction	1/223	50/29370	0.3171002	0.4717832	0.4640491	gene_Car0334790	1
GO:0036211	protein modification process	1/223	50/29370	0.3171002	0.4717832	0.4640491	gene_Car0336460	1
GO:0043130	ubiquitin binding	1/223	50/29370	0.3171002	0.4717832	0.4640491	gene_Car0334940	1
GO:0005249	voltage-gated potassium channel activity	2/223	152/29370	0.3210755	0.4738453	0.4660773	gene_Car0334030/gene_Car0336130	2
GO:0005179	hormone activity	2/223	155/29370	0.3294074	0.4822524	0.4743466	gene_Car0333940/gene_Car0335630	2
GO:0009190	cyclic nucleotide biosynthetic process	1/223	54/29370	0.3376412	0.4903837	0.4823446	gene_Car0335410	1
GO:0003678	DNA helicase activity	1/223	56/29370	0.3476799	0.4964118	0.4882739	gene_Car0335490	1

GO:0007275	multicellular organism development	2/223	163/29370	0.3514809	0.4964118	0.4882739	gene_Car0333550/gene_Car0335730	2
GO:0004553	hydrolase activity, hydrolyzing O-glycosyl compounds	1/223	57/29370	0.3526423	0.4964118	0.4882739	gene_Car0336380	1
GO:0008373	sialyltransferase activity	1/223	57/29370	0.3526423	0.4964118	0.4882739	gene_Car0335640	1
GO:0004970	ionotropic glutamate receptor activity	1/223	61/29370	0.3721188	0.515892	0.5074348	gene_Car0334290	1
GO:0140658	ATP-dependent chromatin remodeler activity	1/223	61/29370	0.3721188	0.515892	0.5074348	gene_Car0336450	1
GO:0016791	phosphatase activity	3/223	293/29370	0.384417	0.5289347	0.5202636	gene_Car0334750/gene_Car0334780/gene_Car0335030	3
GO:0003755	peptidyl-prolyl cis-trans isomerase activity	1/223	68/29370	0.4048094	0.5487416	0.5397459	gene_Car0334470	1
GO:0005667	transcription regulator complex	1/223	68/29370	0.4048094	0.5487416	0.5397459	gene_Car0336210	1
GO:0007264	small GTPase mediated signal transduction	2/223	185/29370	0.4107421	0.5526897	0.5436292	gene_Car0333710/gene_Car0336110	2
GO:0006816	calcium ion transport	1/223	70/29370	0.4138344	0.552786	0.543724	gene_Car0334350	1
GO:0015276	ligand-gated monoatomic ion channel activity	1/223	72/29370	0.4227231	0.5540333	0.5449508	gene_Car0334290	1
GO:0004222	metalloendopeptidase activity	2/223	190/29370	0.4238506	0.5540333	0.5449508	gene_Car0333900/gene_Car0334450	2
GO:0007156	homophilic cell adhesion via plasma membrane adhesion molecules	2/223	190/29370	0.4238506	0.5540333	0.5449508	gene_Car0333570/gene_Car0333580	2
GO:0004674	protein serine/threonine kinase activity	3/223	317/29370	0.4330674	0.5616362	0.5524291	gene_Car0333540/gene_Car0333920/gene_Car0336200	3
GO:0005215	transporter activity	1/223	75/29370	0.4358052	0.5616362	0.5524291	gene_Car0334350	1
GO:0005516	calmodulin binding	1/223	77/29370	0.4443622	0.5681381	0.5588243	gene_Car0333920	1
GO:0005840	ribosome	2/223	199/29370	0.4470595	0.5681381	0.5588243	gene_Car0334630/gene_Car0335700	2
GO:0008017	microtubule binding	2/223	203/29370	0.4572066	0.5770262	0.5675668	gene_Car0334640/gene_Car0336320	2
GO:0000398	mRNA splicing, via spliceosome	1/223	82/29370	0.4651936	0.5830852	0.5735264	gene_Car0335390	1
GO:0003777	microtubule motor activity	1/223	84/29370	0.4733068	0.5892187	0.5795594	gene_Car0334640	1
GO:0006412	translation	2/223	212/29370	0.4796397	0.5930681	0.5833456	gene_Car0334630/gene_Car0335700	2
GO:0008076	voltage-gated potassium channel complex	1/223	87/29370	0.4852475	0.5959751	0.586205	gene_Car0336130	1
GO:0016055	Wnt signaling pathway	1/223	88/29370	0.4891675	0.5967843	0.587001	gene_Car0335730	1
GO:0016491	oxidoreductase activity	3/223	356/29370	0.5088157	0.6166442	0.6065353	gene_Car0334700/gene_Car0334740/gene_Car0336220	3
GO:0005783	endoplasmic reticulum	1/223	96/29370	0.5194763	0.6252648	0.6150145	gene_Car0334090	1
GO:0003779	actin binding	2/223	230/29370	0.5227623	0.6252648	0.6150145	gene_Car0335870/gene_Car0335980	2
GO:0003735	structural constituent of ribosome	2/223	232/29370	0.5274046	0.6267211	0.616447	gene_Car0334630/gene_Car0335700	2
GO:0016192	vesicle-mediated transport	2/223	234/29370	0.5320164	0.6281226	0.6178255	gene_Car0334830/gene_Car0335600	2
GO:0006351	DNA-templated transcription	1/223	101/29370	0.5375032	0.6305326	0.620196	gene_Car0335800	1
GO:0004252	serine-type endopeptidase activity	2/223	239/29370	0.5434117	0.6306829	0.6203438	gene_Car0334230/gene_Car0335120	2
GO:0006396	RNA processing	1/223	103/29370	0.544524	0.6306829	0.6203438	gene_Car0334390	1
GO:0006470	protein dephosphorylation	2/223	242/29370	0.5501562	0.6331986	0.6228183	gene_Car0334750/gene_Car0336170	2
GO:0006813	potassium ion transport	2/223	245/29370	0.5568307	0.6368751	0.6264345	gene_Car0334030/gene_Car0336130	2
GO:0016787	hydrolase activity	2/223	248/29370	0.563435	0.6377794	0.627324	gene_Car0334080/gene_Car0334340	2
GO:0005102	signaling receptor binding	1/223	110/29370	0.5682716	0.6377794	0.627324	gene_Car0335730	1
GO:0007169	transmembrane receptor protein tyrosine kinase signaling pathway	1/223	110/29370	0.5682716	0.6377794	0.627324	gene_Car0334040	1
GO:0004497	monooxygenase activity	1/223	111/29370	0.5715619	0.6377794	0.627324	gene_Car0333800	1
GO:0016301	kinase activity	1/223	115/29370	0.5844755	0.6482365	0.6376096	gene_Car0333590	1
GO:0061630	ubiquitin protein ligase activity	1/223	126/29370	0.6180243	0.6795266	0.6683868	gene_Car0335140	1
GO:0007018	microtubule-based movement	1/223	127/29370	0.6209371	0.6795266	0.6683868	gene_Car0334640	1
GO:0004725	protein tyrosine phosphatase activity	1/223	128/29370	0.6238277	0.6795266	0.6683868	gene_Car0334750	1
GO:0005216	monoatomic ion channel activity	3/223	466/29370	0.6894663	0.7451761	0.7329601	gene_Car0334030/gene_Car0334290/gene_Car0336130	3
GO:0005737	cytoplasm	3/223	468/29370	0.6922401	0.7451761	0.7329601	gene_Car0333960/gene_Car0335330/gene_Car0336210	3
GO:0006357	regulation of transcription by RNA polymerase II	2/223	319/29370	0.6991672	0.7482315	0.7359655	gene_Car0335380/gene_Car0336500	2
GO:0015074	DNA integration	1/223	161/29370	0.7078447	0.7531139	0.7407677	gene_Car0334540	1
GO:0008289	lipid binding	1/223	164/29370	0.7144855	0.7557852	0.7433953	gene_Car0334980	1
GO:0006486	protein glycosylation	1/223	166/29370	0.718829	0.7560098	0.7436162	gene_Car0335640	1
GO:0005886	plasma membrane	3/223	496/29370	0.7291367	0.7624686	0.7499691	gene_Car0333570/gene_Car0333580/gene_Car0334650	3
GO:0006511	ubiquitin-dependent protein catabolic process	1/223	186/29370	0.7588044	0.7883231	0.7753998	gene_Car0334800	1
GO:0000166	nucleotide binding	1/223	188/29370	0.7624764	0.7883231	0.7753998	gene_Car0334350	1
GO:0005085	guanyl-nucleotide exchange factor activity	2/223	372/29370	0.7761682	0.7948135	0.7817837	gene_Car0334790/gene_Car0335750	2
GO:0007155	cell adhesion	2/223	373/29370	0.7774405	0.7948135	0.7817837	gene_Car0333570/gene_Car0333580	2
GO:0004713	protein tyrosine kinase activity	1/223	204/29370	0.7899179	0.8030832	0.7899179	gene_Car0334040	1
GO:0006955	immune response	1/223	208/29370	0.79627	0.8050685	0.7918707	gene_Car0334150	1
GO:0016311	dephosphorylation	1/223	221/29370	0.8156229	0.8201044	0.80666	gene_Car0334750	1
GO:0005975	carbohydrate metabolic process	1/223	251/29370	0.8535806	0.8535806	0.8395875	gene_Car0336380	1

Supplementary Table 15. Antibodies used in this study.

Name	Supplier	Catalog no	Host	Type	Dilution
Anti-Sycp1	Homemade	This work	Mouse	polyclonal	1:100
Anti-Sycp1	Homemade	This work	Guinea pig	polyclonal	1:100
Anti-Sycp3	Homemade	This work	Mouse	polyclonal	1:100
Anti-Sycp3	Homemade	This work	Rabbit	polyclonal	1:100
Anti-γH2AX	Abcam	ab228655	Rabbit	polyclonal	1:50
Anti-Rad51	Abcam	ab88572	Mouse	polyclonal	1:100
Alexa Fluor 647 Goat anti-Guinea IgG secondary Antibody	Thermo-Fisher	A-21450	Goat	polyclonal	1:300
Alexa Fluor 555 Goat anti-Mouse IgG secondary Antibody	Thermo-Fisher	A-21422	Goat	polyclonal	1:300
Alexa Fluor 488 Goat anti-Rabbit IgG secondary Antibody	Thermo-Fisher	A-11008	Goat	polyclonal	1:300

Supplementary Table 16. Statistics of the mapping rate in two reference genomes.

Sample	The mixed genome dataset of *C. cuvieri*, *C. gibelio*, and *C. auratus* (**Reference genome-1**)			The mixed genome dataset of *C. cuvieri* and *C. gibelio* (**Reference genome-2**)	
	C. cuvieri genome	*C. gibelio* genome	*C. auratus* genome	*C. cuvieri* genome	*C. gibelio* genome
C. auratus transcriptome reads	19.1%	35.2%	45.7%	20.0%	80.0%
C. gibelio transcriptome reads	12.9%	48.7%	38.4%	18.9%	81.1%
C. cuvieri transcriptome reads	76.1%	12.4%	11.5%	84.3%	15.7%

Supplementary Table 17-1. The weights of *C. cuvieri* and co-cultured *C. gibelio* clone-A^{+} in the first pond.

C. cuvieri		*C. gibelio* clone-A^{+}	
Samples	Weights (g)	Samples	Weights (g)
1	378.5	1	454.3
2	90.4	2	496.6
3	216.8	3	379.4
4	325.4	4	433.4
5	235.6	5	367.2
6	260.8	6	470.3
7	311.3	7	463.4
8	249.0	8	472.1
9	147.5	9	428.5
10	245.5	10	405.0
11	178.2	11	465.7
12	335.5	12	331.4
13	205.9	13	368.5
14	410.4	14	394.9
15	242.0	15	390.6
16	296.8	16	493.0
17	228.1	17	346.6
18	289.1	18	427.0
19	261.5	19	432.2
20	259.7	20	299.1
21	240.9	21	404.6
22	145.3	22	334.8
23	241.3	23	401.0
24	224.0	24	388.5
25	114.3	25	472.4
26	77.8	26	425.1
27	223.9	27	392.5
28	259.5	28	415.6
29	206.3	29	311.5
30	394.1	30	447.5
Average	243.2		410.4

Supplementary Table 17-2. The weights of GR-A3n clone-1 and co-cultured *C. gibelio* clone-A+ in the second pond.

GR-A3n clone-1		*C. gibelio* clone-A+	
Samples	Weights (g)	Samples	Weights (g)
1	173.7	1	218.9
2	159.0	2	212.8
3	167.7	3	224.7
4	158.2	4	213.2
5	181.6	5	178.8
6	171.8	6	194.0
7	162.8	7	209.4
8	193.2	8	172.6
9	160.2	9	189.9
10	153.6	10	224.4
11	147.1	11	147.3
12	181.7	12	169.7
13	187.9	13	203.3
14	153.2	14	176.9
15	169.0	15	208.9
16	153.6	16	167.6
17	166.6	17	187.9
18	176.3	18	235.2
19	149.9	19	159.7
20	176.8	20	190.3
21	149.9	21	127.9
22	155.9	22	190.7
23	161.3	23	146.4
24	147.1	24	172.9
25	140.0	25	180.4
26	188.0	26	204.8
27	183.5	27	137.5
28	140.0	28	178.6
29	200.0	29	180.3
30	194.1	30	181.2
31	185.9	31	200.0
32	156.7	32	170.5
33	180.7	33	136.0
34	179.4	34	122.3
35	187.6	35	178.8
36	164.3	36	198.7
37	195.7	37	146.7
38	195.7	38	178.9
39	200.0	39	190.9
40	179.2	40	152.3
41	159.9	41	146.6
42	157.9	42	178.5
43	173.5	43	188.4
44	157.7	44	152.1
45	159.5	45	170.2
46	167.7	46	171.7
47	171.2	47	174.1
48	175.9	48	162.7
49	166.0	49	181.9
50	131.9	50	225.3
Average	169.0		180.3

Supplementary Table 17-3. The weights of GR-A3n clone-2 and co-cultured *C. gibelio* clone-A$^+$ in the third pond.

GR-A3n clone-2		*C. gibelio* clone-A$^+$	
Samples	Weights (g)	Samples	Weights (g)
1	301.6	1	197.0
2	279.4	2	193.8
3	302.1	3	214.4
4	305.9	4	226.6
5	276.4	5	290.2
6	232.6	6	197.3
7	269.5	7	217.2
8	258.3	8	274.9
9	280.1	9	177.1
10	289.3	10	262.0
11	294.4	11	314.0
12	285.3	12	272.8
13	257.4	13	282.7
14	270.6	14	264.4
15	264.6	15	189.5
16	272.4	16	244.4
17	271.4	17	242.2
18	318.6	18	264.9
19	246.1	19	295.6
20	183.1	20	316.0
21	283.0	21	272.5
22	294.0	22	293.8
23	175.1	23	293.0
24	294.3	24	214.1
25	278.1	25	253.7
26	255.1	26	187.5
27	190.1	27	214.5
28	283.9	28	190.6
29	284.5	29	222.8
30	269.2	30	176.1
31	271.7	31	201.5
32	278.5	32	247.6
33	275.4	33	220.0
34	306.9	34	200.4
35	280.0	35	205.4
36	291.5		
37	283.3		
38	270.9		
39	283.2		
40	263.9		
41	288.4		
42	279.1		
43	224.8		
44	252.1		
45	301.7		
46	238.8		
47	260.9		
48	275.8		
49	224.1		
50	288.4		
Average	270.1		238.0

Supplementary Table 17-4. The weights of GR-A3n clone-3 and co-cultured *C. gibelio* clone-A$^+$ in the fourth pond.

GR-A3n clone-3		*C. gibelio* clone-A$^+$	
Samples	Weights (g)	Samples	Weights (g)
1	357.2	1	390.9
2	344.6	2	326.1
3	250.2	3	433.9
4	349.9	4	405.3
5	337.0	5	436.0
6	316.2	6	476.6
7	270.0	7	453.8
8	282.2	8	273.2
9	330.9	9	340.0
10	356.9	10	422.8
11	335.3	11	453.7
12	278.3	12	454.6
13	373.6	13	419.1
14	287.8	14	245.2
15	362.4	15	436.1
16	246.0	16	386.9
17	327.5	17	399.5
18	385.1	18	420.6
19	263.6	19	409.6
20	326.2	20	442.9
21	348.0	21	316.9
22	366.2	22	368.1
23	318.2	23	405.5
24	310.4	24	418.4
25	353.0	25	466.2
26	356.0	26	443.8
27	377.5	27	381.2
28	321.7	28	411.3
29	367.3	29	331.3
30	377.3	30	435.8
31	286.2		
32	334.5		
33	327.1		
34	370.5		
35	378.0		
36	323.4		
37	340.0		
38	361.2		
39	374.5		
Average	332.6		400.2
